# Annotated checklist of fish cestodes from South America

**DOI:** 10.3897/zookeys.650.10982

**Published:** 2017-02-01

**Authors:** Philippe V. Alves, Alain de Chambrier, Tomáš Scholz, José L. Luque

**Affiliations:** 1 Programa de Pós-Graduação em Biologia Animal, Universidade Federal Rural do Rio de Janeiro, BR 465, Km 7, 23851-970, Seropédica, Rio de Janeiro, Brazil; 2 Natural History Museum of Geneva, CP 6434, CH - 1211 Geneva 6, Switzerland; 3 Institute of Parasitology, Biology Centre of the Czech Academy of Sciences, Branišovská 31, 370 05 České Budějovice, Czech Republic; 4 Departamento de Parasitologia Animal, Universidade Federal Rural do Rio de Janeiro, CP 74.540, BR 465, Km 7, 23851-970, Seropédica, Rio de Janeiro, Brazil

**Keywords:** Biodiversity, marine ecoregions, river basins, species richness, tapeworms

## Abstract

An exhaustive literature search supplemented by a critical examination of records made it possible to present an annotated checklist of tapeworms (Cestoda) that, as adults or larvae (metacestodes), parasitize freshwater, brackish water and marine fishes, i.e. cartilaginous and bony fishes, in South America. The current knowledge of their species diversity, host associations and geographical distribution is reviewed. Taxonomic problems are discussed based on a critical evaluation of the literature and information on DNA sequences of individual taxa is provided to facilitate future taxonomic and phylogenetic studies. As expected, the current knowledge is quite uneven regarding the number of taxa and host-associations reported from the principal river basins and marine ecoregions. These differences may not only reflect the actual cestode richness but may also be due to the research effort that has been devoted to unravelling the diversity of these endoparasitic helminths in individual countries. A total of 297 valid species, 61 taxa identified to the generic level, in addition to unidentified cestodes, were recorded from 401 species of fish hosts. Among the recognized cestode orders, 13 have been recorded in South America, with the Onchoproteocephalidea displaying the highest species richness, representing *c.* 50% of all species diversity. The majority of records include teleost fish hosts (79%) that harbour larval and adult stages of cestodes, whereas stingrays (Myliobatiformes) exhibit the highest proportion of records (39%) among the elasmobranch hosts. Fish cestodes are ubiquitous in South America, being mostly recorded from the Warm Temperate Southeastern Pacific (WTSP; 31%) for marine hosts and the Amazon River basin (45%) for freshwater ones. The following problems were detected during the compilation of literary data: (i) unreliability of many records; (ii) poor taxonomic resolution, i.e. identification made only to the genus or even family level; (iii) doubtful host identification; and (iv) the absence of voucher specimens that would enable us to verify identification. It is thus strongly recommended to always deposit representative specimens in any type of studies, including faunal surveys and ecological studies. An analysis of the proportion of three basic types of studies, i.e. surveys, taxonomic and ecological papers, has shown a considerable increase of ecological studies over the last decade.

## Introduction

Tapeworms (Cestoda) are a monophyletic assemblage of flatworms (Phylum Platyhelminthes) and they are obligate internal parasites of vertebrates. Their complex life-cycles include one or more intermediate hosts in a wide array of animal phyla (mostly arthropods) and they are exclusively transmitted perorally, i.e. via the food chain (Caira and Littlewood 2013; Littlewood et al. 2015). The cestodes are the second species-richest group of platyhelminths, with more than 5000 species in 751 recognized genera that have radiated through marine, freshwater and terrestrial environments (Waeschenbach et al. 2012; Caira and Littlewood 2013).

Cestodes parasitizing elasmobranchs and teleost fishes in at least one stage of development comprise one of the most diverse lineages of tapeworms (Caira et al. 2014; Caira and Jensen 2014), only comparable in species richness with cyclophyllidean cestodes, parasites of tetrapods (Caira and Littlewood 2013). Since these parasites usually exhibit a strict host specificity, they are considered suitable models for studies of host-parasite co-evolution (Caira and Jensen 2014) or even helping in circumscribing species boundaries of cestode hosts (Caira and Jensen 2015).

South America is a megadiverse continent, including at least five of the world’s biodiversity ‘hotspots’, more than 30.000 km of coastline and two of the 10 largest freshwater drainage systems of the world, i.e. the Amazon and Paraná River basins, which is reflected in its species-rich ichthyofauna (Myers et al. 2000; Miloslavich et al. 2011; Reis 2013). Bearing that in mind, one might expect a high diversity of fish cestodes as well, even though there are no comprehensive checklists or other faunistic studies encompassing the whole continent that could provide an overview of the cestode diversity, except those with regional focus (e.g. Thatcher 2006 for Amazonia; Tantaléan and Huiza 1994 for Peru; Muñoz and Olmos 2008 for Chile).

Studies on fish cestodes from South America date back to the early 19th Century, when C. A. Rudolphi described *Anthocephalus macrourus* Rudolphi, 1819 from an unidentified sparid fish and *Anthocephalus interruptum* Rudolphi, 1819 (both cestodes of the order Trypanorhyncha) from *Trichiurus lepturus* Linnaeus off the Brazilian coast, even though these species are no longer valid (Campbell and Beveridge 1996). Subsequently, K. M. Diesing (1850, 1856) described several species that are now included in three different orders. Both workers studied cestodes collected by renowned naturalists, such as F. Sellow, I.F.W.M. Von Olfers and J. Natterer. With few exceptions, the number of descriptions and/or reports have considerable increased in the 20th Century and a large amount of information has been generated, yet many studies are faunal surveys dispersed in regional journals that are not readily accessible.

Detailed taxonomic studies combining morphological and molecular approaches have recently expanded our knowledge at lower and higher taxonomic levels, mostly under the framework of the National Science Foundation (Planetary Biodiversity Inventory program) funded project called “A survey of the tapeworms (Cestoda: Platyhelminthes) from vertebrate bowels of the earth” (see http://tapewormdb.uconn.edu). This project funded, amongst others, intensive research on fish cestodes in South America, which were mainly undertaken by A. de Chambrier, T. Scholz and A. A. Gil de Pertierra for teleost hosts, and V. A. Ivanov, F. P. L. Marques and F. Reyda for elasmobranch hosts. The present paper aims at addressing the following objectives: (1) to provide for the first time an annotated checklist that summarizes records of cestodes in marine and freshwater fishes from South America, including detailed information on their hosts, site of infection, geographical distribution, stage of development and molecular data; (2) to critically assess some doubtful reports; and (3) to depict the problems that impede a better understanding of the diversity and host associations of cestodes in South America.

## Materials and methods

Parasite-host and host-parasite checklists for fish cestodes from South America were compiled on the basis of an exhaustive search of literature published until August 2016; abstracts of meetings, theses and reports without primary data were not considered. The bibliographic search was complemented by the information gathered from *Helminthological Abstracts*, Host-Parasite Database of the Natural History Museum, London (Gibson et al. 2005), Global Cestode Database (GDC) (Caira et al. 2012), *Google Scholar*, *ScienceDirect*, *Web of Knowledge*, as well as some previously published books (e.g. Palm 2004; Thatcher 2006). The classification of cestodes proposed by Khalil et al. (1994) is basically followed, but it is updated based on revisional papers on individual cestode orders or molecular phylogenetic studies at the ordinal level, such as Kuchta et al. (2008) for bothriocephalideans and diphyllobothriideans, Olson et al. (2010) for trypanorhynchs, Caira et al. (2014) for onchoproteocephalideans, phyllobothriideans and ‘tetraphyllideans’, Healy et al. (2009) and Ruhnke et al. (2015) for rhinebothriideans, and Jensen et al. (2016) for lecanicephalideans.

The species are arranged according to taxonomic categories and are presented in alphabetical order followed by data on their hosts (species name, class and family), habitat, site of infection, stage of development, marine ecoregion according to Spalding et al. (2007), river basins or lakes, country and references (between parentheses). All cestodes presented herein follow the most recent taxonomic literature and the validity of individual taxa or the reliability of their records were critically assessed by the present authors, who consulted with experts for some tapeworm groups.

Host species are arranged in taxonomic and then alphabetical order. The scientific names of hosts have been updated based on Froese and Pauly (2016) and supplemented by the most recent taxonomic papers for certain problematic taxa (e.g. *Cichla* Bloch & Schneider, *Pseudoplatystoma* Bleeker and *Zungaro* Bleeker).

The following abbreviations are used for collections:

BMNH
 The British Museum (Natural History) Collection at the Natural History Museum, London, UK 


CHIOC
 Coleção Helmintológica do Instituto Oswaldo Cruz, Rio de Janeiro, Brazil 


HWML
 Harold W. Manter Laboratory of Parasitology, University of Nebraska State Museum, Lincoln, Nebraska 


MLP
 Departamento de Zoología de Invertebrados (Parasitología), La Plata, Argentina 


MHNG
Natural History Museum, Geneva, Switzerland 


MNHB
 Museum der Naturkunde für Humboldt Universität zu Berlin, Germany 


NHMW
Natural History Museum, Vienna, Austria 


USNPC
United States National Parasite Collection, Beltsville, Maryland, USA , which has been transferred recently to the National Museum of Natural History
(USNM) of the Smithsonian Institution, Washington, D.C., USA

The following abbreviations are used for marine ecoregions according to Spalding et al. (2007):

JFD
 Juan Fernández and Desventuradas 


NBS
 North Brazil Shelf 


TEP
 Tropical Eastern Pacific 


TNA
 Tropical Northwestern Atlantic 


TSA
 Tropical Southwestern Atlantic 


WTSA
 Warm Temperate Southwestern Atlantic 


WTSP
 Warm Temperate Southeastern Pacific 


The following abbreviations are used for molecular markers:

18S
 small subunit of the nuclear ribosomal RNA gene 


ITS1
 first nuclear ribosomal internal transcribed spacer 


5.8S
 5.8S ribosomal RNA gene 


ITS2
 second nuclear ribosomal internal transcribed spacer 


28S
 large subunit of the nuclear ribosomal RNA gene 


16S
 large subunit of the mitochondrial ribosomal RNA gene 


cox1
 cytochrome *c* oxidase I


The following abbreviation is used for records of metacestodes in the host-parasite list:

L
 larvae 


* Asterisks in the parasite-host list indicate the type species of the genus.

## Parasite-Host List

### Class Cestoda Rudolphi, 1808

#### Order Amphilinidea Poche, 1922

##### Family Amphilinidae Claus, 1879

*Nesolecithus janickii* Poche, 1922*

[Syns. *Monostoma liguloideum* Diesing, 1850 (*pro parte*); *Amphilina liguloidea* Monticelli, 1892 sensu Janicki (1808); *Schizochoerus janickii* (Poche, 1922) Bandoni & Brooks, 1987]

*Arapaima gigas* (Actinopterygii: Arapaimidae); freshwater; body cavity; adult; Amazon River basin; Brazil (Janicki 1908; Poche 1922; Travassos and Teixeira de Freitas 1964; Rego et al. 1974; Araújo et al. 2009).

Notes: type host. Bandoni and Brooks (1987) proposed *Schizochoerus janickii* as a new combination for this species, but we are following the classification proposed by Gibson (1994).

*Schizochoerus liguloideus* (Diesing, 1850) Poche, 1922*

[Syns. *Monostoma liguloideum* Diesing, 1850 (*pro parte*); *Amphilina liguloidea* Monticelli, 1892]

*Arapaima gigas* (Actinopterygii: Arapaimidae); freshwater; body cavity; adult; Amazon River basin; Brazil, Peru (Diesing 1850; Poche 1922; Travassos and Teixeira de Freitas 1964; Rego et al. 1974; Mariaux 1998; Olson and Caira 1999; Araújo et al. 2009; Serrano-Martínez et al. 2015).

Notes: type host. Diesing (1850) described *Monostoma liguloideum* based on a mixture of *Schizochoerus liguloideus* and *Nesolecithus janickii*. Monticelli (1892) transferred the former species to *Amphilina* Wagener, 1858, whereas the latter was described by Janicki (1908) also under the name *Amphilina liguloidea*. Poche (1922) distinguished the both species and proposed the name *Nesolecithus janickii*. Sequences of partial 18S (Z98305, Z98306, Z98307, AF124454) (Mariaux 1998; Olson and Caira 1999).

#### Order Bothriocephalidea Kuchta, Scholz, Brabec & Bray, 2008

##### Family Bothriocephalidae Blanchard, 1849

***Bothriocephalus timii* Gil de Pertierra, Arredondo, Kuchta & Incorvaia, 2015**

*Cottoperca gobio* (Actinopterygii: Bovichtidae); marine; intestine, pyloric caeca; adult; Magellanic; Argentina (Brabec et al. 2015; Gil de Pertierra et al. 2015).

Notes: type host. Sequences of 18S (KR780929), 28S (KR780885), 16S (KR780839) and *cox*1 (KR780787) (Brabec et al. 2015).

***Bothriocephalus* sp.**

*Eleginops maclovinus* (Actinopterygii: Eleginopsidae); marine; intestine; adult; WTSP; Chile (George-Nascimento et al. 2009).

*Engraulis anchoita* (Actinopterygii: Engraulidae); marine; pyloric caeca; adult; Magellanic, WTSA; Argentina (Timi 2003; Timi and Poulin 2003).

*Engraulis ringens* (Actinopterygii: Engraulidae); marine; intestine; adult; WTSP; Chile (Chávez et al. 2007; Valdivia et al. 2007; George-Nascimento and Moscoso 2013).

*Helicolenus lengerichi* (Actinopterygii: Sebastidae); marine; intestine; adult; WTSP; Chile (George-Nascimento and Iriarte 1989; Balboa and George-Nascimento 1998).

*Clestobothrium crassiceps* (Rudolphi, 1819) Lühe, 1899*

[Syn. *Bothriocephalus crassiceps* Rudolphi, 1819]

*Aphos porosus* (Actinopterygii: Batrachoididae); marine; intestine; adult (immature); WTSP; Chile (Cortés and Muñoz 2008, 2009).

*Dissostichus eleginoides* (Actinopterygii: Nototheniidae); marine; intestine, pyloric caeca; stage of development not given; Magellanic; Falkland Islands (Brickle et al. 2006).

Note: R. Kuchta (pers. comm.) suggested that this report might be wrong.

*Macruronus magellanicus* (Actinopterygii: Merlucciidae); marine; intestine;

adult; Magellanic; Argentina, Chile (Szidat 1961; Oliva 2001; Chávez et al. 2012).

*Merluccius gayi gayi* (Actinopterygii: Merlucciidae); marine; intestine; adult; WTSP; Chile (Carvajal et al. 1979; George-Nascimento 1996; Oliva and Ballón 2002; Chávez et al. 2012).

Note: Carvajal et al. (1979) reported the species as *Clestobothrium* sp., and R. Kuchta (pers. comm.) suggested that it belongs to *Clestobothrium crassiceps*.

*Merluccius gayi peruanus* (Actinopterygii: Merlucciidae); marine; intestine; adult; WTSP; Peru (Durán and Oliva 1980; Jara 1998; Chero et al. 2014a).

*Merluccius* sp. (Actinopterygii: Merlucciidae); marine; intestine; adult; Magellanic, WTSP; Argentina, Chile (Oliva 1982; Gil de Pertierra et al. 2011).

Note: Gil de Pertierra et al. (2011) studied the vouchers deposited in BMNH; they reported the species as *Clestobothrium* sp., and R. Kuchta (pers. comm.) suggested that it belongs to *Clestobothrium crassiceps*.

*Micromesistius australis australis* (Actinopterygii: Gadidae); marine; intestine; adult; Magellanic; Chile (Chávez et al. 2012).

***Clestobothrium cristinae* Gil de Pertierra, Incorvaia & Arrendondo, 2011**

*Merluccius hubbsi* (Actinopterygii: Merlucciidae); marine; intestine; adult; Magellanic; Argentina (Szidat 1955, 1961; MacKenzie and Longshaw 1995; Sardella and Timi 1996, 2004; Gil de Pertierra et al. 2011; Brabec et al. 2015).

Notes: type host. R. Kuchta (pers. comm.) suggested that all reports from *Merluccius hubbsi* before the original description of *Clestobothrium cristinae* were erroneously reported as *Clestobothrium crassiceps*. Sequences of 18S (KR780948), 28S (KR780901), 16S (KR780862) and *cox*1 (KR7808301) (Brabec et al. 2015).

***Clestobothrium splendidum* Gil de Pertierra, Incorvaia & Arredondo, 2011**

*Merluccius australis* (Actinopterygii: Merlucciidae); marine; intestine; adult; Magellanic; Argentina, Chile (Fernández 1985; George-Nascimento and Arancibia 1994; González and Carvajal 1994; MacKenzie and Longshaw 1995; Gil de Pertierra et al. 2011; Chávez et al. 2012; Brabec et al. 2015).

Notes: type host. R. Kuchta (pers. comm.) suggested that all reports from *Merluccius australis* before the original description of *Clestobothrium splendidum* as well as that of Chávez et al. (2012) were erroneously reported as *Clestobothrium crassiceps*. Sequences of 18S (KR780967), 28S (KR780920), 16S (KR780877) and *cox*1 KR7808271 (Brabec et al. 2015).

*Schyzocotyle acheilognathi* (Yamaguti, 1934) Brabec, Waeschenbach, Scholz, Littlewood & Kuchta, 2015*

[For synonyms, see Kuchta and Scholz (2007) and Brabec et al. (2015)]

*Cyprinus carpio* (Actinopterygii: Cyprinidae); freshwater; intestine; adult; Paraná State (fishpond), Negro River basin; Argentina, Brazil (Rego et al. 1999a; Waicheim et al. 2014).

Notes: these reports from South America are probably result of the import of common carp from Europe to Brazil (Rego et al. 1999a; Scholz et al. 2011).

Waicheim et al. (2014) reported the cestode as *Bothriocephalus* sp., but it was most probably *Schyzocotyle acheilognathi* (R. Kuchta, pers. comm.).

*Pethia conchonius* (Actinopterygii: Cyprinidae); freshwater; intestine; adult; Santa Catarina State; Brazil (Piazza et al. 2006).

Notes: host reported as *Puntius conchonius*.

*Poecilia reticulata* (Actinopterygii: Poeciliidae); freshwater; intestine; adult; Paraná River Basin; Brazil (Moreira et al. 2014).

Note: tapeworms reported as ‘Pseudophyllidea’, but considered as *Schyzocotyle acheilognathi* by R. Kuchta (pers. comm.).

*Xiphophorus hellerii* (Actinopterygii: Poeciliidae); freshwater; intestine; adult; Santa Catarina State; Brazil (Piazza et al. 2006).

*Xiphophorus maculatus* (Actinopterygii: Poeciliidae); freshwater; intestine; adult; Santa Catarina State; Brazil (Piazza et al. 2006)

Notes: *Poecilia conchonius*, *Xiphophorus hellerii* and *Xiphophorus maculatus* are ornamental fish imported to South America (Froese and Pauly 2016); their tapeworms were reported as ‘cestodes’, but they were most probably conspecific with *Schyzocotyle acheilognathi* (R. Kuchta, pers. comm.).

***Senga* sp.**

*Astyanax altiparanae* (Actinopterygii: Characidae); freshwater; pyloric caeca; adult; Rio das Pedras Farm (lakes); Brazil (Azevedo et al. 2007).

*Astyanax scabripinnis* (Actinopterygii: Characidae); freshwater; intestine; adult; São Paulo State; Brazil (Rego 1997).

**Unidentified bothriocephalid cestode**

*Girella laevifrons* (Actinopterygii: Kyphosidae); marine; site of infection not given; adult; WTSP; Chile (Muñoz and Delorme 2011).

##### Family Echinophallidae Schumacher, 1914

*Neobothriocephalus aspinosus* Mateo & Bullock, 1966*

*Seriolella violacea* (Actinopterygii: Centrolophidae); marine; intestine, stomach; adult; WTSP; Chile, Peru (Mateo and Bullock 1966; Soto and Carvajal 1979; Oliva 1982; Iannacone 2003; Brabec et al. 2015).

Notes: type host; it was originally reported as *Neptomenus crassus*. Sequences of 18S (KR780944), 28S (KR780897), 16S (KR780857) and *cox*1 (KR780805) (Brabec et al. 2015).

***Neobothriocephalus* sp.**

*Hippoglossina macrops* (Actinopterygii: Paralichthyidae); marine; intestine; adult; WTSP; Chile (Riffo 1991; González et al. 2001; Oliva et al. 2004; González et al. 2008).

Note: all but one authors reported the cestode as *Neobothriocephalus aspinosus*. Kuchta et al. (2008) stated that further analyses should be performed to confirm these records since there is no material deposited in any helminthological collection and cestodes from this fish host may represent a new species.

*Paralichthys adspersus* (Actinopterygii: Paralichthyidae); marine; intestine; adult; WTSP; Chile (Riffo 1995).

*Paralichthys microps* (Actinopterygii: Paralichthyidae); marine; intestine; adult; WTSP; Chile (Riffo 1995).

***Parabothriocephalus* sp.**

*Macrourus holotrachys* (Actinopterygii: Macrouridae); marine; intestine, pyloric caeca; adult; WTSP: Chile (Ñacari and Oliva 2016).

##### Family Triaenophoridae Lönnberg, 1899

*Ailinella mirabilis* Gil de Pertierra & Semenas, 2006*

*Aplochiton zebra* (Actinopterygii: Galaxiidae); amphidromous; intestine; adult; Patagonian lakes; Argentina (Ortubay et al. 1994; Fernández et al. 2012).

Note: Ortubay et al. (1994) misidentified the parasite as *Nippotaenia* sp. (Gil de Pertierra and Semenas 2006; Fernández et al. 2012).

*Galaxias maculatus* (Actinopterygii: Galaxiidae); amphidromous; intestine; adult; Moreno and Nahuel Huapi Lake systems (Andean-Patagonian region); Argentina (Ortubay et al. 1994; Rauque et al. 2003; Revenga et al. 2005; Gil de Pertierra and Semenas 2006; Viozzi et al. 2009; Fernández et al. 2010).

Notes: type host. Ortubay et al. (1994), Rauque et al. (2003) and Revenga et al. (2005) misidentified the parasite as *Nippotaenia* sp. (Gil de Pertierra and Semanas 2006).

*Anchistrocephalus microcephalus* (Rudolphi, 1819) Monticelli, 1890*

[For synonyms, see Kuchta and Scholz (2007)].

*Mola mola* (Actinopterygii: Molidae); marine; intestine; adult; WTSA; Brazil (Mendes 1944).

Notes: type host. The tapeworms were reported as *Amphigonophorus carvalhoi* Mendes, 1944. Kennedy and Andersen (1982) synonymized *Amphigonophorus* with *Anchistrocephalus*, which was rejected by Bray et al. (1994), but accepted by Kuchta and Scholz (2007). We are following the recent revision of bothriocephalideans by Kuchta et al. (2008).

*Mola ramsayi* (Actinopterygii: Molidae); marine; intestine; adult; WTSP; Chile (Villalba and Fernández 1985).

***Anonchocephalus argentinensis* Szidat, 1961**

*Xystreurys rasile* (Actinopterygii: Paralichthyidae); marine; intestine; adult; WTSA; Argentina (Szidat 1961; Alarcos and Timi 2012, 2013).

*Anonchocephalus chilensis* (Riggenbach, 1896) Lühe, 1902*

[Syn. *Bothriotaenia chilensis* Riggenbach, 1896]

*Genypterus blacodes* (Actinopterygii: Ophidiidae); marine; intestine; adult; WTSA, WTSP; Argentina, Chile (Riffo 1994; Sardella et al. 1998; Suriano and Labriola 1998; Brabec et al. 2015).

Notes: Suriano and Labriola (1998) redescribed this species. Sequence of *cox*1 (KR780782) (Brabec et al. 2015).

*Genypterus brasiliensis* (Actinopterygii: Ophidiidae); marine; intestine; adult; WTSA; Argentina, Brazil (Sardella et al. 1998; Pereira 2000).

Note: Pereira (2000) redescribed this species.

*Genypterus chilensis* (Actinopterygii: Ophidiidae); marine; intestine, pyloric caeca; adult; WTSP; Chile (Riggenbach 1896a; Vergara and George-Nascimento 1982).

Note: type host.

*Genypterus maculatus* (Actinopterygii: Ophidiidae); marine; intestine; adult; WTSP; Chile (George-Nascimento and Huet 1984).

***Anonchocephalus patagonicus* Suriano & Labriola, 1998**

*Paralichthys patagonicus* (Actinopterygii: Paralichthyidae); marine; intestine; adult; Magellanic; Argentina (Suriano and Labriola, 1998).

Note: type host.

***Anonchocephalus* sp.**

*Pinguipes brasilianus* (Actinopterygii: Pinguipedidae); marine; intestine; adult; WTSA; Argentina (Timi et al. 2009, 2010a).

*Galaxitaenia toloi* Gil de Pertierra & Semenas, 2005*

*Galaxias platei* (Actinopterygii: Galaxiidae); amphidromous; intestine; adult; Moreno Lake system (Patagonian region); Argentina (Ortubay et al. 1994; Rauque et al. 2003; Gil de Pertierra and Semenas 2005).

Notes: type host. Ortubay et al. (1994) and Rauque et al. (2003) misidentified the parasite as *Nippotaenia* sp. (Gil de Pertierra and Semenas 2006).

**Unidentified bothriocephalideans**

*Aplochiton taeniatus* (Actinopterygii: Galaxiidae); amphidromous; intestine; adult; Patagonian lakes; Argentina (Ortubay et al. 1994).

Note: reported as *Nippotaenia* sp. but the nippotaeniids are not found in the Americas (Bray 1994).

*Cichla monoculus* (Actinopterygii: Cichlidae); freshwater; intestine; adult; Rio das Pedras Farm (lakes); Brazil (Müller et al. 2008).

Note: reported as *Bothriotaenia cuspidatus* and considered as misidentification by R. Kuchta (pers. comm.).

*Odontesthes smitti* (Actinopterygii: Atherinopsidae); marine; intestine; metacestode; Magellanic; Argentina (Carballo et al. 2012).

*Oncorhynchus mykiss* (Actinopterygii: Salmonidae); anadromous; intestine; adult; Moreno and Nahuel Huapi lakes (Patagonian region); Argentina (Rauque et al. 2003).

Note: reported as *Nippotaenia* sp.

*Paralabrax humeralis* (Actinopterygii: Serranidae); marine; intestine; adult; WTSP; Chile (Henríquez and González 2014).

*Percichthys trucha* (Actinopterygii: Percichthyidae); freshwater; intestine; adult; Moreno and Nahuel Huapi lakes (Patagonian region); Argentina (Ortubay et al. 1994; Rauque et al. 2003).

Note: reported as *Nippotaenia* sp.

*Percophis brasiliensis* (Actinopterygii: Percophidae); marine; mesentery; metacestode; WTSA; Argentina (Braicovich and Timi 2010).

*Plagioscion squamosissimus* (Actinopterygii: Sciaenidae); freshwater; intestine; adult; Amazon River basin; Brazil (Woodland 1935c).

Note: host reported as *Percichthys squamosissima* and its tapeworms as an unidentified ptychobothriid (Woodland 1935c), but considered only as Bothriocephalidea by Kuchta and Scholz (2007).

*Salvelinus fontinalis* (Actinopterygii: Salmonidae); anadromous; intestine; adult; Moreno and Nahuel Huapi lakes (Patagonian region); Argentina (Rauque et al. 2003).

Note: reported as *Nippotaenia* sp.

**Unidentified bothriocephalideans (identified as ‘Pseudophyllidea’)**

*Eleginops maclovinus* (Actinopterygii: Eleginopsidae); marine; intestine; adult; Magellanic; Falkland Islands (Brickle and MacKenzie 2007).

*Paralichthys adspersus* (Actinopterygii: Paralichthyidae); marine; intestine; adult; WTSP; Chile (Oliva et al. 1996).

*Prolatilus jugularis* (Actinopterygii: Pinguipedidae); marine; intestine; adult; Magellanic; Chile (Sepúlveda et al. 2004).

#### Order Caryophyllidea van Beneden in Carus, 1863

[Caryophyllidean tapeworms do not occur in the Neotropical region, where their common hosts, i.e. cyprinid and catostomid fishes, are absent; therefore, these reports need verification]

**Unidentified caryophyllideans**

*Cyprinus carpio* (Actinopterygii: Cyprinidae); freshwater; intestine; adult; Paraná State; Brazil (Rego 2004).

Note: introduced fish host (Froese and Pauly 2016).

*Geophagus brasiliensis* (Actinopterygii: Cichlidae); freshwater; intestine; adult; Paraná State (dams); Brazil (Bellay et al. 2012).

#### Order Cathetocephalidea Schmidt & Beveridge, 1990

##### Family Cathetocephalidae Dailey & Overstreet 1973

***Cathetocephalus australis* Schmidt & Beveridge, 1990**

*Carcharhinus brachyurus* (Elasmobranchii: Carcharhinidae); marine; spiral valve; adult; WTSA; Argentina (Suriano and Labriola 2001a).

Note: type host.

*Cathetocephalus thatcheri* Dailey & Overstreet, 1973*

*Carcharhinus leucas* (Elasmobranchii: Carcharhinidae); marine; spiral valve; adult; WTSP; Peru (Rivera and Sarmiento 1990).

Note: type host.

##### Family Disculicepitidae Joyeux & Baer, 1935

***Disculiceps galapagoensis* Nock & Caira, 1988**

*Carcharhinus longimanus* (Elasmobranchii: Carcharhinidae); marine; spiral valve; adult; Galapagos; Ecuador (Nock and Caira 1988).

Note: type host.

*Disculiceps pileatus* (Linton, 1890) Joyeux & Baer, 1936*

[Syn. *Discocephalum pileatum* Linton, 1890]

*Carcharhinus porosus* (Elasmobranchii: Carcharhinidae); marine; spiral valve; adult; WTSP; Peru (Tantaleán 1991).

Note: tapeworms reported as *Discocephalum pileatum*.

***Disculiceps* sp.**

*Aetobatus narinari* (Elasmobranchii: Myliobatidae); marine; spiral valve; adult; TNA; Venezuela (Mayes and Brooks 1981).

Note: these specimens probably belong to *Tylocephalum* (Lecanicephalidea), according to Koch et al. (2012).

#### Order Cyclophyllidea van Beneden in Braun, 1900

##### Family Gryporhynchidae Spassky & Spasskaya, 1973

***Glossocercus auritus* (Rudolphi, 1819) Bona, 1994**

[For synonyms, see Scholz et al. (2004)]

*Poecilia reticulata* (Actinopterygii: Poeciliidae); freshwater; mesentery; metacestode; Pampulha Dam, Minas Gerais State; Brazil (Pinto and Melo 2011a).

***Parvitaenia macropeos* (Wedl, 1855) Baer & Bona, 1960**

[For synonyms, see Scholz et al. (2004)]

*Australoheros facetus* (Actinopterygii: Cichlidae); freshwater; intestine; metacestode; Pampulha Dam, Minas Gerais State; Brazil (Pinto and Melo 2011b).

***Valipora campylancristrota* (Wedl, 1855) Baer & Bona, 1960**

[For synonyms, see Scholz et al. (2004)]

*Geophagus brasiliensis* (Actinopterygii: Cichlidae); freshwater; gallbladder; metacestode; Paraná State (dams); Brazil (Bellay et al. 2012).

*Hoplosternum littorale* (Actinopterygii: Callichthyidae); freshwater; gallbladder; metacestode; Paraná River basin; Brazil (Takemoto et al. 2009).

*Prochilodus lineatus* (Actinopterygii: Prochilodontidae); freshwater; gallbladder; metacestode; Paraná River basin; Brazil (Lizama et al. 2005, 2006).

***Valipora* sp.**

*Crenicichla britskii* (Actinopterygii: Cichlidae); freshwater; gallbladder; metacestode; Paraná River basin; Brazil (Takemoto et al. 2009).

*Pimelodus maculatus* (Actinopterygii: Pimelodidae); freshwater; gallbladder; metacestode; Paraná River basin; Brazil (Takemoto et al. 2009).

*Prochilodus argenteus* (Actinopterygii: Prochilodontidae); freshwater; gallbladder; metacestode; São Francisco River basin; Brazil (Monteiro et al. 2009).

**Unidentified cyclophyllideans**

*Dormitator maculatus* (Actinopterygii: Eleotridae); amphidromous; liver, intestine, gonads; metacestode; TNA; Venezuela (Moreno et al. 2008).

*Percichthys trucha* (Actinopterygii: Percichthyidae); freshwater; body cavity; metacestode; Negro River basin (Patagonian region); Argentina (Ortubay et al. 1994).

*Satanoperca pappaterra* (Actinopterygii: Cichlidae); freshwater; site of infection and stage of development not given; Paraná River basin; Brazil (Kohn et al. 2011).

#### Order Diphyllidea van Beneden in Carus, 1863

##### Family Echinobothriidae Perrier, 1897

*Ahamulina catarina* Marques, Jensen & Caira, 2012*

*Scyliorhinus besnardi* (Elasmobranchii: Scyliorhinidae); marine; spiral valve; adult; WTSA; Brazil (Marques et al. 2012; Caira et al. 2013).

Notes: type host. Sequences of partial 18S (KC860176–KC860180), 28S (KC860128–KC860132) and *cox*1 (KC860220–KC860224) (Caira et al. 2013).

***Coronocestus notoguidoi* (Ivanov, 1997) Caira, Marques, Jensen, Kuchta & Ivanov, 2013**

[Syn. *Echinobothrium notoguidoi* Ivanov, 1997]

*Mustelus schmitti* (Elasmobranchii: Triakidae); marine; spiral valve; adult; WSTA; Argentina (Ivanov 1997; Alarcos et al. 2006; Tyler 2006).

Notes: type host. Tyler (2006) provided new morphological data based on examination of the type specimens deposited in MLP and USNPC.

***Halysioncum euzeti* (Campbell & Carvajal, 1980) Caira, Marques, Jensen, Kuchta & Ivanov, 2013**

[Syn. *Echinobothrium euzeti* Campbell & Carvajal, 1980]

*Sympterygia lima* (Elasmobranchii: Arhynchobatidae) marine; spiral valve; adult; WTSP; Chile (Campbell and Carvajal 1980; Tyler 2006).

Notes: type host; it was originally reported as *Psammobatis lima*. Tyler (2006) provided new morphological data based on examination of the type specimens deposited in USNPC.

***Halysioncum megacanthum* (Ivanov & Campbell, 1998) Caira, Marques, Jensen, Kuchta & Ivanov, 2013**

[Syn. *Echinobothrium megacanthum* Ivanov & Campbell, 1998]

*Myliobatis goodei* (Elasmobranchii: Myliobatidae); marine; spiral valve; adult; Magellanic; Argentina (Ivanov and Campbell 1998b; Tyler 2006).

Notes: type host. Tyler (2006) provided new morphological data based on examination of the type specimens deposited in MLP and USNPC.

***Halysioncum pigmentatum* (Ostrowski de Núñez, 1971) Caira, Marques, Jensen, Kuchta & Ivanov, 2013**

[Syn. *Echinobothrium pigmentatum* Ostrowski de Núñez, 1971]

*Zapteryx brevirostris* (Elasmobranchii: Rhinobatidae); marine; spiral valve; adult; WTSA; Argentina (Ostrowski de Núñez 1971; Tyler 2006).

Note: type host. Tyler (2006) provided new morphological data based on examination of the type specimens deposited in the Ostrowski de Núñez's collection.

**Unidentified diphyllideans**

Notothenia cf. angustata (Actinopterygii: Nototheniidae); marine; intestine; metacestode; WTSP; Chile (Muñoz et al. 2001).

*Sebastes capensis* (Actinopterygii: Sebastidae); marine; unspecified site of infection; metacestode; WTSP; Chile (González and Poulin 2005a, b; González et al. 2006).

Note: two morphotypes were distinguished by González et al. (2006).

#### Order Diphyllobothriidea Kuchta, Scholz, Brabec & Bray, 2008

##### Family Diphyllobothriidae Lühe, 1910

***Adenocephalus pacificus* Nybelin, 1931**

[For synonyms, see Hernández-Orts et al. (2015)]

*Anisotremus scapularis* (Actinopterygii: Haemulidae); marine; body cavity, viscera; metacestode; WTSP; Peru (Luque 1991).

*Ariopsis seemanni* (Actinopterygii: Ariidae); brackish, marine; peritoneum; metacestode; WTSP; Peru (Escalante and Miranda 1986).

Note: Escalante and Miranda (1986) performed experimental infection in dogs; they reported the host as *Galeichthys jordani*.

*Cilus gilberti* (Actinopterygii: Sciaenidae); marine; viscera; metacestode; WTSP; Peru (Chero et al. 2014b).

*Cynoscion analis* (Actinopterygii: Sciaenidae); marine; viscera; metacestode; WTSP; Peru (Escalante 1983).

*Galeichthys peruvianus* (Actinopterygii: Ariidae); marine; viscera, peritoneum; metacestode; WTSP; Peru (Kuchta et al. 2015).

Note: Kuchta et al. (2015) provided a list of records gathered from published data in an appendix.

*Genypterus maculatus* (Actinopterygii: Ophidiidae); marine; viscera; metacestode; WTSP; Peru (Escalante 1983).

*Menticirrhus ophicephalus* (Actinopterygii: Sciaenidae); marine; body cavity, viscera; metacestode; WTSP; Peru (Luque 1991).

*Merluccius gayi peruanus* (Actinopterygii: Merlucciidae); marine; peritoneum, viscera, stomach surface; metacestode; WTSP; Peru (Escalante 1983; Chero et al. 2014a).

Note: Chero et al. (2014a) also reported *Diphyllobothrium arctocephalinum* Johnston, 1937 from this host, but this species is a junior synonym of *Adenocephalus pacificus* (see Hernández-Orts et al. 2015).

*Paralabrax humeralis* (Actinopterygii: Serranidae); marine; site of infection not given; metacestode; WTSP; Peru (Iannacone and Alvariño 2009).

*Paralichthys adspersus* (Actinopterygii: Paralichthyidae); marine; stomach surface; metacestode; WTSP; Peru (Escalante and Miranda 1986).

*Paralonchurus peruanus* (Actinopterygii: Sciaenidae); marine; intestinal surface; metacestode; WTSP; Peru (Tantaleán 1975).

Note: the author reported the host as *Polyclemus peruanus*.

*Sarda chiliensis* (Actinopterygii: Scombridae); marine; body cavity; metacestode; WTSP; Peru (Baer 1969; Hernández-Orts et al. 2015; Kuchta et al. 2015).

Notes: the genus *Adenocephalus* Nybelin, 1931 was resurrected by Hernández-Orts et al. (2015), based on morphological and molecular data. Sequences of partial 28S (KR269760) and *cox*1 (KR269747) (Hernández-Orts et al. 2015).

*Sciaena callaensis* (Actinopterygii: Sciaenidae); marine; peritoneum, stomach surface; metacestode; WTSP; Peru (Tantaleán 1975).

*Sciaena deliciosa* (Actinopterygii: Sciaenidae); marine; peritoneum, stomach surface, viscera; metacestode; WTSP; Peru (Tantaleán 1975; Escalante and Miranda 1986; Llerena et al. 2001; Wicht et al. 2010; Chero et al. 2014c; Kuchta et al. 2015).

Note: Chero et al. (2014a) also reported *Diphyllobothrium arctocephalinum* (syn. of *Adenocephalus pacificus*) from this host.

*Scomberomorus sierra* (Actinopterygii: Scombridae); marine; body cavity; metacestode; WTSP; Peru (Baer 1969).

Note: host reported as *Serrasalmus maculatus*.

*Seriolella violacea* (Actinopterygii: Centrolophidae); marine; peritoneum; metacestode; WTSP; Peru (Escalante and Miranda 1986).

*Trachinotus paitensis* (Actinopterygii: Carangidae); marine; peritoneum; metacestode; WTSP; Peru (Escalante and Miranda 1986).

*Trachurus murphyi* (Actinopterygii: Carangidae); marine; body cavity; metacestode; WTSP; Peru (Kuchta et al. 2015).

***Diphyllobothrium dendriticum* (Nitzsch, 1824) Lühe, 1910**

[For synonyms, see Scholz et al. (2009)]

*Basilichthys australis* (Actinopterygii: Atherinopsidae); freshwater; mesentery, liver, muscles; metacestode; Riñihue and Panguipulli Lakes; Chile (Torres et al. 1998, 2004, 2012).

*Galaxias maculatus* (Actinopterygii: Galaxiidae); amphidromous; liver, body cavity, intestinal surface; metacestode; Nahuel Huapi (Patagonian region), Riñihue Lakes; Argentina, Chile (Ortubay 1994; Torres et al. 1998; Viozzi et al. 2009).

*Odontesthes mauleanum* (Actinopterygii: Atherinopsidae); freshwater; mesentery; metacestode; Panguipulli Lake; Chile (Torres et al. 2004, 2012).

*Oncorhynchus kisutch* (Actinopterygii: Salmonidae); anadromous; intestinal surface, mesentery, spleen; metacestode; Llanquihue Lake; Chile (Torres 1990).

*Oncorhynchus mykiss* (Actinopterygii: Salmonidae); anadromous; body cavity, mesentery, internal organs, muscles; metacestode; lakes of Valdivia River basin, Huechulaufquen, Rosario, Moreno and Nahuel Huapi Lakes (Patagonian region), lakes of Chiloé Island; Argentina, Chile (Szidat and Soria 1957; Szidat 1964; Torres et al. 1981, 1983, 1989a, b, 1991, 2004, 2012; Revenga 1993; Semenas and Kreiter 1995; Rozas et al. 2012).

Notes: tapeworms described as *Diphyllobothrium microcordiceps* by Szidat and Soria (1957). Host reported as *Salmo gairdneri* by some authors. Sequences of partial 18S +ITS1 + 5.8S + ITS2 (JN153006‒JN153018) and partial *cox*1 (JN152993‒JN153005) (Rozas et al. 2012).

*Percichthys trucha* (Actinopterygii: Percichthyidae); freshwater; body cavity, mesentery, internal organs, muscles; metacestode; Riñihue Lake; Chile (Torres et al. 1998).

*Percilia gillissi* (Actinopterygii: Perciliidae); freshwater; body cavity, mesentery, internal organs, muscles; metacestode; Riñihue Lake; Chile (Torres et al. 1989a).

*Salmo salar* (Actinopterygii: Salmonidae); anadromous; muscles; metacestode; Nahuel Huapi Lake (Patagonian region); Argentina (Szidat and Soria 1957; Szidat 1964).

*Salmo trutta* (Actinopterygii: Salmonidae); anadromous; body cavity; metacestode; Huechulaufquen and Rosario Lakes (Patagonian region), Valdivia River basin; Argentina, Chile (Torres et al. 1989a, b, 1991; Semenas and Kreiter 1995).

*Salvelinus fontinalis* (Actinopterygii: Salmonidae); anadromous; body cavity, muscles; metacestode; Huechulaufquen, Rosario, Moreno, Nahuel Huapi Lakes (Patagonian region); Argentina (Szidat and Soria 1957; Szidat 1964; Revenga 1993; Semenas and Kreiter 1995).

***Diphyllobothrium latum* (Linnaeus, 1758) Lühe, 1910**

[For synonyms see Scholz et al. (2009) and references therein]

*Basilichthys australis* (Actinopterygii: Atherinopsidae); freshwater; muscles; metacestode; Panguipulli Lake; Chile (Torres et al. 2004, 2012).

*Diplomystes camposensis* (Actinopterygii: Diplomystidae); freshwater; liver; metacestode; Riñihue Lake; Chile (Torres et al. 1989a).

Note: the authors reported the tapeworms as *Diphyllobothrium* sp., but Muñoz and Olmos (2008) and R. Kuchta (pers. comm.) suggested that they belong to *Diphyllobothrium latum*.

*Galaxias maculatus* (Actinopterygii: Galaxiidae); amphidromous; body cavity, muscles; metacestode; Panguipulli and Riñihue Lakes; Chile (Torres et al. 1989a, 1998, 2004).

*Galaxias platei* (Actinopterygii: Galaxiidae); amphidromous; body cavity; metacestode; Valdivia River Basin; Chile (Torres et al. 1989a).

*Odontesthes mauleanum* (Actinopterygii: Atherinopsidae); freshwater; liver, gonads, mesentery, muscles; metacestode; Panguipulli Lake; Chile (Torres et al. 2004, 2012).

*Oncorhynchus mykiss* (Actinopterygii: Salmonidae); anadromous; body cavity, internal organs, muscles; metacestode; lakes of Valdivia River basin, Huechulaufquen, Rosario, Moreno and Nahuel Huapi Lakes (Patagonian region); Argentina, Chile (Neghme et al. 1950; Neghme and Bertín 1951; Torres et al. 1989a, 1991, 2004, 2012; Revenga 1993; Semenas and Kreiter 1995).

Note: host reported as *Salmo gairdneri* by some authors.

*Percichthys trucha* (Actinopterygii: Percichthyidae); freshwater; body cavity, muscles; metacestode; lakes of Valdivia River basin; Chile (Torres et al. 1989a, 1998, 2004, 2012).

*Percichthys* sp. (Actinopterygii: Percichthyidae); freshwater; body cavity, pyloric caeca, liver, stomach, gonads, muscles; metacestode; Moreno and Nahuel Huapi Lakes (Patagonian region); Argentina (Revenga 1993).

*Salmo trutta* (Actinopterygii: Salmonidae); anadromous; body cavity; metacestode; lakes of Valdivia River basin, Huechulaufquen and Rosario Lakes (Patagonian region); Argentina, Chile (Neghme et al. 1950; Neghme and Bertín 1951; Torres et al. 1989a, b, 1991; Semenas and Kreiter 1995).

Note: host reported as *Salmo trutta trutta* and *Salmo trutta fario* by some authors.

*Salvelinus fontinalis* (Actinopterygii: Salmonidae); anadromous; liver; metacestode; Huechulaufquen, Moreno, Nahuel Huapi and Rosario Lakes (Patagonian region); Argentina (Revenga 1993; Semenas and Kreiter 1995).

***Diphyllobothrium* sp.**

[Reports from freshwater fishes most likely correspond to *Diphyllobothrium dendriticum* or *Diphyllobothrium latum* (R. Kuchta, pers. comm.). All reports of unidentified diphyllobothrideans are included in this section]

*Basilichthys australis* (Actinopterygii: Atherinopsidae); freshwater; liver; metacestode; Riñihue Lake; Chile (Torres et al. 1989a, 1998).

*Cilus gilberti* (Actinopterygii: Sciaenidae); marine; site of infection not given; metacestode; WTSP; Chile (Garcías et al. 2001).

*Cynoscion analis* (Actinopterygii: Sciaenidae); marine; body cavity, peritoneum, stomach surface; metacestode; WTSP; Peru (Escalante 1983).

*Engraulis ringens* (Actinopterygii: Engraulidae); marine; site of infection not given; metacestode; WTSP; Chile (George-Nascimento and Moscoso 2013).

*Galaxias maculatus* (Actinopterygii: Galaxiidae); amphidromous; body cavity, liver; metacestode; Moreno Lake (Patagonian region); Argentina (Ortubay 1994; Viozzi et al. 2009).

*Galaxias platei* (Actinopterygii: Galaxiidae); amphidromous; liver; metacestode; Riñihue Lake; Chile (Torres et al. 1989a).

*Genypterus brasiliensis* (Actinopterygii: Ophidiidae); marine; body cavity, intestinal serosa, intestine, muscles; metacestode; WTSA; Brazil (Knoff et al. 2008).

*Genypterus maculatus* (Actinopterygii: Ophidiidae); marine; body cavity, peritoneum, stomach surface; metacestode; WTSP; Chile, Peru (Escalante 1983, George-Nascimento and Huet 1984).

*Lophius gastrophysus* (Actinopterygii: Lophiidae); marine; body cavity, intestinal serosa; metacestode; WTSA; Brazil (Knoff et al. 2011).

*Merluccius australis* (Actinopterygii: Merlucciidae); marine; stomach wall; metacestode; Magellanic, WTSP; Chile, Falkland Islands (MacKenzie and Longshaw 1995).

*Merluccius gayi peruanus* (Actinopterygii: Merlucciidae); marine; body cavity, mesentery, peritoneum, stomach surface; metacestode: WTSP; Peru (Escalante 1983; Jara 1998).

*Merluccius hubbsi* (Actinopterygii: Merlucciidae); marine; stomach wall; metacestode; Magellanic; Argentina, Falkland Islands (MacKenzie and Longshaw 1995).

*Micromesistius australis australis* (Actinopterygii: Gadidae); marine; site of infection not given; metacestode; WTSP; Chile (Niklitschek et al. 2010; George-Nascimento et al. 2011; Chávez et al. 2012).

*Odontesthes regia* (Actinopterygii: Atherinopsidae); freshwater; liver, gonads; metacestode; Riñihue Lake; Chile (Torres et al. 1989a, 1998).

*Oncorhynchus kisutch* (Actinopterygii: Salmonidae); anadromous; stomach, spleen, liver, mesentery, gonads; metacestode; Aisén River basin; Chile (Torres et al. 1995, 2000).

*Oncorhynchus mykiss* (Actinopterygii: Salmonidae); anadromous; body cavity, internal organs, mesentery, muscles; metacestode; lakes of Valdivia River basin, Moreno and Nahuel Huapi Lakes (Patagonian region), Tarahuin Lake (Chiloe Island); Argentina, Chile (Wolffhügel 1949; Torres et al. 1977, 1980, 1982, 1989a, 2002, 2010; González et al. 1978, 1980; Revenga and Semenas 1991; Revenga et al. 1995; Torres and Puga 2011).

Notes: host reported as *Salmo gairdneri* or *Salmo gairdneri irideus* by some authors. After experimental infections of small rodents with metacestodes, González et al. (1980) recovered tapeworms morphologically similar with *Diphyllobothrium dentriticum*.

*Paralichthys isosceles* (Actinopterygii: Paralichthyidae); marine; body cavity, mesentery, liver, ovary, stomach; metacestode; WTSA; Brazil (Felizardo et al. 2010).

Note: Felizardo et al. (2010) distinguished two morphotypes.

*Paralonchurus peruanus* (Actinopterygii: Sciaenidae); marine; body cavity; metacestode; WTSP; Peru (Tantaleán 1975).

Note: host reported as *Polyclemus peruanus*.

*Salmo trutta* (Actinopterygii: Salmonidae); anadromous; body cavity, peritoneum, liver, mesentery, muscles; metacestode; Rupanco and Calafquén Lakes, Moreno and Nahuel Huapi Lakes (Patagonian region); Argentina, Chile (González et al. 1978, 1980; Torres et al. 1980; Revenga and Semenas 1991).

Note: host reported as *Salmo trutta fario* or *Salmo trutta trutta* by some authors.

*Salvelinus fontinalis* (Actinopterygii: Salmonidae); anadromous; body cavity, muscles; metacestode; Moreno and Nahuel Huapi Lakes (Patagonian region); Argentina (Revenga and Semenas 1991; Revenga et al. 1995).

*Sciaena callaensis* (Actinopterygii: Sciaenidae); marine; peritoneum, stomach surface, body cavity; metacestode; WTSP; Peru (Escalante 1983).

*Sciaena deliciosa* (Actinopterygii: Sciaenidae); marine; body cavity, intestinal surface; metacestode; WTSP; Peru (Tantaleán 1975; Llerena et al. 2001).

*Scomber japonicus* (Actinopterygii: Scombridae); marine; site of infection not given; metacestode; WTSP; Peru (Oliva et al. 2008b).

*Sebastes capensis* (Actinopterygii: Sebastidae); marine; site of infection not given; metacestode; Magellanic; Chile (González and Poulin 2005a, b; González et al. 2006).

*Trachurus murphyi* (Actinopterygii: Carangidae); marine; liver; metacestode; WTSP; Chile, Peru (Pérez et al. 1999; George-Nascimento and Oliva 2015).

**Unidentified ‘Pseudophyllidea’ (larval stages)**

[Larval stages found in the body cavity and mesentery are most likely species of *Diphyllobothrium* (R. Kuchta, pers. comm.)]

*Aphos porosus* (Actinopterygii: Batrachoididae); marine; body cavity; metacestode; WTSP; Chile (Cortés and Muñoz 2009).

*Balistes capriscus* (Actinopterygii: Balistidae); marine; site of infection not given; metacestode; WTSA; Brazil (Luque and Poulin 2004; Alves et al. 2005).

*Dissostichus eleginoides* (Actinopterygii: Nototheniidae); marine; anterior

intestine; metacestode; Magellanic; Chile, Falkland Islands (Brickle et al. 2006; Oliva et al. 2008a; Brown et al. 2013).

*Engraulis anchoita* (Actinopterygii: Engraulidae); marine; mesentery; metacestode; WTSA; Argentina (Timi 2003; Timi and Poulin 2003; Timi et al. 2010b).

*Gobiesox marmoratus* (Actinopterygii: Gobiesocidae); marine; site of infection not given; metacestode; WTSP; Chile (Muñoz 2014).

*Helcogrammoides chilensis* (Actinopterygii: Tripterygiidae); marine; site of infection not given; metacestode; WTSP; Chile (Muñoz and Delorme 2011).

*Hypsoblennius sordidus* (Actinopterygii: Blenniidae); marine; intestine; metacestode; Magellanic; Chile (Sepúlveda et al. 2004).

*Macruronus magellanicus* (Actinopterygii: Merlucciidae); marine; body cavity; metacestode; Magellanic; Argentina, Chile (Oliva 2001; MacKenzie et al. 2013).

*Merluccius gayi gayi* (Actinopterygii: Merlucciidae); marine; stomach wall; metacestode; WTSP; Chile (Oliva and Ballón 2002).

*Merluccius hubbsi* (Actinopterygii: Merlucciidae); marine; mesentery; metacestode; Magellanic; Argentina (Sardella and Timi 2004).

Note: Sardella and Timi (2004) distinguished two morphotypes.

*Micromesistius australis australis* (Actinopterygii: Gadidae); marine; unspecified site of infection; metacestode; Magellanic; Chile (Niklitschek et al. 2010; George-Nascimento et al. 2011).

*Odontesthes regia* (Actinopterygii: Atherinopsidae); marine; intestine; metacestode; Magellanic; Chile (Sepúlveda et al. 2004).

*Percophis brasiliensis* (Actinopterygii: Percophidae); marine; mesentery; metacestode; WTSA; Argentina, Uruguay (Braicovich and Timi 2008).

*Prolatilus jugularis* (Actinopterygii: Pinguipedidae); marine; intestine; metacestode; Magellanic; Chile (Sepúlveda et al. 2004).

*Scartichthys viridis* (Actinopterygii: Bleniidae); marine; site of infection not given; metacestode; WTSA; Chile (Muñoz and Delorme 2011; Muñoz and Randhawa 2011).

*Sicyases sanguineus* (Actinopterygii: Gobiesocidae); marine; site of infection not given; metacestode; WTSA; Chile (Muñoz and Delorme 2011; Muñoz and Randhawa 2011).

*Trachurus lathami* (Actinopterygii: Carangidae); marine; mesentery; metacestode; WTSA; Argentina, Brazil (Braicovich et al. 2012).

*Trachurus murphyi* (Actinopterygii: Carangidae); marine; site of infection not given; metacestode; WTSP; Chile (George-Nascimento and Arancibia 1992; George-Nascimento 2000; George-Nascimento and Oliva 2015).

#### Order Gyrocotylidea Poche, 1926

##### Family Gyrocotylidae Benham, 1901

***Gyrocotyle maxima* MacDonagh, 1927**

[Syns. *Gyrocotyle meandrica* Mendívil-Herrera, 1946; *Gyrocotyle urna* sensu Manter, 1951; *Amphiptyches urna* Spencer, 1889]

*Callorhinchus callorynchus* (Holocephali: Callorhinchidae); marine; spiral valve; adult; WTSA, WTSP; Brazil, Chile, Peru, Uruguay (Mendívil-Herrera 1946; Rego et al. 1974; Fernández et al. 1986; Tantaleán 1991).

Note: tapeworms reported as *Gyrocotyle meandrica* by Mendívil-Herrera (1946) and Rego et al. (1974).

*Mustelus schmitti* (Elasmobranchii: Triakidae); marine; spiral valve; adult; WTSA; Argentina (MacDonagh 1927).

Notes: type host; it was reported as *Mustelus asterias*, but most likely MacDonagh (1927) misidentified the elephant fish *Callorhinchus callorynchus* (see Caira et al. 2012), common definitive host of *Gyrocotyle maxima*.

*Gyrocotyle rugosa* Diesing, 1850*

[Syn. *Gyrocotyle plana* Linton, 1924]

*Callorhinchus callorynchus* (Holocephali: Callorhinchidae); marine; spiral valve; adult; WTSA, WTSP; Argentina, Chile (MacDonagh 1927; Fernández et al. 1986).

Note: type host.

**Unidentified gyrocotylidean**

*Callorhinchus callorynchus* (Holocephali: Callorhinchidae); marine; spiral valve; adult; WTSA; Uruguay (Mendívil-Herrera 1946).

#### Order Lecanicephalidea Wardle & McLeod, 1952

##### Family Aberrapecidae Jensen, Caira, Cielocha, Littlewood & Waeschenbach, 2016

***Aberrapex arrhynchum* (Brooks, Mayes & Thorson, 1981) Jensen, 2001**

[Syn. *Discobothrium arrhynchum* Brooks, Mayes & Thorson, 1981]

*Myliobatis goodei* (Elasmobranchii: Myliobatidae); marine; spiral valve; adult; WTSA (estuary of the La Plata River); Uruguay (Brooks et al. 1981a; Jensen 2001).

Notes: type host.

##### Family Cephalobothriidae Pintner, 1928

***Tylocephalum brooksi* Ivanov & Campbell, 2000**

*Rhinoptera bonasus* (Elasmobranchii: Myliobatidae); marine; spiral valve; adult; TNA; Venezuela (Ivanov and Campbell 2000).

Note: type host.

***Tylocephalum* sp.**

*Rhinoptera bonasus* (Elasmobranchii: Myliobatidae); marine; spiral valve; adult; TNA; Venezuela (Mayes and Brooks 1981).

##### Family Lecanicephalidae Braun, 1900

*Lecanicephalum peltatum* Linton, 1890*

*Dasyatis americana* (Elasmobranchii: Dasyatidae); marine; spiral valve; adult; TEP; Colombia (Brooks and Mayes 1980).

##### Family Paraberrapecidae Jensen, Caira, Cielocha, Littlewood & Waeschenbach, 2016

***Paraberrapex atlanticus* Mutti & Ivanov, 2016**

*Squatina guggenheim* (Elasmobranchii: Squatinidae); marine; spiral valve; adult; Magellanic, WTSA; Argentina (Mutti and Ivanov 2016).

Note: type host.

##### Family Polypocephalidae Meggitt, 1924

***Polypocephalus medusia* (Linton, 1890) Southwell, 1925**

[Syn. *Parataenia medusia* Linton, 1890]

*Dasyatis americana* (Elasmobranchii: Dasyatidae) marine; spiral valve; adult; TEP; Colombia (Brooks and Mayes 1980).

#### Order Onchoproteocephalidea Caira, Jensen, Waeschenbach, Olson & Littlewood, 2014

**(Syns. Proteocephalidea Mola, 1928; Tetraphyllidea Carus, 1863 *pro parte*)**

##### Family Onchobothriidae Braun, 1900

***Acanthobothrium amazonense* Mayes, Brooks & Thorson, 1978**

*Potamotrygon constellata* (Elasmobranchii: Potamotrygonidae); freshwater; spiral valve; adult; Amazon River basin; Brazil (Mayes et al. 1978; Brooks et al. 1981b).

Notes: type host; it was reported as *Potamotrygon circularis*. Brooks et al. (1981b) studied the type specimens deposited in USNM.

***Acanthobothrium americanum* Campbell, 1969**

*Dasyatis americana* (Elasmobranchii: Dasyatidae); marine; spiral valve; adult; TNA; Venezuela (Mayes and Brooks 1981).

Note: type host.

***Acanthobothrium annapinkiense* Carvajal & Goldstein, 1971**

*Zearaja chilensis* (Elasmobranchii: Rajidae); marine; spiral valve; adult; Magellanic; Chile (Carvajal and Goldstein 1971).

Note: type host; it was reported as *Raja chilensis*.

***Acanthobothrium atahualpai* Marques, Brooks & Barriga, 1997**

*Gymnura afuerae* (Elasmobranchii: Gymnuridae); marine; spiral valve; adult; TEP; Ecuador (Marques et al. 1997).

Note: type host.

***Acanthobothrium batailloni* Euzet, 1955**

*Myliobatis chilensis* (Elasmobranchii: Myliobatidae); marine; spiral valve; adult; WTSP; Chile, Peru (Carvajal and Jeges 1980; Oliva 1982; Escalante 1986).

***Acanthobothrium brevissime* Linton, 1908**

*Myliobatis peruvianus* (Elasmobranchii: Myliobatidae); marine; spiral valve; adult; WTSP; Peru (Tantaleán 1991).

***Acanthobothrium campbelli* Marques, Brooks & Monks, 1995**

*Dasyatis longa* (Elasmobranchii: Dasyatidae); marine; spiral valve; adult; TEP; Ecuador (Marques et al. 1997).

***Acanthobothrium cartagenense* Brooks & Mayes, 1980**

*Urobatis jamaicensis* (Elasmobranchii: Urotrygonidae); marine; spiral valve; adult; TNA; Colombia (Brooks and Mayes 1980).

Note: type host; it was reported as *Urolophus jamaicensis*.

***Acanthobothrium chilense* Rego, Vicente & Herrera, 1968**

*Sarda chiliensis* (Actinopterygii: Scombridae); marine; intestine; adult; WTSP; Peru (Rego et al. 1968).

Notes: type host. Elasmobranchs are the typical definitive host for *Acanthobothrium* species (Campbell and Beveridge 2002); therefore, the record of adult specimens from a bony fish host needs verification.

***Acanthobothrium colombianum* Brooks & Mayes, 1980**

*Aetobatus narinari* (Elasmobranchii: Myliobatidae); marine; spiral valve; adult; TNA; Colombia (Brooks and Mayes 1980).

Note: type host.

***Acanthobothrium coquimbense* Carvajal & Jeges, 1980**

*Myliobatis chilensis* (Elasmobranchii: Myliobatidae); marine; spiral valve; adult; WTSP; Chile (Carvajal and Jeges 1980).

Note: type host.

***Acanthobothrium costarricense* Marques, Brooks & Monks, 1995**

*Dasyatis longa* (Elasmobranchii: Dasyatidae); marine; spiral valve; adult; TEP; Ecuador (Marques et al. 1997).

Note: type host.

***Acanthobothrium dasybati* Yamaguti, 1934**

Unidentified ray host (Elasmobranchii); marine; spiral valve; adult (only one immature specimen; WTSA; Brazil (Rego et al. 1974).

Note: this species was described from *Dasyatis akajei* in the Western Pacific (Japanese Sea) and its report from the Brazilian coast needs verification.

***Acanthobothrium electricolum* Brooks & Mayes, 1978**

*Narcine brasiliensis* (Elasmobranchii: Narcinidae); marine; spiral valve; adult; TNA; Colombia, Venezuela (Brooks and Mayes 1978; Mayes and Brooks 1981).

Note: type host.

***Acanthobothrium fogeli* Goldstein, 1964**

*Gymnura micrura* (Elasmobranchii: Gymnuridae); marine; spiral valve; adult; TNA; Venezuela (Mayes and Brooks 1981).

Note: type host.

***Acanthobothrium gonzalesmugaburoi* Severino & Sarmiento, 1979**

*Myliobatis peruvianus* (Elasmobranchii: Myliobatidae); marine; spiral valve; adult; WTSP; Peru (Severino and Sarmiento 1979).

Note: type host.

***Acanthobothrium himanturi* Brooks, 1977**

*Himantura schmardae* (Elasmobranchii: Dasyatidae); marine; spiral valve; adult; TNA; Colombia (Brooks 1977).

Note: type host.

***Acanthobothrium holorhini* Alexander, 1953**

*Myliobatis chilensis* (Elasmobranchii: Myliobatidae); marine; spiral valve; adult; WTSP; Peru (Rodriguez and Tantaleán 1980)

***Acanthobothrium lintoni* Goldstein, Henson & Schlicht, 1968**

*Narcine brasiliensis* (Elasmobranchii: Narcinidae); marine; spiral valve; adult; TNA; Colombia (Brooks and Mayes 1978).

Note: type host.

***Acanthobothrium lusarmientoi* Severino & Verano, 1980**

*Sympterygia brevicaudata* (Elasmobranchii: Arhynchobatidae); marine; spiral valve; adult; WTSP; Peru (Severino and Verano 1980).

Note: type host; it was reported as *Psammobatis caudispina*.

***Acanthobothrium marplatense* Ivanov & Campbell, 1998**

*Atlantoraja castelnaui* (Elasmobranchii: Arhynchobatidae); marine; spiral valve; adult; WTSA; Argentina (Ivanov and Campbell 1998a).

Note: type host; it was reported as *Rioraja castelnaui*.

***Acanthobothrium minusculum* Marques, Brooks & Barriga, 1997**

*Urobatis tumbesensis* (Elasmobranchii: Urotrygonidae); marine; spiral valve; adult; TEP; Ecuador (Marques et al. 1997).

Note: type host; it was reported as *Urolophus tumbesensis*.

***Acanthobothrium monksi* Marques, Brooks & Barriga, 1997**

*Aetobatus narinari* (Elasmobranchii: Myliobatidae); marine; spiral valve; adult; TEP; Ecuador (Marques et al. 1997).

Note: type host.

***Acanthobothrium obuncum* Marques, Brooks & Barriga, 1997**

*Dasyatis longa* (Elasmobranchii: Dasyatidae); marine; spiral valve; adult; TEP; Ecuador (Marques et al. 1997).

Note: type host.

***Acanthobothrium olseni* Dailey & Mudry, 1968**

*Rhinobatos planiceps* (Elasmobranchii: Rhinobatidae); marine; spiral valve; adult; WTSP; Chile, Peru (Dailey and Carvajal 1976; Iannacone et al. 2011).

***Acanthobothrium peruviense* Reyda, 2008**

Potamotrygon cf. falkneri (Elasmobranchii: Potamotrygonidae); freshwater; spiral valve; adult; Amazon River basin; Peru (Reyda 2008).

Note: host reported as Potamotrygon cf. castexi and the tapeworms as Acanthobothrium cf. peruviense.

*Potamotrygon motoro* (Elasmobranchii: Potamotrygonidae); freshwater; spiral

valve; adult; Amazon River basin; Peru (Reyda 2008).

Note: type host.

***Acanthobothrium psammobati* Carvajal & Goldstein, 1969**

*Psammobatis scobina* (Elasmobranchii: Arhynchobatidae); marine; spiral valve; adult; WTSP; Chile, Peru (Carvajal and Goldstein 1969; Carvajal et al. 1985; Tantaleán 1991).

Note: type host.

*Sympterygia brevicaudata* (Elasmobranchii: Arhynchobatidae); marine; spiral

valve; adult; WTSP; Chile (Carvajal and Ruíz 1987).

***Acanthobothrium quinonese* Mayes, Brooks & Thorson, 1978**

*Potamotrygon magdalenae* (Elasmobranchii: Potamotrygonidae); freshwater; spiral valve; adult; Magdalena River basin; Colombia (Mayes et al. 1978; Brooks et al. 1981b).

Notes: type host. Brooks et al. (1981b) studied the type specimens deposited in USNM.

*Potamotrygon yepezi* (Elasmobranchii: Potamotrygonidae); freshwater; spiral valve; adult; Maracaibo basin; Venezuela (Brooks et al. 1981b).

***Acanthobothrium ramiroi* Ivanov, 2005**

*Potamotrygon motoro* (Elasmobranchii: Potamotrygonidae); freshwater; spiral valve; adult; Amazon and Paraná River basins; Argentina, Peru (Ivanov 2005; Reyda 2008).

Notes: type host. Reyda (2008) reported the tapeworms as Acanthobothrium cf. ramiroi.

***Acanthobothrium regoi* Brooks, Mayes & Thorson, 1981**

*Potamotrygon falkneri* (Elasmobranchii: Potamotrygonidae); freshwater; spiral valve; adult; Paraná River basin; Brazil (Lacerda et al. 2008, 2009).

*Potamotrygon motoro* (Elasmobranchii: Potamotrygonidae); freshwater; spiral valve; adult; Paraná River basin; Brazil (Brooks and Amato 1992).

*Potamotrygon orbignyi* (Elasmobranchii: Potamotrygonidae); freshwater; spiral valve; adult; Orinoco River basin; Venezuela (Brooks et al. 1981b).

Note: type host; it was reported as *Paratrygon hystrix*.

***Acanthobothrium robustum* Alexander, 1953**

*Rhinobatos planiceps* (Elasmobranchii: Rhinobatidae); marine; spiral valve; adult; WTSP; Peru (Escalante 1986).

***Acanthobothrium tasajerasi* Brooks, 1977**

*Dasyatis guttata* (Elasmobranchii: Dasyatidae); marine; spiral valve; adult; Maracaibo basin; Venezuela (Mayes and Brooks 1981).

*Himantura schmardae* (Elasmobranchii: Dasyatidae); marine; spiral valve; adult; TNA; Colombia (Brooks 1977).

Note: type host.

***Acanthobothrium terezae* Rego & Dias, 1976**

*Paratrygon aiereba* (Elasmobranchii: Potamotrygonidae); freshwater; spiral valve; adult; Amazon River basin; Brazil (Reyda and Marques 2011).

Notes: Reyda and Marques (2011) reported the tapeworms as Acanthobothrium cf. terezae. Sequence of partial *cox*1 (JF803661) (Reyda and Marques 2011).

*Potamotrygon motoro* (Elasmobranchii: Potamotrygonidae); freshwater; spiral valve; adult; Paraná River basin; Brazil (Rego and Dias 1976).

Note: type host; it was reported as *Paratrygon motoro* and *Elipesurus* sp.

***Acanthobothrium tortum* (Linton, 1916) Baer & Euzet, 1962**

*Aetobatus narinari* (Elasmobranchii: Myliobatidae); marine; spiral valve; adult; TNA; Venezuela (Mayes and Brooks 1981).

Note: type host.

***Acanthobothrium urotrygoni* Brooks & Mayes, 1980**

*Dasyatis guttata* (Elasmobranchii: Dasyatidae); marine; spiral valve; adult; TNA; Venezuela (Mayes and Brooks 1981).

*Urotrygon venezuelae* (Elasmobranchii: Urotrygonidae); marine; spiral valve; adult; TNA; Colombia (Brooks and Mayes 1980).

Note: type host.

***Acanthobothrium zapterycum* Ostrowski de Núñez, 1971**

*Zapteryx brevirostris* (Elasmobranchii: Rhinobatidae); marine; spiral valve; adult; WTSA; Argentina (Ostrowski de Núñez 1971).

Note: type host.

***Acanthobothrium* sp.**

*Myliobatis chilensis* (Elasmobranchii: Myliobatidae); marine; spiral valve; adult; WTSP; Peru (Tresierra et al. 1986).

*Myliobatis goodei* (Elasmobranchii: Myliobatidae); marine; spiral valve; adult; WTSA (La Plata River estuary); Uruguay (Brooks et al. 1981a).

Note: the authors distinguished two morphotypes.

*Sympterygia brevicaudata* (Elasmobranchii: Arhynchobatidae); marine; spiral valve; adult; WTSP; Chile (Carvajal and Ruíz 1987).

*Zapteryx brevirostris* (Elasmobranchii: Rhinobatidae); marine; spiral valve; adult; WTSA; Argentina (Ostrowski de Núñez 1971).

*Acanthobothroides thorsoni* Brooks, 1977*

*Dasyatis dipterura* (Elasmobranchii: Dasyatidae); marine; spiral valve; adult; WTSP; Peru (Tantaleán and Rodríguez 1987).

Note: host reported as *Dasyatis brevis*.

*Dasyatis guttata* (Elasmobranchii: Dasyatidae); marine; spiral valve; adult; TNA; Venezuela (Mayes and Brooks 1981).

*Himantura schmardae* (Elasmobranchii: Dasyatidae); marine; spiral valve; adult; TNA; Colombia (Brooks 1977).

Note: type host.

***Platybothrium auriculatum* Yamaguti, 1952**

[Syns. *Platybothrium baeri* Euzet, 1952; *Cylindrophorus posteroporus* Riser, 1955]

*Prionace glauca* (Elasmobranchii: Carcharhinidae); marine; spiral valve; adult; TSA, WTSP; Brazil, Chile, Peru (Carvajal 1974; Rego and Mayer 1976; Escalante 1986).

Note: Healy (2003) revised the genus *Platybothrium* Linton, 1890.

***Platybothrium* sp.**

*Sphyrna zygaena* (Elasmobranchii: Sphyrnidae); marine; spiral valve; adult; WTSP; Peru (López de McDonald and Tantaleán 1985).

***Potamotrygonocestus amazonensis* Mayes, Brooks & Thorson, 1981**

*Potamotrygon constellata* (Elasmobranchii: Potamotrygonidae); freshwater; spiral valve; adult; Amazon River basin; Brazil (Brooks et al. 1981b; Mayes et al. 1981; Marques et al. 2003).

Notes: type host; it was reported as *Potamotrygon circularis*. Marques et al. (2003) reported this host as *Potamotrygon orbignyi*, but according to Brooks and Amato (1992), all potamotrygonids collected near Leticia, Colombia should be considered as *Potamotrygon constellata*.

*Potamotrygon falkneri* (Elasmobranchii: Potamotrygonidae); freshwater; spiral valve; adult; Paraná River basin; Brazil (Marques et al. 2003).

*Potamotrygon motoro* (Elasmobranchii: Potamotrygonidae); freshwater; spiral valve; adult; Amazon and Paraná River basins; Brazil (Marques et al. 2003).

Note: Marques et al. (2003) redescribed this species.

*Potamotrygon orbignyi* (Elasmobranchii: Potamotrygonidae); freshwater; spiral valve; adult; Amazon and Orinoco River basins; Brazil, Venezuela (Brooks et al. 1981b; Marques et al. 2003).

Note: host reported as *Potamotrygon reticulatus* by Brooks et al. (1981b).

*Potamotrygon scobina* (Elasmobranchii: Potamotrygonidae); freshwater; spiral valve; adult; Amazon River basin; Brazil (Marques et al. 2003).

*Potamotrygon yepezi* (Elasmobranchii: Potamotrygonidae); freshwater; spiral valve; adult; Maracaibo basin; Venezuela (Brooks et al. 1981b).

*Potamotrygon* sp. (Elasmobranchii: Potamotrygonidae); freshwater; spiral valve; adult; Amazon River basin; Brazil (Marques et al. 2003).

***Potamotrygonocestus chaoi* Marques, Brooks & Araujo, 2003**

*Plesiotrygon iwamae* (Elasmobranchii: Potamotrygonidae); freshwater; spiral valve; adult; Amazon River basin; Brazil (Marques et al. 2003; Luchetti et al. 2008).

Notes: type host. Luchetti et al. (2008) redescribed this species.

***Potamotrygonocestus fitzgeraldae* Marques, Brooks & Araujo, 2003**

*Paratrygon aiereba* (Elasmobranchii: Potamotrygonidae); freshwater; spiral valve; adult; Amazon River basin; Brazil, Peru (Marques et al. 2003; Reyda 2008).

Notes: type host. Reyda (2008) reported this species as Potamotrygonocestus cf. fitzgeraldae.

Potamotrygon cf. falkneri (Elasmobranchii: Potamotrygonidae); freshwater; spiral valve; adult; Amazon River basin; Peru (Caira et al. 2014).

Notes: host reported as *Potamotrygon castexi* and the tapeworms as Potamotrygonocestus cf. fitzgeraldae. Sequences of partial 18S (KF685832) and 28S (KF685773) (Caira et al. 2014).

*Potamotrygon leopoldi* (Elasmobranchii: Potamotrygonidae); freshwater; spiral valve; adult; Amazon River basin; Brazil (Marques et al. 2003).

*Potamotrygon motoro* (Elasmobranchii: Potamotrygonidae); freshwater; spiral valve; adult; Paraná River basin; Argentina (Marques et al. 2003).

*Potamotrygon orbignyi* (Elasmobranchii: Potamotrygonidae); freshwater; spiral valve; adult; Paraná River basin; Brazil (Marques et al. 2003).

*Potamotrygonocestus magdalenensis* Brooks & Thorson, 1976*

*Potamotrygon magdalenae* (Elasmobranchii: Potamotrygonidae); freshwater; spiral valve; adult; Magdalena River basin; Colombia (Brooks and Thorson 1976; Brooks et al. 1981b; Caira and Orringer 1995; Marques et al. 2003).

Note: type host.

***Potamotrygonocestus marajoara* Luchetti, Marques & Charvet-Almeida, 2008**

*Plesiotrygon iwamae* (Elasmobranchii: Potamotrygonidae); freshwater; spiral valve; adult; Amazon River basin (estuary); Brazil (Luchetti et al. 2008).

Note: type host.

***Potamotrygonocestus maurae* Marques, Brooks & Araujo, 2003**

*Potamotrygon orbignyi* (Elasmobranchii: Potamotrygonidae); freshwater; spiral valve; adult; Amazon River basin; Brazil (Marques et al. 2003).

Note: type host.

***Potamotrygonocestus travassosi* Rego, 1979**

[Syn. *Potamotrygonocestus orinocoensis* Brooks, Mayes & Thorson, 1981]

*Paratrygon aiereba* (Elasmobranchii: Potamotrygonidae); freshwater; spiral valve; adult; Amazon River basin; Brazil (Marques et al. 2003).

*Potamotrygon constellata* (Elasmobranchii: Potamotrygonidae); freshwater; spiral valve; adult; Amazon River basin; Brazil (Marques et al. 2003).

*Potamotrygon falkneri* (Elasmobranchii: Potamotrygonidae); freshwater; spiral valve; adult; Paraná River basin; Brazil (Lacerda et al. 2008, 2009).

*Potamotrygon motoro* (Elasmobranchii: Potamotrygonidae); freshwater; spiral valve; adult; Paraná River basin; Brazil (Brooks and Amato 1992).

Note: tapeworms reported as *Potamotrygonocestus orinocoensis*.

*Potamotrygon orbignyi* (Elasmobranchii: Potamotrygonidae); freshwater; spiral valve; adult; Amazon and Orinoco River basins; Brazil, Venezuela (Rego 1979; Brooks et al. 1981b; Brooks and Amato 1992; Marques et al. 2003).

Notes: type host; it was reported as *Potamotrygon reticulatus* and *Paratrygon hystrix*. The taxon was considered a *species inquirenda* by Brooks et al. (1981b) and Brooks and Amato (1992), but its validity was confirmed by Marques et al. (2003) who also considered *Potamotrygonocestus orinocoensis*, both described from this host, a junior synonym of *Potamotrygonocestus travassosi*.

***Potamotrygonocestus* sp.**

*Paratrygon aiereba* (Elasmobranchii: Potamotrygonidae); freshwater; spiral valve; adult; Amazon River basin; Peru (Reyda and Olson 2003).

Note: Reyda and Olson (2003) reported hyperparasitism caused by metacestodes of proteocephalids.

Potamotrygon cf. falkneri (Elasmobranchii: Potamotrygonidae); freshwater; spiral valve; adult; Amazon River basin; Peru (Reyda 2008).

Note: host reported as Potamotrygon cf. castexi.

*Potamotrygon henlei* (Elasmobranchii: Potamotrygonidae); freshwater; spiral valve; adult; Tocantins-Araguaia River basin; Brazil (Marques et al. 2003).

Note: these cestodes may represent an undescribed species of the genus (Marques et al. 2003).

*Potamotrygon motoro* (Elasmobranchii: Potamotrygonidae); freshwater; spiral valve; adult; Amazon River basin; Peru (Reyda 2008).

*Potamotrygon schroederi* (Elasmobranchii: Potamotrygonidae); freshwater; spiral valve; adult; Amazon River basin; Brazil (Marques et al. 2003).

Note: these cestodes may represent an undescribed species of the genus (Marques et al. 2003).

##### Family Prosobothriidae Baer & Euzet, 1955

*Prosobothrium armigerum* Cohn, 1902*

*Prionace glauca* (Elasmobranchii: Carcharhinidae); marine; spiral valve; adult; WTSP; Peru (Rivera and Sarmiento 1990).

##### Family Proteocephalidae La Rue, 1911

[Even though recent molecular data suggest that most of the traditionally recognized subfamilies are artificial, i.e. non-monophyletic, we are following Woodland’s subfamilial classification for practical reasons]

###### Subfamily Corallobothriinae Freze, 1965

***Corallotaenia* sp.**

*Ageneiosus pardalis* (Actinopterygii: Auchenipteridae); freshwater; intestine; adult (immature specimens); Magdalena River basin; Colombia (Brooks and Deardorff 1980).

Notes: host reported as *Ageneiosus caucanus*. This is the first record of the genus in South America (Brooks and Deardorff 1980), but since only immature specimens were found, this needs verification.

*Megathylacus jandia* Woodland, 1934*

[Syn. *Megathylacus brooksi* Rego & Pavanelli, 1985]

*Zungaro jahu* (Actinopterygii: Pimelodidae); freshwater; intestine; adult; Paraná River basin; Brazil (Rego and Gibson 1989; Rego and Pavanelli 1985; Eiras et al. 1986; Kohn and Fernandes 1987; Pavanelli and Machado 1991; Takemoto and Pavanelli 1994; Kohn et al. 2011; de Chambrier et al. 2014).

Notes: host reported as *Paulicea luetkeni* or *Zungaro zungaro* (for details on the host taxonomic status, see Boni et al. 2011). Rego and Gibson (1989) and Rego and Pavanelli (1985) reported hyperparasitism caused by metacestodes of proteocephalids.

*Zungaro zungaro* (Actinopterygii: Pimelodidae); freshwater; intestine; adult; Amazon River basin; Brazil, Peru (Woodland 1934c; Zehnder and Mariaux 1999; Hypša et al. 2005; de Chambrier et al. 2014, 2015a).

Notes: type host; it was originally reported as *Rhamdia* sp., but it is most likely *Zungaro zungaro* as discussed by de Chambrier et al. (2014), who reassessed the taxonomic status of this cestode species. Sequences of partial 18S (AY551111), complete ITS2 (AY551147), partial 28S (AJ388596) and 16S (AJ389515) (Zehnder and Mariaux 1999; Hypša et al. 2005).

***Megathylacus* sp.**

*Pseudoplatystoma corruscans* (Actinopterygii: Pimelodidae); freshwater; intestine; adult; Paraná River basin; Brazil (Rego 1990).

*Pseudoplatystoma fasciatum* (Actinopterygii: Pimelodidae); freshwater; intestine; adult (also immature specimens); Amazon River basin; Peru (de Chambrier et al. 2014, 2015a).

Note: host reported as *Pseudoplatystoma punctifer* by de Chambrier et al. (2014), however, it falls within the range of genetic variability of *Pseudoplatystoma fasciatum* (*sensu lato*), according to Carvalho-Costa et al. (2011).

***Megathylacus travassosi* Pavanelli & Rego, 1992**

*Pseudoplatystoma corruscans* (Actinopterygii: Pimelodidae); freshwater; intestine; adult; Paraná River basin; Brazil (Pavanelli and Machado 1991; Pavanelli and Rego 1992; Machado et al. 1994, 1995, 1996; Rego 2002; de Chambrier et al. 2014; Ribeiro et al. 2014).

Notes: type host. Pavanelli and Machado (1991) referred to *Megathylacus travassosi* prior its formal publication one year later, which could render the name a *nomen nudum* but which was neglected in subsequent works.

*Pseudoplatystoma fasciatum* (Actinopterygii: Pimelodidae); freshwater; intestine; adult; Paraná River basin; Brazil (Campos et al. 2008, 2009a, b).

*Sciadocephalus megalodiscus* Diesing, 1850*

*Cichla kelberi* (Actinopterygii: Cichlidae); freshwater; intestine; adult; Paraná

River basin; Brazil (Yamada and Takemoto 2013).

Note: this host should be synonymized with *Cichla ocellaris* based on molecular data (Willis et al. 2012).

*Cichla monoculus* (Actinopterygii: Cichlidae); freshwater; intestine, stomach; adult; Amazon and Paraná River basins; Brazil, Peru (Diesing 1850; Woodland 1933b; Rego et al. 1999b; Machado et al. 2000; de Chambrier et al. 2006a, 2015b).

Notes: type host. *Cichla monoculus* should be synonymized with *Cichla ocellaris* based on molecular data (Willis et al. 2012), but it is accepted by morphology-based studies (Kullander and Ferreira 2006). Sequence of partial 28S (KP729403) (de Chambrier et al. 2015b).

*Cichla piquiti* (Actinopterygii: Cichlidae); freshwater; intestine; adult; Paraná and

Tocantins-Araguaia River basins; Brazil (Franceschini et al. 2013; Yamada and Takemoto 2013).

###### Subfamily Endorchiinae Woodland, 1934

***Endorchis auchenipteri* de Chambrier & Vaucher, 1999**

*Auchenipterus osteomystax* (Actinopterygii: Auchenipteridae); freshwater; intestine; adult; Paraná River basin; Paraguay (de Chambrier and Vaucher 1999).

Note: type host.

*Endorchis piraeeba* Woodland, 1934*

*Brachyplatystoma filamentosum* (Actinopterygii: Pimelodidae); freshwater; intestine; adult; Amazon River basin; Brazil; (Woodland 1934b; Rego 1991; de Chambrier and Vaucher 1997; Zehnder and Mariaux 1999; Zehnder et al. 2000; Hypša et al. 2005).

Notes: type host. In type material, *Nominoscolex piraeeba* and *Endorchis piraeeba* are mixed on the same slide (de Chambrier and Vaucher 1997); Rego (1991) synomymized *Endorchis piraeeba* with the former species, but de Chambrier and Vaucher (1997) re-validated it. Sequences of partial 18S (AY551107), complete ITS2 (AY551142), partial 28S (AJ388603) and 16S (AJ389522) (Zehnder and Mariaux 1999; Hypša et al. 2005).

Brachyplatystoma cf. filamentosum (Actinopterygii: Pimelodidae); freshwater; intestine; adult; Amazon River basin; Peru (de Chambrier et al. 2015a).

***Endorchis* sp.**

*Pimelodus altissimus* (Actinopterygii: Pimelodidae); freshwater; intestine; adult; Amazon River basin; Peru (de Chambrier et al. 2015a).

Pimelodus cf. maculatus (Actinopterygii: Pimelodidae); freshwater; intestine; adult; Paraná River basin; Paraguay (de Chambrier and Vaucher 1999).

*Trachelyopterus striatulus* (Actinopterygii: Auchenipteridae); freshwater; intestine; adult; Paraná River basin; Paraguay (de Chambrier and Vaucher 1999).

###### Subfamily Ephedrocephalinae Mola, 1929

*Ephedrocephalus microcephalus* Diesing, 1850*

[Syn. *Rudolphiella microcephalus* (Diesing, 1850) Brooks, 1995]

*Phractocephalus hemioliopterus* (Actinopterygii: Pimelodidae); freshwater; intestine; adult; Amazon River basin; Brazil (Diesing 1850, 1855; Mola 1906; Woodland 1933b; Fuhrmann 1934; Rego 1984b; Zehnder and Mariaux 1999; Hypša et al. 2005; Ruedi and de Chambrier 2012; Scholz et al. 2013).

Notes: type host. Sequences of complete and partial 18S (KC786007, AY551108), respectively; complete ITS2 (AY551143), partial 28S (KC786017, AJ388605), partial 16S (KC785994, AJ389509) and partial *cox*1 (KC785982) (Zehnder and Mariaux 1999; Hypša et al. 2005; Scholz et al. 2013).

###### Subfamily Monticelliinae Mola, 1929

*Ageneiella brevifilis* de Chambrier & Vaucher, 1999*

*Ageneiosus inermis* (Actinopterygii: Auchenipteridae); freshwater; intestine; adult; Paraná River basin; Argentina, Paraguay (de Chambrier and Vaucher 1999; Zehnder and Mariaux 1999; Gil de Pertierra 2009).

Notes: type host; it was reported as *Ageneiosus brevifilis*. Sequences of partial 18S (AY551102), complete ITS2 (AY551138), partial 28S (AJ388600) and 16S (AJ389495) (Zehnder and Mariaux 1999; Hypša et al. 2005).

*Ageneiosus militaris* (Actinopterygii: Auchenipteridae); freshwater; intestine; adult; Paraná River basin; Argentina (Gil de Pertierra 2009).

***Ageneiella* sp.**

*Ageneiosus inermis* (Actinopterygii: Auchenipteridae); freshwater; intestine; adult; Amazon River basin; Peru (de Chambrier et al. 2015a).

*Chambriella agostinhoi* (Pavanelli & Machado, 1992) Rego, Chubb & Pavanelli, 1999*

[Syn. *Goezeella agostinhoi* Pavanelli & Machado, 1992]

*Pimelodus maculatus* (Actinopterygii: Pimelodidae); freshwater; intestine; adult; Paraná River basin; Brazil (Bachmann et al. 2007).

Notes: tapeworms reported as *Goezeella agostinhoi*. This report needs verification, but apparently, there are no vouchers deposited in any museum collection.

*Zungaro jahu* (Actinopterygii: Pimelodidae); freshwater; intestine; adult; Paraná

River basin; Brazil (Pavanelli and Machado 1991, 1992; Takemoto and Pavanelli 1994; Ceccarelli et al. 2006; Kohn et al. 2011).

Notes: type host; it was reported as *Zungaro zungaro* or *Paulicea luetkeni* and the tapeworms as *Goezeella agostinhoi* by some authors. Before formal description of the species, Pavanelli and Machado (1991) had used the name *Goezeella agostinhoi*. Since *Robertiella agostinhoi* and *Rhinebothrium paranaensis* sensu Rego (1999) were not formally described, we considered them as *nomina nuda*.

*Zungaro zungaro* (Actinopterygii: Pimelodidae); freshwater; intestine; adult; Amazon River basin; Peru (de Chambrier et al. 2006a, 2015a).

***Chambriella paranaensis* (Pavanelli & Rego, 1989) Rego, Chubb & Pavaneli, 1999**

[Syns. *Goezeella paranaensis* Pavanelli & Rego, 1989; *Spatulifer paranensis* (sic!) (Pavanelli & Rego, 1989) Brooks, 1995]

*Hemisorubim platyrhynchos* (Actinopterygii: Pimelodidae); freshwater; intestine; adult; Amazon and Paraná River basins; Brazil, Paraguay, Peru (Pavanelli and Rego 1989; Pavanelli and Machado 1991; de Chambrier and Vaucher 1999; Guidelli et al. 2003; de Chambrier et al. 2006a, 2015a).

***Chambriella* sp.**

*Brachyplatystoma vaillantii* (Actinopterygii: Pimelodidae); freshwater; intestine; adult; Amazon River basin; Peru (de Chambrier et al. 2015a).

Note: *Chambriella* sp 1. sensu de Chambrier et al. (2015a).

*Phractocephalus hemioliopterus* (Actinopterygii: Pimelodidae); freshwater; intestine; adult; Amazon River basin; Brazil, Peru (de Chambrier et al. 2006a, 2015a; Ruedi and de Chambrier 2012).

Note: *Chambriella* sp. 2 sensu de Chambrier et al. (2015a).

*Pseudoplatystoma fasciatum* (Actinopterygii: Pimelodidae); freshwater; intestine; adult; Amazon River basin; Peru (de Chambrier et al. 2015a).

Note: *Chambriella* sp. 3 sensu de Chambrier et al. (2015a).

*Sorubimichthys planiceps* (Actinopterygii: Pimelodidae); freshwater; intestine; adult; Amazon River basin; Brazil, Peru (de Chambrier and Scholz 2008; de Chambrier et al. 2015a).

Note: *Chambriella* sp. 4 sensu de Chambrier et al. (2015a).

*Choanoscolex abscisus* (Riggenbach, 1895) La Rue, 1911*

[Syns. *Ichthyotaenia abscisa* Riggenbach, 1895; *Corallobothrium abscissus* (sic!) (Riggenbach, 1895) Meggitt, 1927; *Proteocephalus abscissus* (sic!) (Riggenbach, 1895) Fuhrmann, 1933; *Spatulifer abscissus* (sic!) (Riggenbach, 1895) Brooks, 1995]

*Pseudoplatystoma corruscans* (Actinopterygii: Pimelodidae); freshwater; intestine; adult; Paraná and São Francisco River basins; Brazil, Paraguay (Riggenbach 1895, 1896b; La Rue 1911, 1914; Rego and Gibson 1989; Rego 1990, 2002; Pavanelli and Machado 1991; Machado et al. 1994, 1995, 1996; Mariaux 1998; de Chambrier and Vaucher 1999; Zehnder and Mariaux 1999; Ceccarelli et al. 2006; Kohn et al. 2011; Ribeiro and Takemoto 2014; Ribeiro et al. 2014).

Notes: type host. Riggenbach (1895, 1896b) reported the host as *Silurus* sp. and proposed the parasite name twice. Rego and Gibson (1989) reported hyperparasitism caused by metacestodes of proteocephalids. Sequences of partial 18S (AY551105, Z98382, Z98381, Z98380), complete ITS2 (AY551141), partial 28S (AJ388630) and 16S (AJ389501) (Mariaux 1998; Zehnder and Mariaux 1999; Hypša et al. 2005).

*Pseudoplatystoma fasciatum* (Actinopterygii: Pimelodidae); freshwater; intestine; adult; Amazon, Orinoco, Paraná and São Francisco River basins; Brazil, Peru, Venezuela (Brooks and Rasmussen 1984; Rego 1990, 2002; Zehnder et al. 2000; de Chambrier et al. 2004b, 2015a; Ceccarelli et al. 2006; Campos et al. 2008, 2009a, b; Jerônimo et al. 2013).

Notes: tapeworms reported as Choanoscolex cf. abscisus by Zehnder et al. (2000) and de Chambrier et al. (2004b). Host reported as *Pseudoplatystoma reticulatum* by Jerônimo et al. (2013), but it falls within the range of genetic variability of *Pseudoplatystoma fasciatum* (*sensu lato*), according to Carvalho-Costa et al. (2011). Sequence of partial 28S (AJ275064) (Zehnder et al. 2000).

*Rhaphiodon vulpinus* (Actinopterygii: Cynodontidae); freshwater; intestine; adult; Paraná River basin; Brazil (Rego and Pavanelli 1990).

*Zungaro jahu* (Actinopterygii: Pimelodidae); freshwater; intestine; adult; Paraná

River basin; Brazil (Pavanelli and Machado 1991; Ceccarelli et al. 2006).

Notes: host reported as *Paulicea luetkeni* or *Zungaro zungaro*; it was considered an accidental host by de Chambrier and Vaucher (1999).

***Choanoscolex* sp.**

*Pseudoplatystoma fasciatum* (Actinopterygii: Pimelodidae); freshwater; intestine; adult; Amazon and Paraná River basins; Paraguay, Peru (de Chambrier and Vaucher 1999; de Chambrier et al. 2006a).

*Pseudoplatystoma tigrinum* (Actinopterygii: Pimelodidae); freshwater; intestine; adult (immature specimens); Amazon River basin; Peru (de Chambrier et al. 2006a).

*Sorubimichthys planiceps* (Actinopterygii: Pimelodidae); freshwater; intestine; adult (mostly immature specimens); Amazon River basin; Brazil, Peru (de Chambrier and Scholz 2008; de Chambrier et al. 2015a).

***Goezeella danbrooksi* de Chambrier, Rego & Mariaux, 2004**

[Syn. *Goezeella siluri* sensu Brooks & Deardorff, 1980]

*Ageneiosus pardalis* (Actinopterygii: Auchenipteridae); freshwater; intestine; adult; Magdalena River Basin; Colombia (Brooks and Deardorff 1980; de Chambrier et al. 2004a).

Note: type host; it was originally reported as *Ageneiosus caucanus*, whereas the tapeworms have been reported as *Goezeella siluri* following Brooks and Deardorff’s (1980) description.

*Goezeella siluri* Fuhrmann, 1916*

[Syns. *Goezeella piramutab* Woodland, 1933; *Monticellia piramutab* (Woodland, 1933) Woodland, 1935; *Monticellia siluri* (Fuhrmann, 1916) Woodland, 1935; *Corallobothrium siluri* (Fuhrmann, 1916) Harwood, 1933; *Spatulifer piramutab* (Woodland, 1933) Brooks & Deardorff, 1980; *Spatulifer siluri* (Fuhrmann, 1916) Brooks, 1995]

*Brachyplatystoma vaillantii* (Actinopterygii: Pimelodidae); freshwater; intestine; adult (immature and mature specimens); Amazon and Orinoco River basins; Brazil, Venezuela (Woodland 1933c; Brooks and Rasmussen 1984; de Chambrier et al. 2004a).

Note: the specimens studied by Woodland (1933c) were described as *Goezeella piramutab* and it corresponds in fact to a mixed infection of the present species and *Brooksiella praeputialis*, according to de Chambrier et al. (2004a).

*Cetopsis coecutiens* (Actinopterygii: Cetopsidae); freshwater; intestine; adult; Amazon River basin; Brazil (Fuhrmann 1916; Rego et al. 1974; Rego 1975; de Chambrier and Vaucher 1999; de Chambrier et al. 2004a).

Note: type host.

*Cetopsis othonops* (Actinopterygii: Cetopsidae); freshwater; intestine; adult; Orinoco River basin; Venezuela (Brooks and Rasmussen 1984).

Note: host reported as *Pseudocetopsis othonops*.

*Pinirampus pirinampu* (Actinopterygii: Pimelodidae); freshwater; intestine; adult; Amazon River basin; Brazil (Zehnder and Mariaux 1999; de Chambrier et al. 2004a; Hypša et al. 2005).

Note: sequences of partial 18S (AY551110), complete ITS2 (AY551146), partial 28S (AJ388612) and 16S (AJ389518) (Zehnder and Mariaux 1999; Hypša et al. 2005).

*Lenhataenia megacephala* (Woodland, 1934) de Chambrier & Scholz, 2008*

[Syn. *Monticellia megacephala* Woodland, 1934]

*Rhamdia quelen* (Actinopterygii: Heptapteridae); freshwater; intestine, stomach; adult; Chascomus lagoon (Salado River basin); Argentina (Rabey 1973).

Note: host reported as *Rhamdia sapo*.

*Sorubimichthys planiceps* (Actinopterygii: Pimelodidae); freshwater; intestine; adult; Amazon River basin; Brazil, Peru (Woodland 1934c; Rego 1975; de Chambrier and Scholz 2008; de Chambrier et al. 2015a).

Notes: type host; it was originally reported as *Platystomatichthys sturio*.

*Manaosia bracodemoca* Woodland, 1935*

[Syns. *Paramonticellia itaipuensis* Pavanelli & Rego, 1991; *Goezeella nupeliensis* Pavanelli & Rego, 1991; *Spatulifer nupeliensis* (Pavanelli & Rego, 1991) Brooks, 1995]

*Hemisorubim platyrhynchos* (Actinopterygii: Pimelodidae); freshwater; intestine; adult; Paraná River basin; Brazil (Pavanelli and Machado 1991).

*Sorubim lima* (Actinopterygii: Pimelodidae); freshwater; intestine; adult; Amazon and Paraná River basins; Brazil, Paraguay, Peru (Woodland 1935b; Pavanelli and Machado 1991; Pavanelli and Rego 1991; de Chambrier and Vaucher 1999; Pavanelli and Takemoto 2000; Takemoto and Pavanelli 2000; Rego 2002; de Chambrier 2003; Kohn et al. 2011; de Chambrier et al. 2015a, b).

Notes: type host. Woodland (1935b) reported the host as *Platystoma* sp., but it is supposed to be *Sorubim lima*, locally known as 'braço-de-moça' (de Chambrier 2003). Sequence of partial 28S (KP729414) (de Chambrier et al. 2015b).

***Monticellia amazonica* de Chambrier & Vaucher, 1997**

[Syns. *Nomimoscolex piracatinga* Woodland, 1935; *Monticellia rugata* Rego, 1975 (*pro parte*); *Spatulifer rugata* (Rego, 1975) Brooks & Deardorff, 1980; *Paramonticellia piracatinga* (Woodland, 1935) Brooks, 1995]

*Calophysus macropterus* (Actinopterygii: Pimelodidae); freshwater; intestine; adult; Amazon River basin; Brazil, Peru (Woodland 1935b; Rego 1975; de Chambrier and Vaucher 1997; de Chambrier et al. 2006a, 2015a; Scholz et al. 2008).

Note: type host; it was originally reported as *Pimelodus pati* (syn. of *Luciopimelodus pati* according to Froese and Pauly 2016), but this host does not occur in the Amazon River basin; ‘piracatinga’ is also the vernacular name of *Calophysus macropterus*, which is endemic to the Amazon and Orinoco River basins (for details, see Scholz et al. 2008).

***Monticellia belavistensis* Pavanelli, Machado, Takemoto & dos Santos, 1994**

*Pterodoras granulosus* (Actinopterygii: Doradidae); freshwater; intestine; adult; Amazon and Paraná River basins; Argentina, Brazil, Paraguay, Peru (Pavanelli et al. 1994; de Chambrier and Vaucher 1999; Gil de Pertierra 2005; Kohn et al. 2011; de Chambrier et al. 2015a).

Note: type host.

*Monticellia coryphicephala* (Monticelli, 1891) La Rue, 1911*

[Syns. *Taenia coryphicephala* Monticelli, 1891; *Tetracotylus coryphicephala* Monticelli, 1891; *Ichthyotaenia coryphicephala* (Monticelli, 1891) Lönnberg, 1894; Proteocephalus (Proteocephalus) coryphicephala (Monticelli, 1891) Harwood, 1933]

*Salminus brasiliensis* (Actinopterygii: Bryconidae); freshwater; intestine; adult; Paraná and São Francisco River basins; Brazil, Paraguay (Monticelli 1891; La Rue 1911, 1914; Rego 1975; Rego and Pavanelli 1990; Pavanelli and Machado 1991; de Chambrier and Vaucher 1999; Zehnder and Mariaux 1999; Zehnder and de Chambrier 2000; Brasil-Sato 2003; Mesquita et al. 2012; Karling et al. 2013a, b).

Notes: type host; it was originally reported as *Silurus* sp., but this genus only occurs in the Palaearctic region. Sequences of complete ITS2 (AJ238839), partial 28S (AJ238832) and 16S (AJ238831) (Zehnder and Mariaux 1999; Zehnder and de Chambrier 2000).

*Salminus franciscanus* (Actinopterygii: Bryconidae); freshwater; intestine; adult; São Francisco River basin; Brazil (Rego and Pavanelli 1990).

Note: host reported as *Salminus brevidens*.

***Monticellia dlouhyi* de Chambrier & Vaucher, 1999**

*Acestrorhynchus altus* (Actinopterygii: Acestrorhynchidae); freshwater; intestine; adult; Paraná River basin; Paraguay (de Chambrier and Vaucher 1999).

Note: type host.

***Monticellia magna* (Rego, Santos & Silva, 1974) de Chambrier & Vaucher, 1997**

[Syns. *Nomimoscolex magna* Rego Santos & Silva, 1974 (*pro parte*); *Monticellia loyolai* Pavanelli & Machado, 1992]

*Pimelodus albicans* (Actinopterygii: Pimelodidae); freshwater; intestine; adult; Paraná River basin; Argentina (Gil de Pertierra 2004).

*Pimelodus argenteus* (Actinopterygii: Pimelodidae); freshwater; intestine; adult; Paraná River basin; Argentina (Gil de Pertierra 2004).

Pimelodus cf. blochii (Actinopterygii: Pimelodidae); freshwater; intestine; adult; Paraná River basin; Paraguay (de Chambrier and Vaucher 1999).

*Pimelodus maculatus* (Actinopterygii: Pimelodidae); freshwater; intestine; adult; Paraná and São Francisco River basins; Brazil, Argentina (Rego et al. 1974; Pavanelli and Machado 1991, 1992; de Chambrier and Vaucher 1997, 1999; Brasil-Sato 2003; Gil de Pertierra 2004; Kohn et al. 2011).

Notes: type host; it was reported as *Pimelodus clarias*. There is a mixture of two species in the type material, originally described as *Nomimoscolex magna*, which can be differentiated by the position of internal organs (see p. 255 in de Chambrier and Vaucher (1997); Rego et al. (1999a) proposed the name *Proteocephalus magna* for those specimens considered as *Proteocephalus* sp. by the former authors, but they superficially circumscribed the new taxon.

Pimelodus cf. maculatus (Actinopterygii: Pimelodidae); freshwater; intestine; adult; Paraná River basin; Paraguay (de Chambrier and Vaucher 1999).

Note: they reported the tapeworms as Monticellia cf. magna.

***Monticellia santafesina* Arredondo & Gil de Pertierra, 2010**

*Megalonema platanum* (Actinopterygii: Pimelodidae); freshwater; intestine; adult; Paraná River basin; Argentina (Arredondo and Gil de Pertierra 2010).

Note: type host.

*Megalonema platycephalum* (Actinopterygii: Pimelodidae); freshwater; intestine; adult; Amazon River basin; Peru (de Chambrier et al. 2015a).

***Monticellia ventrei* de Chambrier & Vaucher, 1999**

[Syn. *Myzophorus admonticellia* Woodland 1934 (*pro parte*)]

*Luciopimelodus pati* (Actinopterygii: Pimelodidae); freshwater; intestine; adult; Paraná River basin; Argentina (Gil de Pertierra 2005).

*Pinirampus pirinampu* (Actinopterygii: Pimelodidae); freshwater; intestine; adult; Amazon and Paraná River basins; Brazil, Paraguay, Peru (Woodland 1934a; de Chambrier and Vaucher 1999; de Chambrier et al. 2015a).

Notes: this host is assumed to be the type, because all possible fish hosts cited by Woodland (1934a), i.e. *Pimelodus pinarampu*, *Pinirampus pirinampus* (sic!) and *Pinirampus typus* are junior synonym of *Pinirampus pirinampu* (see Froese and Pauly 2016). *Nomimoscolex admonticellia* and *Monticellia ventrei* are mixed in the type material of *Myzophorus admonticellia*, according to de Chambrier and Vaucher (1999).

***Monticellia* sp.**

*Brycon orbignyanus* (Actinopterygii: Bryconidae); freshwater; intestine; adult (immature specimens); Paraná River basin; Paraguay (de Chambrier and Vaucher 1999).

*Pinirampus pirinampu* (Actinopterygii: Pimelodidae); freshwater; intestine; adult; Paraná River basin; Paraguay (de Chambrier and Vaucher 1999).

*Pseudoplatystoma corruscans* (Actinopterygii: Pimelodidae); freshwater; intestine; adult; São Francisco River basin; Brazil (Brasil-Sato 2003; Santos et al. 2003).

*Pseudoplatystoma fasciatum* (Actinopterygii: Pimelodidae); freshwater; intestine; adult; Paraná River basin; Paraguay (de Chambrier and Vaucher 1999; Santos et al. 2003).

*Regoella brevis* Arredondo, Gil de Pertierra & de Chambrier, 2013*

*Pseudoplatystoma fasciatum* (Actinopterygii: Pimelodidae); freshwater; intestine; adult; Paraná River basin; Argentina (Arredondo et al. 2013; de Chambrier et al. 2015b).

Notes: type host. Sequence of partial 28S (KP729389) (de Chambrier et al. 2015b). Host recorded as *Pseudoplatystoma reticulatum* in the GenBank database.

*Spasskyellina lenha* (Woodland, 1933) Freze, 1965*

[Syn. *Monticellia lenha* Woodland, 1933]

*Sorubimichthys planiceps* (Actinopterygii: Pimelodidae); freshwater; intestine; adult; Amazon River basin; Brazil, Peru (Woodland 1933a; de Chambrier and Scholz 2008; de Chambrier et al. 2015a, b).

Notes: type host. Sequence of 28S (KP729413) under the name *Lenhataenia megacephala* in the GenBank database – see de Chambrier et al. (2015b).

***Spasskyellina mandi* Pavanelli & Takemoto, 1996**

[Syn. *Monticellia mandi* (Pavanelli & Takemoto, 1996) de Chambrier & Vaucher, 1999]

*Pimelodus ornatus* (Actinopterygii: Pimelodidae); freshwater; intestine; adult; Paraná River basin; Brazil (Pavanelli and Takemoto 1996).

Note: type host.

***Spasskyellina spinulifera* (Woodland, 1935) Freze, 1965**

[Syns. *Monticellia spinulifera* (Woodland, 1935); *Monticellia spinulifer* (sic!) of Brooks (1995)]

*Pseudoplatystoma corruscans* (Actinopterygii: Pimelodidae); freshwater; intestine; adult; Paraná River basin; Argentina, Brazil, Paraguay (Rego 1990, 2002; Pavanelli and Machado 1991; Machado et al. 1994, 1995, 1996; de Chambrier and Vaucher 1999; Santos et al. 2003; Ceccarrelli et al. 2006; Kohn et al. 2011; Ribeiro et al. 2014; de Chambrier et al. 2015b).

Note: sequence of 28S (KP729417) (de Chambrier et al. 2015b).

*Pseudoplatystoma fasciatum* (Actinopterygii: Pimelodidae); freshwater; intestine; adult; Amazon and Paraná River basins; Brazil, Peru (Woodland 1935a; Rego 1975, 2002; de Chambrier and Vaucher 1999; Santos et al. 2003; de Chambrier et al. 2006a, 2015a; Ceccarrelli et al. 2006).

Note: type host.

*Pseudoplatystoma tigrinum* (Actinopterygii: Pimelodidae); freshwater; intestine; adult; Amazon River basin; Peru (de Chambrier et al. 2006a, 2015a).

*Sorubim lima* (Actinopterygii: Pimelodidae); freshwater; intestine; adult; Paraná River basin; Brazil (Pavanelli and Takemoto 2000; Takemoto and Pavanelli 2000).

***Spasskyellina* sp.**

*Pimelodus ornatus* (Actinopterygii: Pimelodidae); freshwater; intestine; adult; Paraná River basin; Brazil (Takemoto et al. 2009).

*Pseudoplatystoma fasciatum* (Actinopterygii: Pimelodidae); freshwater; intestine; adult; Paraná River basin; Brazil (Santos et al. 2003).

***Spatulifer maringaensis* Pavanelli & Rego, 1989**

*Hemisorubim platyrhynchos* (Actinopterygii: Pimelodidae); freshwater; intestine, stomach; adult; Amazon and Paraná River basins; Brazil, Paraguay, Peru (Pavanelli and Rego 1989; Pavanelli and Machado 1991; de Chambrier and Vaucher 1999; Guidelli et al. 2003; de Chambrier et al. 2006a, 2015a).

Note: type host.

*Sorubim lima* (Actinopterygii: Pimelodidae); freshwater; intestine; adult; Amazon

and Paraná River basins; Argentina, Brazil, Paraguay, Peru (Pavanelli and Rego 1989; Pavanelli and Machado 1991; Mariaux 1998; de Chambrier and Vaucher 1999; Zehnder and Mariaux 1999; Pavanelli and Takemoto 2000; Takemoto and Pavanelli 2000; Hypša et al. 2005; Arrendondo and Gil de Pertierra 2008; de Chambrier et al. 2015a).

Notes: Arredondo and Gil de Pertierra (2008) suggested, based on ecological data, that *Sorubim lima* is the principal final host. Tapeworms reported as Spatulifer cf. maringaensis by de Chambrier and Vaucher (1999) and confirmed to be *Spatulifer maringaensis* by the former authors, who evaluated the vouchers deposited in MHNG. Sequences of partial 18S (AY551136, Z98385, Z98384, Z98383), complete ITS2 (AY551176), partial 28S (AJ388634) and 16S (AJ389507) (Mariaux 1998; Zehnder and Mariaux 1999; Hypša et al. 2005).

***Spatulifer rugosa* (Woodland, 1935) Brooks & Deardorff, 1980**

[Syn. *Monticellia rugosa* Woodland, 1935]

*Pseudoplatystoma fasciatum* (Actinopterygii: Pimelodidae); freshwater; intestine; adult; Amazon and Paraná River basins; Argentina, Brazil, Peru (Woodland 1935b; Rego 1975, 1989, 2002; de Chambrier et al. 2006a, 2015a; Arrendondo and Gil de Pertierra 2008; Campos et al. 2008, 2009a, b; Lopes et al. 2009).

Notes: type host; it was reported as *Pseudoplatystoma punctifer* by de Chambrier et al. (2006a) and Lopes et al. (2009).

*Spatulifer surubim* Woodland, 1934*

[Syns. *Peltidocotyle rugosa* sensu Woodland, 1933b *nec* Diesing, 1850; *Spatulifer surubim* Woodland, 1934; *Monticellia surubim* (Woodland, 1934) Woodland, 1935]

*Pseudoplatystoma tigrinum* (Actinopterygii: Pimelodidae); freshwater; intestine; adult; Amazon River basin; Brazil (Woodland 1933b, 1934a; Rego 1975, 2002).

Note: type host.

***Spatulifer* sp.**

*Pseudoplatystoma tigrinum* (Actinopterygii: Pimelodidae); freshwater; intestine; adult (immature specimens); Amazon River basin; Peru (de Chambrier et al. 2006a, 2015a).

Note: probably *Spatulifer surubim* according to de Chambrier et al. (2006a).

**Monticelliinae gen. sp.**

*Phractocephalus hemioliopterus* (Actinopterygii: Pimelodidae); freshwater; intestine; adult; Amazon River basin; Peru (de Chambrier et al. 2015a).

###### Subfamily Nupeliinae Pavanelli & Rego, 1991

*Nupelia portoriquensis* Pavanelli & Rego, 1991*

*Sorubim lima* (Actinopterygii: Pimelodidae); freshwater; intestine; adult; Paraná

River basin; Brazil, Paraguay (Pavanelli and Machado 1991; Pavanelli and Rego 1991; de Chambrier and Vaucher 1999; Pavanelli and Takemoto 2000; Takemoto and Pavanelli 2000; de Chambrier et al. 2015b).

Notes: type host. Sequence of partial 28S (KP729401) (de Chambrier et al. 2015b).

***Nupelia tomasi* de Chambrier & Vaucher, 1999**

*Trachelyopterus galeatus* (Actinopterygii: Auchenipteridae); freshwater; intestine; adult; Paraná River basin; Paraguay (de Chambrier and Vaucher 1999).

Note: type host.

Trachelyopterus cf. striatulus (Actinopterygii: Auchenipteridae); freshwater; intestine; adult; Paraná River basin; Paraguay (de Chambrier and Vaucher 1999).

***Nupelia* sp.**

*Goeldiella eques* (Actinopterygii: Heptapteridae); freshwater; intestine; adult; Amazon River basin; Peru (de Chambrier et al. 2006a, 2015a).

###### Subfamily Peltidocotylinae Woodland, 1934

*Amazotaenia yvettae* de Chambrier, 2001*

*Brachyplatystoma capapretum* (Actinopterygii: Pimelodidae); freshwater; intestine; adult; Amazon River basin; Brazil (de Chambrier 2001).

Notes: type host; it was reported as *Brachyplatystoma filamentosum* and re-identified by J. Lundberg (pers. comm.).

*Brachyplatystoma vaillantii* (Actinopterygii: Pimelodidae); freshwater; intestine; adult; Amazon River basin; Brazil (de Chambrier 2001).

*Jauella glandicephalus* Rego & Pavanelli, 1985*

[Syn. *Spatulifer glandicephala* (Rego & Pavanelli, 1985) Brooks, 1995]

*Zungaro jahu* (Actinopterygii: Pimelodidae); freshwater; intestine; adult; Paraná

River basin; Argentina, Brazil, Paraguay (Rego and Pavanelli 1985; Eiras et al. 1986; Rego and Gibson 1989; Pavanelli and Machado 1991; Takemoto and Pavanelli 1994; de Chambrier and Vaucher 1999; Gil de Pertierra 2009; de Chambrier et al. 2015b).

Notes: type host; it was reported as *Paulicea luetkeni* or *Zungaro zungaro*. Rego and Pavanelli (1985) proposed the subfamily Jauellinae Rego and Pavanelli, 1985, which was not followed by other workers. Rego and Gibson (1989) and Rego and Pavanelli (1985) reported hyperparasitism caused by metacestodes of proteocephalids. Sequence of partial 28S (KP729399) (de Chambrier et al. 2015b).

*Zungaro zungaro* (Actinopterygii: Pimelodidae); freshwater; intestine; adult; Amazon River basin; Peru (de Chambrier et al. 2015a).

*Luciaella ivanovae* Gil de Pertierra, 2009*

*Ageneiosus inermis* (Actinopterygii: Auchenipteridae); freshwater; intestine; adult; Paraná River basin; Argentina (Gil de Pertierra 2009).

Note: type host.

*Mariauxiella pimelodi* de Chambrier & Rego, 1995*

*Pimelodus ornatus* (Actinopterygii: Pimelodidae); freshwater; intestine; adult; Paraná River basin; Brazil, Paraguay (de Chambrier and Rego 1995; de Chambrier and Vaucher 1999).

*Pimelodus* sp. (Actinopterygii: Pimelodidae); freshwater; intestine; adult; Paraná River basin; Brazil (de Chambrier and Rego 1995; de Chambrier and Vaucher 1999).

Note: type host.

***Mariauxiella piscatorum* de Chambrier & Vaucher, 1999**

*Hemisorubim platyrhynchos* (Actinopterygii: Pimelodidae); freshwater; intestine; adult; Amazon and Paraná River basins; Brazil, Paraguay, Peru (de Chambrier and Vaucher 1999; Guidelli et al. 2003; de Chambrier et al. 2006a, 2015a).

Note: type host.

***Peltidocotyle lenha* (Woodland, 1933) Woodland, 1934**

[Syns. *Othinoscolex lenha* Woodland, 1933; *Othinoscolex myzofer* Woodland, 1933; *Woodlandiella myzofera* (Woodland, 1933) Freze, 1965; *Peltidocotyle rugosa* of Schmidt, 1986; *Rudolphiella lenha* (Woodland, 1933) Brooks, 1995]

*Sorubimichthys planiceps* (Actinopterygii: Pimelodidae); freshwater; intestine; adult; Amazon River basin; Brazil, Peru (Woodland 1933a, 1934b; Zehnder and Mariaux 1999; Zehnder and de Chambrier 2000; Hypša et al. 2005; de Chambrier and Scholz 2008; de Chambrier et al. 2015a).

Notes: type host; it was originally reported as *Platystomatichthys sturio*. Sequences of partial 18S (AY551122), complete IT2 (AJ238842), partial 28S (AJ238836) and 16S (AJ238827) (Zehnder and Mariaux 1999; Zehnder and de Chambrier 2000; Hypša et al. 2005).

*Zungaro jahu* (Actinopterygii: Pimelodidae); freshwater; intestine; adult; Paraná

River basin; Argentina, Brazil (Rego and Pavanelli 1987; Zehnder and de Chambrier 2000; Gil de Pertierra 2009).

Notes: host reported as *Zungaro zungaro* or *Paulicea luetkeni*. Rego and Pavanelli (1987) mistakenly reported the tapeworm as *Peltidocotyle rugosa*, according to Zehnder and de Chambrier (2000); the former workers also reported hyperparasitism caused by larval cestodes.

*Zungaro zungaro* (Actinopterygii: Pimelodidae); freshwater; intestine; adult; Amazon River basin; Brazil, Peru (Zehnder and Mariaux 1999; de Chambrier et al. 2006a).

Notes: sequences of complete IT2 (AJ238840, AJ238843), partial 28S (AJ238834, AJ238837) and 16S (AJ238826, AJ238829) (Zehnder and Mariaux 1999; Zehnder and de Chambrier 2000).

*Peltidocotyle rugosa* Diesing, 1850*

*Pseudopimelodus mangurus* (Actinopterygii: Pseudopimelodidae); freshwater; intestine; adult; locality not given; Argentina (Rego and Pavanelli 1987).

Note: host reported as *Zungaro mangurus*.

*Pseudoplatystoma corruscans* (Actinopterygii: Pimelodidae); freshwater; intestine; adult; Paraná River basin; Argentina, Brazil (Diesing 1850; Fuhrmann 1934; Rego 1990, 2002; de Chambrier and Vaucher 1999; Zehnder and de Chambrier 2000; Gil de Pertierra 2009).

Note: type host; it was originally reported as *Platystoma tigrinum* (syn. of *Pseudoplatystoma tigrinum*), but it does not occur in the Paraná River basin, thus the fish host is assumed to be *Pseudoplatystoma corruscans* (see de Chambrier and Vaucher 1999).

*Pseudoplatystoma fasciatum* (Actinopterygii: Pimelodidae); freshwater; intestine; adult; Amazon and Paraná River basins; Brazil, Paraguay, Peru (Rego 1989, 1990; de Chambrier and Vaucher 1999; Zehnder and Mariaux 1999; Zehnder and de Chambrier 2000; Olson et al. 2001; Rego 2002; Ceccarrelli et al. 2006; Campos et al. 2008, 2009a, b; de Chambrier et al. 2015a).

Note: sequence of complete 18S (AF286989) and ITS2 (AJ238841), partial 28S (AJ238835, AF286937) and 16S (AJ238828) (Zehnder and Mariaux 1999; Zehnder and de Chambrier 2000; Olson et al. 2001).

*Zungaro jahu* (Actinopterygii: Pimelodidae); freshwater; intestine; adult; Paraná

River basin; Brazil (Rego and Gibson 1989; Pavanelli and Machado 1991; Takemoto and Pavanelli 1994; Ceccarrelli et al. 2006; Kohn et al. 2011).

Notes: host reported as *Paulicea luetkeni* or *Zungaro zungaro*. Rego and Gibson (1989) reported hyperparasitism caused by metacestodes of proteocephalids.

***Peltidocotyle* sp.**

*Zungaro jahu* (Actinopterygii: Pimelodidae); freshwater; intestine; adult; Paraná

River basin; Paraguay (de Chambrier and Vaucher 1999).

Note: host reported as *Paulicea luetkeni*.

###### Subfamily Proteocephalinae La Rue, 1911

*Brayela karuatayi* (Woodland, 1934) Rego, 1984*

[Syn. *Anthobothrium karuatayi* Woodland, 1934]

*Platynematichthys notatus* (Actinopterygii: Pimelodidae); freshwater; intestine; adult (also immature specimens); Amazon River basin; Brazil, Peru (Woodland 1934c; Rego 1984a; de Chambrier et al. 2014, 2015a, b).

Notes: type host; it was originally reported as *Glanidium* sp.; Rego (1984a) erected the new subfamily Brayelainae, which was not accepted by other authors. Sequence of partial 28S (KP729406) (de Chambrier et al. 2015b).

*Cangatiella arandasi* Pavanelli & Machado, 1991*

*Trachelyopterus galeatus* (Actinopterygii: Auchenipteridae); freshwater; intestine; adult; Paraná River basin; Brazil (Pavanelli and Machado 1990, 1991; Kohn et al. 2011; de Chambrier et al. 2015b).

Notes: host also reported as *Parauchenipterus galeatus*. Sequence of 28S (KP729411) (de Chambrier et al. 2015b).

Note: type host.

***Cangatiella macdonaghi* (Szidat & Nani, 1951) Gil de Pertierra & Viozzi, 1999** [Syns. *Ichthyotaenia macdonaghi* Szidat & Nani, 1951; *Proteocephalus macdonaghi* (Szidat & Nani, 1951) Yamaguti, 1959]

*Odontesthes bonariensis* (Actinopterygii: Atherinopsidae); freshwater; intestine; adult; lakes in Buenos Aires and Córdoba Provinces; Argentina (MacDonagh 1932; Ringuelet 1943; Fuster de Plaza and Boschi 1957; Ortubay et al. 1994; Mancini and Grosman 1998; Mancini et al. 2008; Drago 2012; Bethular et al. 2014).

Notes: Mancini and Grosman (1998) reported the tapeworms as *Proteocephalus* sp. (Mancini et al. 2008), whereas MacDonagh (1932) and Ringuelet (1943) reported it as *Ichthyotaenia* sp.

*Odontesthes hatcheri* (Actinopterygii: Atherinopsidae); freshwater; intestine; adult; Peligrini lake; Argentina (Szidat and Nani 1951; Gil de Pertierra and Viozzi 1999).

Notes: type host; it was originally reported as *Basilichthys microlepidotus*.

*Euzetiella tetraphylliformis* de Chambrier, Rego & Vaucher, 1999*

*Pseudoplatystoma fasciatum* (Actinopterygii: Pimelodidae); freshwater; intestine; adult (immature); Amazon River basin; Peru (de Chambrier et al. 2015a).

*Zungaro jahu* (Actinopterygii: Pimelodidae); freshwater; intestine; adult; Paraná River basin; Brazil, Paraguay (de Chambrier et al. 1999).

*Zungaro zungaro* (Actinopterygii: Pimelodidae); freshwater; intestine; adult; Amazon River basin; Brazil, Peru (de Chambrier et al. 1999, 2006a, 2015a).

Notes: host originally reported as *Paulicea luetkeni* (syn. of *Zungaro jahu* and *Zungaro zungaro*); since the holotype was described from a fish collected in the Amazon River, *Zungaro zungaro* should be considered the type host.

*Frezella vaucheri* Alves, de Chambrier, Scholz & Luque, 2015*

*Tocantinsia piresi* (Actinopterygii: Auchenipteridae); freshwater; intestine; adult; Amazon River basin; Brazil (Alves et al. 2015).

Notes: type host. Sequence of partial 28S (KM387399) (Alves et al. 2015).

*Margaritaella gracilis* Arredondo & Gil de Pertierra, 2012*

*Callichthys callichthys* (Actinopterygii: Callichthyidae); freshwater; intestine; adult; Paraná River basin; Argentina (Arredondo and Gil de Pertierra 2012).

Note: type host.

***Proteocephalus bagri* Holcman-Spector & Mañé-Garzón, 1988**

*Rhamdia quelen* (Actinopterygii: Heptapteridae); freshwater; intestine; Chis-Chis, Chascomús, Sauce, Diario and Dos Patos lagoons; Argentina, Brazil, Uruguay (Holcman-Spector and Mañé-Garzón 1988; Gil de Pertierra 2002b).

Note: type host; it was originally reported as *Rhamdia sapo*.

***Proteocephalus fossatus* (Riggenbach, 1895) La Rue, 1911**

[Syn. *Ichthyotaenia fossata* Riggenbach, 1895]

*Luciopimelodus pati* (Actinopterygii: Pimelodidae); freshwater; intestine; adult; Paraná River basin; Paraguay (Riggenbach 1895, 1896; La Rue 1911, 1914).

Note: type host; it was originally reported as *Pimelodus pati*.

***Proteocephalus gibsoni* Rego & Pavanelli, 1991**

[Syn. *Proteocephalus ocellatus* sensu Rego & Pavanelli, 1990 *nec Proteocephalus ocellatus* (Rudolphi, 1802)]

*Astronotus ocellatus* (Actinopterygii: Cichlidae); freshwater; intestine; adult; Amazon River basin; Peru (Rego and Pavanelli 1990; de Chambrier et al. 2006a, 2015a; Bittencourt et al. 2014).

Notes: type host. Rego and Pavanelli (1991) proposed the name *Proteocephalus gibsoni* one year later of its original description in order to avoid the homonym with *Proteocephalus ocellatus* (syn. of *Proteocephalus percae* [Müller, 1780]), a parasite of percids in Europe (Scholz 1989).

*Astronotus* sp. (Actinopterygii: Cichlidae); freshwater; intestine; adult; Amazon River basin; Brazil (Rego and Pavanelli 1990).

*Geophagus brasiliensis* (Actinopterygii: Cichlidae); freshwater; intestine; adult; Paraná and Doce River basins; Brazil (Rego and Pavanelli 1990; Bellay et al. 2012).

***Proteocephalus hemioliopteri* de Chambrier & Vaucher, 1997**

[Syns. *Myzophorus woodlandi* Rego, 1984; *Nomimoscolex woodlandi* (Rego, 1984) Rego & Pavanelli, 1992]

*Phractocephalus hemioliopterus* (Actinopterygii: Pimelodidae); freshwater; intestine; adult; Amazon River basin; Brazil, Peru (Rego 1984b; de Chambrier and Vaucher 1997; Zehnder and Mariaux 1999; de Chambrier et al. 2005, 2015a; Hypša et al. 2005; Ruedi and de Chambrier 2012).

Notes: type host. Sequences of partial 18S (AY551129), complete ITS2 (AY551165) and partial 28S (AJ388622) (Zehnder and Mariaux 1999; Hypša et al. 2005).

***Proteocephalus hobergi* de Chambrier & Vaucher, 1999**

*Oxydoras kneri* (Actinopterygii: Doradidae); freshwater; intestine; adult; Amazon

and Paraná River basins; Paraguay, Peru (de Chambrier and Vaucher 1999; de Chambrier et al. 2004b; de Chambrier et al. 2015a).

Notes: type host. Sequence of partial 28S (AJ275062) (de Chambrier et al. 2004b).

*Oxydoras niger* (Actinopterygii: Doradidae); freshwater; intestine; adult; Amazon

River basin; Peru (de Chambrier et al. 2015).

***Proteocephalus kuyukuyu* Woodland, 1935**

*Megalodoras uranoscopus* (Actinopterygii: Doradidae); freshwater; intestine; adult (immature specimens); Amazon and Orinoco River basins; Peru, Venezuela (Brooks and Rasmussen 1984; de Chambrier et al. 2015a).

Note: Brooks and Rasmussen (1984) reported the host as *Megalodoras irwini* and the tapeworms as Proteocephalus cf. kuyukuyu.

*Oxydoras niger* (Actinopterygii: Doradidae); freshwater; intestine; adult (immature specimens); Amazon River basin; Brazil (Woodland 1935c; Santos and Tavares-Dias 2010; Silva et al. 2011).

Notes: type host; it was reported as *Pseudodoras niger*, but Woodland (1935c) also found some specimens in other doradid fish, assumed to be *Pseudodoras brunnescens* (syn. of *Acanthodoras spinosissimus*). It is argued that the presence of only immature proglottids in the adults is due to a hyperapolytic development (see de Chambrier et al. 2015a); considered *species inquirenda* by some authors, e.g. Freze (1965), Rego (1987b) and Rego et al. (1999a).

*Pterodoras granulosus* (Actinopterygii: Doradidae); freshwater; intestine; adult (immature specimens); Amazon River basin; Peru (de Chambrier et al. 2015a, b).

Note: sequence of partial 28S (KP729388) (de Chambrier et al. 2015b).

*Pterodoras* sp. (Actinopterygii: Doradidae); freshwater; intestine; adult (immature specimens); Amazon River basin; Peru (de Chambrier et al. 2015a).

***Proteocephalus macrophallus* (Diesing, 1850) La Rue, 1914**

[Syns. *Taenia macrophalla* Diesing, 1850; *Ichthyotaenia macrophalla* (Diesing, 1850) Riggenbach, 1896]

*Cichla kelberi* (Actinopterygii: Cichlidae); freshwater; intestine; adult; Paraná and São Francisco River basin; Brazil (Yamada and Takemoto 2013; Santos-Clapp and Brasil-Sato 2014).

*Cichla monoculus* (Actinopterygii: Cichlidae); freshwater; intestine; adult; Amazon and Paraná River basins; Brazil, Peru (Diesing 1850; La Rue 1914; Takemoto and Pavanelli 1996; de Chambrier et al. 2006a, 2015a, b; Machado et al. 2000; Kohn et al. 2011).

Notes: type host; some authors assumed *Cichla ocellaris* as the type host, but the taxonomic status of these cichlids is unclear (see notes on p. 33). Sequence of partial 28S (KP729394) (de Chambrier et al. 2015b).

*Cichla ocellaris* (Actinopterygii: Cichlidae); freshwater; intestine; adult; Amazon, Orinoco, Paraíba do Sul and Paraná River basins; Brazil, Venezuela (Woodland 1933b; Scholz et al. 1996; Azevedo et al. 2010, 2011).

*Cichla piquiti* (Actinopterygii: Cichlidae); freshwater; intestine, stomach; adult; Paraná and Tocantins-Araguaia River basins; Brazil (Martins et al. 2009, 2011; Franceschini et al. 2013; Lacerda et al. 2013; Yamada and Takemoto 2013).

*Cichla* sp. (Actinopterygii: Cichlidae); freshwater; intestine; adult; Paraná River basin; Brazil (Santos et al. 2011).

***Proteocephalus mahnerti* de Chambrier & Vaucher, 1999**

*Hoplerythrinus unitaeniatus* (Actinopterygii: Erythrinidae); freshwater; intestine; adult; Paraná River basin; Paraguay (de Chambrier and Vaucher 1999).

Note: type host.

***Proteocephalus microscopicus* Woodland, 1935**

*Cichla kelberi* (Actinopterygii: Cichlidae); freshwater; intestine; adult; Paraná and São Francisco River basins; Brazil (Yamada and Takemoto 2013; Santos-Clapp and Brasil-Sato 2014).

*Cichla monoculus* (Actinopterygii: Cichlidae); freshwater; intestine; adult; Amazon and Paraná River basins; Brazil, Peru (Takemoto and Pavanelli 1996; Machado et al. 2000; de Chambrier et al. 2006a, 2015a; Müller et al. 2008; Kohn et al. 2011).

*Cichla ocellaris* (Actinopterygii: Cichlidae); freshwater; intestine; adult; Amazon River basin; Brazil (Woodland 1935c).

Note: type host.

*Cichla piquiti* (Actinopterygii: Cichlidae); freshwater; intestine; adult; Paraná and Tocantins-Araguaia River basins; Brazil (Martins et al. 2009, 2011; Franceschini et al. 2013; Lacerda et al. 2013; Yamada and Takemoto 2013).

*Cichla* sp. (Actinopterygii: Cichlidae); freshwater; intestine; adult; Paraná River basin; Brazil (Santos et al. 2011).

***Proteocephalus pilarensis* de Chambrier & Vaucher, 1999**

*Paraloricaria* sp. (Actinopterygii: Loricariidae); freshwater; intestine; adult; Paraná River basin; Paraguay (de Chambrier and Vaucher 1999).

Note: type host.

***Proteocephalus pimelodi* (Gil de Pertierra, 1995) de Chambrier & Vaucher, 1997**

[Syn. *Nomimoscolex pimelodi* Gil de Pertierra, 1995]

*Pimelodus maculatus* (Actinopterygii: Pimelodidae); freshwater; intestine; adult; Paraná River basin; Argentina (Gil de Pertierra 1995; de Chambrier and Vaucher 1997).

Note: type host.

***Proteocephalus platystomi* Lynsdale, 1959**

*Pseudoplatystoma* sp. (Actinopterygii: Pimelodidae); freshwater; intestine; adult; Amazon River basin; Brazil (Lynsdale 1959; Rego 2002).

Note: host originally reported as *Platystoma* sp.; the specimens collected by Woodland in 1937 were deposited without any identification in BMNH (Lynsdale 1959).

Note: type host.

***Proteocephalus regoi* de Chambrier, Scholz and Vaucher, 1996**

*Hoplias malabaricus* (Actinopterygii: Erythrinidae); freshwater; intestine; adult; Paraná River basin; Paraguay (de Chambrier et al. 1996; de Chambrier and Vaucher 1999).

Note: type host.

***Proteocephalus renaudi* de Chambrier & Vaucher, 1994**

*Franciscodoras marmoratus* (Actinopterygii: Doradidae); freshwater; intestine; adult; São Francisco River basin; Brazil (Santos and Brasil-Sato 2006).

*Platydoras costatus* (Actinopterygii: Doradidae); freshwater; intestine; adult; Paraná River basin; Paraguay (de Chambrier and Vaucher 1994, 1999; Zehnder and Mariaux 1999).

Notes: type host; it does not occur in the Paraná River basin (Ferraris 2007; Piorski et al. 2008). Therefore, *Platydoras armatulus* (Valenciennes), which is the only known *Platydoras* species from this river basin, is most probably the true host of *Proteocephalus renaudi*. Sequences of partial 28S (AJ388638) and 16S (AJ389503) (Zehnder and Mariaux 1999).

***Proteocephalus rhamdiae* Holcman-Spector & Mañé-Garzón, 1988**

*Rhamdia quelen* (Actinopterygii: Heptapteridae); freshwater; intestine; adult; Chis-Chis, Chascomús, Sauce, Diario and Dos Patos lagoons and Paraná River basin; Argentina, Brazil, Paraguay, Uruguay (Holcman-Spector and Mañé-Garzón 1988; de Chambrier and Vaucher 1999; Gil de Pertierra 2002b).

Note: type host; it was originally reported as *Rhamdia sapo*.

***Proteocephalus serrasalmus* Rego & Pavanelli, 1990**

*Pygocentrus nattereri* (Actinopterygii: Serrasalmidae); freshwater; intestine; adult; Paraná River basin; Brazil (Rego and Pavanelli 1990).

Note: host originally reported as *Serrasalmus nattereri*.

*Serrasalmus maculatus* (Actinopterygii: Serrasalmidae); freshwater; intestine; adult; Paraná River basin; Brazil, Paraguay (Rego and Pavanelli 1990; Pavanelli and Machado 1991; de Chambrier and Vaucher 1999).

Note: type host; it was originally reported as *Serrasalmus spilopleura*.

***Proteocephalus soniae* de Chambrier & Vaucher, 1994**

*Platydoras costatus* (Actinopterygii: Doradidae); freshwater; intestine; adult; Paraná River basin; Paraguay (de Chambrier and Vaucher 1994, 1999).

Note: type host; it was most likely mistaken (see notes for *Proteocephalus renaudi* on p. 50 for more details).

***Proteocephalus sophiae* de Chambrier & Rego, 1994**

*Zungaro zungaro* (Actinopterygii: Pimelodidae); freshwater; intestine; adult; Amazon River basin; Brazil, Peru (de Chambrier and Rego 1994; de Chambrier et al. 2015a).

Note: type host; it was originally reported as *Paulicea luetkeni*.

***Proteocephalus vazzolerae* Pavanelli & Takemoto, 1995**

*Leporinus friderici* (Actinopterygii: Anostomidae); freshwater; caeca, intestine; adult; Paraná River basin; Brazil (Guidelli et al. 2006, 2011).

*Leporinus lacustris* (Actinopterygii: Anostomidae); freshwater; caeca, intestine; adult; Paraná River basin; Brazil (Guidelli et al. 2006, 2011).

*Piaractus mesopotamicus* (Actinopterygii: Serrasalmidae); freshwater; intestine; adult; Paraná River basin; Brazil (Pavanelli and Takemoto 1995).

Note: type host.

***Proteocephalus vladimirae* de Chambrier & Vaucher, 1999**

*Pinirampus pirinampu* (Actinopterygii: Pimelodidae); freshwater; intestine; adult; Paraná River basin; Paraguay (de Chambrier and Vaucher 1999).

Note: type host.

***Proteocephalus* sp.**

*Franciscodoras marmoratus* (Actinopterygii: Doradidae); freshwater; intestine; adult; São Francisco River basin; Brazil (Santos and Brasil-Sato 2004).

*Gymnotus carapo* (Actinopterygii: Gymnotidae); freshwater; intestine; adult; Paraiba do Sul River basin; Brasil (Azevedo et al. 2010, 2011).

*Phractocephalus hemioliopterus* (Actinopterygii: Pimelodidae); freshwater; intestine; adult (immature specimens); Amazon River basin; Peru (de Chambrier et al. 2006a, 2015a).

Note: they named this morphotype as *Proteocephalus* sp. 1 and it is probably a new species (de Chambrier et al. 2006a).

*Pimelodus blochii* (Actinopterygii: Pimelodidae); freshwater; intestine; adult (immature specimens); Amazon River basin; Peru (de Chambrier et al. 2015a).

*Pimelodus maculatus* (Actinopterygii: Pimelodidae); freshwater; intestine; adult; Paraná River basin; Brazil (Rego et al. 1974; de Chambrier and Vaucher 1997).

Note: Rego et al. (1999a) named part of the material described by Rego et al. (1974) as *Proteocephalus magna* (Rego, Santos and Silva, 1974), but a formal description was not provided.

*Platydoras costatus* (Actinopterygii: Doradidae); freshwater; intestine; adult; Paraná River basin; Paraguay (de Chambrier and Vaucher 1994, 1999).

Note: the host species was most likely misidentified (see notes on p. 50 for more details).

*Pterodoras granulosus* (Actinopterygii: Doradidae); freshwater; intestine; adult (immature specimens); Amazon River basin; Peru (de Chambrier et al. 2006a, 2015a).

Note: they named this morphotype as *Proteocephalus* sp. 2 and it is probably a new species (de Chambrier et al. 2006a).

***Pseudocrepidobothrium chanaorum* Arredondo, Gil de Pertierra & de Chambrier, 2014**

*Pseudoplatystoma fasciatum* (Actinopterygii: Pimelodidae); freshwater; intestine; adult; Paraná River basin; Argentina (Arredondo et al. 2014).

Note: type host; it was reported as *Pseudoplatystoma reticulatum* (see the note on p. 36).

*Pseudocrepidobothrium eirasi* (Rego & de Chambrier, 1995) Rego & Ivanov, 2001*

[Syn. *Crepidobothrium eirasi* Rego & de Chambrier, 1995]

*Phractocephalus hemioliopterus* (Actinopterygii: Pimelodidae); freshwater; intestine; adult; Amazon River basin; Brazil (Rego and de Chambrier 1995; Zehnder and Mariaux 1999; Rego and Ivanov 2001; Hypša et al. 2005; Ruedi and de Chambrier 2012).

Notes: type host. Sequences of partial 18S (AY551106), complete ITS2 (AY551179), partial 28S (AJ388623) and 16S (AJ389494) (Zehnder and Mariaux 1999; Hypša et al. 2005).

***Pseudocrepidobothrium ludovici* Ruedi & de Chambrier, 2012**

*Phractocephalus hemioliopterus* (Actinopterygii: Pimelodidae); freshwater; intestine; adult; Amazon River basin; Brazil (Ruedi and de Chambrier 2012).

Note: type host.

***Pseudocrepidobothrium* sp.**

*Phractocephalus hemioliopterus* (Actinopterygii: Pimelodidae); freshwater; intestine; adult; Amazon River basin; Brazil (Zehnder and de Chambrier 2000; de Chambrier et al. 2004b).

Notes: tapeworms reported as *Crepidobothrium* sp. Sequence of complete ITS2 (AJ238838), partial 28S (AJ238833, AJ275063) and 16S (AJ238830) (Zehnder and de Chambrier 2000; de Chambrier et al. 2004b).

*Scholzia emarginata* (Diesing, 1850) de Chambrier, Rego & Gil de Pertierra, 2005*

[Syns. *Tetrabothrium emarginatum* Diesing, 1850; Tetrabothrium (Eutetrabothrium) emarginatum Diesing, 1856; *Nomimoscolex emarginatum* (Diesing, 1850) Rego, Chubb & Pavaneli, 1999; *Myzophorus pirarara* Woodland, 1935; *Nomimoscolex pirarara* (Woodland, 1935) Rego & Pavanelli, 1992; *Proteocephalus pirarara* (Woodland, 1935) de Chambrier & Vaucher, 1997].

*Phractocephalus hemioliopterus* (Actinopterygii: Pimelodidae); freshwater; intestine; adult; Amazon River basin; Brazil, Peru (Diesing 1850; Woodland 1935a; Rego 1984b; de Chambrier and Vaucher 1997; Zehnder et al. 2000; de Chambrier et al. 2005, 2006a, 2015a; Ruedi and de Chambrier 2012; Scholz et al. 2013).

Notes: type host. Sequences of partial 18S (AY551131, AY551112, KC786006), complete ITS2 (AY551170, AY551148), partial 28S (AJ388616, KC786016), partial 16S (AJ389513, KC785993) and partial *cox*1 (KC785981) (Zehnder and Mariaux 1999; Hypša et al. 2005; Scholz et al. 2013). Hypša et al. (2005) deposited two sequences because they considered *Myzophorus pirarara* and *Proteocephalus pirarara* as different species.

###### Subfamily Rudolphiellinae Woodland, 1935

*Rudolphiella lobosa* (Riggenbach, 1895) Fuhrmann, 1916*

[Syns. *Corallobothrium lobosum* Riggenbach, 1895; *Ephedrocephalus lobosum* (Riggenbach, 1895) Mola, 1906]

*Luciopimelodus pati* (Actinopterygii: Pimelodidae); freshwater; intestine; adult; Paraná River basin; Paraguay (Riggenbach 1895, 1896; Fuhrmann 1916; Rego and Gibson 1989; de Chambrier and Vaucher 1999; Gil de Pertierra and de Chambrier 2000).

Notes: type host; it was originally reported as *Pimelodus pati*, but Gil de Pertierra and de Chambrier (2000) suspected that *Megalonema platanum* is the true host, since they share the same vernacular name 'patí' and similar tapeworms were found in the latter fish host (de Chambrier and Vaucher 1999). Rego and Gibson (1989) reported hyperparasitism caused by metacestodes of proteocephalids.

*Megalonema platanum* (Actinopterygii: Pimelodidae); freshwater; intestine; adult; Paraná River basin; Paraguay (de Chambrier and Vaucher 1999; Hypša et al. 2005).

Note: de Chambrier and Vaucher (1999) reported the tapeworms as Rudolphiella cf. lobosa. Sequences of partial 18S (AY551134) and complete ITS2 (AY551173) (Hypša et al. 2005).

***Rudolphiella myoides* (Woodland, 1934) Woodland, 1935**

[Syn. *Amphilaphorchis myoides* Woodland, 1934]

*Pinirampus pirinampu* (Actinopterygii: Pimelodidae); freshwater; intestine; adult; Amazon River basin; Brazil (Woodland 1934a, 1935b; Gil de Pertierra and de Chambrier 2000).

Note: type host.

***Rudolphiella piracatinga* (Woodland, 1935) Gil de Pertierra & de Chambrier, 2000**

[Syns. *Monticellia piracatinga* Woodland, 1935; *Monticellia rugata* Rego, 1975 (*pro parte*); *Rudolphiella rugata* (Rego 1975) Rego, Chubb & Pavanelli, 1999; *Spatulifer piracatinga* (Woodland, 1935) Brooks & Deardorff, 1980]

*Calophysus macropterus* (Actinopterygii: Pimelodidae); freshwater; intestine; adult; Amazon River basin; Brazil, Peru (Woodland 1935b; Rego 1975; Mariaux 1998; Zehnder and Mariaux 1999; Gil de Pertierra and de Chambrier 2000; de Chambrier et al. 2006a, 2015a).

Notes: host originally described as *Pimelodus pati* (for details, see Gil de Pertierra and de Chambrier 2000). Rego (1975) described *Monticellia rugata*, based on a mixture of two different species, *Nomimoscolex piracatinga* (syn. of *Monticellia amazonica*) and *Monticellia piragatinga* (syn. of *Rudolphiella piracatinga*) (Gil de Pertierra and de Chambrier 2000). Sequences of partial 18S (Z98391, Z98390, Z98389), 28S (AJ388627) and 16S (AJ389504) (Mariaux 1998; Zehnder and Mariaux 1999).

Note: type host.

***Rudolphiella piranabu* (Woodland, 1934) Woodland, 1935**

[Syn. *Amphilaphorchis piranabu* Woodland, 1934]

*Pinirampus pirinampu* (Actinopterygii: Pimelodidae); freshwater; intestine; adult; Amazon and Paraná River basins; Brazil (Woodland 1934a, 1935b; Pavanelli and Machado 1991; Gil de Pertierra and de Chambrier 2000; Kohn et al. 2011).

Note: type host.

***Rudolphiella szidati* Gil de Pertierra & de Chambrier, 2000**

*Luciopimelodus pati* (Actinopterygii: Pimelodidae); freshwater; intestine; adult; Paraná River basin; Argentina (Zehnder and Mariaux 1999; Gil de Pertierra and de Chambrier 2000; Hypša et al. 2005).

Notes: type host. Sequences of partial and complete 18S (AY551135, AF286990), respectively, complete ITS2 (AY551174), partial 28S (AJ388617, AF286938) and 16S (AJ389517) (Zehnder and Mariaux 1999; Olson et al. 2001; Hypša et al. 2005).

***Rudolphiella* sp.**

*Luciopimelodus pati* (Actinopterygii: Pimelodidae); freshwater; intestine; adult (including immature specimens); Paraná River basin; Paraguay (de Chambrier and Vaucher 1999).

*Pinirampus pirinampu* (Actinopterygii: Pimelodidae); freshwater; intestine; adult (immature specimens); Amazon and Paraná River basins; Paraguay (de Chambrier and Vaucher 1999; de Chambrier et al. 2015a).

###### Subfamily Zygobothriinae Woodland, 1933

***Amphoteromorphus ninoi* Carfora, de Chambrier & Vaucher, 2003**

*Brachyplatystoma filamentosum* (Actinopterygii: Pimelodidae); freshwater; intestine; adult; Amazon River basin; Brazil (Carfora et al. 2003; Chambrier et al. 2004b).

Note: sequence of 28S (AJ388624) (Chambrier et al. 2004b).

*Brachyplatystoma vaillantii* (Actinopterygii: Pimelodidae); freshwater; intestine; adult; Amazon River basin; Brazil (Zehnder and Mariaux 1999; Carfora et al. 2003; Chambrier et al. 2004b).

Notes: type host. Tapeworms reported as *Amphoteromorphus piraeeba* by Zehnder and Mariaux (1999). Sequence of partial 28S (AJ275066) (Zehnder and Mariaux 1999).

***Amphoteromorphus ovalis* Carfora, de Chambrier & Vaucher, 2003**

Brachyplatystoma cf. filamentosum (Actinopterygii: Pimelodidae); freshwater; intestine; adult; Amazon River basin; Peru (de Chambrier et al. 2015a).

*Brachyplatystoma* sp. (Actinopterygii: Pimelodidae); freshwater; intestine; adult; Amazon River basin; Brazil (Carfora et al. 2003).

Note: type host.

***Amphoteromorphus parkamoo* Woodland, 1935**

*Zungaro zungaro* (Actinopterygii: Pimelodidae); freshwater; intestine; adult; Amazon River basin; Brazil, Peru (Woodland 1935b; Zehnder and Mariaux 1999; Zehnder et al. 2000; Carfora et al. 2003; Hypša et al. 2005; de Chambrier et al. 2006a, 2015a).

Notes: type host; it was originally reported as *Pseudopimelodus zungaro*, but also as *Paulicea luetkeni* in additional studies. Sequences of partial 18S (AY551103), complete ITS2 (AY551139), partial 28S (AJ388595) and 16S (AJ389523) (Zehnder and Mariaux 1999; Hypša et al. 2005).

*Amphoteromorphus peniculus* Diesing, 1850*

*Brachyplatystoma rousseauxii* (Actinopterygii: Pimelodidae); freshwater; intestine: adult; Amazon River basin; Brazil, Peru (Diesing 1850; Woodland 1933b; Fuhrmann 1934; Carfora et al. 2003; de Chambrier et al. 2015a, b).

Notes: type host; it was originally reported as *Bagrus goliath*, but also as *Brachyplatystoma flavicans* in additional studies. Sequence of partial 28S (KP729410) (de Chambrier et al. 2015b).

***Amphoteromorphus piraeeba* Woodland, 1934**

*Brachyplatystoma filamentosum* (Actinopterygii: Pimelodidae); freshwater; intestine; adult; Amazon River basin; Brazil (Woodland 1933a, 1934b; de Chambrier and Vaucher 1997; Zehnder and Mariaux 1999; Carfora et al. 2003; Hypša et al. 2005; de Chambrier et al. 2015b).

Notes: type host; tapeworm reported as *Amphoteromorphus peniculus* by Woodland (1933b). Sequences of partial 18S (AY551104), complete ITS2 (AY551140), partial 28S (KP729407) and 16S (AJ389510) (Zehnder and Mariaux, 1999; Hypša et al. 2005; de Chambrier et al. 2015b).

***Amphoteromorphus piriformis* Carfora, de Chambrier & Vaucher, 2003**

*Brachyplatystoma rousseauxii* (Actinopterygii: Pimelodidae); freshwater; intestine; adult (also immature specimens); Amazon River basin; Brazil, Peru (Carfora et al. 2003; de Chambrier et al. 2004b, 2006a, 2015a).

Notes: type host; it was originally reported as *Brachyplatystoma flavicans*; de Chambrier et al. (2006a) reported the tapeworms as Amphoteromorphus cf. piriformis. Sequence of partial 28S (AJ275231) (de Chambrier et al. 2004b).

*Brooksiella praeputialis* (Rego, Santos & Silva, 1974) Rego, Chubb & Pavanelli, 1999*

[Syn. *Amphoteromorphus praeputialis* Rego, dos Santos & Silva, 1974]

*Cetopsis coecutiens* (Actinopterygii: Cetopsidae); freshwater; intestine; adult; Amazon River basin; Brazil (Rego et al. 1974; de Chambrier et al. 2004a, b).

Notes: type host; de Chambrier et al. (2004a) redescribed this species. Sequence of partial 28S (AJ275229) (de Chambrier et al. 2004b).

*Cetopsis othonops* (Actinopterygii: Cetopsidae); freshwater; intestine; adult; Orinoco River basin; Venezuela (Brooks and Rasmussen 1984).

Note: host reported as *Pseudocetopsis othonops*.

*Gibsoniela mandube* (Woodland, 1935) Rego, 1984*

[Syns. *Anthobothrium mandube* Woodland, 1935; Endorchis (Pseudendorchis) mandube (Woodland, 1935) Yamaguti, 1959; *Nomimoscolex mandube* (Woodland, 1935) Brooks, 1995]

*Ageneiosus inermis* (Actinopterygii: Auchenipteridae); freshwater; intestine; adult; Amazon River basin; Brazil, Peru (Woodland 1935b; de Chambrier and Vaucher 1999; de Chambrier et al. 2015a).

Notes: type host; it was reported as *Ageneiosus brevifilis* or *Pseudoageneiosus brevifilis*.

*Ageneiosus* sp. (Actinopterygii: Auchenipteridae); freshwater; intestine; adult; Amazon River basin; Peru (de Chambrier et al. 2015a, b).

Note: sequence of partial 28S (KP729412) (de Chambrier et al. 2015b).

***Gibsoniela meursaulti* de Chambrier & Vaucher, 1999**

[Syn. *Endorchis mandube* Woodland, 1935]

*Ageneiosus inermis* (Actinopterygii: Auchenipteridae); freshwater; intestine; adult; Amazon and Paraná River basins; Argentina, Brazil, Paraguay (Woodland 1935b; Rego 1992; de Chambrier and Vaucher 1999; Zehnder and Mariaux 1999; Zehnder et al. 2000; Hypša et al. 2005; Gil de Pertierra 2009).

Notes: type host; it was reported as *Ageneiosus brevifilis* and *Pseudoageneiosus brevifilis*. Rego (1992) redescribed *Gibsoniela mandube* and considered this species as senior synonym of *Endorchis mandube*, which was previously corroborated by de Chambrier (1990); after re-examination of the type (both species) and newly collected material, de Chambrier and Vaucher (1999) assumed that they represent two distinct species of the genus *Gibsoniela*, thus they proposed *Gibsoniela meursaulti* to avoid the homonomy with the specimens tentatively identified as *Endorchis mandube*. Sequences of partial 18S (AY551109), complete ITS2 (AY551145), partial 28S (AJ388631) and 16S (AJ389497) (Zehnder and Mariaux 1999; Hypša et al. 2005).

*Ageneiosus militaris* (Actinopterygii: Auchenipteridae); freshwater; intestine; adult; Paraná River basin; Argentina (Gil de Pertierra 2009).

*Harriscolex kaparari* (Woodland, 1935) Rego, 1987*

[Syns. *Nomimoscolex karapari* Woodland, 1935; *Houssayela karapari* (Woodland, 1935) Brooks, 1995]

*Brachyplatystoma vaillantii* (Actinopterygii: Pimelodidae); freshwater; intestine; adult; Orinoco River basin; Venezuela (Brooks and Rasmussen 1984).

*Pseudoplatystoma corruscans* (Actinopterygii: Pimelodidae); freshwater; intestine; adult; Paraná and São Francisco River basins; Brazil (Rego 1990, 2002; Machado et al. 1994, 1995, 1996; Kohn et al. 2011; Ribeiro et al. 2014)

Note: records of *Harriscolex kaparari* from the Paraná River basin need verification, since *Harriscolex nathaliae* was described from the same river basin and fish host.

*Pseudoplatystoma fasciatum* (Actinopterygii: Pimelodidae); freshwater; intestine; adult; Amazon and Paraná River basins; Brazil, Peru (Pavanelli and Machado 1991; Campos et al. 2008, 2009a, b; de Chambrier et al. 2015a).

*Pseudoplatystoma tigrinum* (Actinopterygii: Pimelodidae); freshwater; intestine; adult; Amazon River basin; Brazil (Woodland 1935b; Rego 1987b, 2002; de Chambrier and Vaucher 1999; Zehnder et al. 2000; de Chambrier et al. 2004b).

Notes: type host. Sequence of partial 28S (AJ275227) and 16S (AJ275223) (de Chambrier et al. 2004b; Zehnder et al. 2000).

***Harriscolex nathaliae* Gil de Pertierra & de Chambrier, 2013**

*Pseudoplatystoma corruscans* (Actinopterygii: Pimelodidae); freshwater; intestine; adult; Paraná River basin; Argentina, Paraguay (de Chambrier and Vaucher 1999; Gil de Pertierra and de Chambrier 2013).

Note: type host; de Chambrier and Vaucher (1999) reported the tapeworms as Harriscolex cf. kaparari; they observed morphological differences between the type material from the Amazon River and their specimens, which were posteriorly described as *Harriscolex nathaliae*.

***Harriscolex piramutab* (Woodland, 1933) de Chambrier, Kuchta & Scholz, 2015** [Syns. *Anthobothrium piramutab* Woodland, 1933; *Proteocephalus piramutab* (Woodland, 1933) Rego, 1984]

*Brachyplatystoma vaillantii* (Actinopterygii: Pimelodidae); freshwater; intestine; adult; Amazon River basin; Brazil, Peru (Woodland 1933c; Rego 1984a, 1987b; de Chambrier et al. 2006a, 2015a).

Note: type host.

*Houssayela sudobim* (Woodland, 1935) Rego, 1987*

[Syns. *Myzophorus sudobim* Woodland, 1935; *Nomimoscolex woodlandi* Freze, 1965 *nec* *Nomimoscolex woodlandi* Rego & Pavanelli, 1992]

*Pseudoplatystoma fasciatum* (Actinopterygii: Pimelodidae); freshwater; intestine; adult; Amazon River basin; Brazil, Peru (Woodland 1935a; Rego 1987b, 2002; de Chambrier and Scholz 2005; de Chambrier et al. 2006a, 2015a, b).

Notes: type host. Rego (1999) presented a scanning electron micrograph of the tapeworm scolex, but he did not mention the host and locality; in fact, it corresponds to the scolex of *Choanoscolex abscisus* – see de Chambrier et al. (2006a).

de Chambrier et al. (2015b) erroneously reported *Sorubimichthys planiceps* as the host in their Table I. Sequence of partial 28S (KP729404) (de Chambrier et al. 2015b).

***Nomimoscolex admonticellia* (Woodland, 1934) Rego & Pavanelli, 1992**

[Syns. *Myzophorus admonticellia* Woodland, 1934 (*pro parte*); *Paramonticellia admonticellia* (Woodland, 1934) Brooks, 1995; *Myzophorus schaefferi* Pavanelli and Machado, 1991 (*nomen nudum*)]

*Pinirampus pirinampu* (Actinopterygii: Pimelodidae); freshwater; intestine; adult; Amazon and Paraná River basins; Brazil, Peru (Woodland 1934a; Pavanelli and Machado 1991; Rego and Pavanelli 1992; Zehnder et al. 2000; Hypša et al. 2005; de Chambrier et al. 2015a).

Notes: see p. 40 in the section of *Monticellia ventrei* for notes on the type host. Rego and Pavanelli (1992) redescribed this species based on a mixture of *Nomimoscolex admonticellia* and *Monticellia ventrei* (see de Chambrier and Vaucher 1999). Sequences of partial 18S (AY551113), complete ITS2 (AY551149), partial 28S (AJ388628) and 16S (AJ389512) (Zehnder and Mariaux 1999; Hypša et al. 2005).

***Nomimoscolex alovarius* Brooks & Deardorff, 1980**

*Pimelodus blochii* (Actinopterygii: Pimelodidae); freshwater; intestine; adult; Magdalena River basin; Colombia (Brooks and Deardorff 1980).

Note: type host; it was reported as *Pimelodus clarias*.

***Nomimoscolex chubbi* (Pavanelli & Takemoto, 1995) de Chambrier & Vaucher, 1997**

[Syn. *Proteocephalus chubbi* Pavanelli & Takemoto, 1995]

*Gymnotus carapo* (Actinopterygii: Gymnotidae); freshwater; intestine; adult; Paraná River basin; Argentina, Brazil, Paraguay (Pavanelli and Takemoto 1995; de Chambrier and Vaucher 1997, 1999; Zehnder and Mariaux 1999; Zehnder et al. 2000; Gil de Pertierra 2003, 2005).

Notes: type host. Sequences of partial 28S (AJ388625) and 16S (AJ389524) (Zehnder and Mariaux 1999).

*Gymnotus* sp. (Actinopterygii: Gymnotidae); freshwater; intestine; adult; Paraná River basin; Brazil (Isaac et al. 2004).

***Nomimoscolex dechambrieri* Gil de Pertierra, 2003**

*Gymnotus carapo* (Actinopterygii: Gymnotidae); freshwater; intestine; adult; Paraná River basin; Argentina (Gil de Pertierra 2003).

Note: type host.

***Nomimoscolex dorad* (Woodland, 1935) Freze, 1965**

[Syn. *Myzophorus dorad* Woodland, 1935]

*Brachyplatystoma rousseauxii* (Actinopterygii: Pimelodidae); freshwater; intestine; adult; Amazon River basin; Brazil (Woodland 1935a; Freze 1965; Zehnder and Mariaux 1999; Zehnder et al. 2000; Hypša et al. 2005).

Notes: type host; it was reported as *Brachyplatystoma flavicans* in some studies. Sequences of partial 18S (AY551114), complete ITS2 (AY551150), partial 28S (AJ388613) and 16S (AJ389498) (Zehnder and Mariaux 1999; Hypša et al. 2005).

***Nomimoscolex guillermoi* Gil de Pertierra, 2003**

*Gymnotus carapo* (Actinopterygii: Gymnotidae); freshwater; intestine; adult; Paraná River basin; Argentina (Gil de Pertierra 2003).

Note: type host.

***Nomimoscolex lenha* (Woodland, 1933) Woodland, 1935**

[Syns. *Proteocephalus lenha* Woodland, 1933; *Paramonticellia lenha* (Woodland, 1933) Brooks, 1995]

*Sorubimichthys planiceps* (Actinopterygii: Pimelodidae); freshwater; intestine; adult; Amazon River basin; Brazil, Peru (Woodland 1933a, 1935b; de Chambrier and Vaucher 1997; Zehnder and Mariaux 1999; Zehnder et al. 2000; Hypša et al. 2005; de Chambrier and Scholz 2008; de Chambrier et al. 2015a).

Notes: type host; it was originally reported as *Platystomatichthys sturio*. Sequences of partial 18S (AY551115), complete ITS2 (AY551151), partial 28S (AJ388611) and 16S (AJ389499) (Zehnder and Mariaux 1999; Hypša et al. 2005).

***Nomimoscolex lopesi* Rego, 1989**

[Syn. *Paramonticellia lopesi* (Rego, 1989) Brooks, 1995]

*Pseudoplatystoma fasciatum* (Actinopterygii: Pimelodidae); freshwater; intestine; adult; Amazon and Paraná River basins; Argentina, Brazil, Paraguay, Peru (Rego 1989, 2002; de Chambrier and Vaucher 1999; Zehnder et al. 2000; Gil de Pertierra 2005; Hypša et al. 2005; de Chambrier et al. 2006a, 2015a).

Note: type host. Sequences of partial 18S (AY551116), complete ITS2 (AY551152), partial 28S (AJ388618) and 16S (AJ389521) (Zehnder and Mariaux 1999; Hypša et al. 2005).

***Nomimoscolex matogrossensis* Rego & Pavanelli, 1990**

*Hoplerythrinus unitaeniatus* (Actinopterygii: Erythrinidae); freshwater; body

cavity, intestine, stomach; metacestode; Amazon River basin; Brazil (Alcântara and Tavares-Dias 2015; Gonçalves et al. 2016).

Note: the authors did not provide any molecular data on these larvae; therefore, this report needs verification.

*Hoplias malabaricus* (Actinopterygii: Erythrinidae); freshwater; intestine; adult

and metacestode; Amazon and Paraná River basin; Brazil, Paraguay (Rego and Pavanelli 1990; de Chambrier et al. 1996; de Chambrier and Vaucher 1999; Zehnder et al. 2000; Alcântara and Tavares-Dias 2015; Gonçalves et al. 2016).

Notes: type host. Sequences of partial 18S (Z98387, Z98386, Z98388), 28S (AJ388614) and 16S (AJ389500) (Mariaux 1998; Zehnder and Mariaux 1999).

***Nomimoscolex microacetabula* Gil de Pertierra, 1995**

*Pimelodus albicans* (Actinopterygii: Pimelodidae); freshwater; intestine; adult; Paraná River basin; Argentina (Gil de Pertierra 1995).

*Pimelodus maculatus* (Actinopterygii: Pimelodidae); freshwater; intestine; adult; Paraná River basin; Argentina (Gil de Pertierra 1995).

Note: type host.

*Pimelodus ornatus* (Actinopterygii: Pimelodidae); freshwater; intestine; adult; Paraná River basin; Paraguay (de Chambrier and Vaucher 1999).

Note: they reported this species as Nomimoscolex cf. microacetabula.

***Nomimoscolex pertierrae* de Chambrier, Takemoto & Pavanelli, 2006**

*Pseudoplatystoma corruscans* (Actinopterygii: Pimelodidae); freshwater; intestine; adult; Paraná river basin; Brazil (Pavanelli and Rego 1992; de Chambrier et al. 2006b; Ribeiro and Takemoto 2014; Ribeiro et al. 2014).

Note: type host. Pavanelli and Rego (1992) reported this species as *Nomimoscolex sudobim*, according to de Chambrier et al. (2006b).

*Nomimoscolex piraeeba* Woodland, 1934*

*Brachyplatystoma capapretum* (Actinopterygii: Pimelodidae); freshwater; intestine; adult; Amazon River basin; Brazil (Woodland 1934b; de Chambrier and Vaucher 1997; Rego 1991; Rego and Schaeffer 1999; Zehnder and Mariaux 1999; Zehnder et al. 2000; Olson et al. 2001; Hypša et al. 2005).

Notes: type host; it was identified as *Brachyplatystoma filamentosum*. *Nomimoscolex piraeeba*, *Nomimoscolex dorad* and *Nomimoscolex suspectus* were defined as *Nomimoscolex* (*sensu stricto*) by Zehnder et al. (2000). Sequences of complete 18S (AF286988) and ITS2 (AY551153), partial (28S AJ388608, AF286936) and 16S (AJ389502) (Zehnder and Mariaux 1999; Olson et al. 2001; Hypša et al. 2005).

*Brachyplatystoma rousseauxii* (Actinopterygii: Pimelodidae); freshwater; intestine; adult; Amazon River basin; Brazil (Rego 1991; de Chambrier and Vaucher 1997; Rego and Schaeffer 1999).

Note: host reported as *Brachyplatystoma flavicans*.

***Nomimoscolex semenasae* Gil de Pertierra, 2002**

*Olivaichthys viedmensis* (Actinopterygii: Diplomystidae); freshwater; intestine; adult; Moreno and Nahuel Huapi Lakes; Argentina (Gil de Pertierra 2002a; Rauque et al. 2003).

Note: type host; it was originally reported as *Diplomystes viedmensis*.

***Nomimoscolex sudobim* Woodland, 1935**

[Syn. *Paramonticellia sudobim* (Woodland, 1935) Brooks, 1995]

*Pseudoplatystoma corruscans* (Actinopterygii: Pimelodidae); freshwater; intestine; adult; Paraná River basin; Brazil (Pavanelli and Machado 1991; Machado et al. 1994, 1995, 1996; Rego 2002).

Note: records of this species from the Paraná River basin need verification, because *Nomimoscolex pertierrae* was described from the same river basin and host.

*Pseudoplatystoma fasciatum* (Actinopterygii: Pimelodidae); freshwater; intestine; adult; Amazon and Paraná River basins; Brazil, Peru (Woodland 1935a; Zehnder and Mariaux 1999; Zehnder et al. 2000; Rego 2002; Santos et al. 2003; Hypša et al. 2005; Ceccarrelli et al. 2006; de Chambrier et al. 2006b, 2015a; Campos et al. 2008, 2009a, b; Jerônimo et al. 2013).

Notes: type host. Host reported as *Pseudoplatystoma reticulatum* by Jerônimo et al. (2013) (see the note on p. 36). Sequences of partial 18S (AY551117), complete ITS2 (AY551154), partial 28S (AJ388597) and 16S (AJ389496) (Zehnder and Mariaux 1999; Hypša et al. 2005).

*Pseudoplatystoma tigrinum* (Actinopterygii: Pimelodidae); freshwater; intestine; adult; Amazon River basin; Peru (de Chambrier et al. 2006a).

Note: they reported this species as Nomimoscolex cf. sudobim.

***Nomimoscolex suspectus* Zehnder, de Chambrier, Vaucher & Mariaux, 2000**

*Brachyplatystoma filamentosum* (Actinopterygii: Pimelodidae); freshwater; intestine; adult; Amazon River basin; Brazil (Zehnder et al. 2000).

Notes: type host. Sequences of partial 28S (AJ275067) (Zehnder et al. 2000).

Brachyplatystoma cf. filamentosum (Actinopterygii: Pimelodidae); freshwater; intestine; adult; Amazon River basin; Peru (de Chambrier et al. 2015a).

*Brachyplatystoma rousseauxii* (Actinopterygii: Pimelodidae); freshwater; intestine; adult; Amazon River basin; Brazil (Zehnder et al. 2000).

Notes: host reported as *Brachyplatystoma flavicans*. Sequence of partial 28S (AJ275068) (Zehnder et al. 2000).

*Brachyplatystoma vaillantii* (Actinopterygii: Pimelodidae); freshwater; intestine; adult; Amazon River basin; Brazil (Zehnder and Mariaux 1999; Zehnder et al. 2000).

Notes: tapeworms reported as *Nomimoscolex* sp. by Zehnder and Mariaux (1999). Sequences of partial 18S (AY551118), complete ITS2 (AY551155), partial 28S (AJ388602) and 16S (AJ389519) (Zehnder and Mariaux 1999; Hypša et al. 2005).

***Nomimoscolex* sp.**

*Brachyplatystoma rousseauxii* (Actinopterygii: Pimelodidae); freshwater; intestine; adult; Amazon River basin (estuary, Marajó Island); Brazil (Rocha et al. 2016).

*Luciopimelodus pati* (Actinopterygii: Pimelodidae); freshwater; intestine; adult; Paraná River basin; Paraguay (de Chambrier and Vaucher 1999).

*Pimelodus maculatus* (Actinopterygii: Pimelodidae); freshwater; intestine; adult; Paraíba do Sul, Paraná and São Francisco River basins; Brazil (Brasil-Sato 2003; Santos et al. 2007; Albuquerque et al. 2008; Azevedo et al. 2010, 2011).

*Pimelodus ornatus* (Actinopterygii: Pimelodidae); freshwater; intestine; adult; Amazon River basin; Peru (de Chambrier et al. 2015a).

*Pseudoplatystoma corruscans* (Actinopterygii: Pimelodidae); freshwater; intestine; adult; São Francisco River basin; Brazil (Brasil-Sato 2003).

*Pseudoplatystoma fasciatum* (Actinopterygii: Pimelodidae); freshwater; intestine; adult; Amazon River Basin; Brazil (Lopes et al. 2009).

Note: host reported as *Pseudoplatystoma punctifer* (see the note on p. 32).

*Travassiella jandia* (Woodland, 1934) de Chambrier, Scholz & Kuchta, 2014*

[Syns. *Proteocephalus jandia* Woodland, 1934; *Travassiella avitellina* Rego & Pavanellli, 1987]

*Rhamdia quelen* (Actinopterygii: Heptapteridae); freshwater; intestine; adult; Chis-Chis lagoon (Buenos Aires); Argentina (Gil de Pertierra and Ostrowski de Núñez 1990).

Note: host reported as *Rhamdia sapo*.

*Zungaro jahu* (Actinopterygii: Pimelodidae); freshwater; intestine; adult; Paraná River basin; Argentina, Brazil, Paraguay (Rego and Pavanelli 1987; Rego and Gibson 1989; Pavanelli and Machado 1991; Takemoto and Pavanelli 1994; de Chambrier and Vaucher 1999; de Chambrier and Gil de Pertierra 2002; Kohn et al. 2011; de Chambrier et al. 2014, 2015b).

Notes: host also reported as *Zunguro zungaro* or *Paulicea luetkeni*. Rego and Gibson (1989) and Rego and Pavanelli (1987) reported hyperparasitism caused by metacestodes of proteocephalids. Sequence of 28S (KP729400) (de Chambrier et al. 2015b).

*Zungaro zungaro* (Actinopterygii: Pimelodidae); freshwater; intestine; adult; Amazon River basin; Brazil, Peru (Woodland 1934c; de Chambrier and Gil de Pertierra 2002; de Chambrier et al. 2006a, 2014, 2015a).

Note: type host; it was originally reported as *Rhamdia* sp.

Unidentified fish (Actinopterygii); freshwater; intestine; adult; Amazon River basin; Brazil (Rego et al. 1974).

*Zygobothrium megacephalum* Diesing, 1850*

*Phractocephalus hemioliopterus* (Actinopterygii: Pimelodidae); freshwater; intestine; adult; Amazon River basin; Brazil, Peru (Diesing 1850, 1855; Woodland 1933b; Fuhrmann 1934; Rego 1984b; Zehnder and Mariaux 1999; Olson et al. 2001; Hypša et al. 2005; de Chambrier et al. 2006a, 2015a; Ruedi and de Chambrier 2012).

Notes: type host. Sequences of complete 18S (AF286991) and ITS2 (AY551177), partial 28S (AF286939, AJ388621) and 16S (AJ389508) (Zehnder and Mariaux 1999; Olson et al. 2001; Hypša et al. 2005).

**Unidentified proteocephalids**

*Aequidens tetramerus* (Actinopterygii: Cichlidae); freshwater; body cavity; metacestode; Amazon River basin; Brazil (Bittencourt et al. 2014).

*Aphyocharax anisitsi* (Actinopterygii: Characidae); freshwater; site of infection not given; metacestode; Paraná River basin; Brazil (Takemoto et al. 2009).

*Astronotus ocellatus* (Actinopterygii: Cichlidae); freshwater; body cavity; metacestode; fish farms in Northeast (Ceará, Pernambuco, Piauí and Rio Grande do Norte States); Brazil (Békési et al. 1992).

*Astyanax altiparanae* (Actinopterygii: Characidae); freshwater; body cavity; metacestode; Rio das Pedras Farm (lakes); Brazil (Azevedo et al. 2007).

*Auchenipterus nigripinnis* (Actinopterygii: Auchenipteridae); freshwater; intestine; adult; Paraná River basin; Brazil (Rego and Vicente 1988).

*Brachyplatystoma filamentosum* (Actinopterygii: Pimelodidae); freshwater; intestine; adult; Amazon River basin; Brazil (Woodland 1935c).

*Brycon cephalus* (Actinopterygii: Bryconidae); freshwater; surface of intestine and pyloric caeca; metacestode; Amazon River basin; Brazil (Andrade et al. 2001).

*Cichla ocellaris* (Actinopterygii: Cichlidae); freshwater; body cavity; metacestode; fish farms in Northeastern Brazil (Ceará, Pernambuco, Piauí and Rio Grande do Norte States); Brazil (Békési et al. 1992).

*Cichlasoma amazonarum* (Actinopterygii: Cichlidae); freshwater; intestine; adult; Amazon River basin; Peru (de Chambrier et al. 2015a).

Note: these tapeworms belong to a new species and genus that will be described in a forthcoming paper.

*Colossoma macropomum* (Actinopterygii: Serrasalmidae); freshwater; body cavity; metacestode; fish farms in Northeastern Brazil (Ceará, Pernambuco, Piauí and Rio Grande do Norte States); Brazil (Békési et al. 1992).

*Corydoras atropersonatus* (Actinopterygii: Callichthyidae); freshwater; mesentery; metacestode; Amazon River basin; Peru (de Chambrier et al. 2006a).

*Corydoras reticulatus* (Actinopterygii: Callichthyidae); freshwater; mesentery; metacestode; Amazon River basin; Peru (de Chambrier et al. 2006a).

*Corydoras sychri* (Actinopterygii: Callichthyidae); freshwater; mesentery; metacestode; Amazon River basin; Peru (de Chambrier et al. 2006a).

*Crenicichla lepidota* (Actinopterygii: Cichlidae); freshwater; intestine; adult (immature specimens); Paraná River Basin; Paraguay (de Chambrier and Vaucher 1999).

*Cynoscion striatus* (Actinopterygii: Sciaenidae); marine; intestine; metacestode; WTSA; Brazil (Rego et al. 1974).

Note: certainly not a larva of the Proteocephalidae.

*Cyprinus carpio* (Actinopterygii: Cyprinidae); freshwater; body cavity; metacestode; fish farms in Northeastern Brazil (Ceará, Pernambuco, Piauí and Rio Grande do Norte states); Brazil (Békési et al. 1992).

Note: introduced fish host (Froese and Pauly 2016).

*Galeocharax knerii* (Actinopterygii: Characidae); freshwater; intestine; metacestode; Paraná River basin; Brazil (Takemoto et al. 2009).

*Geophagus brasiliensis* (Actinopterygii: Cichlidae); freshwater; body cavity, gallbladder; metacestode; Paraná River basin; Brazil (Bellay et al. 2012).

*Geophagus proximus* (Actinopterygii: Cichlidae); freshwater; body cavity; metacestode; Paraná River basin; Brazil (Zago et al. 2013).

*Hemisorubim platyrhynchos* (Actinopterygii: Pimelodidae); freshwater; mesentery; metacestode; Amazon River basin; Peru (de Chambrier et al. 2006a).

*Hypophthalmichthys nobilis* (Actinopterygii: Cyprinidae); freshwater; body cavity; metacestode; fish farms in Northeastern Brazil (Ceará, Pernambuco, Piauí and Rio Grande do Norte States); Brazil (Békési et al. 1992).

Note: introduced fish host (Froese and Pauly 2016).

Hypostomus cf. ternetzi (Actinopterygii: Loricariidae); freshwater; intestine; adult (immature specimens); Paraná River Basin; Paraguay (de Chambrier and Vaucher 1999).

*Iheringichthys labrosus* (Actinopterygii: Pimelodidae); freshwater; intestine; adult; Paraná River basin; Brazil (Moreira et al. 2005).

*Laetacara curviceps* (Actinopterygii: Cichlidae); freshwater; body cavity; metacestode; Amazon River basin; Brazil (Bittencourt et al. 2014).

*Leporellus vittatus* (Actinopterygii: Anostomidae); freshwater; site of infection not given; metacestode; Paraná River basin; Brazil (Takemoto et al. 2009).

Leporinus aff. friderici (Actinopterygii: Anostomidae); freshwater; intestine; adult; Paraná River Basin; Paraguay (de Chambrier and Vaucher 1999).

*Loricariichthys platymetopon* (Actinopterygii: Loricariidae); freshwater; body cavity, internal organs; metacestode; Paraná River basin; Brazil (Schäeffer et al. 1992).

*Megalonema platanum* (Actinopterygii: Pimelodidae); freshwater; intestine; adult (immature specimens); Paraná River Basin; Paraguay (de Chambrier and Vaucher 1999).

*Oxydoras kneri* (Actinopterygii: Doradidae); freshwater; intestine; adult; Paraná River basin; Brazil (Rego and Vicente 1988).

*Oreochromis* sp. (Actinopterygii: Cichlidae); freshwater; body cavity; metacestode; fish farms in Northeast (Ceará, Pernambuco, Piauí and Rio Grande do Norte States); Brazil (Békési et al. 1992).

Note: introduced fish host (Froese and Pauly 2016).

*Phractocephalus hemioliopterus* (Actinopterygii: Pimelodidae); freshwater; intestine; adult; Amazon River basin; Brazil (Rego 1984b).

Note: the author reported the presence of operculate eggs released from a contracted proglottid, but this unique report needs confirmation.

*Piaractus brachypomus* (Actinopterygii: Serrasalmidae); freshwater; body cavity; metacestode; fish farms in northeastern Brazil (Ceará, Pernambuco, Piauí and Rio Grande do Norte States); Brazil (Békési et al. 1992).

Note: host reported as *Colossoma brachipomum*.

*Pimelodella gracilis* (Actinopterygii: Heptapteridae); freshwater; mesentery; metacestode; Amazon River basin; Peru (de Chambrier et al. 2006a).

*Pimelodus maculatus* (Actinopterygii: Pimelodidae); freshwater; intestine; adult (immature specimens), metacestode; Paraná and São Francisco River basins; Brazil, Paraguay (de Chambrier and Vaucher 1999; Brasil-Sato 2003).

*Pimelodus pohli* (Actinopterygii: Pimelodidae); freshwater; intestine; metacestode; São Francisco River basin; Brazil (Sabas and Brasil-Sato 2014).

*Pimelodus* sp. (Actinopterygii: Pimelodidae); freshwater; intestine; adult; Paraná River basin; Brazil (Rego and Vicente 1988).

*Pinirampus pirinampu* (Actinopterygii: Pimelodidae); freshwater; intestine; adult; Paraná River basin; Brazil (Rego and Vicente 1988).

*Prochilodus brevis* (Actinopterygii: Prochilodontidae); freshwater; body cavity; metacestode; fish farms in Northeast (Ceará, Pernambuco, Piauí and Rio Grande do Norte States); Brazil (Békési et al. 1992).

Note: host reported as *Prochilodus cearensis*.

*Prochilodus lineatus* (Actinopterygii: Prochilodontidae); freshwater; body cavity; metacestode; Paraná River basin; Brazil (Lizama et al. 2005, 2006).

*Psellogrammus kennedyi* (Actinopterygii: Characidae); freshwater; site of infection not given; metacestode; Paraná River basin; Brazil (Takemoto et al. 2009).

*Pseudoplatystoma corruscans* (Actinopterygii: Pimelodidae); freshwater; intestine; adult; Paraná River basin; Brazil (Rego and Vicente 1988).

*Pseudoplatystoma fasciatum* (Actinopterygii: Pimelodidae); freshwater; intestine; adult; Paraná River basin; Brazil (Rego and Vicente 1988).

*Rhamdia quelen* (Actinopterygii: Heptapteridae); freshwater; intestine; adult; Guaíba River estuary (Fortes and Hoffman 1987; Rego and Gibson 1989).

Note: host reported as *Rhamdia sapo*; in both studies, the authors reported hyperparasitism caused by metacestodes of proteocephalids.

*Sardinella* sp. (Actinopterygii: Clupeidae); marine; intestine; metacestode; WTSA; Brazil (Feijó et al. 1979).

Note: certainly not a larva of the Proteocephalidae.

*Satanoperca pappaterra* (Actinopterygii: Cichlidae); freshwater; intestine; metacestode; Paraná River basin; Brazil (Yamada et al. 2007).

*Trinectes maculatus* (Actinopterygii: Achiridae); brackish, freshwater; mesentery; metacestode; Amazon River basin; Peru (de Chambrier et al. 2006a).

Hybrid fish host (Actinopterygii: Serrasalmidae); freshwater; intestine; metacestode; Amazon River basin; Brazil (Silva et al. 2013).

Note: the host is a hybrid of *Colossoma macropomum* and *Piaractus mesopotamicus*.

***Species inquirendae***

***Acanthobothroides peruensis* López, 1994**

*Dasyatis dipterura* (Elasmobranchii: Dasyatidae); marine; spiral valve; adult; WTSP; Peru (López 1994).

Notes: type host; it was reported as *Dasyatis brevis*. Only *Acanthobothroides thorsoni* and *Acanthobothroides pacificum* Marques, Brooks & Ureña, 1996 are considered valid species in the genus (Marques et al. 1996).

***Monticellia diesingii* (Monticelli, 1891) La Rue, 1911**

[Syns. *Taenia diesingii* Monticelli, 1891; *Tetracotylus diesingii* Monticelli, 1891; *Ichthyotaenia diesingii* (Monticelli, 1891) Riggenbach, 1896]

‘*Silurus dorgado*’ (unknown fish host); freshwater; intestine; adult; unknown

specific locality (Monticelli 1891; Riggenbach 1896b; La Rue 1911, 1914).

***Monticellia macrocotylea* (Monticelli, 1891) La Rue, 1911**

[Syns. *Taenia macrocotylea* Monticelli, 1891; *Tetracotylus macrocotylea* Monticelli, 1891; *Ichthyotaenia macrocotylea* (Monticelli, 1891) Riggenbach, 1896]

‘*Silurus megacephalus*’ (unknown fish host); freshwater; intestine; adult; unknown locality (Monticelli 1891; Riggenbach 1896b; La Rue 1911, 1914).

***Nomimoscolex arandasregoi* Fortes, 1981**

*Genidens barbus* (Actinopterygii: Ariidae); anadromous; intestine; adult; Guaíba River estuary, WTSA; Brazil (Fortes 1981; Fortes and Hoffmann 1995; Tavares and Luque 2004; Tavares and Luque 2008).

Note: host reported under four different names, *Tachysurus agassizii*, *Tachysurus upsulonophorus*, *Tachysurus barbus* and *Netuma barba*.

*Genidens genidens* (Actinopterygii: Ariidae); anadromous; intestine; adult; Guaíba River estuary; Brazil (Fortes 1981).

Note: Fortes (1981) did not designate the type host.

*Genidens* sp. (Actinopterygii: Ariidae); anadromous; intestine; adult; Guaíba River estuary; Brazil (Rego and Gibson 1989).

Note: Rego and Gibson (1989) reported hyperparasitism caused by metacestodes of proteocephalids.

***Platybothrium parvum* Linton, 1901**

*Sphyrna zygaena* (Elasmobranchii: Sphyrnidae); marine; spiral valve; adult; WTSP; Peru (Rivera and Sarmiento 1990).

Note: for details on the taxonomic status of this species, see Healy (2003).

#### Order Phyllobothriidea Caira, Jensen, Waeschenbach, Olson & Littlewood, 2014

##### Family Phyllobothriidae Braun, 1900

***Crossobothrium antonioi* Ivanov, 2009**

*Notorynchus cepedianus* (Elasmobranchii: Hexanchidae); marine; spiral valve; adult; WTSA; Argentina (Ivanov 2009).

Note: type host.

***Crossobothrium dohrni* (Örley, 1885) Ruhnke, 1996**

[Syns. *Orygmatobothrium dohrni* Örley, 1885; *Phyllobothrium dohrni* (Örley, 1885) Zschokke, 1888]

*Hexanchus griseus* (Elasmobranchii: Hexanchidae); marine; spiral valve; adult; WTSP; Chile (Carvajal 1974).

Note: tapeworms reported as *Phyllobothrium dorhni*.

*Crossobothrium laciniatum* Linton, 1889*

[Syn. *Phyllobothrium laciniatum* (Linton, 1889) Southwell, 1925]

*Hexanchus griseus* (Elasmobranchii: Hexanchidae); marine; spiral valve; adult; Magellanic; Chile (Caira et al. 2014).

Notes: position of the species within Phyllobothriidae, based on molecular data, is unclear. Sequences of partial 18S (KF685824) and 28S (KF685883) (Caira et al. 2014).

***Crossobothrium pequeae* Ivanov, 2009**

*Notorynchus cepedianus* (Elasmobranchii: Hexanchidae); marine; spiral valve; adult; WTSA; Argentina (Ivanov 2009).

Note: type host.

*Nandocestus guariticus* (Marques, Brooks & Lasso, 2001) Reyda, 2008*

[Syn. *Anindobothrium guariticus* Marques, Brooks & Lasso, 2001]

*Paratrygon aiereba* (Elasmobranchii: Potamotrygonidae); freshwater; spiral valve; Amazon and Orinoco River basins; Peru, Venezuela (Marques et al. 2001; Reyda and Olson 2003; Reyda 2008; Caira et al. 2014).

Notes: type host. Reyda and Olson (2003) and Reyda (2008) reported hyperparasitism by larval stages of proteocephalids. Sequences of partial 28S (KF685888) and 18S (KF685817) (Caira et al. 2014).

Potamotrygon cf. falkneri (Elasmobranchii: Potamotrygonidae); freshwater; spiral valve; adult (immature); Amazon River basin; Peru (Reyda 2008).

Note: host reported as Potamotrygon cf. castexi and it may represent an undescribed species of *Potamotrygon* (see Reyda 2008).

***Orygmatobothrium juani* Ivanov, 2008**

*Mustelus fasciatus* (Elasmobranchii: Carcharhinidae); marine; spiral valve; adult; WTSA; Argentina (Ivanov 2008).

Note: type host.

*Orygmatobothrium musteli* (van Beneden, 1849) Diesing, 1863*

[Syns. *Anthobothrium musteli* van Beneden, 1850 (*pro parte*); *Orygmatobothrium versatile* (Diesing, 1854) Diesing, 1863; *Tetrabothrium versatile* Diesing, 1854]

*Mustelus mento* (Elasmobranchii: Triakidae); marine; spiral valve; adult; WTSP; Chile (Carvajal 1974; Whittaker and Carvajal 1980).

*Mustelus whitneyi* (Elasmobranchii: Triakidae); marine; spiral valve; adult; WTSP; Peru (Escalante and Carvajal 1981).

***Orygmatobothrium schmittii* Suriano & Labriola, 2001**

*Mustelus schmitti* (Elasmobranchii: Triakidae); marine; spiral valve; adult; WTSA; Argentina (Ostrowski de Núñez 1973; Suriano and Labriola 2001b; Alarcos et al. 2006; Ivanov 2008).

Notes: type host. Ivanov (2008) redescribed this species and re-assigned the specimens described as *Orygmatobothrium velamentum* Yoshida, 1917 by Ostrowski de Núñez (1973) to *Orygmatobothrium schmitti*.

***Orygmatobothrium* sp.**

*Mustelus mento* (Elasmobranchii: Triakidae); marine; spiral valve; adult; WTSP; Peru (Tresierra et al. 1986).

***Paraorygmatobothrium angustum* (Linton, 1889) Ruhnke, 2011**

[Syns. *Orygmatobothrium angustum* Linton, 1889; *Crossobothrium angustum* (Linton, 1889) Linton, 1901; *Phyllobothrium angustum* (Linton, 1889) Euzet, 1952; *Scyphophyllidium angustum* (Linton, 1889) Riser, 1955]

*Alopias vulpinus* (Elasmobranchii: Alopiidae); marine; spiral valve; adult; WTSP; Chile (Carvajal 1974).

Note: tapeworms reported as *Crossobothrium angustum*.

*Prionace glauca* (Elasmobranchii: Carcharhinidae); marine; spiral valve; adult; WTSP; Chile, Peru (Carvajal 1974; Escalante 1986).

Note: tapeworms reported as *Crossobothrium angustum*.

***Paraorygmatobothrium filiforme* (Yamaguti, 1952) Ruhnke, 1996**

[Syns. *Phyllobothrium filiforme* Yamaguti, 1952; *Crossobothrium filiforme* (Yamaguti, 1952) Williams, 1968]

*Carcharhinus longimanus* (Elasmobranchii: Carcharhinidae); marine; spiral valve; adult; TSA; Brazil (Rego 1977).

Note: tapeworms reported as *Phyllobothrium filiforme*.

*Paraorygmatobothrium prionacis* (Yamaguti, 1934) Ruhnke, 1994*

[Syns. *Phyllobothrium prionacis* Yamaguti, 1934; *Crossobothrium prionacis* (Yamaguti, 1934) Williams, 1968; *Anthobothrium minutum* Guiart, 1935]

*Prionace glauca* (Elasmobranchii: Carcharhinidae); marine; spiral valve; adult; TSA; Brazil (Rego and Mayer 1976).

Notes: type host. Tapeworms reported as *Prionace prionacis*.

***Paraorygmatobothrium triacis* (Yamaguti, 1952) Ruhnke, 1996**

[Syns. *Phyllobothrium triacis* Yamaguti, 1952; *Crossobothrium triacis* (Yamaguti, 1952) Euzet, 1959]

*Mustelus mento* (Elasmobranchii: Triakidae); marine; spiral valve; adult; WTSP; Chile (Carvajal 1974).

Note: tapeworms reported as *Crossobothrium triacis*.

*Mustelus whitneyi* (Elasmobranchii: Triakidae); marine; spiral valve; adult; WTSP; Peru (Escalante and Carvajal 1981).

Note: tapeworms reported as *Crossobothrium triacis*.

*Phyllobothrium lactuca* van Beneden, 1850*

*Dipturus trachyderma* (Elasmobranchii: Rajidae); marine; spiral valve; adult; WTSP; Chile (Leible et al. 1990).

Notes: tapeworms reported as Phyllobothrium cf. lactuca and the host as *Raja trachyderma*. The identification of this cestode is most likely erroneous, since sharks are the definitive hosts for species of the genus *Phyllobothrium* van Beneden, 1849 (see Ruhnke 2011).

*Mustelus mento* (Elasmobranchii: Triakidae); marine; spiral valve; adult; WTSP; Chile, Peru (Carvajal 1974; Escalante and Carvajal 1981; Ruhnke and Workman 2013; Caira et al. 2014).

Notes: tapeworms reported as Phyllobothrium cf. lactuca by Ruhnke and Workman (2013) and Caira et al. (2014). Sequences of partial 18S (KF685770) and 28S (KF685845, KC505628) (Ruhnke and Workman 2013; Caira et al. 2014).

***Phyllobothrium* sp.**

*Dipturus flavirostris* (Elasmobranchii: Rajidae); marine; spiral valve; adult; WTSP; Chile (Leible et al. 1990).

Note: host reported as *Raja flavirostris*.

*Mustelus mento* (Elasmobranchii: Triakidae); marine; spiral valve; adult; WTSP; Peru (Tresierra et al. 1986).

*Myliobatis goodei* (Elasmobranchii: Myliobatidae); marine; spiral valve; adult; WTSA (La Plata River estuary); Uruguay (Brooks et al. 1981a).

*Sympterygia bonapartii* (Elasmobranchii: Arhynchobatidae); marine; spiral valve; adult; WTSA; Argentina (Ostrowski de Núñez 1971).

Notes: host reported as *Psammobatis microps*. The author found only specimens without scolex, which she supposed to belong to *Phyllobothrium* (see Ostrowski de Núñez 1971).

*Urophycis brasiliensis* (Actinopterygii: Phycidae); marine; mesentery; metacestode: WTSA; Argentina (Szidat 1961).

*Zapteryx brevirostris* (Elasmobranchii: Rhinobatidae); marine; spiral valve; metacestode; WTSA; Argentina (Ostrowski de Núñez 1971).

***Scyphophyllidium uruguayense* Brooks, Marques, Perroni & Sidagis, 1999**

*Mustelus mento* (Elasmobranchii: Triakidae); marine; spiral valve; adult; WTSA; Uruguay (Brooks et al. 1999).

Note: type host.

***Thysanocephalum thysanocephalum* (Linton, 1889) Braun, 1900**

[Syns. *Phyllobothrium thysanocephalum* Linton, 1889 *nec Thysanocephalum crispum* (Linton, 1889) Linton, 1890 (*nomen nudum*)]

*Sphyrna zygaena* (Elasmobranchii: Sphyrnidae); marine; spiral valve; adult; WTSP; Peru (López de McDonald and Tantaleán 1985).

Notes: tapeworms reported as *Thysanocephalum crispum*. According to Ruhnke (2011), the genus *Thysanocephalum* should be provisionally retained in the Phyllobothriidae.

**Unidentified phyllobothriideans**

*Merluccius australis* (Actinopterygii: Merlucciidae); marine; intestine; metacestode; Magellanic; Chile, Falkland Islands (MacKenzie and Longshaw 1995).

*Merluccius hubbsi* (Actinopterygii: Merlucciidae); marine; intestine; metacestode; Magellanic; Argentina, Falkland Islands (MacKenzie and Longshaw 1995).

*Mugil liza* (Actinopterygii: Mugilidae); marine; site of infection not given; metacestode; WTSA; Brazil (Luque and Poulin 2004).

Note: host reported as *Mugil platanus*.

*Oncorhynchus mykiss* (Actinopterygii: Salmonidae); anadromous; intestine; metacestode; Aisén River basin; Chile (Torres et al. 2000).

*Prionotus* sp. (Actinopterygii: Triglidae); marine; intestine; metacestode; TSA; Brazil (Vicente and Santos 1974).

*Urophycis brasiliensis* (Actinopterygii: Phycidae); marine; mesentery; metacestode; WTSA; Brazil (Alves et al. 2004; Luque and Poulin 2004).

*Urophycis mystaceus* (Actinopterygii: Phycidae); marine; mesentery; metacestode; WTSA; Brazil (Alves et al. 2002c; Luque and Poulin 2004).

*Urophycis* sp. (Actinopterygii: Phycidae); marine; intestine; metacestode; TSA; Brazil (Vicente and Santos 1974).

**Taxa *incertae sedis***

*Guidus argentinense* Ivanov, 2006*

*Bathyraja brachyurops* (Elasmobranchii: Arhynchobatidae); marine; spiral valve; adult; WTSA; Argentina (Ivanov 2006).

Notes: type host. Ruhnke (2011) considers *Guidus* to represent a genus *incertae sedis*.

***Phyllobothrium sinuosiceps* Williams, 1959**

*Hexanchus griseus* (Elasmobranchii: Hexanchidae); marine; spiral valve; adult; WTSP; Chile (Carvajal 1974).

Notes: type host. This species somewhat resembles members of the genus *Crossobothrium*, but it is treated as *incertae sedis* by Ruhnke (2011).

#### Order Rhinebothriidea Healy, Caira, Jensen, Webster & Littlewood, 2009

##### Family Anthocephaliidae Ruhnke, Caira & Cox, 2015

*Anthocephalum gracile* Linton, 1890*

[Syns. *Phyllobothrium centrurum* Southwell, 1925; *Anthocephalum centrurum* (Southwell, 1925) Ruhnke, 1994]

*Dasyatis americana* (Elasmobranchii: Dasyatidae); marine; spiral valve; adult; TNA; Venezuela (Mayes and Brooks 1981).

Note: tapeworms reported as *Phyllobothrium centrurum*.

***Anthocephalum hobergi* (Zamparo, Brooks & Barriga, 1999) Marques & Caira, 2016**

[Syn. *Pararhinebothroides hobergi* Zamparo, Brooks & Barriga, 1999]

*Urobatis tumbesensis* (Elasmobranchii: Urotrygonidae); marine; spiral valve; adult; TEP; Ecuador (Zamparo et al. 1999; Marques and Caira 2016).

Notes: type host. Sequences of partial 18S (KU295561–KU295564) and 28S (KU295565–KU295568) (Marques and Caira 2016).

***Anthocephalum kingae* (Schmidt, 1978) Ruhnke & Seaman, 2009**

[Syn. *Phyllobothrium kingae* Schmidt, 1978]

*Dasyatis americana* (Elasmobranchii: Dasyatidae); marine; spiral valve; adult; TNA; Colombia (Brooks and Mayes 1980).

Note: tapeworms reported as Anthocephalum cf. kingae by Brooks and Mayes (1980).

*Urobatis jamaicensis* (Elasmobranchii: Urotrygonidae); marine; spiral valve; adult; TNA; Colombia (Brooks and Mayes 1980).

Note: type host; it was reported as *Urolophus jamaicensis*. Tapeworms reported as Anthocephalum cf. kingae by Brooks and Mayes (1980).

##### Family Echeneibothriidae de Beauchamp, 1905

***Echeneibothrium megalosoma* Carvajal & Dailey, 1975**

*Dipturus flavirostris* (Elasmobranchii: Rajidae); marine; spiral valve; adult; WTSP; Chile (Leible et al. 1990).

Note: host reported as *Raja flavirostris*.

*Zearaja chilensis* (Elasmobranchii: Rajidae); marine; spiral valve; adult; WTSP; Chile (Carvajal and Dailey 1975).

Notes: type host; it was reported as *Raja chilensis*.

***Echeneibothrium multiloculatum* Carvajal & Dailey, 1975**

*Dipturus flavirostris* (Elasmobranchii: Rajidae); marine; spiral valve; adult; WTSP; Chile (Leible et al. 1990).

Note: host reported as *Raja flavirostris*.

*Zearaja chilensis* (Elasmobranchii: Rajidae); marine; spiral valve; adult; WTSP; Chile (Carvajal and Dailey 1975; Carvajal et al. 1985).

Note: type host; it was reported as *Raja chilensis*.

***Echeneibothrium williamsi* Carvajal & Dailey, 1975**

*Dipturus flavirostris* (Elasmobranchii: Rajidae); marine; spiral valve; adult; WTSP; Chile (Leible et al. 1990).

Note: host reported as *Raja flavirostris*.

*Zearaja chilensis* (Elasmobranchii: Rajidae); marine; spiral valve; adult; WTSP; Chile (Carvajal and Dailey 1975; Carvajal et al. 1985).

Note: type host; it was reported as *Raja chilensis*.

*Notomegarhynchus navonae* Ivanov & Campbell, 2002*

*Atlantoraja castelnaui* (Elasmobranchii: Arhynchobatidae); marine; spiral valve; adult; WTSA; Argentina (Ivanov and Campbell 2002).

Note: type host.

##### Family Rhinebothriidae Euzet, 1953

***Rhinebothrium brooksi* Reyda & Marques, 2011**

*Paratrygon aiereba* (Elasmobranchii: Potamotrygonidae); freshwater; spiral valve; adult; Amazon River basin; Brazil (Reyda and Marques 2011).

Notes: type host. Sequences of partial *cox*1 (JF803719–JF803724) under the name *Rhinebothrium* sp. 1 (Reyda and Marques 2011).

*Potamotrygon orbignyi* (Elasmobranchii: Potamotrygonidae); freshwater; spiral valve; adult; Amazon River basin; Brazil (Reyda and Marques 2011).

***Rhinebothrium chilensis* Euzet & Carvajal, 1973**

*Sympterygia bonapartii* (Elasmobranchii: Arhynchobatidae); marine; spiral valve; adult; WTSA; Argentina (Tanzola et al. 1998).

*Sympterygia lima* (Elasmobranchii: Arhynchobatidae); marine; spiral valve; adult; WTSP; Chile (Euzet and Carvajal 1973).

Note: type host; it was reported as *Psammobatis lima*.

***Rhinebothrium copianullum* Reyda, 2008**

*Paratrygon aiereba* (Elasmobranchii: Potamotrygonidae); freshwater; spiral valve; adult; Amazon and Tocantins-Araguaia River basins; Brazil, Peru (Reyda 2008; Healy et al. 2009; Reyda and Marques 2011).

Notes: type host. Reyda and Marques (2011) redescribed this species and considered *Rhinebothrium* sp. 1 of Reyda (2008) to be conspecific; the latter author reported hyperparasitism caused by metacestodes of proteocephalids. Tapeworms reported as *Rhinebothrium* sp. 8 by Healy et al. (2009). Sequences of partial *cox*1 (JF803694–JF803698, JF803700, JF803701, JF803703–JF803710, JF803712–JF803714, JF803726–JF803728), 18S (FJ177090) and 28S (FJ177130) (Healy et al. 2009; Reyda and Marques 2011).

*Potamotrygon henlei* (Elasmobranchii: Potamotrygonidae); freshwater; spiral valve; adult; Tocantins-Araguaia River basin; Brazil (Reyda and Marques 2011).

*Potamotrygon leopoldi* (Elasmobranchii: Potamotrygonidae); freshwater; spiral valve; adult; Amazon River basin; Brazil (Reyda and Marques 2011).

Note: sequence of *cox*1 (JF803711) (Reyda and Marques 2011).

*Potamotrygon motoro* (Elasmobranchii: Potamotrygonidae); freshwater; spiral valve; adult (immature); Amazon River basin; Brazil (Reyda and Marques 2011).

Note: accidental host (Reyda and Marques 2011).

*Potamotrygon orbignyi* (Elasmobranchii: Potamotrygonidae); freshwater; spiral valve; adult; Amazon River basin; Brazil (Reyda and Marques 2011).

*Potamotrygon schroederi* (Elasmobranchii: Potamotrygonidae); freshwater; spiral valve; adult (immature); Amazon River basin; Brazil (Reyda and Marques 2011).

Note: accidental host, according to Reyda and Marques (2011).

*Potamotrygon tatianae* (Elasmobranchii: Potamotrygonidae); freshwater; spiral valve; adult (immature); Amazon River basin; Peru (Reyda and Marques 2011).

Notes: accidental host. Sequence of *cox*1 (JF803699) (Reyda and Marques 2011).

*Potamotrygon* sp. (Elasmobranchii: Potamotrygonidae); freshwater; spiral valve; adult; Amazon and Tocantins-Araguaia River basins; Brazil (Reyda and Marques 2011).

Notes: the authors distinguished four morphotypes of the host. Sequence of *cox*1 (JF803702, JF803715–JF803718) (Reyda and Marques 2011).

***Rhinebothrium corbatai* Menoret & Ivanov, 2011**

*Potamotrygon motoro* (Elasmobranchii: Potamotrygonidae); freshwater; spiral valve; adult; Paraná River basin; Argentina (Menoret and Ivanov 2011).

Note: type host.

***Rhinebothrium corymbum* Campbell, 1975**

*Dasyatis americana* (Elasmobranchii: Dasyatidae); marine; spiral valve; adult; TNA; Venezuela (Mayes and Brooks 1981).

Note: type host.

***Rhinebothrium fulbrighti* Reyda & Marques, 2011**

*Potamotrygon orbignyi* (Elasmobranchii: Potamotrygonidae); freshwater; spiral valve; adult; Amazon River basin (estuary); Brazil (Reyda and Marques 2011).

Notes: type host. Sequences of *cox*1 (JF803725, JF803729–JF803734) under the name *Rhinebothrium* sp. 2 (Reyda and Marques 2011).

*Potamotrygon* sp. (Elasmobranchii: Potamotrygonidae); freshwater; spiral valve; adult; Amazon River basin (estuary); Brazil (Reyda and Marques 2011).

***Rhinebothrium jaimei* Marques & Reyda, 2015**

*Potamotrygon orbignyi* (Elasmobranchii: Potamotrygonidae); freshwater; spiral valve; adult; Amazon River basin (estuary); Brazil (Marques and Reyda 2015).

Note: type host.

*Potamotrygon scobina* (Elasmobranchii: Potamotrygonidae); freshwater; spiral valve; adult; Amazon River basin (estuary); Brazil (Marques and Reyda 2015).

***Rhinebothrium leiblei* Euzet & Carvajal, 1973**

*Sympterygia lima* (Elasmobranchii: Arhynchobatidae); marine; spiral valve; adult; WTSP; Chile (Euzet and Carvajal 1973).

Note: type host; it was reported as *Psammobatis lima*.

***Rhinebothrium margaritense* Mayes & Brooks, 1981**

*Dasyatis americana* (Elasmobranchii: Dasyatidae); marine; spiral valve; adult; TNA; Venezuela (Mayes and Brooks 1981).

*Dasyatis guttata* (Elasmobranchii: Dasyatidae); marine; spiral valve; adult; TNA; Venezuela (Mayes and Brooks 1981).

Note: type host.

***Rhinebothrium mistyae* Menoret & Ivanov, 2011**

*Potamotrygon motoro* (Elasmobranchii: Potamotrygonidae); freshwater; spiral valve; adult; Paraná River basin; Argentina (Menoret and Ivanov 2011).

Note: type host.

***Rhinebothrium paratrygoni* Rego & Dias, 1976**

[Syn. *Rhinebothrium paranaensis* Menoret & Ivanov, 2009]

*Potamotrygon brachyura* (Elasmobranchii: Potamotrygonidae); freshwater; spiral valve; adult; Paraná River basin; Brazil (Reyda and Marques 2011).

*Potamotrygon falkneri* (Elasmobranchii: Potamotrygonidae); freshwater; spiral valve; adult; Paraná River basin; Argentina, Brazil, Paraguay (Brooks et al. 1981b; Lacerda et al. 2008, 2009; Menoret and Ivanov 2009a; Reyda and Marques 2011).

Note: sequences of *cox*1 (JF803684, JF803685, JF803687–JF803689, JF803691, JF803692) (Reyda and Marques 2011).

*Potamotrygon histrix* (Elasmobranchii: Potamotrygonidae); freshwater; spiral valve; adult; Paraná River basin; Brazil (Reyda and Marques 2011).

*Potamotrygon motoro* (Elasmobranchii: Potamotrygonidae); freshwater; spiral valve; adult; Amazon and Paraná River basins; Brazil (Brooks and Amato 1992; Reyda and Marques 2011).

Note: sequences of *cox*1 (JF803686, JF803690, JF803693) (Reyda and Marques 2011).

*Potamotrygon orbignyi* (Elasmobranchii: Potamotrygonidae); freshwater; spiral valve; adult; Orinoco River basin; Venezuela (Brooks et al. 1981b).

Note: host reported as *Paratrygon hystrix* and *Potamotrygon reticulatus* (for details, see Brooks and Amato 1992).

*Potamotrygon* sp. (Elasmobranchii: Potamotrygonidae); freshwater; spiral valve; adult; Amazon and Paraná River basins; Brazil (Rego and Dias 1976; Reyda and Marques 2011).

Note: type host; it was reported as *Elipesurus* sp.; Reyda and Marques (2011) distinguished two morphotypes of hosts that are most likely new for science.

***Rhinebothrium rhinobati* Dailey & Carvajal, 1976**

*Rhinobatos planiceps* (Elasmobranchii: Rhinobatidae); marine; spiral valve; adult; WTSP; Chile, Peru (Dailey and Carvajal 1976; Tantaleán 1991; Iannacone et al. 2011).

Note: type host.

***Rhinebothrium scobinae* Euzet & Carvajal, 1973**

*Psammobatis scobina* (Elasmobranchii: Arhynchobatidae); marine; spiral valve; adult; WTSP; Chile (Euzet and Carvajal 1973).

Note: type host.

***Rhinebothrium tetralobatum* Brooks, 1977**

*Himantura schmardae* (Elasmobranchii: Dasyatidae); marine; spiral valve; adult; TNA; Colombia (Brooks 1977).

Note: type host.

***Rhinebothrium* sp.**

*Gobionellus oceanicus* (Actinopterygii: Gobiidae); marine; body cavity; metacestode; TSA; Brazil (Palm 1997).

*Paratrygon aiereba* (Elasmobranchii: Potamotrygonidae); freshwater; spiral valve; adult; Amazon River basin; Peru (Reyda and Olson 2003).

Notes: they reported hyperparasitism caused by metacestodes of proteocephalids. Sequences of partial 28S (AY193880–AY193883) from adult tapeworms and partial 28S (AY193877–AY193879) from the encysted larval forms (Reyda and Olson 2003).

*Scomber colias* (Actinopterygii: Scombridae); marine; intestine, pyloric caeca; metacestode; WTSA; Brazil (Rego and Santos 1983).

Note: host reported as *Scomber japonicus*, but specimens from the Atlantic were re-assigned as *Scomber colias*, according to Froese and Pauly (2016).

*Synodus scituliceps* (Actinopterygii: Synodontidae); marine; intestine, pyloric caeca; metacestode; WTSP; Peru (Escalante et al. 1987).

***Rhinebothroides campbelli* Ivanov, 2004**

*Potamotrygon motoro* (Elasmobranchii: Potamotrygonidae); freshwater; spiral valve; adult; Paraná River basin; Argentina (Ivanov 2004).

Notes: type host. Ivanov (2004) arose doubts concerning the conspecificity between *Rhinebothroides venezuelensis* and *Rhinebothroides circularisi* proposed by Marques and Brooks (2003).

***Rhinebothroides freitasi* (Rego, 1979) Brooks, Mayes & Thorson, 1981**

[Syns. *Rhinebothrium freitasi* Rego, 1979; *Rhinebothroides circularisi* Mayes, Brooks & Thorson, 1981; *Rhinebothroides venezuelensis* Brooks, Mayes & Thorson, 1981]

*Potamotrygon constellata* (Elasmobranchii: Potamotrygonidae); freshwater; spiral valve; adult; Amazon River basin; Brazil (Mayes et al. 1981).

Note: host reported as *Potamotrygon circularis*, whereas tapeworms were reported as *Rhinebothroides circularisi*.

Potamotrygon cf. falkneri (Elasmobranchii: Potamotrygonidae); freshwater; spiral valve; adult; Amazon and Paraná River basins; Brazil, Peru (Marques and Brooks 2003; Healy et al. 2009; Caira et al. 2014).

Notes: Healy et al. (2009) and Caira et al. (2014) reported the tapeworms as Rhinebothroides cf. freitasi and the hosts as Potamotrygon cf. castexi. Sequences of partial 18S (FJ177092) and 28S (FJ177132) (Healy et al. 2009; Caira et al. 2014).

*Potamotrygon henlei* (Elasmobranchii: Potamotrygonidae); freshwater; spiral valve; adult; Tocantins-Araguaia River basin; Brazil (Marques and Brooks 2003).

*Potamotrygon leopoldi* (Elasmobranchii: Potamotrygonidae); freshwater; spiral valve; adult; Amazon River basin; Brazil (Marques and Brooks 2003).

*Potamotrygon motoro* (Elasmobranchii: Potamotrygonidae); freshwater; spiral valve; adult; Amazon and Paraná River basins; Argentina, Brazil (Brooks and Amato 1992; Marques and Brooks 2003).

*Potamotrygon orbignyi* (Elasmobranchii: Potamotrygonidae); freshwater; spiral valve; adult; Amazon and Orinoco River basins; Brazil, Venezuela (Rego 1979; Brooks et al. 1981b; Marques and Brooks 2003).

Notes: type host; it was reported as *Paratrygon hystrix* by Rego (1979) and Brooks et al. (1981b).

*Potamotrygon schroederi* (Elasmobranchii: Potamotrygonidae); freshwater; spiral valve; adult; Amazon River basin; Brazil (Marques and Brooks 2003).

*Potamotrygon scobina* (Elasmobranchii: Potamotrygonidae); freshwater; spiral valve; adult; Amazon River basin; Brazil (Marques and Brooks 2003).

*Potamotrygon yepezi* (Elasmobranchii: Potamotrygonidae); freshwater; spiral valve; adult; Maracaibo basin; Venezuela (Brooks et al. 1981b).

***Rhinebothroides glandularis* Brooks, Mayes & Thorson, 1981**

*Potamotrygon henlei* (Elasmobranchii: Potamotrygonidae); freshwater; spiral valve; adult; Tocantins-Araguaia River basin; Brazil (Marques and Brooks 2003).

*Potamotrygon motoro* (Elasmobranchii: Potamotrygonidae); freshwater; spiral valve; adult; Amazon and Paraná River basins; Argentina, Brazil (Marques and Brooks 2003; Reyda and Marques 2011).

Note: sequence of partial *cox*1 (JF803682) (Reyda and Marques 2011).

*Potamotrygon orbignyi* (Elasmobranchii: Potamotrygonidae); freshwater; spiral valve; adult; Amazon and Orinoco River basins; Brazil, Venezuela (Brooks et al. 1981b; Marques and Brooks 2003; Ivanov 2004).

Notes: type host; it was originally reported as *Paratrygon hystrix*. Ivanov (2004) studied the type specimens deposited in USNPC and HWML.

*Potamotrygon scobina* (Elasmobranchii: Potamotrygonidae); freshwater; spiral valve; adult; Amazon River basin; Brazil (Marques and Brooks 2003).

*Potamotrygon signata* (Elasmobranchii: Potamotrygonidae); freshwater; spiral valve; adult; Parnaíba River basin; Brazil (Marques and Brooks 2003).

*Potamotrygon* sp. (Elasmobranchii: Potamotrygonidae); freshwater; spiral valve; adult; Amazon and Orinoco River basins; Venezuela (Marques and Brooks 2003; Reyda and Marques 2011).

Notes: Marques and Brooks (2003) reported immature specimens. Sequence of partial *cox*1 (JF803683) (Reyda and Marques 2011).

***Rhinebothroides mclennanae* Brooks & Amato, 1992**

*Potamotrygon motoro* (Elasmobranchii: Potamotrygonidae); freshwater; spiral valve; adult; Paraná River basin; Argentina, Brazil (Brooks and Amato 1992; Ivanov 2004).

Notes: type host. Marques and Brooks (2003) synonymized this species with *Rhinebothroides glandularis*, but after evaluation of newly collected material, Ivanov (2004) considered this taxon a valid species.

*Rhinebothroides moralarai* (Brooks & Thorson, 1976) Mayes, Brooks & Thorson, 1981*

[Syn. *Rhinebothrium moralarai* Brooks & Thorson, 1976]

*Potamotrygon magdalenae* (Elasmobranchii: Potamotrygonidae); freshwater; spiral valve; adult; Magdalena River basin; Colombia (Brooks and Thorson 1976; Brooks et al. 1981b).

Notes: type host. Brooks et al. (1981b) studied the type specimens deposited in USNM.

*Potamotrygon* sp. (Elasmobranchii: Potamotrygonidae); freshwater; spiral valve; adult; Amazon River basin; Brazil (Marques and Brooks 2003; Reyda and Marques 2011).

Note: sequence of partial *cox*1 (JF803681) (Reyda and Marques 2011).

***Rhinebothroides scorzai* (Lopez-Neyra & Diaz-Ungria, 1958) Mayes, Brooks & Thorson, 1981**

[Syn. *Rhinebothrium scorzai* Lopez-Neyra & Diaz-Ungria, 1958]

*Paratrygon aiereba* (Elasmobranchii: Potamotrygonidae); freshwater; spiral valve; adult; Amazon River basin; Brazil (Marques and Brooks 2003).

*Potamotrygon motoro* (Elasmobranchii: Potamotrygonidae); freshwater; spiral valve; adult; Amazon and Paraná River basins; Brazil (Rego and Dias 1976; Marques and Brooks 2003).

*Potamotrygon orbignyi* (Elasmobranchii: Potamotrygonidae); freshwater; spiral valve; adult; Orinoco River basin; Venezuela (Lopez-Neyra and Diaz-Ungria 1958).

Note: type host; it was originally reported as *Paratrygon hystrix*.

*Potamotrygon* sp. (Elasmobranchii: Potamotrygonidae); freshwater; spiral valve; adult; Amazon River basin; Brazil (Reyda and Marques 2011).

Note: sequence of partial *cox*1 (JF803680) (Reyda and Marques 2011).

***Rhinebothroides* sp.**

*Paratrygon aiereba* (Elasmobranchii: Potamotrygonidae); freshwater; spiral valve; adult (immature); Amazon River basin; Peru (Reyda 2008).

Potamotrygon cf. falkneri (Elasmobranchii: Potamotrygonidae); freshwater; spiral valve; adult; Amazon and Paraná River basins; Brazil, Peru (Reyda and Olson 2003; Reyda 2008; Reyda and Marques 2011).

Notes: Reyda and Olson (2003) and Reyda (2008) reported the host as *Potamotrygon castexi* and the latter reported the cestodes as *Rhinebothroides* sp. 2. Reyda and Olson (2003) found metacestodes of proteocephalids parasitizing *Rhinebothroides* sp. Sequence of partial *cox*1 (JF803678) (Reyda and Marques 2011).

*Potamotrygon motoro* (Elasmobranchii: Potamotrygonidae); freshwater; spiral valve; adult; Amazon River basin; Peru (Reyda and Olson 2003, Reyda 2008).

Note: the authors reported hyperparasitism caused by metacestodes of proteocephalids.

*Potamotrygon tatianae* (Elasmobranchii: Potamotrygonidae); freshwater; spiral valve; adult; Amazon River basin; Peru (Reyda and Marques 2011).

Note: sequence of partial *cox*1 (JF803679) (Reyda and Marques 2011).

***Rhodobothrium mesodesmatum* (Bahamonde & Lopez, 1962) Campbell & Carvajal, 1979**

[Syns. *Proboscidosaccus mesodesmatis* Bahamonde & Lopez, 1962; *Anthobothrium peruanum* Rego, Vicente & Herrera, 1968]

*Myliobatis chilensis* (Elasmobranchii: Myliobatidae); marine; spiral valve; adult; WTSP; Chile, Peru (Campbell and Carvajal 1979; Rodríguez and Tantaleán 1980; Oliva 1982; Tresierra et al. 1986).

Note: type host. Tapeworm originally described by Bahamonde and Lopez (1962) from a clam, *Mesodesma donacium* (Lamarck), parasitizing the palleal cavity of this intermediate host, which is a common prey for *Myliobatis chilensis*.

*Myliobatis peruvianus* (Elasmobranchii: Myliobatidae); marine; spiral valve; adult; WTSP; Peru (López de McDonald and Tantaleán 1985).

*Sarda chiliensis* (Actinopterygii: Scombridae); marine; intestine; adult (?); WTSP; Peru (Rego et al. 1968).

***Rhodobothrium paucitesticulare* Mayes & Brooks, 1981**

*Rhinoptera bonasus* (Elasmobranchii: Myliobatidae); marine; spiral valve; adult; TNA; Venezuela (Mayes and Brooks 1981; Brooks et al. 1981a).

Note: type host.

*Rhodobothrium pulvinatum* Linton, 1889*

[Syns. *Anthobothrium pulvinatum* Linton, 1890 *nec* *Anthobothrium pulvinatum* Linton, 1889 (*nomen nudum*); *Inermiphyllidium pulvinatum* (Linton, 1890) Riser, 1955]

*Dasyatis americana* (Elasmobranchii: Dasyatidae); marine; spiral valve; adult; TNA; Venezuela (Mayes and Brooks 1981).

*Dasyatis guttata* (Elasmobranchii: Dasyatidae); marine; spiral valve; adult; TNA; Venezuela (Mayes and Brooks 1981).

***Scalithrium magniphallum* (Brooks, 1977) Ball, Neifar & Euzet, 2003**

[Syn. *Rhinebothrium magniphallum* Brooks, 1977]

*Dasyatis americana* (Elasmobranchii: Dasyatidae); marine; spiral valve; adult; TNA; Colombia (Brooks and Mayes 1980).

*Dasyatis guttata* (Elasmobranchii: Dasyatidae); marine; spiral valve; adult; TNA; Venezuela (Mayes and Brooks 1981).

*Himantura schmardae* (Elasmobranchii: Dasyatidae); marine; spiral valve; adult; TNA; Colombia, Venezuela (Brooks 1977; Mayes and Brooks 1981).

Note: type host.

*Urobatis jamaicensis* (Elasmobranchii: Urotrygonidae); marine; spiral valve; adult; TNA; Colombia (Brooks and Mayes 1980).

Notes: host reported as *Urolophus jamaicensis*.

*Urotrygon venezuelae* (Elasmobranchii: Urotrygonidae); marine; spiral valve; adult; TNA; Colombia (Brooks and Mayes 1980).

**Taxa *incertae sedis***

*Anindobothrium anacolum* (Brooks, 1977) Marques, Brooks & Lasso, 2001*

[Syn. *Caulobothrium anacollum* Brooks, 1977]

*Himantura schmardae* (Elasmobranchii: Dasyatidae); marine; spiral valve; adult; TNA; Colombia (Brooks 1977).

Notes: type host. The genus *Anindobothrium* is likely a member of the Rhinebothriidea (see Ruhnke 2011) and it is already treated as such in the GCD within Anthocephalidae (Caira et al. 2012).

***Anindobothrium lisae* Marques, Brooks & Lasso, 2001**

*Potamotrygon orbignyi* (Elasmobranchii: Potamotrygonidae); freshwater; spiral valve; adult; Amazon River basin; Brazil (Marques et al. 2001).

Note: type host.

***Phyllobothrium auricula* van Beneden, 1858**

*Myliobatis chilensis* (Elasmobranchii: Myliobatidae); marine; spiral valve; adult; WTSP; Peru (Tantaleán 1991).

Note: the species is likely a member of the Rhinebothriidea (see Ruhnke 2011) and it is already treated as such in the GCD (Caira et al. 2012).

*Myliobatis peruvianus* (Elasmobranchii: Myliobatidae); marine; spiral valve; adult; WTSP; Peru (Tantaleán 1991).

***Phyllobothrium discopygi* Campbell & Carvajal, 1987**

*Discopyge tschudi* (Elasmobranchii: Torpedinidae); marine; spiral valve; adult; WTSP; Chile (Campbell and Carvajal 1987).

Notes: type host. This species is likely a member of the Rhinebothriidea (see Ruhnke 2011) and it is already treated as such in the GCD (Caira et al. 2012).

***Phyllobothrium myliobatidis* Brooks, Mayes & Thorson, 1981**

*Myliobatis goodei* (Elasmobranchii: Myliobatidae); marine; spiral valve; adult; WTSA (La Plata River estuary); Uruguay (Brooks et al. 1981a).

Notes: type host. The tapeworm is likely a member of the Rhinebothriidea (see Ruhnke 2011) and it is already treated as such in the GCD (Caira et al. 2012).

#### Order ‘Tetraphyllidea’ Carus, 1863

***Anthobothrium laciniatum* Linton, 1890**

*Carcharhinus longimanus* (Elasmobranchii: Carcharhinidae); marine; spiral valve; adult; TSA; Brazil (Rego 1977).

***Anthobothrium galeorhini* Suriano, 2002**

*Galeorhinus galeus* (Elasmobranchii: Triakidae); marine; spiral valve; adult; Magellanic; Argentina (Suriano 2002).

Note: type host.

***Calliobothrium australis* Ostrowski de Núñez, 1973**

*Mustelus schmitti* (Elasmobranchii: Triakidae); marine; spiral valve; adult; WTSA; Argentina, Uruguay (Ostrowski de Núñez 1973; Ivanov and Brooks 2002; Alarcos et al. 2006; Bernot et al. 2015).

Notes: type host. This species was originally described as *Calliobothrium verticillatum australis*, but Ivanov and Brooks (2002) redescribed this species and they raised this subspecies to the species level as *Calliobothrium australis*. Sequences of partial 28S (KP128030, KP128031) (Bernot et al. 2015).

*Calliobothrium verticillatum* (Rudolphi, 1819) van Beneden, 1850*

[Syns. *Bothriocephalus verticillatus* Rudolphi, 1819; *Acanthobothrium verticillatum* (Rudolphi, 1819) van Beneden, 1849]

*Mustelus mento* (Elasmobranchii: Triakidae); marine; spiral valve; adult; WTSP; Chile, Peru (Carvajal 1974; Escalante and Carvajal 1981).

***Calliobothrium* sp.**

*Mustelus mento* (Elasmobranchii: Triakidae); marine; spiral valve; adult; WTSP; Peru (Tresierra et al. 1986).

***Caulobothrium myliobatidis* Carvajal, 1977**

*Myliobatis chilensis* (Elasmobranchii: Myliobatidae); marine; spiral valve; adult; WTSP; Chile, Peru (Carvajal 1977; Tantaleán 1991).

Note: type host.

***Caulobothrium ostrowskiae* Brooks, Mayes & Thorson, 1981**

*Myliobatis goodei* (Elasmobranchii: Myliobatidae); marine; spiral valve; adult; WTSA (La Plata River estuary); Uruguay (Brooks et al. 1981a).

Note: type host.

***Caulobothrium uruguayense* Brooks, Mayes & Thorson, 1981**

*Myliobatis goodei* (Elasmobranchii: Myliobatidae); marine; spiral valve; adult; WTSA (La Plata River estuary); Uruguay (Brooks et al. 1981a).

Note: type host; it was reported as *Myliobatis uruguayensis*.

***Dioecotaenia campbelli* Mayes & Brooks, 1981**

*Rhinoptera bonasus* (Elasmobranchii: Myliobatidae); marine; spiral valve; adult; TNA; Venezuela (Brooks et al. 1981a; Mayes and Brooks 1981).

Note: type host.

*Reesium paciferum* (Sproston, 1948) Euzet, 1955*

[Syn. *Dinobothrium paciferum* Sproston, 1948]

*Cetorhinus maximus* (Elasmobranchii: Cetorhinidae); marine; spiral valve; adult; WTSP; Peru (Rivera and Sarmiento 1990).

Note: type host.

*Serendip deborahae* Brooks & Barriga, 1995*

*Rhinoptera steindachneri* (Elasmobranchii: Myliobatidae); marine; spiral valve; adult; TEP; Ecuador (Brooks and Barriga 1995).

Notes: type host. *Serendip* Brooks & Barriga, 1995 is likely a member of the Rhinebothriidea, according to Ruhnke et al. (2015).

***Symcallio barbarae* (Ivanov & Brooks, 2002) Bernot, Caira & Pickering, 2015**

[Syns. *Calliobothrium eschrichti* of Ostrowski de Núñez (1973) *nec* van Beneden, 1850; *Calliobothrium barbarae* Ivanov & Brooks, 2002]

*Mustelus schmitti* (Elasmobranchii: Triakidae); marine; spiral valve; adult; WTSA; Argentina, Uruguay (Ostrowski de Núñez 1973; Ivanov and Brooks 2002; Alarcos et al. 2006; Bernot et al. 2015).

Note: type host. Ostrowski de Núñez (1973) reported the tapeworms as *Calliobothrium eschrichti* van Beneden, 1850. Sequence of partial 28S (KP128023) (Bernot et al. 2015).

***Symcallio lintoni* (Euzet, 1954) Bernot, Caira & Pickering, 2015**

[Syn. *Calliobothrium lintoni* Euzet, 1954]

*Mustelus whitneyi* (Elasmobranchii: Triakidae); marine; spiral valve; adult; WTSP; Peru (Escalante and Carvajal 1981).

***Symcallio lunae* (Ivanov & Brooks, 2002) Bernot, Caira & Pickering, 2015**

[Syns. of *Calliobothrium lintoni* of Ostrowski de Núñez (1973) *nec* Euzet, 1974; *Calliobothrium lunae* Ivanov & Brooks, 2002]

*Mustelus schmitti* (Elasmobranchii: Triakidae); marine; spiral valve; adult; WTSA; Argentina, Uruguay (Ostrowski de Núñez 1973; Ivanov and Brooks 2002; Alarcos et al. 2006).

Notes: type host. Ostrowski de Núñez (1973) reported this species as *Calliobothrium lintoni* (see Caira et al. 2012).

**Collective group name for larval ‘tetraphyllideans’ and Unidentified taxa**

‘ ***Scolex pleuronectis* ’ Müller, 1788; ‘ *Scolex polymorphus* ’ Rudolphi, 1819; ‘ *Scolex* sp.’; ‘Tetraphyllidea gen. sp.**’

*Anchoa tricolor* (Actinopterygii: Engraulidae); marine; intestine; metacestode; WTSA; Brazil (Tavares et al. 2005).

*Aphos porosus* (Actinopterygii: Batrachoididae); marine; site of infection not given; metacestode; WTSP; Chile (Torres et al. 1993; Cortés and Muñoz 2008, 2009).

*Aspistor luniscutis* (Actinopterygii: Ariidae); marine; site of infection not given; metacestode; WTSA; Brazil (Luque and Poulin 2004).

Note: host reported as *Sciadeichthys luniscutis*.

*Balistes capriscus* (Actinopterygii: Balistidae); marine; site of infection not given; metacestode; WTSA; Brazil (Luque and Poulin 2004; Alves et al. 2005).

*Balistes vetula* (Actinopterygii: Balistidae); marine; site of infection not given; metacestode; WTSA; Brazil (Luque and Poulin 2004).

*Brachyplatystoma* sp. (Actinopterygii: Pimelodidae); freshwater; site of infection not given; metacestode; Amazon River basin (estuary of Amazon River); Brazil (Rego et al. 1999a).

*Caranx latus* (Actinopterygii: Carangidae); marine; site of infection not given; metacestode; WTSA; Brazil (Luque and Alves 2001).

*Cilus gilberti* (Actinopterygii: Sciaenidae); marine; site of infection not given; metacestode; WTSP; Chile (Garcías et al. 2001).

*Conger orbignianus* (Actinopterygii: Congridae); marine; intestine; metacestode; WTSA; Argentina (Tanzola and Guagliardo 2000; Timi and Lanfranchi 2013).

Note: Tanzola and Guagliardo (2000) distinguished two morphotypes.

*Coryphaena hippurus* (Actinopterygii: Coryphaenidae); marine; intestine; metacestode; WTSP; Peru (Escalante et al. 1987).

*Cynoscion guatucupa* (Actinopterygii: Sciaenidae); marine; pyloric caeca, intestine; metacestode; WTSA; Argentina (Timi et al. 2005, 2010b).

*Dactylopterus volitans* (Actinopterygii: Dactylopteridae); marine; mesentery; metacestode; WTSA; Brazil (Luque and Poulin 2004; Cordeiro and Luque 2005).

*Dissostichus eleginoides* (Actinopterygii: Nototheniidae); marine; stomach, intestine; metacestode; Magellanic; Falkland Islands (Brickle et al. 2006; Brown et al. 2013).

*Eleginops maclovinus* (Actinopterygii: Eleginopsidae); marine; intestine; metacestode; Magellanic; Falkland Islands (Brickle and MacKenzie 2007).

*Engraulis anchoita* (Actinopterygii: Engraulidae); marine; pyloric caeca; metacestode; Magellanic, WTSA; Argentina (Timi 2003; Timi and Poulin 2003; Timi et al. 2010b).

*Engraulis ringens* (Actinopterygii: Engraulidae); marine; site of infection not

given; metacestode; WTSP; Chile (George-Nascimento and Moscoso 2013).

*Ethmidium maculatum* (Actinopterygii: Clupeidae); marine; intestine; metacestode; WTSP; Peru (Escalante et al. 1987).

Note: host reported as *Brevoortia maculata*.

*Euthynnus alletteratus* (Actinopterygii: Scombridae); marine; intestine; metacestode; WTSA; Brazil (Luque and Poulin 2004; Alves and Luque 2006).

*Genidens barbus* (Actinopterygii: Ariidae); marine; site of infection not given; metacestode; WTSA; Brazil (Luque and Poulin 2004; Tavares and Luque 2004, 2008).

Note: Luque and Poulin (2004) and Tavares and Luque (2004) reported the host as *Netuma barba*.

*Genypterus blacodes* (Actinopterygii: Ophidiidae); marine; intestine; metacestode; WTSA; Argentina (Sardella et al. 1998).

*Genypterus brasiliensis* (Actinopterygii: Ophidiidae); marine; intestine; metacestode; WTSA; Brazil (Alves et al. 2002a, b; Luque and Poulin 2004).

*Genypterus maculatus* (Actinopterygii: Ophidiidae); marine; intestine; metacestode; WTSP; Chile, Peru (Escalante et al. 1987; Muñoz and George-Nascimento 2008).

*Gobiesox marmoratus* (Actinopterygii: Gobiesocidae); marine; site of infection not given; metacestode; WTSP; Chile (Muñoz 2014).

*Gymnothorax moringa* (Actinopterygii: Muraenidae); marine; site of infection not given; metacestode; WTSA; Brazil (Luque and Poulin 2004).

*Haemulon steindachneri* (Actinopterygii: Haemulidae); marine; intestine; metacestode; WTSA; Brazil (Luque et al. 1995, 1996a, b; Luque and Poulin 2004).

*Helcogrammoides chilensis* (Actinopterygii: Tripterygiidae); marine; site of

infection not given; metacestode; WTSP; Chile (Muñoz and Delorme 2011).

*Hippoglossina macrops* (Actinopterygii: Paralichthyidae); marine; intestine; metacestode WTSP; Chile (Riffo 1991; González et al. 2001, 2008; Oliva et al. 2004).

*Hyporhamphus unifasciatus* (Actinopterygii: Hemiramphidae); brackish, marine; body cavity; metacestode; TSA (Itamaracá Island); Brazil (Palm 1997).

*Katsuwonus pelamis* (Actinopterygii: Scombridae); marine; intestine; metacestode; WTSA, WTSP; Brazil, Peru (Escalante et al. 1987; Alves and Luque 2006)

*Macruronus magellanicus* (Actinopterygii: Merlucciidae); marine; intestine; metacestode; Magellanic; Chile (Oliva 2001).

*Menticirrhus americanus* (Actinopterygii: Sciaenidae); marine; site of infection not given; metacestode; WTSA; Brazil (Luque and Poulin 2004).

*Merluccius australis* (Actinopterygii: Merlucciidae); marine; intestine; metacestode; Magellanic; Chile (Fernández 1985).

*Merluccius gayi gayi* (Actinopterygii: Merlucciidae); marine; intestine; metacestode; WTSP; Chile (Oliva and Ballón 2002).

*Merluccius hubbsi* (Actinopterygii: Merlucciidae); marine; pyloric caeca, intestine; metacestode; Magellanic, WTSA; Argentina, Uruguay (Szidat 1955, 1961; Sardella and Timi 1996, 2004).

*Micropogonias furnieri* (Actinopterygii: Sciaenidae); marine; intestine; metacestode; WTSA; Brazil (Alves and Luque 2001a; Luque and Poulin 2004; Luque et al. 2010).

*Mugil cephalus* (Actinopterygii: Mugilidae); marine; intestine; metacestode; WTSP; Chile (Fernández 1987).

*Mugil liza* (Actinopterygii: Mugilidae); catadromous; intestine; metacestode; WTSA; Brazil (Knoff et al. 1997; Luque and Poulin 2004).

Notes: host reported as *Mugil platanus*. Knoff et al. (1997) distinguished two morphotypes.

*Normanichthys crockeri* (Actinopterygii: Normanichthyidae); marine; intestine, ovary; metacestode; Magellanic; Chile (Sepúlveda et al. 2004).

Notothenia cf. angustata (Actinopterygii: Nototheniidae); marine; intestine; metacestode; WTSP; Chile (Muñoz et al. 2001).

*Odontesthes argentinensis* (Actinopterygii: Atherinopsidae); brackish, marine; site of infection not given; metacestode; WTSA (Mar Chiquita coastal lagoon); Argentina (Alarcos and Etchegoin 2010).

*Odontesthes regia* (Actinopterygii: Atherinopsidae); brackish, marine; intestine; metacestode; WTSP; Peru (Escalante et al. 1987).

Note: host reported as *Odontesthes regia regia*.

*Odontesthes smitti* (Actinopterygii: Atherinopsidae); marine; body cavity, mesentery, stomach; metacestode; Magellanic; Argentina (Carballo et al. 2011, 2012).

*Oligoplites palometa* (Actinopterygii: Carangidae); marine; intestine; metacestode; WTSA; Brazil (Takemoto et al. 1996a, b; Luque and Poulin 2004).

*Oligoplites saliens* (Actinopterygii: Carangidae); marine; intestine; metacestode; WTSA; Brazil (Takemoto et al. 1996a, b; Luque and Poulin 2004).

*Oligoplites saurus* (Actinopterygii: Carangidae); marine; intestine; metacestode; WTSA; Brazil (Takemoto et al. 1996a, b; Luque and Poulin 2004).

*Oncorhynchus kisutch* (Actinopterygii: Salmonidae); anadromous; intestine; metacestode; lakes in Chiloé Island; Chile (Torres et al. 1990, 2010).

*Orthopristis ruber* (Actinopterygii: Haemulidae); marine; intestine; metacestode; WTSA; Brazil (Luque et al. 1995, 1996a, b; Luque and Poulin 2004).

*Pagrus pagrus* (Actinopterygii: Sparidae); marine; mesentery; metacestode; WTSA; Brazil (Paraguassú et al. 2002; Luque and Poulin 2004; Soares et al. 2014).

*Paralichthys adspersus* (Actinopterygii: Paralichthyidae); marine; intestine; metacestode; WTSP; Chile (Riffo 1995; Oliva et al. 1996).

*Paralichthys isosceles* (Actinopterygii: Paralichthyidae); marine; stomach, intestine; metacestode; WTSA; Argentina, Brazil (Felizardo et al. 2010; Alarcos and Timi 2012; Alarcos et al. 2016).

*Paralichthys microps* (Actinopterygii: Paralichthyidae); marine; intestine; metacestode; WTSP; Chile (Riffo 1995).

*Paralichthys orbignyanus* (Actinopterygii: Paralichthyidae); marine; site of

infection not given; metacestode; WTSA (Mar Chiquita coastal lagoon); Argentina (Alarcos and Etchegoin 2010).

*Paralichthys patagonicus* (Actinopterygii: Paralichthyidae); marine; stomach, intestine; metacestode; WTSA; Argentina (Alarcos and Timi 2012).

*Paralonchurus brasiliensis* (Actinopterygii: Sciaenidae); marine; intestine; metacestode; WTSA; Brazil (Ribeiro et al. 2002; Luque et al. 2003; Luque and Poulin 2004).

*Parona signata* (Actinopterygii: Carangidae); marine; intestine; metacestode; WTSA; Argentina (Szidat 1969).

*Percophis brasiliensis* (Actinopterygii: Percophidae); marine; intestine; metacestode; WTSA; Argentina, Uruguay (Braicovich and Timi 2008, 2010).

*Pinguipes brasilianus* (Actinopterygii: Pinguipedidae); marine; intestine; metacestode; WTSA; Argentina, Brazil (Timi et al. 2008, 2009, 2010a).

*Pomatomus saltatrix* (Actinopterygii: Pomatomidae); marine; intestine; metacestode; WTSA; Brazil (Luque and Chaves 1999; Luque and Poulin 2004).

Note: host reported as *Pomatomus saltator*.

*Porichthys porosissimus* (Actinopterygii: Batrachoididae) marine; intestine; metacestode; WTSA (Bahía Blanca estuary); Argentina (Tanzola et al. 1997; Guagliardo et al. 2009).

Notes: Tanzola et al. (1997) distinguished two morphotypes. Guagliardo et al. (2009) studied lesions caused by the larvae.

*Priacanthus arenatus* (Actinopterygii: Priacanthidae); marine; intestine; metacestode; WTSA; Brazil (Tavares et al. 2001; Luque and Poulin 2004).

*Prolatilus jugularis* (Actinopterygii: Pinguipedidae); marine; intestine, ovary; metacestode; Magellanic; Chile (Sepúlveda et al. 2004).

*Pseudopercis numida* (Actinopterygii: Pinguipedidae); marine; intestine; metacestode; WTSA; Brazil (Luque et al. 2008).

*Pseudopercis semifasciata* (Actinopterygii: Pinguipedidae); marine; intestine; metacestode; Magellanic, WTSA; Argentina, Brazil (Luque et al. 2008; Timi and Lanfranchi 2009a).

*Raneya brasiliensis* (Actinopterygii: Ophidiidae); marine; mesentery; metacestode; WTSA; Argentina (Vales et al. 2011).

*Salmo trutta* (Actinopterygii: Salmonidae); anadromous; intestine; metacestode; lakes in Chiloé Island; Chile (Torres et al. 1990).

*Sarda chiliensis* (Actinopterygii: Scombridae); marine; intestine; metacestode; WTSP; Peru (Escalante et al. 1987).

Note: host reported as *Sarda chiliensis chiliensis*.

*Sarda sarda* (Actinopterygii: Scombridae); marine; intestine; metacestode; WTSA; Brazil (Alves and Luque 2006).

*Sardinella brasiliensis* (Actinopterygii: Clupeidae); marine; pyloric caeca (cysts); metacestode; WTSA; Brazil (Lima et al. 1997).

*Sardinops sagax* (Actinopterygii: Clupeidae); marine; intestine; metacestode; WTSP; Peru (Escalante et al. 1987).

Note: host reported as *Sardinops sagax sagax*.

*Scomber colias* (Actinopterygii: Scombridae); marine; body cavity, intestine, stomach; metacestode; WTSA; Argentina, Brazil (Rego and Santos 1983; Cremonte and Sardella 1997; Abdallah et al. 2002; Alves et al. 2003; Luque and Poulin 2004; Oliva et al. 2008b).

Note: host reported as *Scomber japonicus*.

*Scomber japonicus* (Actinopterygii: Scombridae); marine; intestine; metacestode; WTSP; Peru (Escalante et al. 1987; Cruces et al. 2014).

*Scomberomorus brasiliensis* (Actinopterygii: Scombridae); marine; intestine; metacestode; WTSA; Brazil (Luque and Poulin 2004; Alves and Luque 2006).

*Sebastes capensis* (Actinopterygii: Sebastidae); marine; site of infection not

given; metacestode; WTSP; Chile (González and Poulin 2005a, b; González et al. 2006).

*Sicyases sanguineus* (Actinopterygii: Gobiesocidae); marine; site of infection not given; metacestode; WTSP; Chile (Muñoz and Delorme 2011).

*Stellifer minor* (Actinopterygii: Sciaenidae); marine; intestine; metacestode; WTSP; Peru (Luque 1991).

*Strongylura scapularis* (Actinopterygii: Belonidae); marine; intestine; metacestode; WTSP; Peru (Luque 1991).

Note: host reported as *Belone scapularis*.

*Synodus scituliceps* (Actinopterygii: Synodontidae); marine; intestine; metacestode; WTSP; Peru (Escalante et al. 1987).

*Trachurus lathami* (Actinopterygii: Carangidae); marine; intestine; metacestode; WTSA; Argentina, Brazil (Luque and Poulin 2004; Braicovich et al. 2012).

*Trachurus murphyi* (Actinopterygii: Carangidae); marine; intestine; metacestode; WTSP; Chile, Peru (Oliva 1982, 1994, 1999; Luque 1991; Jara 1998).

Note: Luque (1991) and Jara (1998) reported the host as *Trachurus symmetricus murphyi*.

*Trichiurus lepturus* (Actinopterygii: Trichiuridae); marine; stomach, intestine; metacestode; WTSA; Brazil (Silva et al. 2000a, b; Luque and Poulin 2004; Carvalho and Luque 2011; Bueno et al. 2014).

*Tylosurus acus acus* (Actinopterygii: Belonidae); marine; intestine; metacestode; WTSA; Brazil (Tavares et al. 2004; Luque and Poulin 2004).

Note: host reported as *Tylosurus acus*.

*Urophycis brasiliensis* (Actinopterygii: Phycidae); marine; intestine; metacestode; WTSA; Argentina, Brazil (Szidat 1960, 1961; Alves et al. 2004; Luque and Poulin 2004; Pereira et al. 2014).

Note: Pereira et al. (2014) distinguished two morphotypes of larvae.

*Urophycis mystaceus* (Actinopterygii: Phycidae); marine; intestine; metacestode; WTSA; Brazil (Alves et al. 2002c; Luque and Poulin 2004).

*Xystreurys rasile* (Actinopterygii: Paralichthyidae); marine; stomach, intestine; metacestode; WTSA; Argentina (Szidat 1961; Alarcos and Timi 2012, 2013).

**Taxon *incertae sedis***

***Anthobothrium pristis* Woodland, 1934**

*Pristis pristis* (Elasmobranchii: Pristidae); brackish, freshwater, marine; spiral valve; adult; Amazon River basin; Brazil (Woodland 1934c).

Note: host reported as *Pristis perotteti*. Ruhnke and Caira (2009) did not place this species among six species of *Anthobothrium* (*sensu stricto*); therefore, it is considered *incertae sedis* (see Caira et al. 2012).

#### Order Trypanorhyncha Diesing, 1863

##### Suborder Trypanoselachoida Olson, Caira, Jensen, Overstreet, Palm & Beveridge, 2010

###### Superfamily Gymnorhynchoidea Dollfus, 1935

####### Family Gilquiniidae Dollfus, 1935

*Gilquinia squali* (Fabricius, 1794) Dollfus, 1930*

[Syns. *Taenia squali* Fabricius, 1794; *Bothriocephalus paleaceus* Rudolphi, 1810; *Rhynchobothrium tetrabothrium* van Beneden, 1849; *Tetrarhynchobothrium affine* Diesing, 1854; *Tetrarhynchus anteroporus* Hart, 1936]

*Etmopterus granulosus* (Elasmobranchii: Etmopteridae); marine; spiral valve; adult (immature); WTSP; Chile (Carvajal 1974).

Note: host reported as *Centroscyllium granulosus*.

***Gilquinia* sp.**

*Micropogonias furnieri* (Actinopterygii: Sciaenidae); marine; mesentery; metacestode; WTSA (La Plata River estuary); Argentina (Suriano 1966).

Note: Pereira (1993) suggested that these specimens might have corresponded to two different species of *Pterobothrium* Diesing, 1850.

####### Family Gymnorhynchidae Dollfus, 1935

***Gymnorhynchus isuri* Robinson, 1959**

*Isurus oxyrinchus* (Elasmobranchii: Lamnidae); marine; spiral valve; adult; WTSA; Brazil (Knoff et al. 2002, 2007).

*Molicola horridus* (Goodsir, 1841) Dollfus, 1935*

[For synonyms, see Palm (2004)]

*Isurus oxyrinchus* (Elasmobranchii: Lamnidae); marine; spiral valve; adult; WTSA; Brazil (Palm 2004).

*Mola ramsayi* (Actinopterygii: Molidae); marine; liver; metacestode; WTSP; Chile (Villalba and Fernández 1985).

Note: tapeworms reported as Gymnorhynchus (Molicola) horridus.

*Prionace glauca* (Elasmobranchii: Carcharhinidae); marine; spiral valve; adult; WTSA; Brazil (Knoff et al. 2002, 2004c).

***Molicola* sp.**

*Mola mola* (Actinopterygii: Molidae); marine; site of infection not given; metacestode; WTSA; Brazil (Palm 2004).

*Thyrsites atun* (Actinopterygii: Gempylidae); marine; muscles; metacestode; WTSP; Chile (Torres et al. 2014).

###### Superfamily Lacistorhynchoidea Guiart, 1927

####### Family Lacistorhynchidae Guiart, 1927

*Callitetrarhynchus gracilis* (Rudolphi, 1819) Pintner, 1931*

[For synonyms, see Palm (2004)]

*Balistes capriscus* (Actinopterygii: Balistidae); marine; liver, mesentery; metacestode; WTSA; Brazil (Dias et al. 2009).

*Balistes vetula* (Actinopterygii: Balistidae); marine; body cavity, muscle; metacestode; WTSA; Brazil (São Clemente et al. 1995).

*Caranx crysos* (Actinopterygii: Carangidae); marine; body cavity; metacestode; TSA; Brazil (Palm 1997).

*Caranx hippos* (Actinopterygii: Carangidae); marine; mesentery; metacestode; WTSA; Brazil (Luque and Alves 2001; Luque and Poulin 2004).

*Caranx latus* (Actinopterygii: Carangidae); marine; mesentery; metacestode; WTSA; Brazil (Luque and Alves 2001; Luque and Poulin 2004; Ferreira et al. 2006).

*Centropomus undecimalis* (Actinopterygii: Centropomidae); amphidromous; peritoneum; metacestode; NBS (Marajó Island); Brazil (Dollfus 1942).

Note: the cestodes were collected by Göldi in 1896.

*Chloroscombrus chrysurus* (Actinopterygii: Carangidae); marine; body cavity; metacestode; TSA, WTSA; Brazil (Palm 1997, 2004).

*Cynoscion acoupa* (Actinopterygii: Sciaenidae); marine; muscles; metacestode; NBS; Brazil (Dias et al. 2011b).

*Cynoscion guatucupa* (Actinopterygii: Sciaenidae); marine; body cavity, kidney; metacestode; WTSA; Argentina, Brazil, Uruguay (Pereira and Boeger 2005; Timi et al. 2005, 2010b).

*Euthynnus alletteratus* (Actinopterygii: Scombridae); marine; mesentery; metacestode; WTSA; Brazil (Luque and Poulin 2004; Alves and Luque 2006).

*Genidens barbus* (Actinopterygii: Ariidae); marine; body cavity, viscera; metacestode; WTSA; Brazil (São Clemente et al. 1991a).

Note: host reported as *Netuma barba*.

*Genypterus brasiliensis* (Actinopterygii: Ophidiidae); marine; body cavity, mesentery, muscles; metacestode; WTSA; Brazil (São Clemente et al. 2004; Knoff et al. 2008).

*Haemulon aurolineatum* (Actinopterygii: Haemulidae); marine; body cavity, muscles; metacestode; TSA; Brazil (Palm 1997).

*Harengula clupeola* (Actinopterygii: Clupeidae); marine; body cavity; metacestode; TSA; Brazil (Palm 1997).

*Hemilutjanus macrophthalmos* (Actinopterygii: Sciaenidae); marine; surface of

internal organs, serous membrane; metacestode; WTSP; Peru (Escalante and Carvajal 1984).

*Hyporthodus niveatus* (Actinopterygii: Serranidae); marine; body cavity; metacestode; WTSA; Brazil (Palm 2004).

Note: host reported as *Epinephelus niveatus*.

*Larimus breviceps* (Actinopterygii: Sciaenidae); marine; body cavity; metacestode; TSA; Brazil (Palm 1997).

*Lutjanus synagris* (Actinopterygii: Lutjanidae); marine; muscles; metacestode; WTSA; Brazil (Silva and São Clemente 2001).

*Macrodon ancylodon* (Actinopterygii: Sciaenidae); marine; body cavity, kidney, mesentery; metacestode; WTSA; Brazil (Sabas and Luque 2003; Luque and Poulin 2004; Pereira and Boeger 2005).

*Merluccius gayi peruanus* (Actinopterygii: Merlucciidae); marine; mesentery; metacestode; WTSP; Peru (Durán and Oliva 1980; Chero et al. 2014a).

*Micropogonias furnieri* (Actinopterygii: Sciaenidae); marine; body cavity, kidney, mesentery; metacestode; TNA, WTSA; Brazil, Venezuela (São Clemente 1986a, b, 1987; Vicente et al. 1989; Pereira 1993; Alves and Luque 1999, 2001a; Luque and Poulin 2004; Palm 2004; Pereira and Boeger 2005; Luque et al. 2010; Timi et al. 2010b).

*Mustelus canis* (Elasmobranchii: Triakidae); marine; spiral valve; adult; WTSA; Brazil (São Clemente and Gomes 1989b; São Clemente et al. 1991b).

*Oligoplites palometa* (Actinopterygii: Carangidae); marine; body cavity; metacestode; TSA, WTSA; Brazil (Takemoto et al. 1996a, b; Palm 1997; Luque and Poulin 2004).

*Oligoplites saurus* (Actinopterygii: Carangidae); marine; body cavity; metacestode; WTSA; Brazil (Takemoto et al. 1996a, b; Luque and Poulin 2004).

*Opisthonema oglinum* (Actinopterygii: Clupeidae); marine; body cavity; metacestode; TSA; Brazil (Palm 1997).

*Pagrus pagrus* (Actinopterygii: Sparidae); marine; body cavity; metacestode; WTSA; Brazil (Palm 2004).

*Paralichthys isosceles* (Actinopterygii: Paralichthyidae); marine; muscles; metacestode; WTSA; Brazil (Felizardo et al. 2010).

*Paralichthys patagonicus* (Actinopterygii: Paralichthyidae); marine; body cavity, kidney, mesentery, spleen; metacestode; WTSA; Brazil (Fonseca et al. 2012).

*Paralonchurus peruanus* (Actinopterygii: Sciaenidae); marine; surface of internal organs, serous membrane; metacestode; WTSP; Peru (Escalante and Carvajal 1984).

Note: host reported as *Polyclemus peruanus*.

*Percophis brasiliensis* (Actinopterygii: Percophidae); marine; mesentery; metacestode; WTSA; Argentina, Uruguay (Braicovich and Timi 2008, 2010).

*Pinguipes brasilianus* (Actinopterygii: Pinguipedidae); marine; mesentery; metacestode; WTSA; Brazil (Timi et al. 2010a).

*Plagioscion squamosissimus* (Actinopterygii: Sciaenidae); freshwater; muscles; metacestode; Amazon River basin; Brazil (Silva 2010).

*Pomatomus saltatrix* (Actinopterygii: Pomatomidae); marine; body cavity, mesentery, peritoneum; metacestode; WTSA; Brazil (Carvajal and Rego 1985; Carvajal et al. 1987; São Clemente et al. 1997; Palm 2004, Ferreira et al. 2006).

Note: host reported as *Pomatomus saltator* by some authors.

*Prionace glauca* (Elasmobranchii: Carcharhinidae); marine; spiral valve; metacestode; WTSA; Brazil (Knoff et al. 2002; Pinto et al. 2006).

*Pseudopercis numida* (Actinopterygii: Pinguipedidae); marine; mesentery; metacestode; WTSA; Brazil (Luque et al. 2008).

*Sardinella brasiliensis* (Actinopterygii: Clupeidae); marine; body cavity; metacestode; WTSA; Brazil (Moreira et al. 2015).

*Sciaena deliciosa* (Actinopterygii: Sciaenidae); marine; surface of internal organs, serous membrane; metacestode; WTSP; Peru (Escalante and Carvajal 1984).

*Scomberomorus brasiliensis* (Actinopterygii: Scombridae); marine; body cavity; metacestode; TSA; Brazil (Palm 1997).

Note: host reported as *Serrasalmus maculatus*.

*Scomberomorus cavalla* (Actinopterygii: Scombridae); marine; mesentery; metacestode; WTSA; Brazil (Dias et al. 2011a).

*Selene setapinnis* (Actinopterygii: Carangidae); marine; mesentery; metacestode; WTSA; Brazil (Cordeiro and Luque 2004; Luque and Poulin 2004).

*Selene vomer* (Actinopterygii: Carangidae); marine; body cavity; metacestode; TSA; Brazil (Palm 1997).

*Sphyraena guachancho* (Actinopterygii: Sphyraenidae); marine; body cavity; metacestode; TSA; Brazil (Palm 1997).

*Trachurus lathami* (Actinopterygii: Carangidae); marine; mesentery; metacestode; WTSA; Argentina, Brazil (Braicovich et al. 2012).

*Trichiurus lepturus* (Actinopterygii: Trichiuridae); marine; body cavity, mesentery, stomach; WTSA; Brazil (Silva et al. 2000a, b; Carvalho and Luque 2011; Bueno et al. 2014).

*Umbrina canosai* (Actinopterygii: Sciaenidae); marine; site of infection not given; metacestode; WTSA; Brazil (Luque and Poulin 2004).

*Urophycis brasiliensis* (Actinopterygii: Phycidae); marine; site of infection not given; metacestode; WTSA; Argentina (Pereira et al. 2014).

***Callitetrarhynchus speciosus* (Linton, 1897) Carvajal & Rego, 1985**

[Syns. *Rhynchobothrium speciosum* Linton, 1897; *Tentacularia pseudodera* Schuler, 1938]

*Aluterus monoceros* (Actinopterygii: Monacanthidae); marine; liver, mesentery; metacestode; WTSA; Brazil (Dias et al. 2010).

*Balistes capriscus* (Actinopterygii: Balistidae); marine; mesentery; metacestode; WTSA; Brazil (Luque and Poulin 2004; Dias et al. 2009).

*Balistes vetula* (Actinopterygii: Balistidae); marine; body cavity, mesentery, muscles; metacestode; WTSA; Brazil (São Clemente et al. 1995; Luque and Poulin 2004).

*Cynoscion guatucupa* (Actinopterygii: Sciaenidae); marine; mesentery; metacestode; WTSA; Brazil (Pereira and Boeger 2005; Pinto et al. 2006).

*Genidens barbus* (Actinopterygii: Ariidae); marine; body cavity, viscera; metacestode; WTSA; Brazil (São Clemente et al. 1991a).

Note: host reported as *Netuma barba*.

*Micropogonias furnieri* (Actinopterygii: Sciaenidae); marine; mesentery; metacestode; WTSA; Brazil (Pereira 1993; Pereira and Boeger 2005; Pinto et al. 2006).

*Pomatomus saltatrix* (Actinopterygii: Pomatomidae); marine; body cavity, mesentery; metacestode; WTSA; Brazil (Carvajal and Rego 1985; São Clemente et al. 1997; Ferreira et al. 2006).

Note: type host; it was reported as *Pomatomus saltator* by some authors.

*Priacanthus arenatus* (Actinopterygii: Priacanthidae); marine; mesentery, serosa of intestine and ovary; metacestode; WTSA; Brazil (Kuraiem et al. 2016).

*Scomberomorus cavalla* (Actinopterygii: Scombridae); marine; mesentery; metacestode; WTSA; Brazil (Dias et al. 2011a).

*Sphyrna zygaena* (Elasmobranchii: Sphyrnidae); marine; spiral valve; metacestode; WTSA; Brazil (Knoff et al. 2002; Pinto et al. 2006).

*Stephanolepis hispidus* (Actinopterygii: Monacanthidae); marine; mesentery; metacestode; WTSA; Brazil (Palm 2004).

***Callitetrarhynchus* sp.**

*Balistes capriscus* (Actinopterygii: Balistidae); marine; mesentery; metacestode; WTSA; Brazil (Alves et al. 2005).

*Balistes vetula* (Actinopterygii: Balistidae); marine; body cavity, mesentery, muscles; metacestode; WTSA; Brazil (Alves et al. 2005).

*Conodon nobilis* (Actinopterygii: Haemulidae); marine; body cavity; metacestode; WTSA; Brazil (Paschoal et al. 2015).

***Dasyrhynchus giganteus* (Diesing, 1850) Pintner, 1929**

[Syns. *Anthocephalus giganteus* Diesing, 1850; *Rhynchobothrium insigne* Linton, 1924; *Sbesterium insigne* Dollfus, 1929]

*Caranx hippos* (Actinopterygii: Carangidae); marine; brain; metacestode; NBS, TSA; Brazil (São Clemente et al. 1993; Palm 1997).

*Oligoplites saliens* (Actinopterygii: Carangidae); marine; subcutaneous; metacestode; NBS (estuary of Tapajós River); Brazil (Diesing 1850, 1856).

Notes: type host; it was originally reported as *Chorinemus saliens*. Beveridge and Campbell (1993) redescribed this species.

***Dasyrhynchus pacificus* Robinson, 1965**

*Carcharhinus brachyurus* (Elasmobranchii: Carcharhinidae); marine; spiral valve; adult; WTSA; Brazil (São Clemente and Gomes 1989a; São Clemente et al. 1991b).

*Carcharhinus limbatus* (Elasmobranchii: Carcharhinidae); marine; spiral valve; adult; WTSA; Brazil (Beveridge and Campbell 1993; Palm 2004).

*Cynoscion guatucupa* (Actinopterygii: Sciaenidae); marine; body cavity, haemal

arches, mesentery, kidney; metacestode; WTSA; Argentina, Brazil, Uruguay (Pereira and Boeger 2005; Timi et al. 2005, 2010b).

*Cynoscion jamaicensis* (Actinopterygii: Sciaenidae); marine; mesentery; metacestode; WTSA; Brazil (Pereira and Boeger 2005).

*Macrodon ancylodon* (Actinopterygii: Sciaenidae); marine; body cavity, pericardium, kidney; metacestode; WTSA; Brazil (Pereira and Boeger 2005).

*Menticirrhus americanus* (Actinopterygii: Sciaenidae); marine; body cavity, mesentery, kidney; metacestode; WTSA; Brazil (Pereira and Boeger 2005).

*Sciaena deliciosa* (Actinopterygii: Sciaenidae); marine; peritoneum; metacestode; WTSP; Peru (Escalante and Carvajal 1984).

*Scyliorhinus haeckelii* (Elasmobranchii: Scyliorhinidae); marine; spiral valve; adult; WTSA; Brazil (Beveridge and Campbell 1993; Palm 2004).

*Sphyrna* sp. (Elasmobranchii: Sphyrnidae); marine; spiral valve; adult; WTSA; Brazil (Beveridge and Campbell 1993; Palm 2004).

*Floriceps saccatus* Cuvier, 1817*

[Syns. *Anthocephalus elongatus* Rudolphi, 1819; *Rhynchobothrium ingens* Linton, 1921; *Rhynchobothrium carangis* MacCallum, 1921; *Floriceps caballeroi* Ceuz-Reyes, 1977]

*Aluterus monoceros* (Actinopterygii: Monacanthidae); marine; liver, mesentery; metacestode; WTSA; Brazil (Dias et al. 2010).

*Centropomus nigrescens* (Actinopterygii: Centropomidae); amphidromous; peritoneum; metacestode; WTSP; Peru (Escalante and Carvajal 1984).

*Coryphaena hippurus* (Actinopterygii: Coryphaenidae); marine; muscles; metacestode; WTSA; Brazil (Silva and São Clemente 2001).

*Prionace glauca* (Elasmobranchii: Carcharhinidae); marine; spiral valve; adult; WTSA; Brazil (Knoff et al. 2002; Pinto et al. 2006).

*Seriola lalandi* (Actinopterygii: Carangidae); marine; muscle; metacestode; WTSP; Chile (Soto and Carvajal 1979).

Note: host reported as *Seriola mazatlana*.

**Grillotia (Christianella) carvajalregorum Menoret & Ivanov, 2009**

[Syns. *Progrillotia dollfusi* Carvajal & Rego 1983; Grillotia (Progrillotia) dollfusi (Carvajal & Rego, 1983) Palm, 2004; *Grillotia carvajalregorum* Menoret & Ivanov, 2009]

*Acanthistius brasilianus* (Actinopterygii: Serranidae); marine; mesentery; metacestode; WTSA; Argentina (Menoret and Ivanov 2009b).

*Carcharhinus signatus* (Elasmobranchii: Carcharhinidae); marine; stomach; adult; WTSA; Brazil (Knoff et al. 2002, 2004c).

*Conger orbignianus* (Actinopterygii: Congridae); marine; intestinal surface, mesentery; metacestode; WTSA; Argentina (Timi and Lanfranchi 2013).

*Ctenosciaena gracilicirrhus* (Actinopterygii: Sciaenidae); marine; body cavity; metacestode; WTSA; Brazil (Pereira and Boeger 2005).

*Cynoscion guatucupa* (Actinopterygii: Sciaenidae); marine; body cavity, mesentery; metacestode; WTSA; Argentina, Brazil (Sabas and Luque 2003; Luque and Poulin 2004; Pereira and Boeger 2005; Timi et al. 2005, 2010b; Menoret and Ivanov 2009b).

*Cynoscion jamaicensis* (Actinopterygii: Sciaenidae); marine; body cavity; metacestode; WTSA; Brazil (Pereira and Boeger 2005).

*Cynoscion striatus* (Actinopterygii: Sciaenidae); marine; body cavity; metacestode; WTSA; Brazil (Rego et al. 1974; Carvajal and Rego 1983).

Notes: type host. Carvaval and Rego (1983) re-identified the larvae collected by Rego et al. (1974).

*Dules auriga* (Actinopterygii: Serranidae); marine; mesentery; metacestode; WTSA; Argentina (Menoret and Ivanov 2012; Braicovich and Timi 2015).

Note: Menoret and Ivanov (2012) reported the host as *Serranus auriga* (Cuvier).

*Genypterus brasiliensis* (Actinopterygii: Ophidiidae); marine; mesentery; metacestode; WTSA; Brazil (Alves et al. 2002a, b; Luque and Poulin 2004; São Clemente et al. 2004).

Note: after morphological re-evalution of specimens deposited by Alves et al. (2002b), São Clemente et al. (2004) assigned this species to *Progrillotia dollfusi* (syn. of Grillotia (Christianella) carvajalregorum).

*Heptranchias perlo* (Elasmobranchii: Hexanchidae); marine; spiral valve; metacestode; WTSA; Brazil (Knoff et al. 2002, 2004c).

*Lophius gastrophysus* (Actinopterygii: Lophiidae); marine; body cavity; metacestode; WTSA; Brazil (São Clemente et al. 2007).

*Macrodon ancylodon* (Actinopterygii: Sciaenidae); marine; mesentery, body

cavity; metacestode; WTSA; Brazil (Sabas and Luque 2003; Luque and Poulin 2004; Pereira and Boeger 2005).

*Menticirrhus americanus* (Actinopterygii: Sciaenidae); marine; body cavity; metacestode; WTSA; Brazil (Pereira and Boeger 2005).

*Menticirrhus littoralis* (Actinopterygii: Sciaenidae); marine; body cavity; metacestode; WTSA; Brazil (Pereira and Boeger 2005).

*Merluccius hubbsi* (Actinopterygii: Merlucciidae); marine; mesentery; metacestode; Magellanic; Argentina (Menoret and Ivanov 2012).

*Micropogonias furnieri* (Actinopterygii: Sciaenidae); marine; mesentery; metacestode; WTSA; Argentina, Brazil (Pereira and Boeger 2005; Menoret and Ivanov 2009b).

*Nemadactylus bergi* (Actinopterygii: Cheilodactylidae); marine; mesentery; metacestode; WTSA; Argentina (Menoret and Ivanov 2009b; Rossin and Timi 2010).

*Paralichthys isosceles* (Actinopterygii: Paralichthyidae); marine; body cavity, liver, mesentery, muscles; metacestode; WTSA; Argentina, Brazil (Felizardo et al. 2010; Alarcos and Timi 2012; Alarcos et al. 2016).

*Paralichthys patagonicus* (Actinopterygii: Paralichthyidae); marine; body cavity, mesentery; metacestode; WTSA; Argentina, Brazil (Alarcos and Timi 2012; Fonseca et al. 2012).

*Paralonchurus brasiliensis* (Actinopterygii: Sciaenidae); marine; body cavity; metacestode; WTSA; Brazil (Pereira and Boeger 2005).

*Parona signata* (Actinopterygii: Carangidae); marine; mesentery; metacestode; WTSA; Argentina (Menoret and Ivanov 2012).

*Percophis brasiliensis* (Actinopterygii: Percophidae); marine; mesentery; metacestode; WTSA; Argentina (Menoret and Ivanov 2009b; Braicovich and Timi 2010).

*Pomatomus saltatrix* (Actinopterygii: Pomatomidae); marine; mesentery; metacestode; WTSA; Argentina (Menoret and Ivanov 2012).

*Porichthys porosissimus* (Actinopterygii: Batrachoididae); marine; mesentery; metacestode; WTSA; Argentina (Menoret and Ivanov 2012).

*Prionotus nudigula* (Actinopterygii: Triglidae); marine; mesentery; metacestode; Magellanic; Argentina (Menoret and Ivanov 2012).

*Prionotus punctatus* (Actinopterygii: Triglidae); marine; intestinal surface, mesentery; metacestode; WTSA; Argentina, Brazil (Bicudo et al. 2005; Menoret and Ivanov 2009b).

*Pseudopercis numida* (Actinopterygii: Pinguipedidae); marine; mesentery; metacestode; WTSA; Brazil (Luque et al. 2008).

*Pseudopercis semifasciata* (Actinopterygii: Pinguipedidae); marine; mesentery; metacestode; WTSA; Brazil (Luque et al. 2008).

*Raneya brasiliensis* (Actinopterygii: Ophidiidae); marine; mesentery; metacestode; Magellanic, WTSA; Argentina (Vales et al. 2011).

*Squalus* sp. (Elasmobranchii: Squalidae); marine; spiral valve; adult; WTSA; Brazil (Knoff et al. 2002, 2004c).

*Squatina guggenheim* (Elasmobranchii: Squatinidae); marine; spiral valve; adult; WTSA; Argentina (Menoret and Ivanov 2009b).

Note: Menoret and Ivanov (2009b) provided a new name for *Progrillotia dollfusi* and a detailed description of adult forms.

*Trachurus lathami* (Actinopterygii: Carangidae); marine; mesentery; metacestode; WTSA; Argentina, Brazil (Menoret and Ivanov 2009b; Braicovich et al. 2012).

*Umbrina canosai* (Actinopterygii: Sciaenidae); marine; body cavity, mesentery; metacestode; WTSA; Argentina, Brazil (Pereira and Boeger 2005; Menoret and Ivanov 2009b).

*Urophycis brasiliensis* (Actinopterygii: Phycidae); marine; mesentery; metacestode; WTSA; Argentina (Menoret and Ivanov 2009b; Pereira et al. 2014).

*Xystreurys rasile* (Actinopterygii: Paralichthyidae); marine; body cavity, mesentery; metacestode; WTSA; Argentina, Brazil (Menoret and Ivanov 2009b; Alarcos and Timi 2012, 2013; Fonseca et al. 2012).

Grillotia (Christianella) minuta (van Beneden, 1849) Guiart, 1931*

[Syns. *Tetrarhynchus minutus* van Beneden, 1849; *Grillotia minuta* (van Beneden, 1849); *Tetrarhynchus smarisgora* Wagener, 1854; *Tetrarhynchus smarismaenae* Wagener, 1854; *Tetrarhynchus smaridum* Pintner, 1893; *Grillotia angeli* Dollfus, 1969; *Grillotia bothridiopunctata* Dollfus, 1969]

*Cynoscion guatucupa* (Actinopterygii: Sciaenidae); marine; mesentery; metacestode; WTSA; Argentina, Uruguay (Timi et al. 2005).

Notes: tapeworms reported as *Grillotia bothridiopunctata*. Grillotia (Christianella) minuta is distributed across the Northeastern Atlantic and morphologically similar to Grillotia (Christianella) carvajalregorum; therefore, its occurrence in the Southwestern Atlantic is doubtful (Menoret and Ivanov 2012).

**Grillotia (Grillotia) borealis Keeney & Campbell, 2001**

*Macrourus carinatus* (Actinopterygii: Macrouridae); marine; mesentery; metacestode; Magellanic; Argentina (Palm 2004).

Note: this species was described from fishes in the North Pacific Ocean and it is morphologically similar to *Grillotia patagonica* Menoret and Ivanov, 2012; thus, its occurrence in the Southwestern Atlantic is doubtful (Menoret and Ivanov 2012).

*Salilota australis* (Actinopterygii: Moridae); marine; mesentery; metacestode; Magellanic; Argentina (Palm 2004).

**Grillotia (Grillotia) dollfusi Carvajal, 1971**

*Dipturus flavirostris* (Elasmobranchii: Rajidae); marine; spiral valve; adult; WTSP; Chile (Leible et al. 1990).

Note: host reported as *Raja flavirostris*.

*Macruronus magellanicus* (Actinopterygii: Merlucciidae); marine; body cavity; metacestode; WTSP; Chile (Oliva 2001).

*Merluccius gayi gayi* (Actinopterygii: Merlucciidae); marine; body cavity, intestinal serosa, gonads, liver, stomach wall; metacestode; WTSP; Chile (Carvajal and Cattan 1978; Carvajal et al. 1979; George-Nascimento 1996; Oliva and Ballón 2002).

*Merluccius gayi peruanus* (Actinopterygii: Merlucciidae); marine; mesentery; metacestode; WTSP; Peru (Chero et al. 2014a).

*Zearaja chilensis* (Elasmobrachii: Rajidae); marine; spiral valve; adult; WTSP; Chile (Carvajal 1971; Whittaker et al. 1982).

Notes: type host; it was reported as *Raja chilensis*. Beveridge and Campbell (2007) redescribed *Diphyllobothrium dollfusi* based on the type-specimens.

**Grillotia (Grillotia) erinaceus (van Beneden, 1858) Guiart, 1927**

[Syns. *Tetrarhynchus erinaceus* van Beneden, 1858; *Rhynchobothrium imparispine* Linton, 1897; *Grillotia pseuderinaceus* Dollfus, 1969; *Grillotia recurvispinis* Dollfus, 1969]

*Conger orbignianus* (Actinopterygii: Congridae); marine; mesentery; metacestode; WTSA; Argentina (Tanzola and Guagliardo 2000).

*Dissostichus eleginoides* (Actinopterygii: Nototheniidae); marine; mesentery, stomach, intestine wall; metacestode; Magellanic; Falkland Islands (Brickle et al. 2006; Brown et al. 2013).

*Eleginops maclovinus* (Actinopterygii: Eleginopsidae); marine; mesentery; metacestode; Magellanic; Falkland Islands (Brickle and MacKenzie 2007).

*Porichthys porosissimus* (Actinopterygii: Batrachoididae); marine; mesentery; metacestode; WTSA; Argentina (Tanzola et al. 1997).

Note: Menoret and Ivanov (2012) dissected 7 specimens of this fish host, also from the Argentine Sea, and they only found Grillotia (Christianella) carvajalregorum; therefore, they questioned the reliability of this report.

*Sympterygia bonapartii* (Elasmobranchii: Arhynchobatidae); marine; spiral valve; adult; WTSA; Argentina (Tanzola et al. 1998).

Note: Menoret and Ivanov (2012) dissected 32 specimens of this fish host, also from the Argentine Sea, and they did not find any *Grillotia erinaceus*; therefore, they questioned the reliability of this report.

**Grillotia (Grillotia) patagonica Menoret & Ivanov, 2012**

*Cottoperca gobio* (Actinopterygii: Bovichtidae); marine; body cavity; metacestode; Magellanic; Argentina (Menoret and Ivanov 2012).

*Nemadactylus bergi* (Actinopterygii: Cheilodactylidae); marine; mesentery; metacestode; Magellanic; Argentina (Menoret and Ivanov 2012).

*Patagonotothen brevicauda brevicauda* (Actinopterygii: Nototheniidae); marine; mesentery; metacestode; Magellanic; Argentina (Menoret and Ivanov 2012).

*Patagonotothen ramsayi* (Actinopterygii: Nototheniidae); marine; mesentery; metacestode; Magellanic; Argentina (Menoret and Ivanov 2012).

*Psammobatis rudis* (Elasmobranchii: Arhynchobatidae); marine; spiral valve; adult; Magellanic; Argentina (Menoret and Ivanov 2012).

Note: type host.

*Salilota australis* (Actinopterygii: Moridae); marine; mesentery; metacestode; Magellanic; Argentina (Menoret and Ivanov 2012).

***Grillotia heptanchi* (Vaullegeard, 1899) Dollfus, 1942** (*Grillotia* sensu lato)

[For synonyms, see Beveridge and Campbell (2013)]

*Genypterus chilensis* (Actinopterygii: Ophidiidae); marine; muscles; metacestode; Magellanic; Chile (Carvajal and Campbell 1979).

*Hexanchus griseus* (Elasmobranchii: Hexanchidae); marine; spiral valve; adult; WTSP; Chile (Carvajal 1971, 1974).

*Macruronus magellanicus* (Actinopterygii: Merlucciidae); marine; body cavity, viscera; metacestode; Magellanic; Chile (Carvajal and Campbell 1979; Torres et al. 1993).

*Merluccius australis* (Actinopterygii: Merlucciidae); marine; mesentery, muscles; metacestode; Magellanic; Chile (Carvajal and Campbell 1979; Fernández 1985; George-Nascimento and Arancibia 1994; González and Carvajal 1994; Chávez et al. 2012).

Note: Carvajal and Campbell (1979) reported the host as *Merluccius polylepis*.

*Merluccius gayi gayi* (Actinopterygii: Merlucciidae); marine; muscles; metacestode; WTSP; Chile (Tagle 1951).

***Grillotia* sp.**

*Aphos porosus* (Actinopterygii: Batrachoididae); marine; body cavity; metacestode; WTSP; Chile (Cortés and Muñoz 2008, 2009).

*Bathyraja magellanica* (Elasmobranchii: Arhynchobatidae); marine; spiral valve; adult; Magellanic; Argentina (Menoret and Ivanov 2012).

Note: the specimens are most likely Grillotia (Grillotia) patagonica, but the internal morphology could not be assessed in frozen material, which prevented the precise identification (Menoret and Ivanov 2012).

*Coelorinchus chilensis* (Actinopterygii: Macrouridae); marine; stomach; metacestode; JFD; Chile (Pardo-Gandarilhas et al. 2008).

*Eleginops maclovinus* (Actinopterygii: Eleginopsidae); marine; site of infection not given; metacestode; WTSP; Chile (Henríquez et al. 2011).

*Lutjanus analis* (Actinopterygii: Lutjanidae); marine; body cavity; metacestode; TSA; Brazil (Palm 1997).

Note: Palm (2004) suggested the genus *Pseudolacistorhynchus* Palm, 1995 as the correct generic identification.

*Merluccius australis* (Actinopterygii: Merlucciidae); marine; body cavity, mesentery; metacestode; Magellanic; Falkland Islands (MacKenzie and Longshaw 1995).

*Merluccius hubbsi* (Actinopterygii: Merlucciidae); marine; body cavity; metacestode; Magellanic, WTSA; Argentina, Falkland Islands, Uruguay (MacKenzie and Longshaw 1995; Sardella and Timi 1996, 2004).

*Micromesistius australis australis* (Actinopterygii: Gadidae); marine; site of infection not given; metacestode; Magellanic; Argentina, Chile, Falkland Islands (Niklitschek et al. 2010; George-Nascimento et al. 2011).

*Paralabrax humeralis* (Actinopterygii: Serranidae); marine; stomach; metacestode; WTSP; Peru (Armas 1983).

*Paralichthys orbignyanus* (Actinopterygii: Paralichthyidae); marine; site of infection not given; metacestode; WTSA (Mar Chiquita coastal lagoon); Argentina (Alarcos and Etchegoin 2010).

*Percophis brasiliensis* (Actinopterygii: Percophidae); marine; mesentery; metacestode; WTSA; Argentina, Brazil, Uruguay (Luque and Poulin 2004; Braicovich and Timi 2008).

*Pinguipes brasilianus* (Actinopterygii: Pinguipedidae); marine; mesentery; metacestode; Magellanic, WTSA; Argentina, Brazil (Timi et al. 2008, 2009, 2010a).

*Prionotus nudigula* (Actinopterygii: Triglidae); marine; mesentery; metacestode; WTSA; Argentina (Timi and Lanfranchi 2009b).

*Pseudopercis semifasciata* (Actinopterygii: Pinguipedidae); marine; mesentery; metacestode; Magellanic, WTSA; Argentina (Timi and Lanfranchi 2009a).

*Squatina armata* (Elasmobranchii: Squatinidae); marine; spiral valve; adult; WTSP; Peru (Tresierra et al. 1986).

***Lacistorhynchus dollfusi* Beveridge & Sakanari, 1987**

[Syn. *Lacistorhynchus tenuis* (van Beneden, 1858) (*pro parte*)]

*Cheilotrema fasciatum* (Actinopterygii: Sciaenidae); marine; body cavity; metacestode; WTSP; Peru (Oliva and Luque 1998).

Note: host reported as *Sciaena fasciata*.

*Paralichthys adspersus* (Actinopterygii: Paralichthyidae); marine; body cavity, muscles; metacestode; WTSP; Chile (Oliva et al. 1996).

*Lacistorhynchus tenuis* (van Beneden, 1858) Pintner, 1913*

[Syns. *Tetrarhynchus tenuis* van Beneden, 1858; *Rhynchobothrium heterospine* Linton, 1897; *Rhynchobothrium bulbifer* Linton, 1897]

*Anisotremus scapularis* (Actinopterygii: Haemulidae); marine; mesentery, muscles; metacestode; WTSP; Peru (Luque 1991).

Note: Beveridge and Sakanari (1987) suggested that this apparently cosmopolitan taxon might represent a composite of two or more species and its distribution in the Pacific waters needs confirmation. Therefore, a taxonomic reassessment of *Lacistorhynchus tenuis* is pending.

*Cheilodactylus variegatus* (Actinopterygii: Cheilodactylidae); marine; mesentery, muscles; metacestode; WTSP; Peru (Luque 1991).

*Cheilotrema fasciatum* (Actinopterygii: Sciaenidae); marine; body cavity; metacestode; WTSP; Peru (Luque 1991)

Note: host reported as *Sciaena fasciata*.

*Cilus gilberti* (Actinopterygii: Sciaenidae); marine; site of infection not given; metacestode; WTSP; Chile (Garcías et al. 2001).

*Labrisomus philippii* (Actinopterygii: Labrisomidae); marine; mesentery, muscles; metacestode; WTSP; Peru (Rivera and Sarmiento 1990; Oliva and Luque 2002; Cruces et al. 2015).

*Merluccius gayi peruanus* (Actinopterygii: Merlucciidae); marine; mesentery; metacestode; WTSP; Peru (Durán and Oliva 1980; Jara 1998).

*Mugil cephalus* (Actinopterygii: Mugilidae); marine; mesentery, muscles; metacestode; WTSP; Peru (Luque 1991).

*Odontesthes regia* (Actinopterygii: Atherinopsidae); marine; muscles; metacestode; WTSP; Peru (Escalante and Carvajal 1984).

*Paralichthys patagonicus* (Actinopterygii: Paralichthyidae); marine; body cavity; metacestode; WTSA; Argentina (Szidat 1961).

*Triakis maculata* (Elasmobranchii: Triakidae); marine; spiral valve; adult; WTSP; Chile (Carvajal 1974).

***Lacistorhynchus* sp.**

*Merluccius australis* (Actinopterygii: Merlucciidae); marine; muscles; metacestode; Magellanic; Chile, Falkland Island (MacKenzie and Longshaw 1995).

*Scartichthys viridis* (Actinopterygii: Bleniidae); marine; site of infection not

given; metacestode; WTSP; Chile (Flores and George-Nascimento 2009).

*Urophycis brasiliensis* (Actinopterygii: Phycidae); marine; surface of pyloric

caeca; metacestode; WTSA; Argentina (Szidat 1961).

*Urophycis mystaceus* (Actinopterygii: Phycidae); marine; mesentery; metacestode; WTSA; Brazil (Alves et al. 2002c; Luque and Poulin 2004).

***Paragrillotia* sp.**

*Dipturus trachyderma* (Elasmobranchii: Rajidae); marine; site of infection and

stage of development not given; WTSP; Chile (Leible et al. 1990).

Notes: host reported as *Raja trachyderma*. Neither were the three known species of *Paragrillotia* Dollfus, 1969 described from rays nor reported from Southeastern Pacific (see Beveridge and Justine 2007a). Since the vouchers were apparently not deposited, we considered this record doubtful.

***Pseudogrillotia peruviana* Escalante & Carvajal, 1984**

*Scomberomorus sierra* (Actinopterygii: Scombridae); marine; mesentery; metacestode; WTSP; Peru (Escalante and Carvajal 1984).

Note: type host; it was reported as *Serrasalmus maculatus*.

***Pseudogrillotia* sp.**

*Pomatomus saltatrix* (Actinopterygii: Pomatomidae); marine; mesentery; metacestode; WTSA; Brazil (Palm 2004).

*Pseudolacistorhynchus noodti* Palm, 1995*

[Syn. *Rhynchobothrium* sp. of Linton 1909, 1924]

*Pseudupeneus maculatus* (Actinopterygii: Mullidae); marine; body cavity; metacestode; TSA; Brazil (Palm 1997).

Note: type host.

*Scomberomorus brasiliensis* (Actinopterygii: Scombridae); marine; body cavity; metacestode; TSA; Brazil (Palm 1997).

Note: host reported as *Serrasalmus maculatus*.

####### Family Pterobothriidae Pintner, 1931

***Pterobothrium acanthotruncatum* Escalante & Carvajal, 1984**

[Syns. *Synbothrium hemuloni* MacCallum, 1921; *Gymnorhynchus gigas* (Cuvier, 1817) sensu Southwell (1929); *Pterobothrium heteracanthum* Diesing, 1850 sensu Palm (1995)]

*Coryphaena hippurus* (Actinopterygii: Coryphaenidae); marine; gallbladder, mesentery; metacestode; WTSP; Peru (Escalante and Carvajal 1984).

Note: type host.

*Micropogonias furnieri* (Actinopterygii: Sciaenidae); marine; body cavity; metacestode; WTSA; Brazil (Palm 2004).

*Paralonchurus peruanus* (Actinopterygii: Sciaenidae); marine; gallbladder, gonads, mesentery, peritoneum; metacestode; WTSP; Peru (Tantaleán and Huiza 1994).

***Pterobothrium crassicole* Diesing, 1850**

[Syns. *Synbothrium felis* of MacCallum – USNMHC 35978; *Synbothrium* sp. of MacCallum – USNMHC 35744]

*Aspistor luniscutis* (Actinopterygii: Ariidae); marine; body cavity; metacestode; WTSA; Brazil (Tavares and Luque 2008).

*Bagre marinus* (Actinopterygii: Ariidae); marine; body cavity; metacestode; NBS (estuary of Amazon River); Brazil (Rego 1987a).

Note: host reported as *Bagrus marinus*.

*Brachyplatystoma rousseauxii* (Actinopterygii: Pimelodidae); freshwater; body cavity; metacestode; Amazon River basin (estuary); Brazil (Rego 1987a).

Note: host reported as *Brachyplatystoma flavicans*.

*Brachyplatystoma vaillantii* (Actinopterygii: Pimelodidae); freshwater; body cavity; metacestode; Amazon River basin (estuary); Brazil (Rego 1987a).

*Citharichthys spilopterus* (Actinopterygii: Paralichthyidae); marine; muscles; metacestode; TSA; Brazil (Palm 2004).

*Cynoscion acoupa* (Actinopterygii: Sciaenidae); marine; muscles; metacestode; NBS; Brazil (Dias et al. 2011b).

*Cynoscion leiachus* (Actinopterygii: Sciaenidae); marine; body cavity; metacestode; WTSA; Brazil (Rego et al. 1974; Palm 2004).

Note: Rego et al. (1974) reported this species as *Pterobohrium* sp., but Pereira (1993) considered them to be *Pterobothrium crassicole*.

*Cynoscion* sp. (Actinopterygii: Sciaenidae); marine; site of infection not given; metacestode; WTSA; Brazil (Ferreira et al. 2006).

*Genidens barbus* (Actinopterygii: Ariidae); marine; muscles; metacestode; WTSA; Brazil (São Clemente et al. 1991a).

Note: host reported as *Netuma barba*.

*Gobioides broussonnetii* (Actinopterygii: Gobiidae); brackish; mesentery; metacestode; Amazon River basin (estuary, Marajó Island); Brazil (Videira et al. 2013).

*Micropogonias furnieri* (Actinopterygii: Sciaenidae); marine; body cavity, liver, mesentery, peritoneum; metacestode; WTSA; Brazil (São Clemente 1986a, b, 1987; Pereira 1993; Campbell and Beveridge 1996; Pereira and Boeger 2005; Porto et al. 2009).

*Micropogonias undulatus* (Actinopterygii: Sciaenidae); marine; mesentery; metacestode; WTSA; Brazil (Palm 2004).

Note: The host is assigned to *Tachysurus* sp. in the CHIOC database.

*Oligoplites palometa* (Actinopterygii: Carangidae); marine; body cavity; metacestode; WTSA; Brazil (Takemoto et al. 1996a, b; Luque and Poulin 2004).

Note: Palm (2004) re-examined the specimens deposited in CHIOC and considered them as *Pterobothrium* sp.

*Paralichthys isosceles* (Actinopterygii: Paralichthyidae); marine; stomach serosa; metacestode; WTSA; Brazil (Felizardo et al. 2010).

*Paralichthys patagonicus* (Actinopterygii: Paralichthyidae); marine; body cavity, kidney, liver, mesentery, muscles, stomach serosa; metacestode; WTSA; Brazil (Fonseca et al. 2012).

*Pimelodus* sp. (Actinopterygii: Pimelodidae); freshwater; mesentery; metacestode; Amazon River basin; Brazil (Diesing 1850, 1856).

Notes: type host. Diesing (1850) reported the specimens from *Erythrinus unitaeniatus* (syn. of *Hoplerythrinus unitaeniatus*), but it was re-identified by Diesing (1856). The Diesing’s types deposited in NHMW consist only of cestode fragments (see the redescription of Campbell and Beveridge 1996).

*Plagioscion squamosissimus* (Actinopterygii: Sciaenidae); freshwater; muscles; metacestode; Amazon River Basin (estuary); Brazil (Silva 2010).

*Pogonias cromis* (Actinopterygii: Sciaenidae); marine; body cavity; metacestode; WTSA; Brazil (Pereira and Boeger 2005).

*Pomatomus saltatrix* (Actinopterygii: Pomatomidae); marine; mesentery; metacestode; WTSA; Brazil (São Clemente et al. 1997).

*Scomberomorus cavalla* (Actinopterygii: Scombridae); marine; mesentery; metacestode; WTSA; Brazil (Dias et al. 2011a).

*Scorpaena* sp. (Actinopterygii: Scorpaenidae); marine; mesentery; metacestode; WTSA; Brazil (Palm 2004).

***Pterobothrium heteracanthum* Diesing, 1850**

[Syns. *Syndesmobothrium filicolle* Linton, 1890; *Synbothrium fillicolle* Linton, 1897; *Synbothrium hemuloni* MacCallum, 1921; *Gymnorhynchus cymbiumi* Chincholikar & Shinde, 1977; *Neogymnorhynchus platycephali* Bilqees & Shah, 1982]

*Cynoscion acoupa* (Actinopterygii: Sciaenidae); marine; muscles; metacestode; NBS; Brazil (Dias et al. 2011b).

*Cynoscion guatucupa* (Actinopterygii: Sciaenidae); marine; body cavity; metacestode; WTSA; Brazil (Pereira and Boeger 2005).

*Micropogonias furnieri* (Actinopterygii: Sciaenidae); marine; body cavity, mesentery, peritoneum; metacestode; TSA, WTSA; Argentina, Brazil (São Clemente 1986a, b, 1987; Pereira 1993; Campbell and Beveridge 1996; Alves and Luque 2000, 2001a, b; Luque and Poulin 2004; Pereira and Boeger 2005; Alarcos and Etchegoin 2010; Luque et al. 2010; Timi et al. 2010b; Mattos et al. 2015).

Note: Campbell and Beveridge (1996) studied specimens collected by São Clemente in 1980 that are deposited in CHIOC; they suggested that these worms are 'topotypes', i.e. specimens that do not belong to the type series, but were collected in the type locality (this term is not considered by the ICZN), even though there is no evidence that J. Natterer collected the worms in the Rio de Janeiro coast as stated by São Clemente.

*Micropogonias undulatus* (Actinopterygii: Sciaenidae); marine; gallbladder surface, intestine, mesentery; metacestode; NBS (Amazon River estuary), WTSA; Brazil, Uruguay (Diesing 1850, Palm 2004).

Notes: type host; it was reported as *Micropogonias lineatus*. Campbell and Beveridge (1996) also mentioned *Micropogonias furnieri* as type host, but Diesing (1850) reported the worms only from *Micropogonias lineatus*. The type material is no longer extant in NHMW (Campbell and Beveridge 1996).

*Paralichthys isosceles* (Actinopterygii: Paralichthyidae); marine; muscles; metacestode; WTSA; Brazil (Felizardo et al. 2010).

*Plagioscion squamosissimus* (Actinopterygii: Sciaenidae); freshwater; muscles; metacestode; Amazon River basin (estuary); Brazil (Silva 2010).

*Pogonias cromis* (Actinopterygii: Sciaenidae); marine; body cavity; metacestode; WTSA; Brazil (Pereira and Boeger 2005).

*Pomadasys crocro* (Actinopterygii: Haemulidae); marine; muscles; metacestode; NBS (Amazon River estuary); Brazil (Diesing 1855).

Note: host reported as *Pristipoma coro*.

*Umbrina canosai* (Actinopterygii: Sciaenidae); marine; body cavity; metacestode; WTSA; Brazil (Pereira and Boeger 2005).

Clupeidae gen. sp. (Actinopterygii); marine; mesentery; metacestode; WTSA; Brazil (Palm 2004).

***Pterobothrium kingstoni* Campbell & Beveridge, 1996**

[Syn. *Synbothrium lintoni* MacCallum, 1921 (*pro parte*)]

*Citharichthys spilopterus* (Actinopterygii: Paralichthyidae); marine; body cavity, muscles; metacestode; TSA; Brazil (Palm 1997).

*Haemulon aurolineatum* (Actinopterygii: Haemulidae); marine; muscles; metacestode; TSA; Brazil (Campbell and Beveridge 1996).

Note: the worms were collected by Palm in Brazil’s Northeastern coast.

***Pterobothrium* sp.**

*Conodon nobilis* (Actinopterygii: Haemulidae); marine; body cavity; metacestode; WTSA; Brazil (Paschoal et al. 2015).

*Cynoscion acoupa* (Actinopterygii: Sciaenidae); marine; body cavity; metacestode; WTSA; Brazil (Palm 2004).

*Cynoscion striatus* (Actinopterygii: Sciaenidae); marine; muscles; metacestode; WTSA; Brazil (Santos and Zogbi 1971).

Note: metacestode described as *Tetrarhynchus fragilis* (*nomen nudum*) and identified as *Pterobotrium* sp. by Palm (2004).

*Epinephelus* sp. (Actinopterygii: Serranidae); marine; body cavity; metacestode; TNA; Venezuela (Palm 2004).

*Gymnura* sp. (Elasmobranchii: Gymnuridae); marine; spiral valve; adult; WTSA; Brazil (Palm 2004).

*Macrodon ancylodon* (Actinopterygii: Sciaenidae); marine; muscles; metacestode; WTSA; Brazil (Santos and Zogbi 1971).

Note: Santos and Zogbi (1971) reported the metacestode as *Tetrarhynchus fragilis* (see Palm 2004).

*Menticirrhus americanus* (Actinopterygii: Sciaenidae); marine; body cavity; metacestode; WTSA; Brazil (Palm 2004).

Note: Palm (2004) studied specimens collected by Travassos in 1921 that are deposited in CHIOC.

*Micropogonias furnieri* (Actinopterygii: Sciaenidae); marine; body cavity, muscles; metacestode; WTSA; Brazil (Santos and Zogbi 1971; São Clemente 1986a, b, 1987).

Note: Santos and Zogbi (1971) reported the host as *Micropogon opercularis* and the metacestode as *Tetrarhynchus fragilis* (see Palm 2004).

*Mycteroperca bonaci* (Actinopterygii: Serranidae); marine; body cavity; metacestode; WTSA; Brazil (Palm 2004).

*Pagrus pagrus* (Actinopterygii: Sparidae); marine; body cavity; metacestode; WTSA; Brazil (Palm 2004).

*Paralichthys isosceles* (Actinopterygii: Paralichthyidae); marine; site of infection not given; metacestode; WTSA; Brazil (Luque and Poulin 2004).

*Paralichthys* sp. (Actinopterygii: Paralichthyidae); marine; muscles; metacestode; WTSA; Brazil (Santos and Zogbi 1971).

Notes: Santos and Zogbi (1971) reported the metacestode as *Tetrarhynchus fragilis* (see Palm 2004).

*Pomatomus saltatrix* (Actinopterygii: Pomatomidae); marine; intestine; metacestode; WTSA; Brazil (Palm 2004).

Note: host reported as *Pomatomus saltator*.

*Prionotus* sp. (Actinopterygii: Triglidae); marine; body cavity; metacestode; WTSA; Brazil (Palm 2004).

*Umbrina canosai* (Actinopterygii: Sciaenidae); marine; muscles; metacestode; WTSA; Brazil (Santos and Zogbi 1971).

Notes: Santos and Zogbi (1971) described the metacestodes as *Tetrarhynchus fragilis* (see Palm 2004).

Siluriform fish (Actinopterygii); marine; body cavity; metacestode; WTSA; Brazil (Palm 2004).

Unidentified ray (Elasmobranchii); marine; spiral valve; adult; WTSA; Brazil (Rego et al. 1974).

Note: Rego et al. (1974) named the cestodes as Pterobothriidae gen. sp. and Palm (2004) assigned them to *Pterobothrium* sp.

**Unidentified pterobothriids**

*Bagre bagre* (Actinopterygii: Ariidae); marine; site of infection not given; metacestode; NBS; Brazil (Vicente and Fernandes 1978).

*Macrodon ancylodon* (Actinopterygii: Sciaenidae); marine; site of infection not given; metacestode; NBS; Brazil (Vicente and Fernandes 1978).

###### Superfamily Otobothrioidea Dollfus, 1942

####### Family Otobothriidae Dollfus, 1942

***Otobothrium* sp.**

*Balistes vetula* (Actinopterygii: Balistidae); marine; body cavity, muscles; metacestode; WTSA; Brazil (São Clemente et al. 1995; Alves et al. 2005).

*Paralichthys isosceles* (Actinopterygii: Paralichthyidae); marine; body cavity, liver, mesentery, intestine, stomach; metacestode; WTSA; Brazil (Felizardo et al. 2010).

*Poecilancistrium caryophyllum* (Diesing, 1850) Dollfus, 1929*

[For synonyms, see Palm (2004)]

*Carcharhinus leucas* (Elasmobranchii: Carcharhinidae); marine; spiral valve; adult; NBS (estuarine waters); Brazil (Diesing 1850, 1856).

Note: host reported as *Prionodon leucas*.

*Cilus gilberti* (Actinopterygii: Sciaenidae); marine; peritoneum; metacestode; WTSP; Peru (Escalante and Carvajal 1984).

Note: host reported as *Sciaena gilberti*.

*Cynoscion acoupa* (Actinopterygii: Sciaenidae); marine; muscles; metacestode; NBS; Brazil (Dias et al. 2011b).

*Macrodon ancylodon* (Actinopterygii: Sciaenidae); marine; muscles; metacestode; NBS; Brazil (Oliveira et al. 2009; Dias et al. 2011b).

*Micropogonias altipinnis* (Actinopterygii: Sciaenidae); marine; muscles; metacestode; TEP; Ecuador (Palm 2004).

*Micropogonias furnieri* (Actinopterygii: Sciaenidae); marine; body cavity, muscles; metacestode; TNA, WTSA; Brazil, Venezuela (São Clemente 1986a, b, 1987; Vicente et al. 1989; Pereira 1993; Pereira and Boeger 2005).

*Plagioscion squamosissimus* (Actinopterygii: Sciaenidae); freshwater; muscles; metacestode; Amazon River basin (estuary of Amazon River); Brazil (Silva 2010).

*Rhizoprionodon lalandii* (Elasmobranchii: Carcharhinidae); marine; spiral valve; adult; NBS (estuarine waters); Brazil (Diesing 1850, 1856).

Note: type host; it was reported as *Scoliodon lalandii*.

####### Family Pseudotobothriidae Palm, 1995

*Pseudotobothrium dipsacum* (Linton, 1897) Dollfus 1942*

[Syn. *Otobothrium dipsacum* Linton, 1897]

*Haemulon plumierii* (Actinopterygii: Haemulidae); marine; body cavity; metacestode; TSA; Brazil (Palm 1997).

*Hyporthodus niveatus* (Actinopterygii: Serranidae); marine; body cavity; metacestode; WTSA; Brazil (Palm 2004).

Note: host reported as *Epinephelus niveatus*.

*Pseudupeneus maculatus* (Actinopterygii: Mullidae); marine; body cavity; metacestode; TSA; Brazil (Palm 1997).

##### Suborder Trypanobatoida Olson, Caira, Jensen, Overstreet, Palm & Beveridge, 2010

###### Superfamily Eutetrarhynchoidea Guiart, 1927

####### Family Eutetrarhynchidae Guiart, 1927

***Dollfusiella acuta* Menoret & Ivanov, 2015**

*Atlantoraja castelnaui* (Elasmobranchii: Arhynchobatidae); marine; spiral valve; adult; WTSA; Argentina (Menoret and Ivanov 2015).

*Atlantoraja platana* (Elasmobranchii: Arhynchobatidae); marine; spiral valve; adult; Magellanic; Argentina (Menoret and Ivanov 2015).

*Sympterygia acuta* (Elasmobranchii: Arhynchobatidae); marine; spiral valve; adult; Magellanic, WTSA; Argentina (Menoret and Ivanov 2015).

Note: type host.

*Sympterygia bonapartii* (Elasmobranchii: Arhynchobatidae); marine; spiral valve; adult; WTSA; Argentina (Menoret and Ivanov 2015).

***Dollfusiella musteli* (Carvajal, 1974) Beveridge, Neifar & Euzet, 2004**

[Syns. *Prochristianella musteli* Carvajal, 1974; *Eutetrarhynchus musteli* (Carvajal, 1974) Beveridge, 1990]

*Mustelus mento* (Elasmobranchii: Triakidae); marine; spiral valve; adult; WTSP; Chile (Carvajal 1974).

Note: type host.

***Dollfusiella taminii* Menoret & Ivanov, 2014**

*Psammobatis bergi* (Elasmobranchii: Arhynchobatidae); marine; spiral valve; adult; WTSA; Argentina (Menoret and Ivanov 2014).

Note: type host.

***Dollfusiella vooremi* (São Clemente & Gomes, 1989) Beveridge, Neifar & Euzet, 2004**

[Syn. *Eutetrarhynchus vooremi* São Clemente & Gomes, 1989]

*Mustelus canis* (Elasmobranchii: Triakidae); marine; spiral valve; adult; WTSA; Brazil (São Clemente and Gomes 1989b; São Clemente et al. 1991b).

Note: type host.

*Mustelus schmitti* (Elasmobranchii: Triakidae); marine; spiral valve; adult; Magellanic, WTSA; Argentina, Brazil (São Clemente and Gomes 1989b; São Clemente et al. 1991b; Alarcos et al. 2006; Menoret and Ivanov 2014).

Note: tapeworms reported as *Eutetrarhynchus vooremi* by Alarcos et al. (2006).

*Sympterygia bonapartii* (Elasmobranchii: Arhynchobatidae); marine; spiral valve; adult; WTSA; Argentina (Tanzola et al. 1998).

Notes: Tanzola et al. (1998) reported the tapeworms as *Eutetrarhynchus vooremi*. Menoret and Ivanov (2014) examined 42 specimens of *Sympterygia bonapartii*, also from the Argentinian coast, and none *Dollfusiella vooremi* was found in these sharks. Thus, they suggested that this report is most likely a result of misidentification.

***Dollfusiella* sp.**

*Micropogonias furnieri* (Actinopterygii: Sciaenidae); marine; body cavity; metacestode; WTSA, WTSP; Brazil, Chile (Oliva 1999; Pereira and Boeger 2005).

Note: Oliva (1999) reported tapeworms as *Eutetrarhynchus* sp., but most likely they belonged to the closely-related genus *Dollfusiella*.

***Mecistobothrium oblongum* Menoret & Ivanov, 2015**

*Myliobatis goodei* (Elasmobranchii: Myliobatidae); marine; spiral valve; adult; Magellanic; Argentina (Menoret and Ivanov 2015).

Note: type host.

***Parachristianella damiani* Menoret & Ivanov, 2014**

*Myliobatis goodei* (Elasmobranchii: Myliobatidae); marine; spiral valve; adult; WTSA; Argentina (Menoret and Ivanov 2014).

Note: type host.

***Parachristianella monomegacantha* Kruse, 1959**

*Himantura schmardae* (Elasmobranchii: Dasyatidae); marine; spiral valve; adult; TNA; Venezuela (Mayes and Brooks 1981).

Note: tapeworms reported as Parachristianella cf. monomegacantha.

*Rhinobatos planiceps* (Elasmobranchii: Rhinobatidae); marine; spiral valve; adult; WTSP; Chile (Dailey and Carvajal 1976).

*Paroncomegas araya* (Woodland, 1934) Campbell, Marques & Ivanov, 1999*

[Syns. *Tentacularia araya* Woodland, 1934; *Eutetrarhynchus araya* (Woodland, 1934) Rego & Dias, 1976]

*Potamotrygon falkneri* (Elasmobranchii: Potamotrygonidae); freshwater; spiral valve; adult; Paraná River basin; Paraguay (Brooks et al. 1981b; Lacerda et al. 2008, 2009).

Potamotrygon cf. falkneri (Elasmobranchii: Potamotrygonidae); freshwater; spiral valve; adult; Amazon River basin; Peru (Reyda 2008).

Note: host reported as Potamotrygon cf. castexi.

*Potamotrygon motoro* (Elasmobranchii: Potamotrygonidae); freshwater; spiral valve; adult; Amazon and Paraná River basins; Argentina, Brazil (Rego and Dias 1976; Campbell et al. 1999; Reyda 2008).

Notes: Rego and Dias (1976) synonymized *Eutetrarhynchus baeri* Lopez-Neyra & Diaz-Ungria, 1958 with *Eutetrarhynchus araya*, but Campbell et al. (1999) recognized their morphological distinctness and named the former species as *Paroncomegas* sp. until further studies confirm its validity. Sequences of partial 18S (DQ642963) and 28S (DQ642801) (Olson et al. 2010).

*Potamotrygon orbignyi* (Elasmobranchii: Potamotrygonidae); freshwater; spiral valve; adult; Orinoco River basin; Venezuela (Brooks et al. 1981b).

Note: host reported as *Potamotrygon reticulatus*.

*Potamotrygon* sp. (Elasmobranchii: Potamotrygonidae); freshwater; spiral valve; adult; Amazon River basin; Brazil (Woodland 1934c).

Note: type host; it was described as *Trygon* sp.

***Paroncomegas* sp.**

[Syn. *Eutetrarhynchus baeri* Lopez-Neyra & Diaz-Ungria, 1958]

*Potamotrygon orbignyi* (Elasmobranchii: Potamotrygonidae); freshwater; spiral valve; adult; Orinoco River basin; Venezuela (Lopez-Neyra and Diaz-Ungria, 1958).

Note: host reported as *Paratrygon hystrix*. Campbell et al. (1999) assigned these tapeworms to *Paroncomegas* sp. until new morphological data are available (see the note above). It is treated as *Platybothrium baeri* (Lopez-Neyra & Diaz-Ungria, 1958) in GCD (Caira et al. 2012).

***Prochristianella heteracantha* Dailey & Carvajal, 1976**

*Rhinobatos planiceps* (Elasmobranchii: Rhinobatidae); marine; spiral valve; adult; WTSP; Chile, Peru (Dailey and Carvajal 1976; Tantaleán and Rodríguez 1987; Iannacone et al. 2011).

Note: type host.

####### Family Rhinoptericolidae Carvajal & Campbell, 1975

*Rhinoptericola megacantha* Carvajal & Campbell, 1975*

*Rhinoptera bonasus* (Elasmobranchii: Myliobatidae); marine; spiral valve; adult; TNA; Venezuela (Mayes and Brooks 1981).

Note: type host.

*Rhinoptera brasiliensis* (Elasmobranchii: Myliobatidae); marine; spiral valve; adult; WTSA; Brazil (Napoleão et al. 2015).

###### Superfamily Tentacularioidea Poche, 1926

####### Family Sphyriocephalidae Pintner, 1913

***Hepatoxylon megacephalum* (Rudolphi, 1819) Dollfus, 1942**

[Syns. *Tetrarhynchus megacephalus* Rudolphi, 1819; *Tetrarhynchus scyllium canicula* Wagener, 1854]

*Trichomycterus punctulatus* (Actinopterygii: Trichomycteridae); freshwater; site

of infection not given; metacestode; Lima; Peru (Dollfus 1942).

*Hepatoxylon trichiuri* (Holten, 1802) Bosc, 1811*

[For synonyms, see Palm (2004)]

*Brama australis* (Actinopterygii: Bramidae); marine; gonads, mesentery, muscles; metacestode; WTSP; Chile (George-Nascimento and Ortiz 1982; George-Nascimento et al. 2002)

Note: host reported as *Lepidotus australis* by George-Nascimento and Ortiz (1982).

*Brama japonica* (Actinopterygii: Bramidae); marine; body cavity; metacestode; WTSP; Peru (Iannacone and Alvariño 2013).

*Coelorinchus chilensis* (Actinopterygii: Macrouridae); marine; intestine; metacestode; JFD; Chile (Pardo-Gandarilhas et al. 2008).

*Coryphaena hippurus* (Actinopterygii: Coryphaenidae); marine; liver, intestinal

serosa, stomach wall; metacestode; WTSA; Brazil (Rudolphi 1819; São Clemente et al. 2001).

*Dissostichus eleginoides* (Actinopterygii: Nototheniidae); marine; body cavity, mesentery, stomach wall; metacestode; Magellanic, WTSP; Chile, Falkland Islands (Rodríguez and George-Nascimento 1996; Brickle et al. 2006; Oliva et al. 2008a).

*Genypterus blacodes* (Actinopterygii: Ophidiidae); marine; stomach; metacestode; WTSP; Chile (Cattan 1977; Riffo 1994).

*Genypterus brasiliensis* (Actinopterygii: Ophidiidae); marine; mesentery, muscles; metacestode; WTSA; Brazil (São Clemente et al. 2004).

*Genypterus chilensis* (Actinopterygii: Ophidiidae); marine; mesentery; metacestode; WTSP; Chile (Vergara and George-Nascimento 1982).

*Genypterus maculatus* (Actinopterygii: Ophidiidae); marine; gonads, mesentery, muscles; metacestode; WTSP; Chile (George-Nascimento and Ortiz 1982; George-Nascimento and Huet 1984; Muñoz and George-Nascimento 2008).

*Isacia conceptionis* (Actinopterygii: Haemulidae); marine; intestinal surface; metacestode; WTSP; Chile (Dollfus 1930).

*Lampris guttatus* (Actinopterygii: Lampridae); marine; gonads, mesentery, muscles; metacestode; WTSP; Chile (George-Nascimento and Ortiz 1982).

Note: host reported as *Lampris regia*.

*Macruronus magellanicus* (Actinopterygii: Merlucciidae); marine; body cavity, gonads, mesentery, muscles; Magellanic; Chile, Falkland Islands (George-Nascimento and Ortiz 1982; Oliva 2001; Chávez et al. 2012; MacKenzie et al. 2013).

*Merluccius australis* (Actinopterygii: Merlucciidae); marine; body cavity, muscles; metacestode; Magellanic, WTSP; Argentina, Chile, Falkland Islands (Fernández 1985; George-Nascimento and Arancibia 1994; González and Carvajal 1994; MacKenzie and Longshaw 1995; Chávez et al. 2012; Torres et al. 2014).

*Merluccius gayi gayi* (Actinopterygii: Merlucciidae); marine; body cavity, mesentery; metacestode; Magellanic, WTSP; Chile (Tagle 1951; George-Nascimento 1996; Oliva and Ballón 2002; Chávez et al. 2012).

Note: host reported as *Merluccius gayi* by some authors.

*Merluccius hubbsi* (Actinopterygii: Merlucciidae); marine; body cavity, mesentery; metacestode; Magellanic, WTSA; Argentina, Falkland Islands, Uruguay (Szidat 1955, 1961; MacKenzie and Longshaw 1995; Sardella and Timi 1996, 2004).

*Micromesistius australis australis* (Actinopterygii: Gadidae); marine; site of

infection not given; metacestode; Magellanic; Chile, Falkland Islands (George-Nascimento et al. 2011; Chávez et al. 2012).

*Notacanthus sexspinis* (Actinopterygii: Notacanthidae); marine; intestine; metacestode; JFD; Chile (Pardo-Gandarilhas et al. 2008).

*Oncorhynchus tshawytscha* (Actinopterygii: Salmonidae); anadromous; body cavity; metacestode; Magellanic (Curaco de Vélez); Chile (Reyes-Piraino 1982).

*Prionace glauca* (Elasmobranchii: Carcharhinidae); marine; body cavity, stomach serosa, liver; metacestode; JFD, WTSA, WTSP; Brazil, Chile, Peru (Yáñez 1950; Carvajal 1974; Cattan et al. 1979; Escalante 1986; São Clemente et al. 2001; Knoff et al. 2002, 2004b; Cousin et al. 2003).

*Pseudopercis semifasciata* (Actinopterygii: Pinguipedidae); marine; mesentery; metacestode; Magellanic; Argentina (Timi and Lanfranchi 2009a).

*Salilota australis* (Actinopterygii: Moridae); marine; body cavity; metacestode; Magellanic; Argentina (Szidat 1961).

*Scomber japonicus* (Actinopterygii: Scombridae); marine; site of infection not given; metacestode; WTSP; Chile (Rodríguez et al. 2000).

*Sebastes capensis* (Actinopterygii: Sebastidae); marine; site of infection not given; metacestode; WTSP; Chile (González and Poulin 2005a, b; González et al. 2006).

*Somniosus pacificus* (Elasmobranchii: Somniosidae); marine; intestinal surface; metacestode; WTSP; Chile (Reyes-Piraino 1982).

*Trachurus murphyi* (Actinopterygii: Carangidae); marine; site of infection not given; metacestode; WTSP; Chile (George-Nascimento 2000; Rodríguez et al. 2000; Oliva 1999).

***Hepatoxylon* sp.**

*Helicolenus lengerichi* (Actinopterygii: Sebastidae); marine; mesentery; metacestode; WTSP; Chile (George-Nascimento and Iriarte 1989; Balboa and George-Nascimento 1998).

*Macrourus holotrachys* (Actinopterygii: Macrouridae); marine; visceral cavity; metacestode; WTSP: Chile (Ñacari and Oliva 2016).

*Micromesistius australis australis* (Actinopterygii: Gadidae); marine; site of infection not given; metacestode; Magellanic; Argentina, Chile (Niklitschek et al. 2010).

*Trachurus murphyi* (Actinopterygii: Carangidae); marine; site of infection not given; metacestode; WTSP; Chile (George-Nascimento and Oliva 2015).

***Heterosphyriocephalus tergestinus* (Pintner, 1913) Dallarés, Carrassón & Schaeffner, 2016**

[Syn. *Sphyriocephalus tergestinus* Pintner, 1913]

*Sarda chilensis* (Actinopterygii: Scombridae); marine; stomach; metacestode; WTSP; Peru (Chero et al. 2015).

####### Family Tentaculariidae Poche, 1926

***Heteronybelinia annakohnae* Pereira & Boeger, 2005**

*Ctenosciaena gracilicirrhus* (Actinopterygii: Sciaenidae); marine; body cavity; metacestode; WTSA; Brazil (Pereira and Boeger 2005).

Notes: type host. Pereira (1998) described *Nybelinia annakohnae* in his thesis (the type-material was deposited in CHIOC and USNPC), but it does not represent a formal publication (ICZN 1999). Six years later, Pereira and Boeger (2005) properly described the species, but transferred it to *Heteronybelinia*.

*Cynoscion guatucupa* (Actinopterygii: Sciaenidae); marine; body cavity; metacestode; WTSA; Brazil (Pereira and Boeger 2005).

*Cynoscion jamaicensis* (Actinopterygii: Sciaenidae); marine; body cavity; metacestode; WTSA; Brazil (Pereira and Boeger 2005).

*Menticirrhus americanus* (Actinopterygii: Sciaenidae); marine; body cavity; metacestode; WTSA; Brazil (Pereira and Boeger 2005).

*Heteronybelinia estigmena* (Dollfus, 1960) Palm, 1999*

[For synonyms, see Palm (2004)]

*Cynoscion jamaicensis* (Actinopterygii: Sciaenidae); marine; body cavity; metacestode; WTSA; Brazil (Pereira and Boeger 2005).

*Haemulon plumierii* (Actinopterygii: Haemulidae); marine; body cavity; metacestode; TSA; Brazil (Palm 1997).

Note: tapeworms reported as *Nybelinia senegalensis* Dollfus, 1960.

*Sphyraena guachancho* (Actinopterygii: Sphyraenidae); marine; body cavity; metacestode; TSA; Brazil (Palm 1997).

***Heteronybelinia mattisi* Menoret & Ivanov, 2013**

*Nemadactylus bergi* (Actinopterygii: Cheilodactylidae); marine; mesentery; metacestode; WTSA; Argentina (Menoret and Ivanov 2013).

*Raneya brasiliensis* (Actinopterygii: Ophidiidae); marine; mesentery; metacestode; WTSA; Argentina (Menoret and Ivanov 2013).

*Sympterygia bonapartii* (Elasmobranchii: Arhynchobatidae); marine; spiral valve; adult; WTSA; Argentina (Menoret and Ivanov 2013).

Notes: type host. Menoret and Ivanov (2013) also found larval forms in the pyloric caeca of *Sympterygia bonapartii*.

***Heteronybelinia nipponica* (Yamaguti, 1952) Palm, 1999**

[Syns. *Nybelinia nipponica* Yamaguti, 1952; *Nybelinia rougetcampanae* Dollfus, 1960; *Heteronybelinia rougetcampanae* (Dollfus, 1960) Palm, 1999]

*Carcharhinus signatus* (Elasmobranchii: Carcharhinidae); marine; spiral valve; metacestode; WTSA; Brazil (Knoff et al. 2002, 2004a).

*Genypterus brasiliensis* (Actinopterygii: Ophidiidae); marine; body cavity; metacestode; WTSA; Brazil (São Clemente et al. 2004).

*Menticirrhus americanus* (Actinopterygii: Sciaenidae); marine; body cavity; metacestode; WTSA; Brazil (Pereira and Boeger 2005).

*Mullus argentinae* (Actinopterygii: Mullidae); marine; body cavity; metacestode; WTSA; Brazil (Luque et al. 2002).

*Paralichthys isosceles* (Actinopterygii: Paralichthyidae); marine; body cavity, intestine, kidney, muscles; metacestode; WTSA; Brazil (Felizardo et al. 2010).

*Paralichthys patagonicus* (Actinopterygii: Paralichthyidae); marine; stomach; metacestode; WTSA; Brazil (Fonseca et al. 2012).

*Sphyrna lewini* (Elasmobranchii: Sphyrnidae); marine; spiral valve; adult; WTSA; Brazil (São Clemente et al. 1991b; São Clemente and Gomes 1992).

Note: tapeworms reported as Nybelinia (Syngenes) rougetcampanae.

*Sphyrna zygaena* (Elasmobranchii: Sphyrnidae); marine; spiral valve; metacestode; WTSA; Brazil (Knoff et al. 2002; Gomes et al. 2005).

*Umbrina canosai* (Actinopterygii: Sciaenidae); marine; body cavity; metacestode; WTSA; Brazil (Pereira and Boeger 2005).

*Xystreurys rasile* (Actinopterygii: Paralichthyidae); marine; mesentery, stomach; metacestode; WTSA; Brazil (Fonseca et al. 2012).

***Heteronybelinia overstreeti* Palm, 2004**

*Pseudupeneus maculatus* (Actinopterygii: Mullidae); marine; body cavity; metacestode; TSA; Brazil (Palm 1997).

Note: Palm (1997) reported plerocercoids of Nybelinia cf. lingualis Cuvier, 1817 from this host, but later (Palm 2004) re-assigned these larvae to *Heteronybelinia overstreeti*.

***Heteronybelinia perideraeus* (Shipley & Hornell, 1906) Palm, 1999**

[Syns. *Tetrarhynchus perideraeus* Shipley & Hornell, 1906; *Stenobothrium perideraeum* (Shipley & Hornell, 1906) Pintner, 1913; *Nybelinia dakari* Dollfus, 1960]

*Notorynchus cepedianus* (Elasmobranchii: Hexanchidae); marine; spiral valve; adult; WTSA; Brazil (São Clemente et al. 1991b; São Clemente and Gomes 1992).

Note: host reported as *Notorynchus pectorosus*. São Clemente and Gomes (1992) recorded the tapeworms as Nybelinia (Nybelinia) bisulcata (Linton, 1889), but Palm (2004) re-assigned the material deposited in CHIOC to *Heteronybelinia perideraeus*.

***Heteronybelinia yamagutii* (Dollfus, 1960) Palm, 1999**

[Syn. *Nybelinia yamagutii* Dollfus, 1960]

*Carcharhinus signatus* (Elasmobranchii: Carcharhinidae); marine; spiral valve; metacestode; WTSA; Brazil (Knoff et al. 2002, 2004a).

***Heteronybelinia* sp.**

*Urophycis brasiliensis* (Actinopterygii: Phycidae); marine; site of infection not

given; metacestode; WTSA; Brazil (Luque and Poulin 2004).

*Mixonybelinia beveridgei* (Palm, Walter, Schwerdtfeger & Reimer, 1997) Palm, 1999*

[Syn. *Nybelinia beveridgei* Palm, Walter, Schwerdtfeger & Reimer, 1997]

*Dipturus trachyderma* (Elasmobranchii: Rajidae); marine; stomach; metacestode; WTSA; Brazil (Knoff et al. 2002, 2004a).

Note: host reported as *Diphyllobothrium trachydermus*.

*Genypterus brasiliensis* (Actinopterygii: Ophidiidae); marine; liver, mesentery, serosa of stomach; metacestode; WTSA; Brazil (São Clemente et al. 2004).

***Mixonybelinia edwinlintoni* (Dollfus, 1960) Palm & Walter, 2000**

[Syn. *Nybelinia edwinlintoni* Dollfus, 1960]

*Pseudupeneus maculatus* (Actinopterygii: Mullidae); marine; body cavity; metacestode; TSA; Brazil (Palm 1997).

***Mixonybelinia* sp.**

*Lophius gastrophysus* (Actinopterygii: Lophiidae); marine; muscles; metacestode; WTSA; Brazil (São Clemente et al. 2007).

***Nybelinia africana* Dollfus, 1960**

*Pseudupeneus maculatus* (Actinopterygii: Mullidae); marine; body cavity; metacestode; TSA; Brazil (Palm 2004).

***Nybelinia bisulcata* (Linton, 1889) Dollfus, 1929**

[Syns. *Rhynchobothrium bisulcatum* Linton, 1889; *Tetrarhynchus bisulcatus* (Linton, 1889) Linton, 1890]

*Umbrina canosai* (Actinopterygii: Sciaenidae); marine; body cavity; metacestode; WTSA; Brazil (Pereira and Boeger 2005).

Note: the taxonomic status of this species is problematic since the type material is a mixture of different species under the same name (Palm 2004).

***Nybelinia erythraea* Dollfus, 1960**

*Paralichthys patagonicus* (Actinopterygii: Paralichthyidae); marine; stomach; metacestode; WTSA; Brazil (Fonseca et al. 2012).

*Xystreurys rasile* (Actinopterygii: Paralichthyidae); marine; stomach; metacestode; WTSA; Brazil (Fonseca et al. 2012).

***Nybelinia fayapaulazariahi* Reimer, 1980**

*Rhizoprionodon terraenovae* (Elasmobranchii: Carcharhinidae); marine; spiral valve; adult; WTSA; Brazil (Palm 2004).

***Nybelinia indica* Chandra, 1986**

*Pseudupeneus maculatus* (Actinopterygii: Mullidae); marine; body cavity; metacestode; TSA; Brazil (Palm 1997).

*Nybelinia lingualis* (Cuvier, 1817) Dollfus, 1929*

[For synonyms, see Palm (2004)]

*Cynoscion* sp. (Actinopterygii: Sciaenidae); marine; mesentery; metacestode; WTSA; Brazil (Ortubay 1944).

*Haemulon plumierii* (Actinopterygii: Haemulidae); marine; body cavity; metacestode; TSA; Brazil (Palm 1997).

Note: tapeworms reported as Nybelinia cf. lingualis.

*Isurus oxyrinchus* (Elasmobranchii: Lamnidae); marine; spiral valve; adult; WTSA; Brazil (Knoff et al. 2002; Gomes et al. 2005).

*Mustelus canis* (Elasmobranchii: Carcharhinidae); marine; spiral valve; metacestode; WTSA; Brazil (São Clemente and Gomes 1989b; São Clemente et al. 1991b).

*Mustelus schmitti* (Elasmobranchii: Carcharhinidae); marine; spiral valve; metacestode; WTSA; Brazil (São Clemente and Gomes 1989b; São Clemente et al. 1991b).

Note: tapeworms reported as Nybelinia (Nybelinia) lingualis.

*Oncopterus darwinii* (Actinopterygii: Pleuronectidae); marine; intestinal surface; metacestode; WTSA; Argentina (Szidat 1961).

*Paralichthys isosceles* (Actinopterygii: Paralichthyidae); marine; stomach, intestine, mesentery, spleen serosa, muscles; metacestode; WTSA; Brazil (Felizardo et al. 2010).

*Paralichthys patagonicus* (Actinopterygii: Paralichthyidae); marine; body cavity, mesentery, stomach; metacestode; WTSA; Argentina, Brazil (Szidat 1961; Fonseca et al. 2012).

*Porichthys porosissimus* (Actinopterygii: Batrachoididae); marine; body cavity; metacestode; WTSA; Argentina (Tanzola et al. 1997).

*Pseudupeneus maculatus* (Actinopterygii: Mullidae); marine; body cavity; metacestode; TSA; Brazil (Palm 2004).

*Selene vomer* (Actinopterygii: Carangidae); marine; body cavity; metacestode; TSA; Brazil (Palm 1997).

Note: tapeworms reported as Nybelinia cf. lingualis.

*Sympterygia bonapartii* (Elasmobranchii: Arhynchobatidae); marine; spiral valve; metacestode; WTSA; Argentina (Tanzola et al. 1998).

*Trachurus murphyi* (Actinopterygii: Carangidae); marine; wall of pharynx; metacestode; WTSP; Chile (Palm 2004).

*Xystreurys rasile* (Actinopterygii: Paralichthyidae); marine; body cavity, mesentery, stomach; metacestode; WTSA; Brazil (Fonseca et al. 2012).

***Nybelinia surmenicola* Okada in Dollfus, 1929**

*Hippoglossina macrops* (Actinopterygii: Paralichthyidae); marine; intestine; metacestode; WTSP; Chile (González et al. 2001, 2008; Oliva et al. 2004).

*Merluccius gayi gayi* (Actinopterygii: Merlucciidae); marine; body cavity, mesentery; metacestode; WTSP; Chile (Oliva and Ballón 2002).

*Paralichthys adspersus* (Actinopterygii: Paralichthyidae); marine; body cavity, gill arches, muscles; metacestode; WTSP; Chile (Oliva et al. 1996).

*Trachurus murphyi* (Actinopterygii: Carangidae); marine; site of infection not given; metacestode; WTSP; Chile, Peru (Oliva 1999).

***Nybelinia* sp.**

*Aphos porosus* (Actinopterygii: Batrachoididae); marine; body cavity; metacestode; WTSP; Chile (Cortés and Muñoz 2008, 2009).

*Balistes capriscus* (Actinopterygii: Balistidae); marine; mesentery; metacestode; WTSA; Brazil (Luque and Poulin 2004; Alves et al. 2005).

*Brama australis* (Actinopterygii: Bramidae); marine; site of infection not given; metacestode; WTSP; Chile (George-Nascimento et al. 2002).

*Brama japonica* (Actinopterygii: Bramidae); marine; body cavity; metacestode; WTSP; Peru (Iannacone and Alvariño 2013).

*Caranx hippos* (Actinopterygii: Carangidae); marine; mesentery; metacestode; WTSA; Brazil (Luque and Alves 2001; Luque and Poulin 2004).

*Caranx latus* (Actinopterygii: Carangidae); marine; mesentery; metacestode; WTSA; Brazil (Luque and Alves 2001; Luque and Poulin 2004).

*Cilus gilberti* (Actinopterygii: Sciaenidae); marine; site of infection not given; metacestode; WTSP; Chile (Garcías et al. 2001).

*Conger orbignianus* (Actinopterygii: Congridae); marine; intestinal surface, mesentery; metacestode; WTSA; Argentina (Timi and Lanfranchi 2013).

*Coryphaena hippurus* (Actinopterygii: Coryphaenidae); marine; body cavity, stomach; metacestode; WTSP; Peru (López de McDonald and Tantaleán 1985; Vásquez-Ruiz and Jara-Campos 2012).

*Cynoscion analis* (Actinopterygii: Sciaenidae); marine; intestine, surface of stomach; metacestode; WTSP; Peru (Llerena et al. 2001).

*Cynoscion guatucupa* (Actinopterygii: Sciaenidae); marine; body cavity, mesentery; metacestode; WTSA; Brazil (Sabas and Luque 2003; Luque and Poulin 2004; Timi et al. 2005).

*Dactylopterus volitans* (Actinopterygii: Dactylopteridae); marine; mesentery; metacestode; WTSA; Brazil (Luque and Poulin 2004; Cordeiro and Luque 2005).

*Diapterus rhombeus* (Actinopterygii: Gerreidae); marine; mesentery; metacestode; WTSA; Brazil (Luque and Poulin 2004).

*Genypterus brasiliensis* (Actinopterygii: Ophidiidae); marine; body cavity, mesentery, intestinal serosa; metacestode; WTSA; Brazil (Alves et al. 2002a, b; Luque and Poulin 2004; São Clemente et al. 2004).

*Genypterus maculatus* (Actinopterygii: Ophidiidae); marine; site of infection not given; metacestode; WTSP; Chile (George-Nascimento and Huet 1984; Muñoz and George-Nascimento 2008).

*Hippoglossina macrops* (Actinopterygii: Paralichthyidae); marine; gill arches; metacestode; WTSP; Chile (Riffo 1991).

*Isacia conceptionis* (Actinopterygii: Haemulidae); marine; mesentery; metacestode; WTSP; Peru (Iannacone et al. 2015).

*Lophius gastrophysus* (Actinopterygii: Lophiidae); marine; mesentery, body

cavity, muscles; metacestode; WTSA; Brazil (São Clemente et al. 2007).

*Macrodon ancylodon* (Actinopterygii: Sciaenidae); marine; mesentery; metacestode; WTSA; Brazil (Sabas and Luque 2003; Luque and Poulin 2004).

*Merluccius gayi peruanus* (Actinopterygii: Merlucciidae); marine; intestine; metacestode; WTSP; Peru (Durán and Oliva 1980).

*Merluccius hubbsi* (Actinopterygii: Merlucciidae); marine; mesentery; metacestode; Magellanic; Argentina (Sardella and Timi 2004).

*Micropogonias furnieri* (Actinopterygii: Sciaenidae); marine; mesentery; metacestode; TSA; Brazil (Luque et al. 2010).

*Mola ramsayi* (Actinopterygii: Molidae); marine; intestine; metacestode; WTSP; Chile (Villalba and Fernández 1985).

Note: the authors distinguished two different morphotypes.

*Mullus argentinae* (Actinopterygii: Mullidae); marine; mesentery; metacestode; WTSA; Argentina, Brazil (Luque et al. 2002; Luque and Poulin 2004; Lanfranchi et al. 2009).

*Odontesthes regia* (Actinopterygii: Atherinopsidae); marine; site of infection not given; metacestode; WTSP; Chile (Torres et al. 1993).

Note: host reported as *Austromenidia laticlava*.

*Paralabrax humeralis* (Actinopterygii: Serranidae); marine; site of infection not given; metacestode; WTSP; Chile (Henríquez and González 2014).

*Paralichthys adspersus* (Actinopterygii: Paralichthyidae); marine; intestine; metacestode; WTSP; Chile (Riffo 1995).

*Paralichthys isosceles* (Actinopterygii: Paralichthyidae); marine; intestine, stomach; metacestode; WTSA; Argentina, Brazil (Luque and Poulin 2004; Alarcos and Timi 2012).

*Paralichthys microps* (Actinopterygii: Paralichthyidae); marine; intestine; metacestode; WTSP; Chile (Riffo 1995).

*Paralichthys patagonicus* (Actinopterygii: Paralichthyidae); marine; intestine, stomach; metacestode; WTSA; Argentina (Alarcos and Timi 2012).

*Paralonchurus brasiliensis* (Actinopterygii: Sciaenidae); marine; mesentery; metacestode; WTSA; Brazil (Ribeiro et al. 2002; Luque et al. 2003; Luque and Poulin 2004).

*Percophis brasiliensis* (Actinopterygii: Percophidae); marine; mesentery; metacestode; WTSA; Argentina, Brazil, Uruguay (Luque and Poulin 2004; Braicovich and Timi 2008, 2010).

Note: Luque and Poulin (2004) distinguished two morphotypes.

*Pomatomus saltatrix* (Actinopterygii: Pomatomidae); marine; site of infection not given; metacestode; WTSA; Brazil (Luque and Chaves 1999; Luque and Poulin 2004).

Notes: host reported as *Pomatomus saltator*. Luque and Poulin (2004) distinguished two different morphotypes.

*Prionotus punctatus* (Actinopterygii: Triglidae); marine; intestine; metacestode; WTSA; Brazil (Luque and Poulin 2004; Bicudo et al. 2005).

Note: Luque and Poulin (2004) distinguished two different morphotypes.

*Pseudopercis numida* (Actinopterygii: Pinguipedidae); marine; mesentery; metacestode; WTSA; Brazil (Luque et al. 2008).

*Pseudopercis semifasciata* (Actinopterygii: Pinguipedidae); marine; mesentery; metacestode; Magellanic, WTSA; Argentina (Timi and Lanfranchi 2009a).

*Raneya brasiliensis* (Actinopterygii: Ophidiidae); marine; site of infection not given; metacestode; Magellanic, WTSA; Argentina (Vales et al. 2011).

*Sarda chilensis* (Actinopterygii: Scombridae); marine; site of infection not given; metacestode; WTSP; Peru (Pérez et al. 1999).

*Sardinella brasiliensis* (Actinopterygii: Clupeidae); marine; body cavity; metacestode; WTSA; Brazil (Moreira et al. 2015).

*Sciaena deliciosa* (Actinopterygii: Scombridae); marine; body cavity, intestinal surface; metacestode; WTSP; Peru (Pérez et al. 1999; Llerena et al. 2001; Chero et al. 2014c).

*Scomber japonicus* (Actinopterygii: Scombridae); marine; site of infection not given; metacestode; WTSP; Chile (Rodríguez et al. 2000).

*Scomberomorus brasiliensis* (Actinopterygii: Scombridae); marine; mesentery; metacestode; WTSA; Brazil (Luque and Poulin 2004; Alves and Luque 2006).

*Selene setapinnis* (Actinopterygii: Carangidae); marine; mesentery; metacestode; WTSA; Brazil (Cordeiro and Luque 2004; Luque and Poulin 2004).

*Seriolella porosa* (Actinopterygii: Centrolophidae); marine; body cavity; metacestode; Magellanic; Argentina (Guagliardo et al. 2014).

*Sympterygia bonapartii* (Elasmobranchii: Arhynchobatidae); marine; spiral valve; adult; WTSA; Argentina (Ostrowski de Núñez 1971).

Note: host reported as *Psammobatis microps*.

*Trachurus lathami* (Actinopterygii: Carangidae); marine; mesentery; metacestode; WTSA; Brazil (Braicovich et al. 2012).

*Trachurus murphyi* (Actinopterygii: Carangidae); marine; body cavity, mesentery; metacestode; WTSP; Chile, Peru (Soto and Carvajal 1979; Oliva 1982, 1994, 1999; George-Nascimento and Arancibia 1992; Jara 1998; Pérez et al. 1999; George-Nascimento 2000; Rodríguez et al. 2000; George-Nascimento and Oliva 2015).

*Umbrina canosai* (Actinopterygii: Sciaenidae); marine; site of infection not given; metacestode; WTSA; Brazil (Luque and Poulin 2004).

*Urophycis brasiliensis* (Actinopterygii: Phycidae); marine; mesentery; metacestode; WTSA; Brazil (Alves et al. 2004; Luque and Poulin 2004).

Note: Alves et al. (2004) distinguished two different morphotypes.

*Urophycis mystaceus* (Actinopterygii: Phycidae); marine; mesentery; metacestode; WTSA; Brazil (Alves et al. 2002c; Luque and Poulin 2004).

*Xystreurys rasile* (Actinopterygii: Paralichthyidae); marine; intestine, stomach; metacestode; WTSA; Argentina (Alarcos and Timi 2012).

*Tentacularia coryphaenae* Bosc, 1797*

[For synonyms, see Palm (2004)]

*Carcharhinus longimanus* (Elasmobranchii: Carcharhinidae); marine; spiral valve; adult; TSA, WTSA; Brazil (Rego 1977; Knoff et al. 2002, 2004b).

*Carcharhinus obscurus* (Elasmobranchii: Carcharhinidae); marine; spiral valve; adult; WTSA; Brazil (Knoff et al. 2002, 2004b).

*Carcharodon carcharias* (Elasmobranchii: Lamnidae); marine; duodenum; adult; specific locality not given; Brazil (Dollfus 1942).

*Centropomus nigrescens* (Actinopterygii: Centropomidae); amphidromous; peritoneum; metacestode; WTSP; Peru (Escalante and Carvajal 1984).

*Coryphaena equiselis* (Actinopterygii: Coryphaenidae); marine; body cavity; metacestode; specific locality not given; Brazil (Rudolphi 1819; Dollfus 1942).

*Coryphaena hippurus* (Actinopterygii: Coryphaenidae); marine; body cavity, liver, gonads, muscles; metacestode; WTSA, WTSP; Brazil, Peru (Escalante and Carvajal 1984; López de McDonald and Tantaleán 1985; Silva and São Clemente 2001; Vásquez-Ruiz and Jara-Campos 2012).

Note: type host.

*Genypterus brasiliensis* (Actinopterygii: Ophidiidae); marine; mesentery; metacestode; WTSA; Brazil (São Clemente et al. 2004).

*Katsuwonus pelamis* (Actinopterygii: Scombridae); marine; abdominal flaps, body cavity, muscles; metacestode; WTSA, WTSP; Brazil, Peru (Escalante and Carvajal 1984; Amato et al. 1990; Alves and Luque 2006).

*Lophius gastrophysus* (Actinopterygii: Lophiidae); marine; muscles; metacestode; WTSA; Brazil (São Clemente et al. 2007).

*Merluccius gayi peruanus* (Actinopterygii: Merlucciidae); marine; mesentery; metacestode; WTSP; Peru (Durán and Oliva 1980; Jara 1998).

*Polyprion oxygeneios* (Actinopterygii: Polyprionidae); marine; peritoneal cavity; metacestode; JFD; Chile (Cattan et al. 1979).

*Prionace glauca* (Elasmobranchii: Carcharhinidae); marine; stomach, spiral valve; adult; JFD, WTSA, WTSP; Brazil, Chile, Peru (Cattan et al. 1979; Escalante 1986; Knoff et al. 2002, 2004b).

*Scomber japonicus* (Actinopterygii: Scombridae); marine; body cavity; metacestode; WTSP; Chile, Peru (Llerena et al. 2001; Oliva et al. 2008b).

*Scomberomorus cavalla* (Actinopterygii: Scombridae); marine; mesentery; metacestode; WTSA; Brazil (Dias et al. 2011a).

*Trachurus murphyi* (Actinopterygii: Carangidae); marine; mesentery; metacestode; WTSP; Chile, Peru (Soto and Carvajal 1979; Oliva 1982 ,1994, 1999; Pérez et al. 1999; Jara 1998).

Carangidae gen. sp. (Actinopterygii: Carangidae); marine; kidney, mesentery; metacestode; WTSP; Peru (Palm 2004).

Note: material is deposited in H. Palm’s personal collection.

**Unidentified tentaculariids**

*Dules auriga* (Actinopterygii: Serranidae); marine; mesentery; metacestode; WTSA; Argentina (Braicovich and Timi 2015).

*Paralichthys isosceles* (Actinopterygii: Paralichthyidae); marine; site of infection not given; metacestode; WTSA; Brazil (Alarcos et al. 2016).

*Urophycis brasiliensis* (Actinopterygii: Phycidae); marine; mesentery; metacestode; WTSA; Argentina, Brazil (Pereira et al. 2014).

*Xystreurys rasile* (Actinopterygii: Paralichthyidae); marine; intestine, mesentery; metacestode; WTSA; Argentina (Alarcos and Timi 2013).

**Unidentified trypanorhynchs**

*Antimora rostrata* (Actinopterygii: Moridae); marine; visceral cavity; metacestode; WTSP: Chile (Ñacari and Oliva 2016).

*Auxis thazard* (Actinopterygii: Scombridae); marine; body cavity; metacestode; WTSA; Brazil (Faria and Silva 1934).

*Balistes vetula* (Actinopterygii: Balistidae); marine; body cavity; metacestode; WTSA; Brazil (Cardoso et al. 2006).

*Brama australis* (Actinopterygii: Bramidae); marine; site of infection not given; metacestode; WTSP; Chile (George-Nascimento et al. 2002).

Note: George-Nascimento et al. (2002) distinguished two morphotypes.

*Caranx crysos* (Actinopterygii: Carangidae); marine; body cavity; metacestode; WTSA; Brazil (Cardoso et al. 2006).

*Carcharhinus limbatus* (Elasmobranchii: Carcharhinidae); marine; site of infection not given; adult; WTSA; Brazil (Faria and Silva 1934).

Note: host reported as *Carcharias limbatus*.

*Carcharodon carcharias* (Elasmobranchii: Lamnidae); marine; site of infection not given; adult; WTSA; Brazil (Faria and Silva 1934).

Note: host reported as *Carcharias lamia*.

*Centropomus undecimalis* (Actinopterygii: Centropomidae); amphidromous; intestine; metacestode; Paraíba do Sul River basin (estuary of Guandú River); Brazil (Azevedo et al. 2011).

*Coryphaenoides ariommus* (Actinopterygii: Macrouridae); marine; visceral cavity; metacestode; WTSP: Chile (Ñacari and Oliva 2016).

*Cynoscion acoupa* (Actinopterygii: Scianidae); marine; mesentery, peritoneum; metacestode; WTSA; Brazil (Faria and Silva 1934).

*Cynoscion jamaicensis* (Actinopterygii: Sciaenidae); marine; body cavity; metacestode; WTSA; Brazil (Pereira and Boeger 2005).

*Cynoscion leiarchus* (Actinopterygii: Scianidae); marine; mesentery; metacestode; WTSA; Brazil (Faria and Silva 1934).

*Cynoscion striatus* (Actinopterygii: Scianidae); marine; body cavity; metacestode; WTSA; Brazil (Faria and Silva 1934; Rego et al. 1974).

Note: Rego et al. (1974) distinguished two morphotypes.

*Cynoscion* sp. (Actinopterygii: Scianidae); marine; muscles; metacestode; TNA; Venezuela (Vogelsang and Mayaudon 1959).

Note: tapeworms reported as *Tetrarhynchus fragilis*.

*Dissostichus eleginoides* (Actinopterygii: Nototheniidae); marine; site of infection not given; metacestode; Magellanic; Falkland Islands (Brown et al. 2013).

*Epinephelus morio* (Actinopterygii: Serranidae); marine; liver, mesentery, muscles, peritoneum; metacestode; WTSA; Brazil (Faria and Silva 1934).

Note: host reported as *Cerna morio*.

*Epinephelus* sp. (Actinopterygii: Serranidae); marine; muscles; metacestode; TNA; Venezuela (Vogelsang and Mayaudon 1959).

Note: tapeworms reported as *Tetrarhynchus fragilis*.

*Euthynnus alletteratus* (Actinopterygii: Scombridae); marine; liver, mesentery, peritoneum; metacestode; WTSA; Brazil (Faria and Silva 1934).

Note: host reported as *Gymnosarda alleterada*.

*Genidens barbus* (Actinopterygii: Ariidae); marine; body cavity; metacestode; WTSA; Brazil (Cardoso et al. 2006).

Note: host reported as *Netuma barba*.

*Genidens* sp. (Actinopterygii: Ariidae); marine; mesentery; metacestode; WTSA; Brazil (Faria and Silva 1934).

Note: host reported as *Thachysurus* sp. (sic!)

*Genypterus blacodes* (Actinopterygii: Ophidiidae); marine; mesentery; metacestode; WTSP; Chile (Riffo 1994).

*Genypterus brasiliensis* (Actinopterygii: Ophidiidae); marine; mesentery; metacestode; WTSA; Argentina (Sardella et al. 1998).

*Genypterus maculatus* (Actinopterygii: Ophidiidae); marine; site of infection not given; metacestode; WTSP; Chile (Muñoz and George-Nascimento 2008).

*Helicolenus lengerichi* (Actinopterygii: Sebastidae); marine; intestinal serosa, mesentery, peritoneum, stomach serosa; WTSP; Chile (George-Nascimento and Iriarte 1989; Balboa and George-Nascimento 1998).

*Hyporthodus niveatus* (Actinopterygii: Serranidae); marine; liver, mesentery, peritoneum; metacestode; WTSA; Brazil (Faria and Silva 1934).

Note: host reported as *Garrupa niveata*.

*Lophius gastrophysus* (Actinopterygii: Lophiidae); marine; body cavity; metacestode; WTSA; Brazil (Cardoso et al. 2006).

*Lutjanus analis* (Actinopterygii: Lutjanidae); marine; body cavity; metacestode; TSA; Brazil (Hermida et al. 2014).

*Macrodon ancylodon* (Actinopterygii: Sciaenidae); marine; body cavity; metacestode; WTSA; Brazil (Pereira and Boeger 2005).

*Macrourus holotrachys* (Actinopterygii: Macrouridae); marine; visceral cavity; metacestode; WTSP; Chile (Ñacari and Oliva 2016).

*Masturus lanceolatus* (Actinopterygii: Molidae); marine; liver; metacestode; TSA (estuary of Una River); Brazil (Araújo et al. 2010).

*Micropogonias furnieri* (Actinopterygii: Scianidae); marine; body cavity, mesentery, peritoneum; metacestode; WTSA; Brazil, Uruguay (Faria and Silva 1934; Bertullo 1965; São Clemente 1986b; Pereira 1993; Pereira and Boeger 2005; Cardoso et al. 2006).

Notes: Faria and Silva (1934) reported the host as *Micropogon opercularis* and Bertullo (1965) reported the cestode as *Tetrarhynchus fragilis*.

*Micropogonias undulatus* (Actinopterygii: Scianidae); marine; mesentery, peritoneum; metacestode; WTSA; Brazil (Faria and Silva 1934).

Note: host reported as *Micropogon undulatus*.

*Mola mola* (Actinopterygii: Molidae); marine; oral cavity, heart, intestine, liver, muscles, stomach; metacestode; TSA; Brazil (Ahid et al. 2009).

*Mycteroperca bonaci* (Actinopterygii: Serranidae); marine; liver, mesentery, peritoneum; metacestode; WTSA; Brazil (Faria and Silva 1934).

Note: host reported as *Epinephelus bonaci*.

Notothenia cf. angustata (Actinopterygii: Nototheniidae); marine; body cavity; metacestode; WTSP; Chile (Muñoz et al. 2001).

*Orthopristis ruber* (Actinopterygii: Haemulidae); marine; mesentery, peritoneum; metacestode; WTSA; Brazil (Faria and Silva 1934).

*Pagrus pagrus* (Actinopterygii: Sparidae); marine; body cavity; metacestode; WTSA; Brazil (Cardoso et al. 2006).

*Paralabrax humeralis* (Actinopterygii: Serranidae); marine; site of infection not given; metacestode; WTSP; Peru (Iannacone and Alvariño 2009).

*Pomatomus saltatrix* (Actinopterygii: Pomatomidae); marine; mesentery, intestine; peritoneum; metacestode; WTSA; Brazil (Faria and Silva 1934; Travassos et al. 1967; Gomes et al. 1972; Luque and Chaves 1999).

Note: host reported as *Cheilodipterus saltator* and *Pomatomus saltator* by Faria and Silva (1934) and Luque and Chaves (1999), respectively.

*Porichthys porosissimus* (Actinopterygii: Batrachoididae); marine; liver, mesentery; metacestode; WTSA; Brazil (Faria and Silva 1934).

*Pseudopercis numida* (Actinopterygii: Pinguipedidae); marine; mesentery, peritoneum; metacestode; WTSA; Brazil (Faria and Silva 1934).

*Rhinobatos percellens* (Elasmobranchii: Rhinobatidae); marine; site of infection not given; adult; WTSA; Brazil (Faria and Silva 1934).

Note: host reported as 'cação viola', vernacular name of *Rhinobatos percellens*.

*Rhizoprionodon terraenovae* (Elasmobranchii: Carcharhinidae); marine; site of infection not given; adult; WTSA; Brazil (Faria and Silva 1934).

Note: host reported as 'cação alecrim' vernacular name of *Rhizoprionodon terraenovae*.

*Sardinella brasiliensis* (Actinopterygii: Clupeidae); marine; body cavity; metacestode; WTSA; Brazil (Cardoso et al. 2006).

*Sardinella* sp. (Actinopterygii: Clupeidae); marine; liver; metacestode; WTSA; Brazil (Feijó et al. 1979; Rodrigues et al. 1990).

*Scomber colias* (Actinopterygii: Scombridae); marine; body cavity, mesentery, peritoneum, stomach; metacestode; WTSA; Brazil (Faria and Silva 1934; Rego and Santos 1983).

*Selene vomer* (Actinopterygii: Carangidae); marine; mesentery, peritoneum; metacestode; WTSA; Brazil (Faria and Silva 1934).

*Trachurus murphyi* (Actinopterygii: Carangidae); marine; site of infection not given; metacestode; WTSP; Chile (George-Nascimento and Arancibia 1992; George-Nascimento 2000).

*Trichiurus lepturus* (Actinopterygii: Trichiuridae); marine; muscle; metacestode; WTSA; Brazil (Bueno et al. 2014).

*Urophycis brasiliensis* (Actinopterygii: Phycidae); marine; body cavity; metacestode; WTSA; Brazil (Cardoso et al. 2006).

Sciaenidae gen. sp. (Actinopterygii: Sciaenidae); site of infection not given; metacestode; WTSA; Brazil (Travassos et al. 1967).

Note: host reported as ‘pescadinha’, vernacular name of sciaenid fishes.

***Species inquirendae***

***Otobothrium cysticum* (Mayer, 1842) Dollfus, 1942**

[Syn. *Tetrarhynchus cysticus* Mayer, 1842]

*Genypterus brasiliensis* (Actinopterygii: Ophidiidae); marine; body cavity, mesentery; metacestode; WTSA; Brazil (São Clemente et al. 2004).

Note: Palm (2004) treated the type-species of the genus, *Otobothrium crenacolle* Linton, 1890, and *Otobothrium curtum* (Linton, 1909) as synonyms of *Otobothrium cysticum*, but Beverige and Justine (2007b) re-established those species and considered *Otobothrium cysticum* as *species inquirenda*.

*Pagrus pagrus* (Actinopterygii: Sparidae); marine; body cavity; metacestode; WTSA; Brazil (Palm 2004).

*Pomatomus saltatrix* (Actinopterygii: Pomatomidae); marine; body cavity; metacestode; WTSA; Brazil (Palm 2004).

Note: host reported as *Pomatomus saltator*.

*Scomberomorus brasiliensis* (Actinopterygii: Scombridae); marine; body cavity; metacestode; TSA; Brazil (Palm 1997).

Note: Palm (1997) reported the host as *Serrasalmus maculatus* (Mitchill).

*Sphyraena guachancho* (Actinopterygii: Sphyraenidae); marine; body cavity; metacestode; TSA; Brazil (Palm 1997).

***Pterobothrium fragile* (Diesing, 1850) Dollfus, 1942**

[Syns. *Synbothrium fragile* Diesing, 1850; *Syndesmobothrium fragile* (Diesing, 1850) Diesing, 1863; Pterobothrium (Synbothrium) fragile (Diesing, 1850) sensu Dollfus 1942]

*Pristis pristis* (Elasmobranchii: Pristidae); marine; intestine; adult; NBS (estuary of Amazon River); Brazil (Diesing 1850).

Notes: host reported as *Pterobothrium perotteti*. Type specimens collected by J. Natterer are poorly preserved and the validity of *Pterobothrium fragile* needs confirmation (see Campbell and Beveridge 1996).

***Pterobothrium interruptum* (Rudolphi, 1819) Diesing, 1850**

[Syn. *Anthocephalus interruptum* Rudolphi, 1819]

*Trichiurus lepturus* (Actinopterygii: Trichiuridae); marine; body cavity; metacestode; type locality not given; Brazil (Diesing 1850, 1856).

Note: the type material deposited in MNHB was destroyed during the World War II (Campbell and Beveridge 1996).

***Nomen nudum***

*Pterobothrium macrourum* (Rudolphi, 1819) Diesing, 1850*

[Syn. *Anthocephalus macrourus* Rudolphi, 1819]

Sparidae gen. sp. (Actinopterygii: Sparidae); marine; liver, mesentery; metacestode; type locality not given; Brazil (Rudolphi 1819, Diesing 1850).

Note: type specimens collected by Olfers are barely recognized as cestodes and basic taxonomic information is missing in the original description; therefore, Cambpell and Beveridge (1996) considered this taxon, type species of *Pterobothrium*, as *nomen nudum*.

**Unidentified cestodes**

*Acestrorhynchus altus* (Actinopterygii: Acestrorhynchidae); freshwater; intestine; stage of development not given; Paraná River basin; Brazil (Takemoto et al. 2009).

*Ageneiosus inermis* (Actinopterygii: Auchenipteridae); freshwater; site of infection and stage of development not given; Amazon and Paraná River basins; Brazil (Travassos et al. 1927; Travassos and Teixeira de Freitas 1964).

Note: hosts reported as *Ageneiosus valenciennesi* and *Pseudoageneiosus brevifilis* by Travassos and Teixeira de Freitas (1964) and Travassos et al. (1927), respectively.

*Astyanax altiparanae* (Actinopterygii: Characidae); freshwater; mesentery; metacestode; Paraná River basin; Brazil (Lizama et al. 2008).

*Astyanax bimaculatus* (Actinopterygii: Characidae); freshwater; site of infection and stage of development not given; Paraná River basin; Brazil (Travassos 1945).

*Astyanax* sp. (Actinopterygii: Characidae); freshwater; site of infection and stage of development not given; Amazon River basin; Brazil (Travassos and Teixeira de Freitas 1964).

*Australoheros facetus* (Actinopterygii: Cichlidae); freshwater; site of infection and stage of development not given; Doce River basin; Brazil (Travassos and Teixeira de Freitas 1948).

*Brachyplatystoma* sp. (Actinopterygii: Pimelodidae); freshwater; site of infection and stage of development not given; Paraná River basin; Brazil (Travassos and Teixeira de Freitas 1942, 1943).

*Brama australis* (Actinopterygii: Bramidae); marine; site of infection not given; metacestode; WTSP; Chile (George-Nascimento et al. 2002).

*Brevoortia aurea* (Actinopterygii: Clupeidae); marine; site of infection not given; metacestode; WTSA; Argentina (Alarcos and Etchegoin 2010).

Notes: host collected in a coastal lagoon.

*Calophysus macropterus* (Actinopterygii: Pimelodidae); freshwater; site of infection and stage of development not given; Amazon River basin; Brazil (Travassos and Teixeira de Freitas 1964).

*Caranx hippos* (Actinopterygii: Carangidae); brackish, marine; site of infection and stage of development not given; TSA; Brazil (Travassos et al. 1967).

*Centropomus* sp. (Actinopterygii: Centropomidae); amphidromous; site of infection and stage of development not given; Doce River basin; Brazil (Travassos and Teixeira de Freitas 1948).

*Cetopsis coecutiens* (Actinopterygii: Cetopsidae); freshwater; site of infection and stage of development not given; Amazon River basin; Brazil (Travassos and Teixeira de Freitas 1964).

*Cichla ocellaris* (Actinopterygii: Cichlidae); freshwater; site of infection not given; adult; Pereira de Miranda fishpond, Ceará State; Brazil (Kohn et al. 2004).

*Cichlasoma bimaculatum* (Actinopterygii: Cichlidae); freshwater; site of infection not given; adult; Pereira de Miranda fishpond, Ceará State, Paraná River basin; Brazil (Travassos 1940; Kohn et al. 2004).

*Colossoma macropomum* (Actinopterygii: Serrasalmidae); freshwater; site of infection not given; metacestode; Paraná River basin; Brazil (Kohn et al. 1985).

*Coryphaena hippurus* (Actinopterygii: Coryphaenidae); brackish, marine; site of infection and stage of development not given; TSA; Brazil (Travassos et al. 1967).

*Crenicichla haroldoi* (Actinopterygii: Cichlidae); freshwater; intestine; adult (immature); Paraná River basin; Brazil (Kohn et al. 2011).

*Cynoscion guatucupa* (Actinopterygii: Sciaenidae); marine; site of infection not given; metacestode; WTSA; Argentina (Timi et al. 2010b).

*Dules auriga* (Actinopterygii: Serranidae); marine; mesentery; metacestode; WTSA; Argentina (Braicovich and Timi 2015).

*Galaxias maculatus* (Actinopterygii: Galaxiidae); amphidromous; intestine; metacestode; Maullin River basin; Chile (Bravo et al. 2007).

*Galeocharax knerii* (Actinopterygii: Characidae); freshwater; intestine; adult (immature); Paraná River basin; Brazil (Kohn et al. 2011).

*Geophagus brasiliensis* (Actinopterygii: Characidae); freshwater; site of infection and stage of development not given; Doce River basin; Brazil (Travassos 1944; Travassos and Teixeira de Freitas 1948).

*Gymnocharacinus bergii* (Actinopterygii: Characidae); freshwater; site of infection not given; metacestode; Negro River Basin; Argentina (Ortubay et al. 1994).

*Gymnotus carapo* (Actinopterygii: Gymnotidae); freshwater; site of infection and stage of development not given; Doce River basin; Brazil (Travassos and Teixeira de Freitas 1948).

Note: host reported as *Giton fasciatus*.

*Gymnotus inaequilabiatus* (Actinopterygii: Gymnotidae); freshwater; intestine; stage of development not given; Paraná River basin; Brazil (Takemoto et al. 2009).

*Harengula* sp. (Actinopterygii: Clupeidae); marine; site of infection and stage of development not given; TSA; Brazil (Travassos et al. 1967).

*Hemisorubim platyrhynchos* (Actinopterygii: Pimelodidae); freshwater; mesentery, intestine; metacestode, adult; Amazon and Paraná River basins; Brazil, Peru (Travassos and Teixeira de Freitas 1942, 1943; Kohn et al. 2011).

*Hoplerythrinus unitaeniatus* (Actinopterygii: Erythrinidae); freshwater; site of infection and stage of development not given; Paraná River basin; Brazil (Travassos 1940; Travassos and Teixeira de Freitas 1943).

*Hoplias malabaricus* (Actinopterygii: Erythrinidae); freshwater; site of infection and stage of development not given; Paraná River basin; Brazil (Travassos 1940).

*Lagocephalus laevigatus* (Actinopterygii: Tetraodontidae); brackish, marine; site of infection and stage of development not given; TSA; Brazil (Travassos et al. 1967).

*Leporinus obtusidens* (Actinopterygii: Anostomidae); freshwater; intestine; adult; Paraná River basin; Brazil (Kohn et al. 2011).

*Loricariichthys* sp. (Actinopterygii: Loricariidae); freshwater; intestine; adult (immature); Paraná River basin; Brazil (Kohn et al. 2011).

*Lutjanus jocu* (Actinopterygii: Lutjanidae); brackish, freshwater; marine; site of infection and stage of development not given; TSA; Brazil (Travassos et al. 1967).

*Macrodon ancylodon* (Actinopterygii: Sciaenidae); marine; mesentery, muscles; metacestode; WTSA; Brazil (Oliveira 1985).

*Merluccius australis* (Actinopterygii: Merlucciidae); marine; mesentery, muscles; metacestode; WTSP; Chile (Fernández 1985).

*Micropogonias furnieri* (Actinopterygii: Sciaenidae); marine; mesentery, muscles; metacestode; WTSA; Brazil (Oliveira 1985).

*Micropogonias* sp. (Actinopterygii: Sciaenidae); marine; site of infection and stage of development not given; TSA; Brazil (Travassos et al. 1967).

*Oligoplites saurus* (Actinopterygii: Carangidae); brackish, marine; site of infection and stage of development not given; TSA; Brazil (Travassos et al. 1967).

*Oreochromis niloticus* (Actinopterygii: Cichlidae); freshwater; site of infection not given; metacestode; Pereira de Miranda fishpond, Ceará and Paraná States; Brazil (Kohn et al. 2004; Graça and Machado 2007).

Note: introduced fish host (Froese and Pauly 2016).

*Paralichthys isosceles* (Actinopterygii: Paralichthyidae); marine; site of infection not given; metacestode; WTSA; Brazil (Alarcos et al. 2016).

*Piaractus mesopotamicus* (Actinopterygii: Characidae); freshwater; site of infection and stage of development not given; Paraná River basin; Brazil (Kohn et al. 2011).

*Pimelodella gracilis* (Actinopterygii: Heptapteridae); freshwater; intestine, mesentery; adult, metacestode; Amazon and Paraná River basins; Brazil (Kohn et al. 2011).

*Pimelodella lateristriga* (Actinopterygii: Heptapteridae); freshwater; intestine; adult; Paraná River basin; Brazil (Kohn et al. 2011).

*Pimelodus maculatus* (Actinopterygii: Pimelodidae); freshwater; intestine; adult, metacestode; Paraná River basin; Brazil (Travassos and Teixeira de Freitas 1940, 1943; Travassos 1947; Kohn et al. 1985; Kohn and Fernandes 1987).

Notes: host reported as *Pimelodus clarias* by all authors.

*Pimelodus ortmanni* (Actinopterygii: Pimelodidae); freshwater; intestine; metacestode; Paraná River basin; Brazil (Kohn et al. 1988).

*Pinirampus pirinampu* (Actinopterygii: Pimelodidae); freshwater; site of infection and stage of development not given; Paraná River basin; Brazil (Travassos et al. 1927).

*Plagioscion squamosissimus* (Actinopterygii: Sciaenidae); freshwater; mesentery, intestine; metacestode, adult (immature); Amazon, Paraná and Tocantins-Araguaia River basins; Brazil (Kohn et al. 2011; Lacerda et al. 2012).

*Poecilia vivipara* (Actinopterygii: Poeciliidae); freshwater; site of infection not given; adult; Pereira de Miranda fishpond, Ceará State; Brazil (Kohn et al. 2004).

*Potamotrygon motoro* (Elasmobranchii: Potamotrygonidae); freshwater; intestine; adult; Paraná River basin; Brazil (Travassos and Teixeira de Freitas 1940, 1941, 1942, 1943; Kohn et al. 2011).

Notes: Travassos and Teixeira de Freitas (1940, 1941, 1942, 1943) did not specify the site of infection and the stage of development; they also reported the host as *Ellipesurus motoro*.

*Potamotrygon orbignyi* (Elasmobranchii: Potamotrygonidae); freshwater; site of infection and stage of development not given; Amazon River basin; Brazil (Travassos and Teixeira de Freitas 1964).

Note: host reported as *Paratrygon hystrix*.

*Prochilodus argenteus* (Actinopterygii: Prochilodontidae); freshwater; intestinal serosa; metacestode; São Francisco River Basin; Brazil (Monteiro et al. 2009).

*Pseudopercis semifasciata* (Actinopterygii: Pinguipedidae); marine; stomach wall; metacestode; Magellanic, WTSA; Argentina (Timi and Lanfranchi 2009a).

*Pseudoplatystoma fasciatum* (Actinopterygii: Pimelodidae); freshwater; intestine; adult; Paraná River basin; Brazil (Campos et al. 2008, 2009b).

*Pseudoplatystoma* sp. (Actinopterygii: Pimelodidae); freshwater; site of infection and stage of development not given; Amazon and Paraná River basins; Brazil (Travassos et al. 1927; Travassos 1940; Travassos and Teixeira de Freitas 1964).

Note: Travassos et al. (1927) reported the host as *Pseudoplatystoma tigrinum*, which does not occur in the Paraná River basin (Froese and Pauly 2016).

*Raneya brasiliensis* (Actinopterygii: Ophidiidae); marine; mesentery; metacestode; Magellanic, WTSA; Argentina (Vales et al. 2011).

*Rhaphiodon vulpinus* (Actinopterygii: Cynodontidae); freshwater; site of infection and stage of development not given; Paraná River basin; Brazil (Travassos and Teixeira de Freitas 1942).

*Salilota australis* (Actinopterygii: Moridae); marine; body cavity; metacestode; Magellanic; Argentina (Szidat 1961).

*Salminus brasiliensis* (Actinopterygii: Bryconidae); freshwater; intestine; adult (immature); Paraná River basin; Brazil (Ceccarrelli et al. 2006; Kohn et al. 2011).

*Scomber colias* (Actinopterygii: Scombridae); brackish, marine; site of infection

and stage of development not given; TSA; Brazil (Travassos et al. 1967).

*Selene vomer* (Actinopterygii: Carangidae); brackish, marine; site of infection and stage of development not given; TSA; Brazil (Travassos et al. 1967).

*Sphoeroides testudineus* (Actinopterygii: Tetraodontidae); brackish, marine; site of infection and stage of development not given; TSA; Brazil (Travassos et al. 1967).

*Steindachneridion parahybae* (Actinopterygii: Pimelodidae); freshwater; site of infection and stage of development not given; Paraná River basin; Brazil (Travassos and Teixeira de Freitas 1943; Kohn and Fernandes 1987).

Note: this fish is found only in the Paraíba do Sul and Jequitinhonha River basins (Froese and Pauly 2016).

*Sternopygus macrurus* (Actinopterygii: Sternopygidae); freshwater; intestine; adult; Paraná River basin; Brazil (Kohn et al. 2011).

*Tilapia rendalli* (Actinopterygii: Cichlidae); freshwater; intestine; metacestode; Ingá Lake, Paraná State, Brazil (Graça and Machado 2007).

*Trachurus murphyi* (Actinopterygii: Carangidae); marine; body cavity; metacestode; WTSP; Chile (George-Nascimento and Oliva 2015).

*Umbrina coroides* (Actinopterygii: Sciaenidae); brackish, marine; site of infection and stage of development not given; TSA; Brazil (Travassos et al. 1967).

*Xystreurys rasile* (Actinopterygii: Paralichthyidae); marine; mesentery; metacestode; WTSA; Argentina (Alarcos and Timi 2012, 2013).

*Zungaro jahu* (Actinopterygii: Pimelodidae); freshwater; site of infection and stage of development not given; Paraná River basin; Brazil (Travassos 1940, 1947; Travassos and Teixeira de Freitas 1942, 1943).

Note: host reported as *Paulicea luetkeni*.

## Host-Parasite List

### Phylum Chordata

**Class Actinopterygii**

**Order Anguilliformes**

Family Congridae

***Conger orbignianus*** Valenciennes: Grillotia (Christianella) carvajalregorum (L), Grillotia (Grillotia) erinaceus (L), *Nybelinia* sp. (L), ‘*Scolex* spp.’ (L)

Family Muraenidae

***Gymnothorax moringa*** (Cuvier): ‘*Scolex* spp.’ (L)

**Order Atheriniformes**

Family Atherinopsidae

***Basilichthys australis*** Eigenmann: *Diphyllobothrium dendriticum* (L), *Diphyllobothrium latum* (L), *Diphyllobothrium* sp. (L).

***Odontesthes argentinensis*** (Valenciennes): ‘*Scolex* spp.’ (L)

***Odontesthes bonariensis*** (Valenciennes): *Cangatiella macdonaghi*

***Odontesthes hatcheri*** (Eigenmann): *Cangatiella macdonaghi*

***Odontesthes mauleanum*** (Steindachner): *Diphyllobothrium dendriticum* (L), *Diphyllobothrium latum* (L), *Diphyllobothrium* sp. (L).

***Odontesthes regia*** (Humboldt): *Diphyllobothrium* sp. (L), *Lacistorhynchus tenuis* (L), *Nybelinia* sp. (L), unidentified ‘pseudophyllidean’ (L), ‘*Scolex* spp.’ (L)

***Odontesthes smitti*** (Lahille): unidentified bothriocephalidean (L), ‘*Scolex* spp.’ (L)

**Order Aulopiformes**

Family Synodontidae

***Synodus scituliceps*** Jordan & Gilbert: *Rhinebothrium* sp. (L), ‘*Scolex* spp.’ (L)

**Order Batrachoidiformes**

Family Batrachoididae

***Aphos porosus*** (Valenciennes): *Clestobothrium crassiceps*, *Grillotia* sp. (L), *Nybelinia* sp. (L), unidentified ‘pseudophyllidean’ (L), ‘*Scolex* spp.’ (L)

***Porichthys porosissimus*** (Cuvier): Grillotia (Christianella) carvajalregorum (L), Grillotia (Grillotia) erinaceus (?) (L), *Nybelinia lingualis* (L), unidentified trypanorhynch (L), ‘*Scolex* spp.’ (L)

**Order Beloniformes**

Family Belonidae

***Strongylura scapularis*** (Jordan & Gilbert): ‘*Scolex* spp.’ (L)

***Tylosurus acus acus*** (Lacépède): ‘*Scolex*’ spp. (L)

Family Hemiramphidae

***Hyporhamphus unifasciatus*** (Ranzani): ‘*Scolex* spp.’ (L)

**Order Characiformes**

Family Acestrorhynchidae

***Acestrorhynchus altus*** Menezes: *Monticellia dlouhyi*, unidentified cestode

Family Anostomidae

***Leporellus vittatus*** (Valenciennes): unidentified proteocephalid

***Leporinus friderici*** (Bloch): *Proteocephalus vazzolerae*, unidentified proteocephalid

***Leporinus lacustris*** Amaral Campos: *Proteocephalus vazzolerae*

***Leporinus obtusidens*** (Valenciennes): unidentified cestode

Family Bryconidae

***Brycon cephalus*** (Günther): unidentified proteocephalid

***Brycon orbignyanus*** (Valenciennes): *Monticellia* sp.

***Salminus brasiliensis*** (Cuvier): *Monticellia coryphicephala*, unidentified cestode

***Salminus franciscanus*** Lima & Britski: *Monticellia coryphicephala*

Family Characidae

***Aphyocharax anisitsi*** Eigenmann & Kennedy: unidentified proteocephalid

***Astyanax altiparanae*** Garutti & Britski: *Senga* sp., unidentified proteocephalid, unidentified cestode

***Astyanax bimaculatus*** (Linnaeus): unidentified cestode

***Astyanax scabripinnis*** (Jenyns): *Senga* sp.

***Astyanax* sp.**: unidentified cestode

***Galeocharax knerii*** (Steindachner): unidentified cestode

***Gymnocharacinus bergii*** Steindachner: unidentified cestode

***Psellogrammus kennedyi*** (Eigenmann): unidentified proteocephalid

Family Cynodontidae

***Rhaphiodon vulpinus*** Spix & Agassiz: *Choanoscolex abscisus*, unidentified cestode

Family Erythrinidae

***Hoplerythrinus unitaeniatus*** (Spix & Agassiz): *Nomimoscolex matogrossensis* (?), *Proteocephalus mahnerti*, unidentified cestode

***Hoplias malabaricus*** (Bloch): *Nomimoscolex matogrossensis*, *Proteocephalus regoi*, unidentified cestode

Family Prochilodontidae

***Prochilodus argenteus*** Spix & Agassiz: *Valipora* sp. (L), unidentified cestode

***Prochilodus brevis*** Steindachner: unidentified proteocephalid

***Prochilodus lineatus*** (Valenciennes): unidentified proteocephalid, *Valipora campylancristrota* (L)

Family Serrasalmidae

***Colossoma macropomum*** (Cuvier): unidentified proteocephalid, unidentified cestode

***Colossoma macropomum* × *Piaractus mesopotamicus***: unidentified proteocephalid

***Piaractus brachypomus*** (Cuvier): unidentified proteocephalid

***Piaractus mesopotamicus*** (Holmberg): *Proteocephalus vazzolerae*, unidentified cestode

***Pygocentrus nattereri*** Kner: *Proteocephalus serrasalmus*

***Serrasalmus maculatus*** Kner: *Proteocephalus serrasalmus*

**Order Clupeiformes**

Family Clupeidae

***Brevoortia aurea*** (Spix & Agassiz): unidentified cestode

***Ethmidium maculatum*** (Valenciennes): ‘*Scolex* spp.’ (L)

***Harengula clupeola*** (Cuvier): *Callitetrarhynchus gracilis* (L)

***Harengula* sp.**: unidentified cestode

***Opisthonema oglinum*** (Lesueur): *Callitetrarhynchus gracilis* (L)

***Sardinella brasiliensis*** (Steindachner): *Callitetrarhynchus gracilis* (L), *Nybelinia* sp. (L), unidentified trypanorhynch, ‘*Scolex* spp.’ (L)

***Sardinella* sp.**: unidentified proteocephalid, unidentified trypanorhynch

***Sardinops sagax*** (Jenyns): ‘*Scolex* spp.’ (L)

**Clupeidae gen. sp.**: *Pterobothrium heteracanthum* (L)

Family Engraulidae

***Anchoa tricolor*** (Spix & Agassiz): ‘*Scolex* spp.’ (L)

***Engraulis anchoita*** Hubbs & Marini: *Bothriocephalus* sp., unidentified ‘pseudophyllidean’, ‘*Scolex* spp.’ (L)

***Engraulis ringens*** Jenyns: *Bothriocephalus* sp., *Diphyllobothrium* sp. (L), ‘*Scolex* spp.’ (L)

**Order Cypriniformes**

Family Cyprinidae

***Cyprinus carpio*** Linnaeus: *Schyzocotyle acheilognathi*, unidentified caryophyllidean, unidentified proteocephalid

***Hypophthalmichthys nobilis*** (Richardson): unidentified proteocephalid

***Pethia conchonius*** (Hamilton): *Schyzocotyle acheilognathi*

**Order Cyprinodontiformes**

Family Poeciliidae

***Poecilia reticulata*** Peters: *Glossocercus auritus* (L), *Schyzocotyle acheilognathi*

***Poecilia vivipara*** Bloch & Schneider: unidentified cestode

***Xiphophorus hellerii*** Heckel: *Schyzocotyle acheilognathi*

***Xiphophorus maculatus*** (Günther): *Schyzocotyle acheilognathi*

**Order Gadiformes**

Family Gadidae

***Micromesistius australis australis*** Norman: *Clestobothrium crassiceps*, *Diphyllobothrium* sp. (L), *Grillotia* sp. (L), *Hepatoxylon trichiuri* (L), *Hepatoxylon* sp. (L), unidentified ‘pseudophyllidean’

Family Macrouridae

***Coelorinchus chilensis*** Gilbert & Thompson: *Grillotia* sp. (L), *Hepatoxylon trichiuri* (L)

***Coryphaenoides ariommus*** Gilbert & Thompson: unidentified trypanorhynch

***Macrourus carinatus*** (Günther): Grillotia (Grillotia) borealis (?) (L)

***Macrourus holotrachys*** Günther: *Hepatoxylon* sp. (L), *Parabothriocephalus* sp. (L),

unidentified trypanorhynch

Family Merlucciidae

***Macruronus magellanicus*** Lönnberg: *Clestobothrium crassiceps*, Grillotia (Grillotia) dollfusi (L), *Grillotia heptanchi* (L), *Hepatoxylon trichiuri* (L), unidentified ‘pseudophyllidean’, ‘*Scolex* spp.’ (L)

***Merluccius australis*** (Hutton): *Clestobothrium splendidum*, *Diphyllobothrium* sp. (L), *Grillotia heptanchi* (L), *Grillotia* sp. (L), *Hepatoxylon trichiuri* (L), *Lacistorhynchus* sp. (L), unidentified phyllobothriidean, ‘*Scolex* spp.’ (L), unidentified cestode

***Merluccius gayi gayi*** (Guichenot): *Clestobothrium crassiceps*, Grillotia (Grillotia) dollfusi (L), *Grillotia heptanchi* (L), *Hepatoxylon trichiuri* (L), *Nybelinia surmenicola* (L), unidentified ‘pseudophyllidean’, ‘*Scolex* spp.’ (L)

***Merluccius gayi peruanus*** (Ginsburg): *Adenocephalus pacificus* (L), *Callitetrarhynchus gracilis* (L), *Clestobothrium crassiceps*, *Diphyllobothrium* sp. (L), Grillotia (Grillotia) dollfusi (L), *Lacistorhynchus tenuis* (L), *Nybelinia* sp. (L), *Tentacularia coryphaenae* (L)

***Merluccius hubbsi*** Marini: *Clestobothrium cristinae*, *Diphyllobothrium* sp. (L), Grillotia (Christianella) carvajalregorum (L), *Grillotia* sp. (L), *Hepatoxylon trichiuri* (L), *Nybelinia* sp. (L), unidentified phyllobothriidean, unidentified ‘pseudophyllidean’, ‘*Scolex* spp.’ (L)

***Merluccius* sp.**: *Clestobothrium crassiceps*

Family Moridae

***Antimora rostrata*** (Günther): unidentified trypanorhynch

***Salilota australis*** (Günther): Grillotia (Grillotia) borealis (?) (L), Grillotia (Grillotia) patagonica (L), *Hepatoxylon trichiuri* (L), unidentified cestode

Family Phycidae

***Urophycis brasiliensis*** (Kaup): *Callitetrarhynchus gracilis* (L), Grillotia (Christianella) carvajalregorum (L), *Heteronybelinia* sp. (L), *Lacistorhynchus* sp. (L), *Nybelinia* sp. (L), *Phyllobothrium* sp. (L), unidentified phyllobothriidean, unidentified trypanorhynch, ‘*Scolex* spp.’ (L)

***Urophycis mystaceus*** Ribeiro: *Lacistorhynchus* sp. (L), *Nybelinia* sp. (L), unidentified phyllobothriidean, ‘*Scolex* spp.’ (L)

***Urophycis* sp.**: unidentified phyllobothriidean

**Order Gobiesociformes**

Family Gobiesocidae

***Gobiesox marmoratus*** Jenyns: unidentified ‘pseudophyllidean’, ‘*Scolex* spp.’ (L)

***Sicyases sanguineus*** Müller & Troschel: unidentified ‘pseudophyllidean’, ‘*Scolex* spp.’ (L)

**Order Gymnotiformes**

Family Gymnotidae

***Gymnotus carapo*** Linnaeus: *Nomimoscolex chubbi*, *Nomimoscolex dechambrieri*, *Nomimoscolex guillermoi*, *Proteocephalus* sp., unidentified cestode

***Gymnotus inaequilabiatus*** (Valenciennes): unidentified cestode

***Gymnotus* sp.**: *Nomimoscolex chubbi*

Family Sternopygidae

***Sternopygus macrurus*** (Bloch & Schneider): unidentified cestode

**Order Lampriformes**

Family Lampridae

***Lampris guttatus*** (Brünnich): *Hepatoxylon trichiuri* (L)

**Order Lophiiformes**

Family Lophiidae

***Lophius gastrophysus*** Miranda Ribeiro: *Diphyllobothrium* sp. (L), Grillotia (Christianella) carvajalregorum (L), *Mixonybelinia* sp. (L), *Nybelinia* sp. (L), *Tentacularia coryphaenae* (L), unidentified trypanorhynch

**Ordem Mugiliformes**

Family Mugilidae

***Mugil cephalus*** Linnaeus: *Lacistorhynchus tenuis* (L), ‘*Scolex* spp.’ (L)

***Mugil liza*** Valenciennes: unidentified phyllobothriidean, ‘*Scolex* spp.’ (L)

**Order Notacanthiformes**

Family Notacanthidae

***Notacanthus sexspinis*** Richardson: *Hepatoxylon trichiuri* (L)

**Order Ophidiiformes**

Family Ophidiidae

***Genypterus blacodes*** (Forster): *Anonchocephalus chilensis*, *Hepatoxylon trichiuri* (L), unidentified trypanorhynch, ‘*Scolex* spp.’ (L)

***Genypterus brasiliensis*** Regan: *Anonchocephalus chilensis*, *Callitetrarhynchus gracilis* (L), *Diphyllobothrium* sp. (L), Grillotia (Christianella) carvajalregorum (L), *Hepatoxylon trichiuri* (L), *Heteronybelinia nipponica* (L), *Mixonybelinia beveridgei* (L), *Nybelinia* sp. (L), *Otobothrium cysticum* (L), *Tentacularia coryphaenae* (L), unidentified trypanorhynch, ‘*Scolex* spp.’ (L)

***Genypterus chilensis*** (Guichenot): *Anonchocephalus chilensis*, *Grillotia heptanchi* (L), *Hepatoxylon trichiuri* (L)

***Genypterus maculatus*** (Tschudi): *Adenocephalus pacificus* (L), *Anonchocephalus chilensis*, *Diphyllobothrium* sp. (L), *Hepatoxylon trichiuri* (L), *Nybelinia* sp. (L), unidentified trypanorhynch, ‘*Scolex* spp.’

***Raneya brasiliensis*** (Kaup): Grillotia (Christianella) carvajalregorum (L), *Heteronybelinia mattisi* (L), *Nybelinia* sp. (L), ‘*Scolex* spp.’ (L), unidentified cestode

**Order Osmeriformes**

Family Galaxiidae

***Aplochiton taeniatus*** Jenyns: unidentified bothriocephalidean

***Aplochiton zebra*** Jenyns: *Ailinella mirabilis*

***Galaxias maculatus*** (Jenyns): *Ailinella mirabilis*, *Diphyllobothrium dendriticum* (L), *Diphyllobothrium latum* (L), *Diphyllobothrium* sp. (L), unidentified cestode

***Galaxias platei*** Steindachner: *Diphyllobothrium latum* (L), *Diphyllobothrium* sp. (L), *Galaxitaenia toloi*

**Order Osteoglossiformes**

Family Arapaimidae

***Arapaima gigas*** (Schinz): *Nesolecithus janickii*, *Schizochoerus liguloideus*

**Order Perciformes**

Family Blenniidae

***Hypsoblennius sordidus*** (Bennett): unidentified ‘pseudophyllidean’

***Scartichthys viridis*** (Valenciennes): *Lacistorhynchus* sp. (L), unidentified ‘pseudophyllidean’

Family Bovichtidae

***Cottoperca gobio*** (Günther): *Bothriocephalus timii*, Grillotia (Grillotia) patagonica (L)

Family Bramidae

***Brama australis*** Valenciennes: *Hepatoxylon trichiuri* (L), *Nybelinia* sp. (L), unidentified trypanorhynch, unidentified cestode

***Brama japonica*** Hilgendorf: *Hepatoxylon trichiuri* (L), *Nybelinia* sp. (L)

Family Carangidae

***Caranx crysos*** (Mitchill): *Callitetrarhynchus gracilis* (L), unidentified trypanorhynch

***Caranx hippos*** (Linnaeus): *Callitetrarhynchus gracilis* (L), *Dasyrhynchus giganteus* (L), *Nybelinia* sp. (L), unidentified cestode

***Caranx latus*** Agassiz: *Callitetrarhynchus gracilis* (L), *Nybelinia* sp. (L), ‘*Scolex* spp.’ (L)

***Chloroscombrus chrysurus*** (Linnaeus): *Callitetrarhynchus gracilis* (L)

***Oligoplites palometa*** (Cuvier): *Callitetrarhynchus gracilis* (L), *Pterobothrium crassicole* (L), ‘*Scolex* spp.’ (L)

***Oligoplites saliens*** (Bloch): *Dasyrhynchus giganteus* (L), ‘*Scolex* spp.’ (L)

***Oligoplites saurus*** (Bloch & Schneider): *Callitetrarhynchus gracilis* (L), ‘*Scolex* spp.’ (L), unidentified cestode

***Parona signata*** (Jenyns): Grillotia (Christianella) carvajalregorum (L), ‘*Scolex* spp.’ (L)

***Selene setapinnis*** (Mitchill): *Callitetrarhynchus gracilis* (L), *Nybelinia* sp. (L)

***Selene vomer*** (Linnaeus): *Callitetrarhynchus gracilis* (L), *Nybelinia lingualis* (L), unidentified trypanorhynch, unidentified cestode

***Seriola lalandi*** Valenciennes: *Floriceps saccatus* (L)

***Trachinotus paitensis*** Cuvier: *Adenocephalus pacificus* (L)

***Trachurus lathami*** Nichols: *Callitetrarhynchus gracilis* (L), Grillotia (Christianella) carvajalregorum (L), *Nybelinia* sp. (L), unidentified ‘pseudophyllidean’, ‘*Scolex* spp.’ (L)

***Trachurus murphyi*** Nichols: *Adenocephalus pacificus* (L), *Diphyllobothrium* sp. (L), *Hepatoxylon trichiuri* (L), *Hepatoxylon* sp. (L), *Nybelinia lingualis* (L), *Nybelinia surmenicola* (L), *Nybelinia* sp. (L), *Tentacularia coryphaenae* (L), unidentified ‘pseudophyllidean’, unidentified trypanorhynch, ‘*Scolex* spp.’ (L), unidentified cestode

**Carangidae gen. sp.**: *Tentacularia coryphaenae* (L)

Family Centrolophidae

***Seriolella porosa*** Guichenot: *Nybelinia* sp. (L).

***Seriolella violacea*** Guichenot: *Adenocephalus pacificus* (L), *Neobothriocephalus aspinosus*

Family Centropomidae

***Centropomus nigrescens*** Günther: *Floriceps saccatus* (L), *Tentacularia coryphaenae* (L)

***Centropomus undecimalis*** (Bloch): *Callitetrarhynchus gracilis* (L), unidentified trypanorhynch

***Centropomus* sp.**: unidentified cestode

Family Cheilodactylidae

***Cheilodactylus variegatus*** Valenciennes: *Lacistorhynchus tenuis* (L)

***Nemadactylus bergi*** (Norman): Grillotia (Christianella) carvajalregorum (L), Grillotia (Grillotia) patagonica (L), *Heteronybelinia mattisi* (L)

Family Cichlidae

***Aequidens tetramerus*** (Heckel): unidentified proteocephalid

***Astronotus ocellatus*** (Agassiz): *Proteocephalus gibsoni*, unidentified proteocephalid

***Astronotus* sp.**: *Proteocephalus gibsoni*

***Australoheros facetus*** (Jenyns): *Parvitaenia macropeos* (L), unidentified cestode

***Cichla kelberi*** Kullander & Ferreira: *Proteocephalus macrophallus*, *Proteocephalus microscopicus*, *Sciadocephalus megalodiscus*

***Cichla monoculus*** Spix: *Proteocephalus macrophallus*, *Proteocephalus microscopicus*, *Sciadocephalus megalodiscus*, unidentified bothriocephalidean

***Cichla ocellaris*** Schneider: *Proteocephalus macrophallus*, *Proteocephalus microscopicus*, unidentified proteocephalid, unidentified cestode

***Cichla piquiti*** Kullander & Ferreira: *Proteocephalus macrophallus*, *Proteocephalus microscopicus*, *Sciadocephalus megalodiscus*

***Cichla* sp.**: *Proteocephalus macrophallus*, *Proteocephalus microscopicus*

***Cichlasoma amazonarum*** Kullander: unidentified proteocephalid (new species and genus, see the parasite-host list)

***Cichlasoma bimaculatum*** (Linnaeus): unidentified cestode

***Crenicichla britskii*** Kullander: *Valipora* sp. (L)

***Crenicichla haroldoi*** Luengo & Britski: unidentified cestode

***Crenicichla lepidota*** Heckel: unidentified proteocephalid

***Geophagus brasiliensis*** (Quoy & Gaimard): unidentified caryophyllidean, *Proteocephalus gibsoni*, *Valipora campylancristrota* (L), unidentified proteocephalid, unidentified cestode

***Geophagus proximus*** (Castelnau): unidentified proteocephalid

***Laetacara curviceps*** (Ahl): unidentified proteocephalid

***Oreochromis niloticus*** (Linnaeus): unidentified cestode

***Oreochromis* sp.**: unidentified proteocephalid

***Satanoperca pappaterra*** (Heckel): unidentified cyclophyllidean, unidentified proteocephalid

***Tilapia rendalli*** (Boulenger): unidentified cestode

Family Coryphaenidae

***Coryphaena equiselis*** Linnaeus: *Tentacularia coryphaenae* (L)

***Coryphaena hippurus*** Linnaeus: *Floriceps saccatus* (L), *Hepatoxylon trichiuri* (L),

*Pterobothrium acanthotruncatum*, *Nybelinia* sp., *Tentacularia coryphaenae*, ‘*Scolex* spp.’, unidentified cestode

Family Eleginopsidae

***Eleginops maclovinus*** (Cuvier): *Bothriocephalus* sp., Grillotia (Grillotia) erinaceus (L), *Grillotia* sp. (L), unidentified bothriocephalidean, ‘*Scolex* spp.’ (L)

Family Eleotridae

***Dormitator maculatus*** (Bloch): unidentified cyclophyllidean (L)

Family Gempylidae

***Thyrsites atun*** (Euphrasen): *Molicola* sp. (L)

Family Gerreidae

***Diapterus rhombeus*** (Cuvier): *Nybelinia* sp. (L)

Family Gobiidae

***Gobioides broussonnetii*** Lacépède: *Pterobothrium crassicole* (L)

***Gobionellus oceanicus*** (Pallas): *Rhinebothrium* sp. (L)

Family Haemulidae

***Conodon nobilis*** (Linnaeus): *Callitetrarhynchus* sp. (L), *Pterobothrium* sp. (L)

***Haemulon aurolineatum*** Cuvier: *Callitetrarhynchus gracilis* (L), *Pterobothrium kingstoni* (L)

***Haemulon plumierii*** (Lacépède): *Heteronybelinia estigmena* (L), *Nybelinia lingualis* (L), *Pseudotobothrium dipsacum* (L)

***Haemulon steindachneri*** (Jordan & Gilbert): ‘*Scolex* spp.’ (L)

***Isacia conceptionis*** (Cuvier): *Hepatoxylon trichiuri* (L), *Nybelinia* sp. (L)

***Orthopristis ruber*** (Cuvier): unidentified trypanorhynch, ‘*Scolex* spp.’ (L)

***Pomadasys crocro*** (Cuvier): *Pterobothrium heteracanthum* (L)

Family Kyphosidae

***Girella laevifrons*** (Tschudi): unidentified bothriocephalidean

Family Labrisomidae

***Labrisomus philippii*** (Steindachner): *Lacistorhynchus tenuis* (L)

Family Lutjanidae

***Lutjanus analis*** (Cuvier): *Grillotia* sp. (L), unidentified trypanorhynch

***Lutjanus jocu*** (Bloch & Schneider): unidentified cestode

***Lutjanus synagris*** (Linnaeus): *Callitetrarhynchus gracilis* (L)

Family Mullidae

***Mullus argentinae*** Hubbs & Marini: *Heteronybelinia nipponica* (L), *Nybelinia* sp. (L)

***Pseudupeneus maculatus*** (Bloch): *Heteronybelinia overstreeti* (L), *Mixonybelinia edwinlintoni* (L), *Nybelinia africana* (L), *Nybelinia indica* (L), *Nybelinia lingualis* (L), *Pseudolacistorhynchus noodti* (L), *Pseudotobothrium dipsacum* (L)

Family Nototheniidae

***Dissostichus eleginoides*** Smitt: *Clestobothrium crassiceps* (?), Grillotia (Grillotia) erinaceus (L), *Hepatoxylon trichiuri* (L), unidentified ‘pseudophyllidean’, unidentified trypanorhynch, ‘*Scolex* spp.’ (L)

**Notothenia cf. angustata** Hutton: unidentified diphyllidean, unidentified trypanorhynch, ‘*Scolex* spp.’ (L)

***Patagonotothen brevicauda brevicauda*** (Lönnberg): Grillotia (Grillotia) patagonica (L)

***Patagonotothen ramsayi*** (Regan): Grillotia (Grillotia) patagonica (L)

Family Percichthyidae

***Percichthys trucha*** (Valenciennes): *Diphyllobothrium dendriticum* (L), *Diphyllobothrium latum* (L), unidentified bothriocephalidean, unidentified cyclophyllidean (L)

***Percichthys* sp.**: *Diphyllobothrium latum* (L)

Family Perciliidae

***Percilia gillissi*** Girard: *Diphyllobothrium dendriticum* (L)

Family Percophidae

***Percophis brasiliensis*** Quoy & Gaimard: *Callitetrarhynchus gracilis* (L), Grillotia (Christianella) carvajalregorum (L), *Grillotia* sp. (L), *Nybelinia* sp. (L), unidentified bothriocephalidean, unidentified ‘pseudophyllidean’, ‘*Scolex* spp.’ (L)

Family Pinguipedidae

***Pinguipes brasilianus*** Cuvier: *Anonchocephalus* sp., *Callitetrarhynchus gracilis* (L), *Grillotia* sp. (L), ‘*Scolex* spp.’ (L)

***Prolatilus jugularis*** (Valenciennes): ‘*Scolex* spp. (L)’, unidentified bothriocephalidean unidentified ‘pseudophyllidean’

***Pseudopercis numida*** Miranda Ribeiro: *Callitetrarhynchus gracilis* (L), Grillotia (Christianella) carvajalregorum (L), *Nybelinia* sp. (L), unidentified trypanorhynch, ‘*Scolex* spp.’ (L)

***Pseudopercis semifasciata*** (Cuvier): Grillotia (Christianella) carvajalregorum (L), *Grillotia* sp. (L), *Hepatoxylon trichiuri* (L), *Nybelinia* sp. (L), ‘*Scolex* spp.’ (L), unidentified cestode

Family Polyprionidae

***Polyprion oxygeneios*** (Schneider & Forster): *Tentacularia coryphaenae* (L)

Family Pomatomidae

***Pomatomus saltatrix*** (Linnaeus): *Callitetrarhynchus gracilis* (L), *Callitetrarhynchus speciosus* (L), Grillotia (Christianella) carvajalregorum (L), *Nybelinia* sp. (L), *Otobothrium cysticum* (L), *Pseudogrillotia* sp. (L), *Pterobothrium crassicole* (L), *Pterobothrium* sp. (L), unidentified trypanorhynch, ‘*Scolex* spp.’ (L)

Family Priacanthidae

***Priacanthus arenatus*** Cuvier: *Callitetrarhynchus speciosus* (L), ‘*Scolex* spp.’ (L)

Family Sciaenidae

***Cheilotrema fasciatum*** Tschudi: *Lacistorhynchus dollfusi* (L), *Lacistorhynchus tenuis* (L)

***Cilus gilberti*** (Abbott): *Adenocephalus pacificus* (L), *Diphyllobothrium* sp. (L), *Lacistorhynchus tenuis* (L), *Poecilancistrium caryophyllum* (L), *Nybelinia* sp. (L), ‘*Scolex* spp.’ (L)

***Ctenosciaena gracilicirrhus*** (Metzelaar): Grillotia (Christianella) carvajalregorum (L), *Heteronybelinia annakohnae* (L)

***Cynoscion acoupa*** (Lacépède): *Callitetrarhynchus gracilis* (L), *Poecilancistrium caryophyllum* (L), *Pterobothrium crassicole* (L), *Pterobothrium heteracanthum* (L), *Pterobothrium* sp. (L), unidentified trypanorhynch

***Cynoscion analis*** (Jenyns): *Adenocephalus pacificus* (L), *Diphyllobothrium* sp. (L), *Nybelinia* sp. (L)

***Cynoscion guatucupa*** (Cuvier): *Callitetrarhynchus gracilis* (L), *Callitetrarhynchus speciosus* (L), *Dasyrhynchus pacificus* (L), Grillotia (Christianella) carvajalregorum (L), Grillotia (Christianella) minuta (?) (L), *Heteronybelinia annakohnae* (L), *Nybelinia* sp. (L), *Pterobothrium heteracanthum* (L),‘*Scolex* spp.’ (L), unidentified cestode

***Cynoscion jamaicensis*** (Vaillant & Bocourt): *Dasyrhynchus pacificus* (L), Grillotia (Christianella) carvajalregorum (L), *Heteronybelinia annakohnae* (L), *Heteronybelinia estigmena* (L), unidentified trypanorhynch

***Cynoscion leiarchus*** (Cuvier): *Pterobothrium crassicole* (L), unidentified trypanorhynch

***Cynoscion striatus*** (Cuvier): Grillotia (Christianella) carvajalregorum (L), *Pterobothrium* sp. (L), unidentified proteocephalid, unidentified trypanorhynch

***Cynoscion* sp.**: *Nybelinia lingualis* (L), *Pterobothrium crassicole* (L), unidentified trypanorhynch

***Larimus breviceps*** Cuvier: *Callitetrarhynchus gracilis* (L)

***Macrodon ancylodon*** (Bloch & Schneider): *Callitetrarhynchus gracilis* (L), *Dasyrhynchus pacificus* (L), Grillotia (Christianella) carvajalregorum (L), *Nybelinia* sp. (L), *Poecilancistrium caryophyllum* (L), *Pterobothrium* sp. (L), unidentified trypanorhynch, unidentified cestode

***Menticirrhus americanus*** (Linnaeus): *Dasyrhynchus pacificus* (L), Grillotia (Christianella) carvajalregorum (L), *Heteronybelinia annakohnae* (L), *Heteronybelinia nipponica* (L), *Pterobothrium* sp. (L), ‘*Scolex* spp.’ (L)

***Menticirrhus littoralis*** (Holbrook): Grillotia (Christianella) carvajalregorum (L)

***Menticirrhus ophicephalus*** (Jenyns): *Adenocephalus pacificus* (L)

***Micropogonias altipinnis*** (Günther): *Poecilancistrium caryophyllum* (L)

***Micropogonias furnieri*** (Desmarest): *Callitetrarhynchus gracilis* (L), *Callitetrarhynchus speciosus* (L), *Dollfusiella* sp. (L), *Gilquinia* sp. (L), Grillotia (Christianella) carvajalregorum (L), *Nybelinia* sp. (L), *Poecilancistrium caryophyllum* (L), *Pterobothrium acanthotruncatum* (L), *Pterobothrium crassicole* (L), *Pterobothrium heteracanthum* (L), *Pterobothrium* sp. (L), unidentified trypanorhynch, ‘*Scolex* spp.’ (L), unidentified cestode

***Micropogonias undulatus*** (Linnaeus): *Pterobothrium crassicole* (L), *Pterobothrium heteracanthum* (L), unidentified trypanorhynch

***Micropogonias* sp.**: unidentified cestode

***Paralabrax humeralis*** (Valenciennes): *Adenocephalus pacificus* (L), *Grillotia* sp. (L), *Nybelinia* sp. (L), unidentified bothriocephalidean, unidentified trypanorhynch

***Paralonchurus brasiliensis*** (Steindachner): Grillotia (Christianella) carvajalregorum (L), *Nybelinia* sp. (L), ‘*Scolex* spp.’ (L)

***Paralonchurus peruanus*** (Steindachner): *Adenocephalus pacificus* (L), *Callitetrarhynchus gracilis* (L), *Diphyllobothrium* sp. (L), *Pterobothrium acanthotruncatum* (L)

***Plagioscion squamosissimus*** (Heckel): *Callitetrarhynchus gracilis* (L), *Pterobothrium crassicole* (L), *Pterobothrium heteracanthum* (L), *Poecilancistrium caryophyllum* (L), unidentified bothriocephalidean, unidentified cestode

***Pogonias cromis*** (Linnaeus): *Pterobothrium crassicole* (L), *Pterobothrium heteracanthum* (L)

***Sciaena callaensis*** Hildebrand: *Adenocephalus pacificus* (L), *Diphyllobothrium* sp. (L).

***Sciaena deliciosa*** (Tschudi): *Adenocephalus pacificus* (L), *Callitetrarhynchus gracilis* (L), *Dasyrhynchus pacificus* (L), *Diphyllobothrium* sp. (L), *Nybelinia* sp. (L)

***Umbrina canosai*** Berg: *Callitetrarhynchus gracilis* (L), Grillotia (Christianella) carvajalregorum (L), *Heteronybelinia nipponica* (L), *Nybelinia bisulcata* (?) (L), *Nybelinia* sp. (L), *Pterobothrium heteracanthum* (L), *Pterobothrium* sp. (L)

***Umbrina coroides*** Cuvier: unidentified cestode

**Sciaenidae gen. sp.**: unidentified trypanorhynch

Family Scombridae

***Auxis thazard*** (Lacépède): unidentified trypanorhynch

***Euthynnus alletteratus*** (Rafinesque): *Callitetrarhynchus gracilis* (L), unidentified trypanorhynch, ‘*Scolex* spp.’ (L)

***Katsuwonus pelamis*** (Linnaeus): *Tentacularia coryphaenae* (L), ‘*Scolex* spp.’ (L)

***Sarda chiliensis*** (Cuvier): *Adenocephalus pacificus* (L), *Acanthobothrium chilense*, *Nybelinia* sp. (L), *Rhodobothrium mesodesmatum* (L), *Sphyriocephalus tergestinus* (L), ‘*Scolex* spp.’ (L)

***Scomber colias*** Gmelin: *Rhinebothrium* sp. (L), unidentified trypanorhynch, unidentified cestode, ‘*Scolex* spp.’ (L)

***Scomber japonicus*** Houttuyn: *Diphyllobothrium* sp. (L), *Hepatoxylon trichiuri* (L), *Nybelinia* sp. (L), *Tentacularia coryphaenae* (L), unidentified trypanorhynch, ‘*Scolex* spp.’ (L)

***Scomberomorus brasiliensis*** Collette, Russo & Zavala-Camin: *Callitetrarhynchus gracilis* (L), *Nybelinia* sp. (L), *Otobothrium cysticum* (L), *Pseudolacistorhynchus noodti* (L), ‘*Scolex* spp.’ (L)

***Scomberomorus cavalla*** (Cuvier): *Callitetrarhynchus gracilis* (L), *Callitetrarhynchus speciosus* (L), *Pterobothrium crassicole* (L), *Tentacularia coryphaenae* (L)

***Scomberomorus sierra*** Jordan & Starks: *Adenocephalus pacificus* (L), *Pseudogrillotia peruviana* (L)

***Stellifer minor*** (Tschudi): ‘*Scolex* spp.’ (L)

Family Serranidae

***Acanthistius brasilianus*** (Cuvier): Grillotia (Christianella) carvajalregorum (L)

***Dules auriga*** Cuvier: Grillotia (Christianella) carvajalregorum (L), unidentified trypanorhynch, unidentified cestode

***Epinephelus morio*** (Valenciennes): unidentified trypanorhynch

***Epinephelus* sp.**: *Pterobothrium* sp. (L), unidentified trypanorhynch

***Hemilutjanus macrophthalmos*** (Tschudi): *Callitetrarhynchus gracilis* (L)

***Hyporthodus niveatus*** (Valenciennes): *Callitetrarhynchus gracilis* (L), *Pseudotobothrium dipsacum* (L), unidentified trypanorhynch

***Mycteroperca bonaci*** (Poey): *Pterobothrium* sp. (L), unidentified trypanorhynch

Family Sparidae

***Pagrus pagrus*** (Linnaeus): *Callitetrarhynchus gracilis* (L), *Pterobothrium* sp. (L), *Otobothrium cysticum* (L), unidentified trypanorhynch, ‘*Scolex* spp.’ (L)

**Sparidae gen. sp.**: *Pterobothrium macrourum* (L)

Family Sphyraenidae

***Sphyraena guachancho*** Cuvier: *Callitetrarhynchus gracilis* (L), *Heteronybelinia estigmena* (L), *Otobothrium cysticum* (L)

Family Trichiuridae

***Trichiurus lepturus*** Linnaeus: *Callitetrarhynchus gracilis* (L), *Pterobothrium interruptum* (L), unidentified trypanorhynch, ‘*Scolex* spp.’ (L)

Family Tripterygiidae

***Helcogrammoides chilensis*** (Cancino): unidentified ‘pseudophyllidean’, ‘*Scolex* spp.’ (L)

**Order Pleuronectiformes**

Family Achiridae

***Trinectes maculatus*** (Bloch & Schneider): unidentified cestode

Family Paralichthyidae

***Citharichthys spilopterus*** Günther: *Pterobothrium crassicole* (L), *Pterobothrium kingstoni* (L)

***Hippoglossina macrops*** Steindachner: *Neobothriocephalus* sp., *Nybelinia surmenicola* (L), *Nybelinia* sp. (L), ‘*Scolex* spp.’ (L)

***Paralichthys adspersus*** (Steindachner): *Adenocephalus pacificus* (L), *Lacistorhynchus dollfusi* (L), *Neobothriocephalus* sp., *Nybelinia surmenicola* (L), *Nybelinia* sp. (L), unidentified bothriocephalidean, ‘*Scolex* spp.’ (L)

***Paralichthys isosceles*** Jordan: *Callitetrarhynchus gracilis* (L), Grillotia (Christianella) carvajalregorum (L), *Diphyllobothrium* sp. (L), *Heteronybelinia nipponica* (L), *Nybelinia lingualis* (L), *Nybelinia* sp. (L), *Otobothrium* sp. (L), *Pterobothrium crassicole* (L), *Pterobothrium heteracanthum* (L), *Pterobothrium* sp. (L), unidentified trypanorhynch, ‘*Scolex* spp.’ (L), unidentified cestode

***Paralichthys microps*** (Günther): *Neobothriocephalus* sp., *Nybelinia* sp. (L), ‘*Scolex* spp.’ (L)

***Paralichthys orbignyanus*** (Valenciennes): *Grillotia* sp. (L), ‘*Scolex* spp.’ (L)

***Paralichthys patagonicus*** Jordan: *Anonchocephalus patagonicus*, *Callitetrarhynchus gracilis* (L), Grillotia (Christianella) carvajalregorum (L), *Heteronybelinia nipponica* (L), *Lacistorhynchus tenuis* (L), *Nybelinia erythraea* (L), *Nybelinia lingualis* (L), *Nybelinia* sp. (L), *Pterobothrium crassicole* (L), ‘*Scolex* spp.’ (L)

***Paralichthys* sp.**: *Pterobothrium* sp. (L)

***Xystreurys rasile*** (Jordan): *Anonchocephalus argentinensis*, Grillotia (Christianella) carvajalregorum (L), *Heteronybelinia nipponica* (L), *Nybelinia erythraea* (L), *Nybelinia lingualis* (L), *Nybelinia* sp. (L), unidentified trypanorhynch, ‘*Scolex* spp.’ (L), unidentified cestode

Family Pleuronectidae

***Oncopterus darwinii*** Steindachner: *Nybelinia lingualis* (L)

**Order Salmoniformes**

Family Salmonidae

***Oncorhynchus kisutch*** (Walbaum): *Diphyllobothrium dendriticum* (L), *Diphyllobothrium* sp. (L), ‘*Scolex* spp.’ (L)

***Oncorhynchus mykiss*** (Walbaum): *Diphyllobothrium dendriticum* (L), *Diphyllobothrium latum* (L), *Diphyllobothrium* sp. (L), unidentified bothriocephalidean, unidentified phyllobothriidean

***Oncorhynchus tshawytscha*** (Walbaum): *Hepatoxylon trichiuri* (L)

***Salmo salar*** Linnaeus: *Diphyllobothrium dendriticum* (L)

***Salmo trutta*** Linnaeus: *Diphyllobothrium dendriticum* (L), *Diphyllobothrium latum* (L), *Diphyllobothrium* sp. (L), ‘*Scolex* spp.’ (L)

***Salvelinus fontinalis*** (Mitchill): *Diphyllobothrium dendriticum* (L), *Diphyllobothrium latum* (L), *Diphyllobothrium* sp. (L), unidentified bothriocephalidean

**Order Scorpaeniformes**

Family Dactylopteridae

***Dactylopterus volitans*** (Linnaeus): *Nybelinia* sp. (L), ‘*Scolex* spp.’ (L)

Family Normanichthyidae

***Normanichthys crockeri*** Clark: ‘*Scolex* spp.’ (L)

Family Scorpaenidae

***Scorpaena* sp.**: *Pterobothrium crassicole* (L)

Family Sebastidae

***Helicolenus lengerichi*** Norman: *Bothriocephalus* sp., *Hepatoxylon* sp. (L), unidentified trypanorhynch

***Sebastes capensis*** (Gmelin): *Diphyllobothrium* sp. (L), *Hepatoxylon trichiuri* (L), unidentified diphyllidean, ‘*Scolex* spp.’ (L)

Family Triglidae

***Prionotus nudigula*** Ginsburg: Grillotia (Christianella) carvajalregorum (L), *Grillotia* sp. (L)

***Prionotus punctatus*** (Bloch): Grillotia (Christianella) carvajalregorum (L), *Nybelinia* sp. (L)

***Prionotus* sp.**: *Pterobothrium* sp. (L), unidentified phyllobothriidean

**Order Siluriformes**

Family Ariidae

***Ariopsis seemanni*** (Günther): *Adenocephalus pacificus* (L)

***Aspistor luniscutis*** (Valenciennes): *Pterobothrium crassicole* (L), ‘*Scolex* spp.’ (L)

***Bagre bagre*** (Linnaeus): unidentified trypanorhynch

***Bagre marinus*** (Mitchill): *Pterobothrium crassicole* (L)

***Galeichthys peruvianus*** Lütken: *Adenocephalus pacificus* (L)

***Genidens barbus*** (Lacépède): *Callitetrarhynchus gracilis* (L), *Callitetrarhynchus speciosus* (L), *Nomimoscolex arandasregoi*, *Pterobothrium crassicole* (L), unidentified trypanorhynch, ‘*Scolex* spp.’ (L)

***Genidens genidens*** (Cuvier): *Nomimoscolex arandasregoi*

***Genidens* sp.**: *Nomimoscolex arandasregoi*, unidentified trypanorhynch

Family Auchenipteridae

***Ageneiosus inermis*** (Linnaeus): *Ageneiella brevifilis*, *Ageneiella* sp., *Gibsoniela mandube*, *Gibsoniela meursaulti*, *Luciaella ivanovae*, unidentified cestode

***Ageneiosus militaris*** Valenciennes: *Ageneiella brevifilis*, *Gibsoniela meursaulti*

***Ageneiosus pardalis*** Lütken: *Corallotaenia* sp. (?), *Goezeella danbrooksi*

***Ageneiosus* sp.**: *Gibsoniela mandube*

***Auchenipterus nigripinnis*** (Boulenger): unidentified proteocephalid

***Auchenipterus osteomystax*** (Miranda Ribeiro): *Endorchis auchenipteri*

***Tocantinsia piresi*** (Miranda Ribeiro): *Frezella vaucheri*

***Trachelyopterus galeatus*** (Linnaeus): *Cangatiella arandasi*, *Nupelia tomasi*

***Trachelyopterus striatulus*** (Steindachner): *Endorchis* sp.

**Trachelyopterus cf. striatulus**: *Nupelia tomasi*

Family Callichthyidae

***Callichthys callichthys*** (Linnaeus): *Margaritaella gracilis*

***Corydoras atropersonatus*** Weitzman & Nijssen: unidentified proteocephalid

***Corydoras reticulatus*** Fraser-Brunner: unidentified proteocephalid

***Corydoras sychri*** Weitzman: unidentified proteocephalid

***Hoplosternum littorale*** (Hancock): *Valipora campylancristrota* (L)

Family Cetopsidae

***Cetopsis coecutiens*** (Lichtenstein): *Brooksiella praeputialis*, *Goezeella siluri*, unidentified cestode

***Cetopsis othonops*** (Eigenmann): *Brooksiella praeputialis*, *Goezeella siluri*

Family Diplomystimidae

***Diplomystes camposensis*** Arratia: *Diphyllobothrium latum* (L)

***Olivaichthys viedmensis*** (MacDonagh): *Nomimoscolex semenasae*

Family Doradidae

***Franciscodoras marmoratus*** (Lütken): *Proteocephalus renauldi*, *Proteocephalus* sp.

***Megalodoras uranoscopus*** (Eigenmann & Eigenmann): *Proteocephalus kuyukuyu*

***Oxydoras kneri*** Bleeker: *Proteocephalus hobergi*, unidentified proteocephalid

***Oxydoras niger*** (Valenciennes): *Proteocephalus hobergi*, *Proteocephalus kuyukuyu*

***Platydoras costatus*** (Linnaeus): *Proteocephalus renaudi* (?), *Proteocephalus soniae* (?), *Proteocephalus* sp. (?)

***Pterodoras granulosus*** (Valenciennes): *Monticellia belavistensis*, *Proteocephalus kuyukuyu*, *Proteocephalus* sp.

***Pterodoras* sp.**: *Proteocephalus kuyukuyu*

Family Heptapteridae

***Goeldiella eques*** (Müller & Troschel): *Nupelia* sp.

***Pimelodella gracilis*** (Valenciennes): unidentified proteocephalid, unidentified cestode

***Pimelodella lateristriga*** (Lichtenstein): unidentified cestode

***Rhamdia quelen*** (Quoy & Gaimard): *Lenhataenia megacephala*, *Proteocephalus bagri*, *Proteocephalus rhamdiae*, *Travassiella jandia*, unidentified proteocephalid

Family Loricariidae

**Hypostomus cf. ternetzi** (Boulenger): unidentified proteocephalid

***Loricariichthys platymetopon*** Isbrücker & Nijssen: unidentified proteocephalid

***Loricariichthys* sp.**: unidentified cestode

***Paraloricaria* sp.**: *Proteocephalus pilarensis*

Family Pimelodidae

***Brachyplatystoma capapretum*** Lundberg & Akama: *Amazotaenia yvettae*, *Endorchis piraeeba*

***Brachyplatystoma filamentosum*** (Lichtenstein): *Amphoteromorphus ninoi*, *Amphoteromorphus piraeeba*, *Nomimoscolex piraeeba*, *Nomimoscolex suspectus*, unidentified proteocephalid

**Brachyplatystoma cf. filamentosum**: *Amphoteromorphus ovalis*, *Endorchis piraeeba*, *Nomimoscolex suspectus*

***Brachyplatystoma rousseauxii*** (Castelnau): *Amphoteromorphus peniculus*, *Amphoteromorphus piriformis*, *Nomimoscolex dorad*, *Nomimoscolex piraeeba*, *Nomimoscolex suspectus*, *Nomimoscolex* sp., *Pterobothrium crassicole* (L)

***Brachyplatystoma vaillantii*** (Valenciennes): *Amazotaenia yvettae*, *Amphoteromorphus*

*ninoi*, *Chambriella* sp., *Goezeella siluri*, *Harriscolex piramutab*, *Nomimoscolex suspectus*, *Pterobothrium crassicole* (L)

***Brachyplatystoma* sp.**: *Amphoteromorphus ovalis*, ‘*Scolex* spp.’ (L), unidentified cestode

***Calophysus macropterus*** (Lichtenstein): *Monticellia amazonica*, *Rudolphiella piracatinga*, unidentified cestode

***Hemisorubim platyrhynchos*** (Valenciennes): *Chambriella paranaensis*, *Manaosia bracodemoca*, *Mariauxiella piscatorum*, *Spatulifer maringaensis*, unidentified cestode

***Iheringichthys labrosus*** (Lütken): unidentified proteocephalid

***Luciopimelodus pati*** (Valenciennes): *Monticellia ventrei*, *Nomimoscolex* sp., *Proteocephalus fossatus*, *Rudolphiella lobosa* (?), *Rudolphiella szidati*, *Rudolphiella* sp.

***Megalonema platanum*** (Günther): *Monticellia santafesina*, *Rudolphiella lobosa*, unidentified proteocephalid

***Megalonema platycephalum*** Eigenmann: *Monticellia santafesina*

***Phractocephalus hemioliopterus*** (Schneider): *Chambriella* sp., *Ephedrocephalus microcephalus*, *Proteocephalus hemioliopteri*, *Proteocephalus* sp., *Pseudocrepidobothrium eirasi*, *Pseudocrepidobothrium ludovici*, *Pseudocrepidobothrium* sp., *Scholzia emarginata*, *Zygobothrium megacephalum*, Monticelliinae gen. sp., unidentified proteocephalid

***Pimelodus albicans*** (Valenciennes): *Monticellia magna*, *Nomimoscolex microacetabula*

***Pimelodus altissimus*** Eigenmann & Pearson: *Endorchis* sp.

***Pimelodus argenteus*** Perugia: *Monticellia magna*

***Pimelodus blochii*** Valenciennes: *Nomimoscolex alovarius*, *Proteocephalus* sp.

**Pimelodus cf. blochii**: *Monticellia magna* (?)

***Pimelodus maculatus*** Lacépède: *Chambriella agostinhoi*, *Monticellia magna*, *Nomimoscolex microacetabula*, *Nomimoscolex* sp., *Proteocephalus pimelodi*, *Proteocephalus* sp., *Valipora* sp. (L), unidentified proteocephalid, unidentified cestode

**Pimelodus cf. maculatus**: *Endorchis* sp., *Monticellia magna*

***Pimelodus ornatus*** Kner: *Mariauxiella pimelodi*, *Nomimoscolex microacetabula*, *Nomimoscolex* sp., *Spasskyellina mandi*, *Spasskyellina* sp.

***Pimelodus ortmanni*** Haseman: unidentified cestode

***Pimelodus pohli*** Ribeiro & Lucena: unidentified proteocephalid

***Pimelodus* sp.**: *Mariauxiella pimelodi*, *Pterobothrium crassicole* (L), unidentified proteocephalid

***Pinirampus pirinampu*** (Spix & Agassiz): *Goezeella siluri*, *Monticellia ventrei*, *Monticellia* sp., *Nomimoscolex admonticellia*, *Proteocephalus vladimirae*, *Rudolphiella myoides*, *Rudolphiella piranabu*, *Rudolphiella* sp., unidentified proteocephalid, unidentified cestode

***Platynematichthys notatus*** (Jardine): *Brayela karuatayi*

***Pseudoplatystoma corruscans*** (Agassiz): *Choanoscolex abscisus*, *Harriscolex kaparari* (?), *Harriscolex nathaliae*, *Megathylacus travassosi*, *Megathylacus* sp., *Monticellia* sp., *Nomimoscolex pertierrae*, *Nomimoscolex sudobim* (?), *Nomimoscolex* sp., *Peltidocotyle rugosa*, *Spasskyellina spinulifera*, unidentified proteocephalid

***Pseudoplatystoma fasciatum*** (Linnaeus) (*sensu lato*): *Chambriella* sp., *Choanoscolex abscisus*, *Choanoscolex* sp., *Euzetiella tetraphylliformis*, *Harriscolex kaparari*, *Houssayela sudobim*, *Megathylacus travassosi*, *Megathylacus* sp., *Monticellia* sp., *Nomimoscolex lopesi*, *Nomimoscolex sudobim*, *Nomimoscolex* sp., *Peltidocotyle rugosa*, *Pseudocrepidobothrium chanaorum*, *Regoella brevis*, *Spasskyellina spinulifera*, *Spasskyellina* sp., *Spatulifer rugosa*, unidentified proteocephalid, unidentified cestode

***Pseudoplatystoma tigrinum*** (Valenciennes): *Choanoscolex* sp., *Harriscolex kaparari*, *Nomimoscolex sudobim*, *Spasskyellina spinulifera*, *Spatulifer surubim*, *Spatulifer* sp.

***Pseudoplatystoma* sp.**: *Proteocephalus platystomi*, unidentified cestode

***Sorubim lima*** (Bloch & Schneider): *Manaosia bracodemoca*, *Nupelia portoriquensis*, *Spasskyellina spinulifera*, *Spatulifer maringaensis*

***Sorubimichthys planiceps*** (Spix & Agassiz): *Chambriella* sp., *Choanoscolex* sp., *Lenhataenia megacephala*, *Nomimoscolex lenha*, *Peltidocotyle lenha*, *Spasskyellina lenha*

***Steindachneridion parahybae*** (Steindachner): unidentified cestode

***Zungaro jahu*** (Ihering): *Chambriella agostinhoi*, *Choanoscolex abscisus*, *Euzetiella tetraphylliformis*, *Jauella glandicephalus*, *Megathylacus jandia*, *Peltidocotyle lenha*, *Peltidocotyle rugosa*, *Peltidocotyle* sp., *Travassiella jandia*, unidentified cestode

***Zungaro zungaro*** (Humboldt): *Amphoteromorphus parkamoo*, *Chambriella agostinhoi*, *Euzetiella tetraphylliformis*, *Jauella glandicephalus*, *Megathylacus jandia*, *Peltidocotyle lenha*, *Proteocephalus sophiae*, *Travassiella jandia*

Family Pseudopimelodidae

***Pseudopimelodus mangurus*** (Valenciennes): *Peltidocotyle rugosa*

Family Trichomycteridae

***Trichomycterus punctulatus*** Valenciennes: *Hepatoxylon megacephalum* (L)

Unidentified siluriform fish

‘***Silurus dorgado***’: *Monticellia diesingii*

‘***Silurus megacephalus***’: *Monticellia macrocotylea*

**Siluriform fish**: *Pterobothrium* sp. (L)

**Order Tetraodontiformes**

Family Balistidae

***Balistes capriscus*** Gmelin: *Callitetrarhynchus gracilis* (L), *Callitetrarhynchus speciosus* (L), *Callitetrarhynchus* sp. (L), *Nybelinia* sp. (L), unidentified ‘pseudophyllidean’, ‘*Scolex* spp.’ (L)

***Balistes vetula*** Linnaeus: *Callitetrarhynchus gracilis* (L), *Callitetrarhynchus speciosus* (L), *Callitetrarhynchus* sp. (L), *Otobothrium* sp. (L), unidentified trypanorhynch, ‘*Scolex* spp.’ (L)

Family Molidae

***Masturus lanceolatus*** Liénard: unidentified trypanorhynch

***Mola mola*** (Linnaeus): *Anchistrocephalus microcephalus*, *Molicola* sp. (L), unidentified trypanorhynch

***Mola ramsayi*** (Giglioli): *Anchistrocephalus microcephalus*, *Molicola horridus* (L), *Nybelinia* sp. (L)

Family Monacanthidae

***Aluterus monoceros*** (Linnaeus): *Callitetrarhynchus speciosus* (L), *Floriceps saccatus* (L)

***Stephanolepis hispidus*** (Linnaeus): *Callitetrarhynchus speciosus* (L)

Family Tetraodontidae

***Lagocephalus laevigatus*** (Linnaeus): unidentified cestode

***Sphoeroides testudineus*** (Linnaeus): unidentified cestode

**Class Chondrichthyes**

**Subclass Elasmobranchii**

**Order Carcharhiniformes**

Family Carcharhinidae

***Carcharhinus brachyurus*** (Günther): *Cathetocephalus australis*, *Dasyrhynchus pacificus*

***Carcharhinus leucas*** (Müller & Henle): *Cathetocephalus thatcheri*, *Poecilancistrium caryophyllum*

***Carcharhinus limbatus*** (Müller & Henle): *Dasyrhynchus pacificus*, unidentified trypanorhynch

***Carcharhinus longimanus*** (Poey): *Anthobothrium laciniatum*, *Disculiceps galapagoensis*, *Paraorygmatobothrium filiforme*, *Tentacularia coryphaenae*

***Carcharhinus obscurus*** (Lesueur): *Tentacularia coryphaenae*

***Carcharhinus porosus*** (Ranzani): *Disculiceps pileatus*

***Carcharhinus signatus*** (Poey): Grillotia (Christianella) carvajalregorum, *Heteronybelinia nipponica* (L), *Heteronybelinia yamagutii* (L)

***Prionace glauca*** (Linnaeus): *Callitetrarhynchus gracilis* (L), *Floriceps saccatus*, *Hepatoxylon trichiuri* (L), *Molicola horridus*, *Paraorygmatobothrium angustum*, *Paraorygmatobothrium prionacis*, *Platybothrium auriculatum*, *Prosobothrium armigerum*, *Tentacularia coryphaenae*

***Rhizoprionodon lalandii*** (Müller & Henle): *Poecilancistrium caryophyllum*

***Rhizoprionodon terraenovae*** (Richardson): *Nybelinia fayapaulazariahi*, unidentified trypanorhynch

Family Scyliorhinidae

***Scyliorhinus besnardi*** Springer & Sadowsky: *Ahamulina catarina*

***Scyliorhinus haeckelii*** (Miranda Ribeiro): *Dasyrhynchus pacificus*

Family Sphyrnidae

***Sphyrna lewini*** (Griffith & Smith): *Heteronybelinia nipponica*

***Sphyrna zygaena*** (Linnaeus): *Callitetrarhynchus speciosus* (L), *Heteronybelinia nipponica* (L), *Platybothrium parvum*, *Platybothrium* sp., *Thysanocephalum thysanocephalum*

***Sphyrna* sp.**: *Dasyrhynchus pacificus*

Family Triakidae

***Galeorhinus galeus*** (Linnaeus): *Anthobothrium galeorhini*

***Mustelus canis*** (Mitchill): *Callitetrarhynchus gracilis*, *Dollfusiella vooremi*, *Nybelinia lingualis* (L)

***Mustelus fasciatus*** (Garman): *Orygmatobothrium juani*

***Mustelus mento*** Cope: *Calliobothrium verticillatum*, *Calliobothrium* sp., *Dollfusiella musteli*, *Orygmatobothrium musteli*, *Orygmatobothrium* sp., *Paraorygmatobothrium triacis*, *Phyllobothrium lactuca*, *Phyllobothrium* sp., *Scyphophyllidium uruguayense*

***Mustelus schmitti*** Springer: *Calliobothrium australis*, *Coronocestus notoguidoi*, *Dollfusiella vooremi*, *Gyrocotyle maxima* (?), *Nybelinia lingualis*, *Orygmatobothrium schmittii*, *Symcallio barbarae*, *Symcallio lunae*

***Mustelus whitneyi*** Chirichigno: *Orygmatobothrium musteli*, *Paraorygmatobothrium triacis*, *Symcallio lintoni*

***Triakis maculata*** Kner & Steindachner: *Lacistorhynchus tenuis*

**Order Hexanchiformes**

Family Hexanchidae

***Heptranchias perlo*** (Bonnaterre): Grillotia (Christianella) carvajalregorum (L)

***Hexanchus griseus*** (Bonnaterre): *Crossobothrium dohrni*, *Crossobothrium laciniatum*, *Grillotia heptanchi*, *Phyllobothrium sinuosiceps*

***Notorynchus cepedianus*** (Péron): *Crossobothrium antonioi*, *Crossobothrium pequeae*, *Heteronybelinia perideraeus*

**Order Lamniformes**

Family Alopiidae

***Alopias vulpinus*** (Bonnaterre): *Paraorygmatobothrium angustum*

Family Cetorhinidae

***Cetorhinus maximus*** (Gunnerus): *Reesium paciferum*

Family Lamnidae

***Carcharodon carcharias*** (Linnaeus): *Tentacularia coryphaenae*, unidentified trypanorhynch

***Isurus oxyrinchus*** Rafinesque: *Gymnorhynchus isuri*, *Molicola horridus*, *Nybelinia lingualis*

**Order Myliobatiformes**

Family Dasyatidae

***Dasyatis americana*** Hildebrand & Schroeder: *Acanthobothrium americanum*, *Anthocephalum gracile*, *Anthocephalum kingae*, *Lecanicephalum peltatum*, *Polypocephalus medusia*, *Rhinebothrium corymbum*, *Rhinebothrium margaritense*, *Rhodobothrium pulvinatum*, *Scalithrium magniphallum*

***Dasyatis dipterura*** (Jordan & Gilbert): *Acanthobothroides peruensis*, *Acanthobothroides thorsoni*

***Dasyatis guttata*** (Bloch & Schneider): *Acanthobothrium tasajerasi*, *Acanthobothrium urotrygoni*, *Acanthobothroides thorsoni*, *Rhinebothrium margaritense*, *Rhodobothrium pulvinatum*, *Scalithrium magniphallum*

***Dasyatis longa*** (Garman): *Acanthobothrium campbelli*, *Acanthobothrium costarricense*, *Acanthobothrium obuncum*

***Himantura schmardae*** (Werner): *Acanthobothrium himanturi*, *Acanthobothrium tasajerasi*, *Acanthobothroides thorsoni*, *Anindobothrium anacolum*, *Parachristianella monomegacantha*, *Rhinebothrium tetralobatum*, *Scalithrium magniphallum*

Family Gymnuridae

***Gymnura afuerae*** (Hildebrand): *Acanthobothrium atahualpai*

***Gymnura micrura*** (Bloch & Schneider): *Acanthobothrium fogeli*

***Gymnura* sp.**: *Pterobothrium* sp.

Family Myliobatidae

***Aetobatus narinari*** (Euphrasen): *Acanthobothrium colombianum*, *Acanthobothrium monksi*, *A. tortumDisculiceps* sp. (?)

***Myliobatis chilensis*** Philippi: *Acanthobothrium batailloni*, *Acanthobothrium coquimbense*, *Acanthobothrium holorhini*, *Acanthobothrium* sp., *Caulobothrium myliobatidis*, *Phyllobothrium auricula*, *Rhodobothrium mesodesmatum*

***Myliobatis goodei*** Garman: *Aberrapex arrhynchum*, *Acanthobothrium* sp., *Caulobothrium ostrowskiae*, *Caulobothrium uruguayense*, *Halysioncum megacanthum*, *Mecistobothrium oblongum*, *Parachristianella damiani*, *Phyllobothrium myliobatidis*, *Phyllobothrium* sp.

***Myliobatis peruvianus*** Garman: *Acanthobothrium brevissime*, *Acanthobothrium gonzalesmugaburoi*, *Phyllobothrium auricula*, *Rhodobothrium mesodesmatum*

***Rhinoptera bonasus*** (Mitchill): *Dioecotaenia campbelli*, *Rhinoptericola megacantha*, *Rhodobothrium paucitesticulare*, *Tylocephalum brooksi*, *Tylocephalum* sp.

***Rhinoptera brasiliensis*** Müller: *Rhinoptericola megacantha*

***Rhinoptera steindachneri*** Evermann & Jenkins: *Serendip deborahae*

Family Potamotrygonidae

***Paratrygon aiereba*** (Müller & Henle): *Acanthobothrium terezae*, *Nandocestus guariticus*, *Potamotrygonocestus fitzgeraldae*, *Potamotrygonocestus travassosi*, *Potamotrygonocestus* sp., *Rhinebothrium brooksi*, *Rhinebothrium copianullum*, *Rhinebothrium* sp., *Rhinebothroides scorzai*, *Rhinebothroides* sp.

***Plesiotrygon iwamae*** Rosa, Castello & Thorson: *Potamotrygonocestus chaoi*, *Potamotrygonocestus marajoara*

***Potamotrygon brachyura*** (Günther): *Rhinebothrium paratrygoni*

***Potamotrygon constellata*** (Vaillant): *Acanthobothrium amazonense*, *Potamotrygonocestus amazonensis*, *Potamotrygonocestus travassosi*, *Rhinebothroides freitasi*

***Potamotrygon falkneri*** Castex & Maciel: *Acanthobothrium regoi*, *Paroncomegas araya*, *Potamotrygonocestus amazonensis*, *Potamotrygonocestus travassosi*, *Rhinebothrium paratrygoni*

**Potamotrygon cf. falkneri**: *Acanthobothrium peruviense*, *Nandocestus guariticus*, *Paroncomegas araya*, *Potamotrygonocestus fitzgeraldae*, *Potamotrygonocestus* sp., *Rhinebothroides freitasi*, *Rhinebothroides* sp.

***Potamotrygon henlei*** (Castelnau): *Potamotrygonocestus* sp., *Rhinebothrium copianullum*, *Rhinebothroides freitasi*, *Rhinebothroides glandularis*

***Potamotrygon histrix*** (Müller & Henle): *Rhinebothrium paratrygoni*

***Potamotrygon leopoldi*** Castex & Castello: *Potamotrygonocestus fitzgeraldae*, *Rhinebothrium copianullum*, *Rhinebothroides freitasi*

***Potamotrygon magdalenae*** (Duméril): *Acanthobothrium quinonese*, *Potamotrygonocestus magdalenensis*, *Rhinebothroides moralarai*

***Potamotrygon motoro*** (Müller & Henle): *Acanthobothrium peruviense*, *Acanthobothrium ramiroi*, *Acanthobothrium regoi*, *Acanthobothrium terezae*, *Paroncomegas araya*, *Potamotrygonocestus amazonensis*, *Potamotrygonocestus fitzgeraldae*, *Potamotrygonocestus travassosi*, *Potamotrygonocestus* sp., *Rhinebothrium copianullum*, *Rhinebothrium corbatai*, *Rhinebothrium mistyae*, *Rhinebothrium paratrygoni*, *Rhinebothroides campbelli*, *Rhinebothroides freitasi*, *Rhinebothroides glandularis*, *Rhinebothroides mclennanae*, *Rhinebothroides scorzai*, unidentified cestode

***Potamotrygon orbignyi*** (Castelnau): *Acanthobothrium regoi*, *Anindobothrium lisae*, *Paroncomegas araya*, *Paroncomegas* sp., *Potamotrygonocestus amazonensis*, *Potamotrygonocestus fitzgeraldae*, *Potamotrygonocestus maurae*, *Potamotrygonocestus travassosi*, *Rhinebothrium brooksi*, *Rhinebothrium copianullum*, *Rhinebothrium fulbrighti*, *Rhinebothrium jaimei*, *Rhinebothrium paratrygoni*, *Rhinebothroides freitasi*, *Rhinebothroides glandularis*, *Rhinebothroides scorzai*, unidentified cestode

***Potamotrygon schroederi*** Fernández-Yépez: *Potamotrygonocestus* sp., *Rhinebothrium copianullum*, *Rhinebothroides freitasi*

***Potamotrygon scobina*** Garman: *Potamotrygonocestus amazonensis*, *Rhinebothrium jaimei*, *Rhinebothroides freitasi*, *Rhinebothroides glandularis*

***Potamotrygon signata*** Garman: *Rhinebothroides glandularis*

***Potamotrygon tatianae*** Silva & Carvalho: *Rhinebothrium copianullum*, *Rhinebothroides* sp.

***Potamotrygon yepezi*** Castex & Castello: *Acanthobothrium quinonese*, *Potamotrygonocestus amazonensis*, *Rhinebothroides freitasi*

***Potamotrygon* sp.**: *Paroncomegas araya*, *Potamotrygonocestus amazonensis*, *Rhinebothrium copianullum*, *Rhinebothrium fulbrighti*, *Rhinebothrium paratrygoni*, *Rhinebothrium glandularis*, *Rhinebothrium moralarai*, *Rhinebothrium scorzai*

Family Urotrygonidae

***Urobatis jamaicensis*** (Cuvier): *Acanthobothrium cartaginense*, *Anthocephalum kingae*, *Scalithrium magniphallum*

***Urobatis tumbesensis*** (Chirichigno & McEachran): *Acanthobothrium minusculum*, *Anthocephalum hobergi*

***Urotrygon venezuelae*** Schultz: *Acanthobothrium urotrygoni*, *Scalithrium magniphallum*

**Order Pristiformes**

Family Pristidae

***Pristis pristis*** (Linnaeus): *Anthobothrium pristis*, *Pterobothrium fragile*

**Order Rajiformes**

Family Arhynchobatidae

***Atlantoraja castelnaui*** (Miranda Ribeiro): *Acanthobothrium marplatense*, *Dollfusiella acuta*, *Notomegarhynchus navonae*

***Atlantoraja platana*** (Günther): *Dollfusiella acuta*

***Bathyraja brachyurops*** (Fowler): *Guidus argentinense*

***Bathyraja magellanica*** (Philippi): *Grillotia* sp.

***Psammobatis bergi*** Marini: *Dollfusiella taminii*

***Psammobatis rudis*** Günther: Grillotia (Grillotia) patagonica

***Psammobatis scobina*** (Philippi): *Acanthobothrium psammobati*, *Rhinebothrium scobinae*

***Sympterygia acuta*** Garman: *Dollfusiella acuta*

***Sympterygia bonapartii*** Müller & Henle: *Dollfusiella acuta*, *Dollfusiella vooremi* (?), Grillotia (Grillotia) erinaceus (?), *Heteronybelinia mattisi*, *Nybelinia lingualis*, *Nybelinia* sp., *Phyllobothrium* sp., *Rhinebothrium chilensis*

***Sympterygia brevicaudata*** (Cope): *Acanthobothrium lusarmientoi*, *Acanthobothrium psammobati*, *Acanthobothrium* sp.

***Sympterygia lima*** (Poeppig): *Halysioncum euzeti*, *Rhinebothrium chilensis*, *Rhinebothrium leiblei*

Family Rajidae

***Dipturus flavirostris*** (Philippi): *Echeneibothrium megalosoma*, *Echeneibothrium multiloculatum*, *Echeneibothrium williamsi*, Grillotia (Grillotia) dollfusi, *Phyllobothrium* sp.

***Dipturus trachyderma*** (Krefft & Stehmann): *Mixonybelinia beveridgei* (L), *Paragrillotia* sp. (?), *Phyllobothrium lactuca* (?)

***Zearaja chilensis*** (Guichenot): *Acanthobothrium annapinkiense*, *Echeneibothrium megalosoma*, *Echeneibothrium multiloculatum*, *Echeneibothrium williamsi*, Grillotia (Grillotia) dollfusi

Family Rhinobatidae

***Rhinobatos percellens*** (Walbaum): unidentified trypanorhynch

***Rhinobatos planiceps*** Garman: *Acanthobothrium olseni*, *Acanthobothrium robustum*, *Parachristianella monomegacantha*, *Prochristianella heteracantha*, *Rhinebothrium rhinobati*

***Zapteryx brevirostris*** (Müller & Henle): *Acanthobothrium zapterycum*, *Acanthobothrium* sp., *Halysioncum pigmentatum*, *Phyllobothrium* sp. (L)

**Order Squaliformes**

Family Etmopteridae

***Etmopterus granulosus*** (Günther): *Gilquinia squali*

Family Somniosidae

***Somniosus pacificus*** Bigelow & Schroeder: *Hepatoxylon trichiuri* (L)

Family Squalidae

***Squalus* sp.**: Grillotia (Christianella) carvajalregorum

**Order Squatiniformes**

Family Squatinidae

***Squatina armata*** (Philippi): *Grillotia* sp.

***Squatina guggenheim*** Marini: Grillotia (Christianella) carvajalregorum, *Paraberrapex atlanticus*

**Order Torpediniformes**

Family Narcinidae

***Discopyge tschudii*** Heckel: *Phyllobothrium discopygi*

***Narcine brasiliensis*** (Olfers): *Acanthobothrium electricolum*, *Acanthobothrium lintoni*

**Unidentified ray order**

**Unidentified ray**: *Acanthobothrium dasybati* (?), *Pterobothrium* sp.

**Subclass Holocephali**

**Order Chimaeriformes**

Family Callorhinchidae

***Callorhinchus callorynchus*** (Linnaeus): *Gyrocotyle maxima*, *Gyrocotyle rugosa*

## Results and discussion

The database compiled from the available literature on fish cestodes in South America comprises records of 297 species recognized as valid as well as unidentified ones included in 120 genera and 32 families, associated with 401 cartilaginous and bony fish hosts (Tables 1, 2). Among the recognized 19 orders of tapeworms, 13 have been found in marine and freshwater systems in South America (excluding the doubtful reports of the Caryophyllidea). The recently erected order Onchoproteocephalidea, which accommodates several taxa previously placed in the tetraphyllidean family Onchobothriidae and the entire former order Proteocephalidea, is the most diverse group, being represented by 148 species in 43 genera.

**Table 1.** Survey of fish cestodes from South America according to their high taxonomic level classification.

Order	Family	No. of genera	No. of species	Identified to generic level	No. of sequences*
Amphilinidea	Amphilinidae	2	2	0	4
Bothriocephalidea	Bothriocephalidae	4	5	2	12
	Echinophallidae	2	1	2	4
	Triaenophoridae	4	6	1	1
Cathetocephalidea	Cathetocephalidae	1	2	0	0
	Disculicepitidae	1	2	1	0
Cyclophyllidea	Gryporhynchidae	3	2	1	0
Diphyllidea	Echinobothriidae	3	5	0	12
Diphyllobothriidea	Diphyllobothriidae	2	3	1	26
Gyrocotylidea	Gyrocotylidae	1	2	0	0
Lecanicephalidea	Aberrapecidae	1	1	0	0
	Cephalobothriidae	1	1	1	0
	Lecanicephalidae	1	1	0	0
	Paraberrapecidae	1	1	0	0
	Polypocephalidae	1	1	0	0
Onchoproteocephalidea	Onchobothriidae	4	45	3	3
	Prosobothriidae	1	1	0	0
	Proteocephalidae	38	102	15	164
Phyllobothriidea	Phyllobothriidae	7	15	2	7
Rhinebothriidea	Anthocephaliidae	1	3	0	6
	Echeneibothriidae	2	4	0	0
	Rhinebothriidae	4	24	2	57
‘Tetraphyllidea’	*incertae sedis*	7	13	1	3
Trypanorhyncha	Eutetrarhynchidae	5	9	2	2
	Gilquiniidae	1	1	1	0
	Gymnorhynchidae	2	2	1	0
	Lacistorhynchidae	9	16	5	0
	Otobothriidae	2	1	1	0
	Pseudotobothriidae	1	1	0	0
	Pterobothriidae	1	4	1	0
	Rhinoptericolidae	1	1	0	0
	Sphyriocephalidae	2	3	1	0
	Tentaculariidae	4	17	3	0
	**Total**	**120**	**297**	**61**	**301**

*Only sequences of cestodes collected in South America were considered.

**Table 2.** Survey of fish hosts that harbour cestodes in South America.

Class	Subclass	Order	No. of genera	No. of species	No. of cestodes reported*
**ACTINOPTERYGII**	Neopterygii	Anguilliformes	2	2	2
		Atheriniformes	2	7	4
		Aulopiformes	1	1	0
		Batrachoidiformes	2	2	4
		Beloniformes	3	3	0
		Characiformes	18	27	8
		Clupeiformes	8	9	2
		Cypriniformes	3	3	1
		Cyprinodontiformes	2	4	2
		Gadiformes	9	14	14
		Gobiesociformes	2	2	0
		Gymnotiformes	2	3	3
		Lampriformes	1	1	1
		Lophiiformes	1	1	2
		Mugiliformes	1	2	1
		Notacanthiformes	1	1	1
		Ophidiiformes	2	5	11
		Osmeriformes	2	4	4
		Osteoglossiformes	1	1	2
		Perciformes	87	125	46
		Pleuronectiformes	6	9	14
		Salmoniformes	3	6	3
		Scorpaeniformes	6	6	3
		Siluriformes	45	68	95
		Tetraodontiformes	7	9	5
**CHONDRICHTHYES**	Elasmobranchii	Carcharhiniformes**	8	21	44
		Hexanchiformes**	3	3	8
		Lamniformes**	4	4	6
		Myliobatiformes***	11	33	86
		Pristiformes***	1	1	2
		Rajiformes***	8	17	30
		Squaliformes**	3	2	3
		Squatiniformes**	1	2	2
		Torpediniformes***	2	2	3
	Holocephali	Chimaeriformes	1	1	2
		**TOTAL**	**259**	**401**	**414**

*Cestodes with no specific identification were not counted; **Selachii (sharks); ***Batoidea (rays)

The tapeworm with the widest spectrum of definitive hosts is *Rhinebothroides freitasi* (Rhinebothriidea) that parasitizes nine species of stingrays of the genus *Potamotrygon*, even though it exhibits only a mesostenoxenous specificity, i.e. occurrence limited to a single host genus. Conversely, members of five orders, namely Amphilinidea, Cathetocephalidea, Diphyllidea, Lecanicephalidea, ‘Tetraphyllidea’ and most likely Gyrocotylidea (see the checklist records for details), showed only a single fish host (oioxenous specificity). It is also worth noting the usually broad spectrum of intermediate teleost hosts for metacestodes, mainly diphyllobothriideans, ‘tetraphyllideans’ and trypanorhynchs, which is reflected in the higher number of actinopterygian (315) than chondrichthyean (86) hosts. However, the stingray *Potamotrygon motoro* harbours the highest number of cestodes (17) belonging to the species-rich genera *Acanthobothrium* Blanchard, 1848, *Potamotrygonocestus* Brooks & Thorson, 1976, *Rhinebothrium* Linton, 1890 and *Rhinebothroides* Mayes, Brooks & Thorson, 1981, in addition to *Paroncomegas araya*.

**Figure 1.**
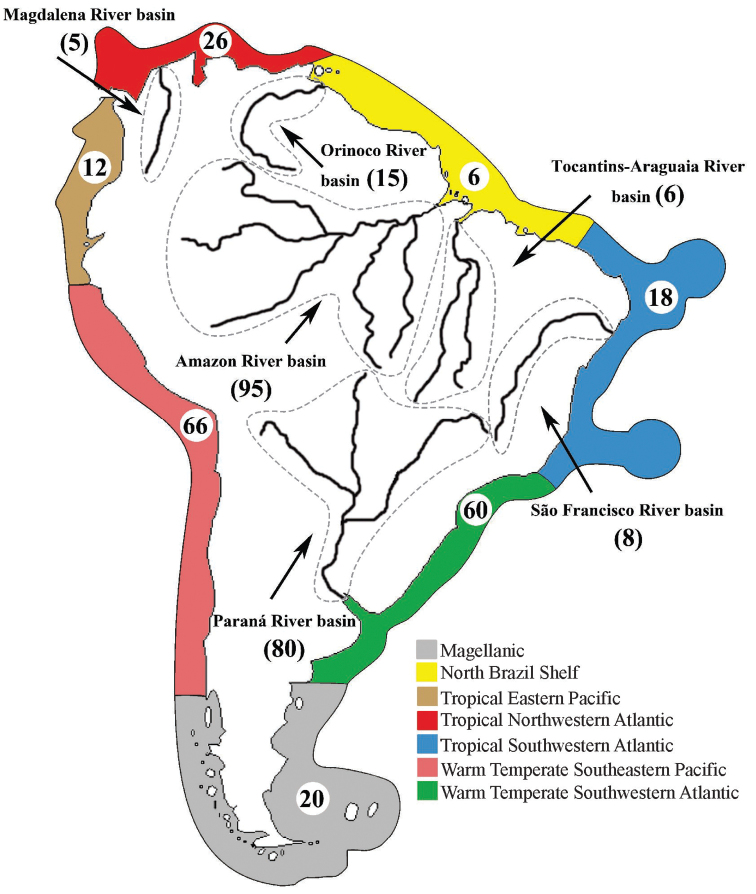
The geographical distribution of tapeworms in South America associated with their fish hosts from the major marine ecoregions of Spalding et al. (2007) and river basins in the continent. Each species may occur in more than one basin or ecoregion.

A total of 208 species of tapeworms are found across seven major ecoregions of South American coast (one additional species is found in Galapagos), being the highest species richness reported from WTSP (66) and WTSA (60), whereas 209 species are found throughout six major river basins of South America (Fig. 1). The major number of species comes from the Amazon and Paraná River basins, with 95 and 80 species, respectively. At least four species were reported from particular lakes, mostly parasitizing osmeriforms and salmoniforms in Argentina and Chile.

**Figure 2.**
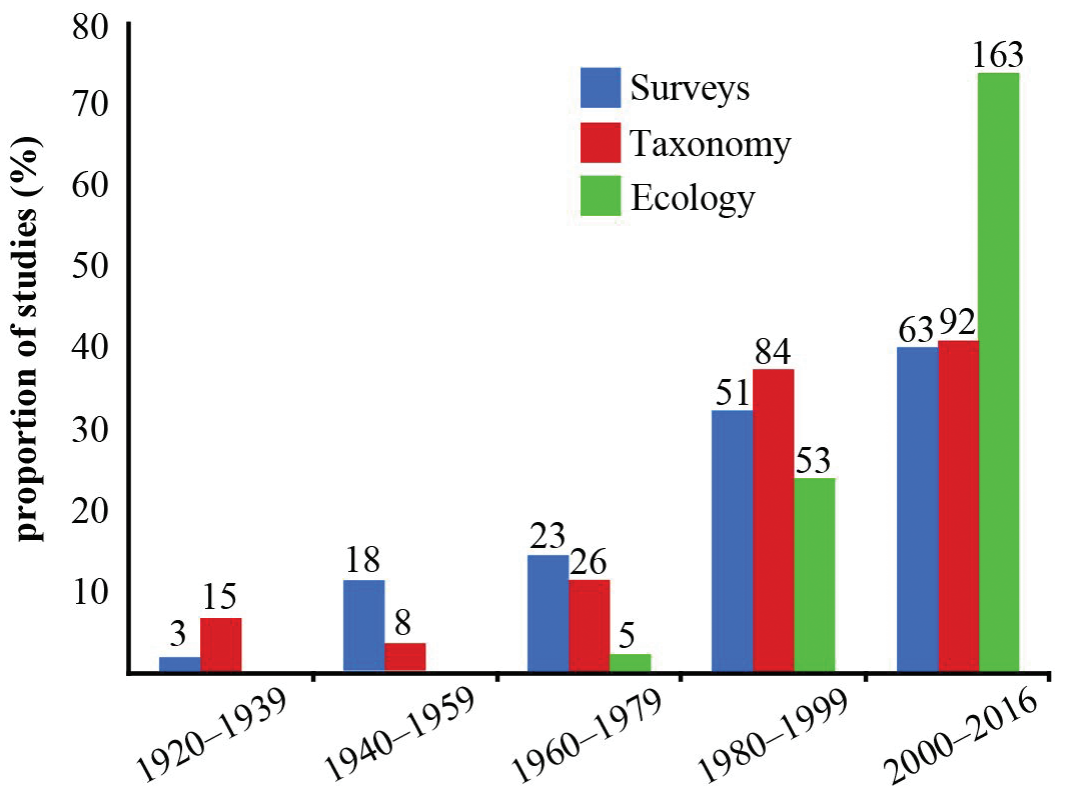
Proportion of articles on the fish cestodes from South America published per intervals of years sorted by categories. The numerals above individual bars indicate the absolute number of articles.

The number of taxonomic studies has been steadily growing since 1940, but only 16 papers were based on an integrated taxonomy approach, using molecular data as an important tool. The number of general parasitological surveys has also increased since the beginning of the last century, whereas ecological studies have launched the first publications only in the mid-sixties, with a peak in the last sixteen years, noticeably higher than the previous period (Fig. 2).

### Taxonomic resolution

Among the genera of fish cestodes reported from South America, one half was either identified only at generic level or they were specifically identified in some reports and at generic level in others. The numerous papers published in the last 30 years, mostly those ecological ones (see Fig. 2) focused on marine teleost hosts as models, include a high number of records of unidentified larvae. Most of them corresponded to the ‘Tetraphyllidea’ named as ‘*Scolex* spp.’, even though these individuals can belong to other orders that were previously included within this catch-all group (Chambers et al. 2000; Jensen and Bullard 2010). Poulin and Leung (2010) stated that the presence of larval stages in a community is inversely proportional to the taxonomic resolution achieved and those parasites in fish hosts exhibit lower taxonomic resolution than endohelminths parasitizing birds and mammals.

The accurate identification of larval stages of cestodes is usually challenging, because they lack key morphological traits that are present in their adult forms, and studies dealing with their genetic characterization are rare in South America (Rozas et al. 2012). An even more important concern is the high number of records of unidentified diphyllobothrid plerocercoids in teleosts (see the Parasite-Host list), because these metacestodes can infect humans who consume raw or undercooked fish and may cause a disease known as diphyllobothriosis (Scholz et al. 2009; Kuchta et al. 2015). Larval trypanorhynchs are the only exception, because they may be precisely identified based on their tentacular armature (Palm 2004; Caira and Jensen 2014). For instance, all three valid species of *Pterobothrium* Diesing, 1850 originally described from South America have teleost fishes as type hosts.

One of the main obstacles that hampers our understanding of the diversity of fish cestodes in South America is the deficient knowledge of their life cycles and failure to match the morphologically amorphous or divergent larval forms to their adult stages; to date, no life cycle studies have been undertaken in this continent. Jensen and Bullard (2010) performed the most comprehensive study combining molecular and morphological approaches to elucidate life cycles of marine cestodes from four metazoan phyla in the Gulf of Mexico. They found as many as eight larval types which could be associated with their adult forms and provided a useful morphological key for the 15 recognized types, including larvae of the currently recognized Onchoproteocephalidea, Phyllobothriidea, Rhinebothriidea and ‘Tetraphyllidea’.

Unlike the poor taxonomic resolution of marine larvae from teleosts, adult forms, typically those infecting freshwater catfishes (Siluriformes) and potamotrygonid stingrays (Potamotrygonidae), have been fairly well-documented (Reyda and Marques 2011; de Chambrier et al. 2015b). Their characterisation using modern descriptive tools, e.g. scanning electron micrographs and molecular data, associated with the traditional morphological approach, deeply contributed to the improvement of their taxonomic resolution and to elucidating the high cestode diversity associated with these groups of hosts.

### Elasmobranch and teleost fish hosts

Miloslavich et al. (2011) estimated the fish diversity in five subregions along the South American coast and suggested the occurrence of more than 5000 species in these marine systems. Reis (2013) estimated a value slightly higher for fishes from freshwater drainage systems in South America, *c.* 5400 species. Considering that predictions for estimating the global species richness of parasites suggest that they exceed twice the number of their hosts (Dobson 2008) and that only 4% of the potential fish hosts have been scrutinized for cestodes in South America, it is straightforward to conclude that our knowledge of the diversity of these parasites is far from adequate. Similar results were also found for trematodes infecting freshwater fishes in the same continent (Choudhury et al. 2016) and it may be valid also for others groups of helminths.

Contrasting the generally poorly-known diversity of fish cestodes in South America, some groups of hosts have been extensively studied compared to others. Among the elasmobranch hosts, the stingrays (Myliobatiformes) have been steadily examined for tapeworms, exhibiting the highest proportion of records (39%), which were mainly reported from marine and freshwater systems (e.g. Brooks et al. 1981a, b; Reyda and Marques 2011). Regarding teleosts, members of the order Perciformes are the most representative hosts, representing *c.* 40% of all records among this group. The majority of these studies have been conducted by ecological research teams interested in unravelling the structure of fish parasite communities and, more recently, their use as biological tags for stock discrimination (e.g. Luque et al. 2010; Timi et al. 2010a).

According to Luque and Poulin (2007), the study effort and local priorities of research teams play an important role on the uneven knowledge of parasite species richness in Neotropical fishes. Since cestodes are ubiquitously distributed in fishes from South America, it is likely that the higher the number of elasmobranchs and teleosts examined in parasitological surveys, the higher the number of parasite-host associations that will be identified.

### Accurate identification of fish hosts

During the development of this checklist, we have faced several examples of problematic identification and controversial taxonomy of hosts, which may compromise the reliability of any parasitological survey and limit our understanding of host specificity, the relationship between parasite and host phylogenies, as well as the establishment of trophic links elucidated by life-cycle studies (Naylor et al. 2012). Some genera, such as *Cichla*, *Pimelodus*, *Potamotrygon*, *Pseudoplatystoma* and *Zungaro*, have a convoluted taxonomic history and their species boundaries can diverge depending upon the approach used. Kullander and Ferreira (2006) for instance, recognized 15 species of *Cichla* distributed across South American rivers, based on morphological characters. However, Willis et al. (2012) recognized only eight species using multi-locus genetic data, suggesting that the number of *Cichla* species in South America may have been overestimated.

Therefore, we recommend that parasitologists keep a piece of host tissue in a molecular-grade ethanol for sequencing and to work in synergy with fish taxonomists to be as accurate as possible in fish identification, as already advocated by Naylor et al. (2012) for elasmobranch hosts. Caira and Jensen (2014) provided a field-sampling protocol that may be useful not only for parasite taxonomists, but also for those who are interested in general host-parasite associations.

## Conclusions

Poulin et al. (2016) tested the completeness of 25 checklists of metazoan parasites in vertebrate hosts from several geographic regions based on three approaches. None of the studies analyzed performed well and only three of them passed two of the tests. Several obstacles contribute to a lack of completeness of checklists, including: (1) the reliability of information depends on the accuracy of the description or report; (2) geographically biased studies may not reflect the real distribution of diversity; (3) cryptic species, i.e. genetically distinct species that look similar morphologically, may contribute to an underestimate of the true number of species; and (4) only a small fraction of the potential fish hosts in South America have been examined for parasites. To mitigate these issues, we have attempted to critically gather as much information as possible and have obtained expert opinions. Therefore, we hope that we provide here the most robust database up to date that may help in a reliable estimation of the true diversity of fish cestodes in South America.
